# An extended transcription factor regulatory network controls hepatocyte identity

**DOI:** 10.15252/embr.202357020

**Published:** 2023-07-10

**Authors:** Julie Dubois‐Chevalier, Céline Gheeraert, Alexandre Berthier, Clémence Boulet, Vanessa Dubois, Loïc Guille, Marie Fourcot, Guillemette Marot, Karine Gauthier, Laurent Dubuquoy, Bart Staels, Philippe Lefebvre, Jérôme Eeckhoute

**Affiliations:** ^1^ Univ. Lille, Inserm, CHU Lille, Institut Pasteur de Lille, U1011‐EGID Lille France; ^2^ Basic and Translational Endocrinology (BaTE), Department of Basic and Applied Medical Sciences Ghent University Ghent Belgium; ^3^ Univ. Lille, CNRS, Inserm, CHU Lille, Institut Pasteur de Lille, US 41 – UAR 2014 – PLBS Lille France; ^4^ Univ. Lille, Inria, CHU Lille, ULR 2694 – METRICS: Évaluation des technologies de santé et des pratiques médicales Lille France; ^5^ Institut de Génomique Fonctionnelle de Lyon (IGFL), CNRS UMR 5242, INRAE USC 1370, École Normale Supérieure de Lyon Lyon France; ^6^ Univ. Lille, Inserm, CHU Lille, U1286 – INFINITE – Institute for Translational Research in Inflammation Lille France

**Keywords:** cell identity, core regulatory network, hepatocyte dedifferentiation, liver disease, transcription factors, Chromatin, Transcription & Genomics, Molecular Biology of Disease

## Abstract

Cell identity is specified by a core transcriptional regulatory circuitry (CoRC), typically limited to a small set of interconnected cell‐specific transcription factors (TFs). By mining global hepatic TF regulons, we reveal a more complex organization of the transcriptional regulatory network controlling hepatocyte identity. We show that tight functional interconnections controlling hepatocyte identity extend to non‐cell‐specific TFs beyond the CoRC, which we call hepatocyte identity (Hep‐ID)^CONNECT^ TFs. Besides controlling identity effector genes, Hep‐ID^CONNECT^ TFs also engage in reciprocal transcriptional regulation with TFs of the CoRC. In homeostatic basal conditions, this translates into Hep‐ID^CONNECT^ TFs being involved in fine tuning CoRC TF expression including their rhythmic expression patterns. Moreover, a role for Hep‐ID^CONNECT^ TFs in the control of hepatocyte identity is revealed in dedifferentiated hepatocytes where Hep‐ID^CONNECT^ TFs are able to reset CoRC TF expression. This is observed upon activation of NR1H3 or THRB in hepatocarcinoma or in hepatocytes subjected to inflammation‐induced loss of identity. Our study establishes that hepatocyte identity is controlled by an extended array of TFs beyond the CoRC.

## Graphical abstract

Hepatocyte identity is controlled by interconnected transcription factors (TFs) extending beyond the core regulatory TF network (CoRC). The extended network comprises thyroid hormone receptor beta whose activation resets CoRC TFs expression and hepatocyte identity in dedifferentiated hepatocytes.

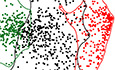

## Introduction

Multicellular organisms are built upon collaborative activities of phenotypically and functionally distinct cell‐types. Individual cell identities and functions are acquired thanks to the activity of cell‐specific transcription factors (TFs). Indeed, cell‐specific TFs directly control the expression of non‐TF genes exerting activities/functions characterizing individual cell‐types, alternatively known as identity effector genes (Arendt *et al*, 2016; Almeida *et al*, 2021). A limited set of identity TFs engaging into auto‐ and cross‐regulatory loops typically defines the core transcriptional regulatory circuitry (CoRC; Arendt *et al*, 2016; Almeida *et al*, 2021). CoRCs are considered cornerstones for establishment and maintenance of cell identities (Arendt *et al*, 2016; Almeida *et al*, 2021). Indeed, CoRCs allows to self‐sustain high expression of identity TFs and their target effector genes in addition to being involved in the continuous modulation of the hepatic transcriptome in response to environmental stimuli (Boyer *et al*, 2005; Arendt *et al*, 2016; Wilkinson *et al*, 2017; Almeida *et al*, 2021).

In line, hepatocyte identity is typically defined as relying on a handful of interconnected hepatocyte‐specific CoRC TFs (hereafter called Hep‐ID TFs) including HNF4A, FOXA or NR1H4 (FXR; Tachmatzidi *et al*, 2021). However, many additional TFs beyond these hepatic identity CoRC TFs have been ascribed with roles in the control of hepatocyte activities (Bideyan *et al*, 2021). However, whether and how these additional TFs exert their functions in concert with the hepatic CoRC is not fully understood. Moreover, considering that sustained expression of Hep‐ID TFs has a positive impact on hepatic pathological conditions (Berasain *et al*, 2022), better defining how CoRC TFs are functionally linked to additional TFs is of pathophysiological interest. Indeed, breakdown in liver functions in advanced stages of liver injuries and in cancer results from hepatocyte loss of identity stemming from compromised expression of the hepatic CoRC TFs (Dubois *et al*, 2020a; Berasain *et al*, 2022).

As outlined hereabove, apprehending the transcriptional regulation of cell identity through the lens of CoRCs, by definition, only leads to consider the role of a very limited subset of TFs. In this context, the real architecture and outline of the hepatocyte identity TF network has not been clearly established beyond Hep‐ID TFs. We postulated that focusing on TF interconnections at the genomic‐scale would allow to define how the Hep‐ID TF network spreads beyond the CoRC and would refine our understanding of hepatocyte identity transcriptional control.

## Results

### Hepatocyte identity TFs extensively co‐bind TF‐encoding gene promoters beyond the CoRC

**Figure 1:**
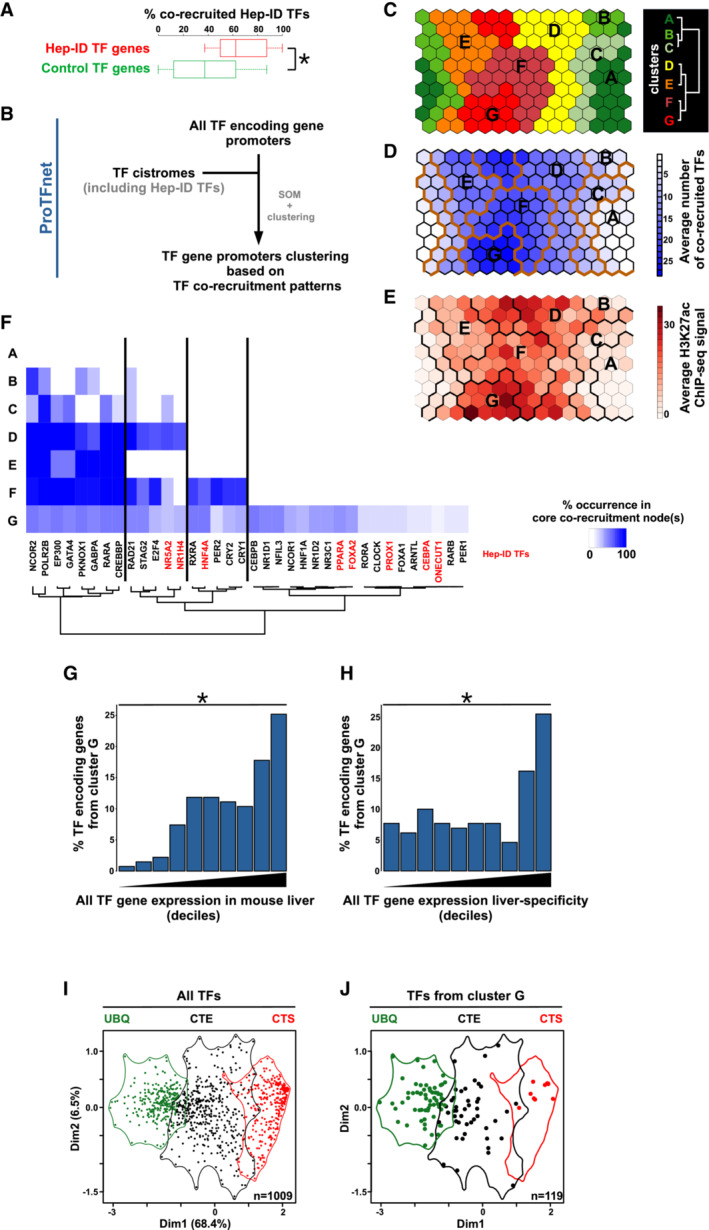
Promoter‐centric mining of the hepatic TF network using ProTFnet AThe cistromes of 8 Hep‐ID TFs (CEBPA, FOXA2, HNF4A, NR1H4, NR5A2, ONECUT1, PPARA, PROX1; Dataset EV2) were used to define the percentage of those TFs binding to Hep‐ID TF (*n* = 13 genes) or non‐Hep‐ID TF (control TF genes; *n* = 13) encoding gene promoters. The control group used was selected for providing data representative of those obtained with 1,000 reiterations of this analysis. Box plots are composed of a box from the 25^th^ to the 75^th^ percentile with the median as a line. Whiskers extent to the most extreme data point which is no more than 1.5 times the interquartile range from the box. Two‐sided Wilcoxon rank sum test with continuity correction was used to assess statistical significance. *P < 0.05.BOverview of the ProTFnet strategy implemented in this study where (identity) TF binding to TF‐encoding gene promoters is monitored and subsequently used to define distinct clusters of promoters through SOM and hierarchical clustering. Clusters are subsequently characterized using multi‐omics (cistromic, epigenomic and transcriptomic) data mining to explore the complexity of the identity TF network.CPlanar view of the toroidal map issued from the SOM analysis was used here to display clusters A–G and hereafter to visualize different features of these clusters (panels D, E and Appendix Fig S2E). The dendrogram issued from the hierarchical clustering analysis is shown on the right.DThe map issued from the SOM analyses was used to show the average number of co‐recruited TFs at gene promoters contained in individual cells. Bold orange lines indicate the borders of clusters A–G.EThe map issued from the SOM analyses was used to show the average ChIP‐seq signal for mouse liver H3K27ac at gene promoters contained in individual cells. Bold black lines indicate the borders of clusters A–G.FHeatmap showing the occurrence (percentage) of individual transcriptional regulators in the core co‐recruitment nodes of clusters A–G, that is, binding combinations found in at least 50% of the promoters of a given cluster.G, H(G) All TF‐encoding genes were grouped into deciles based on increasing expression levels in the mouse liver. Then, the distribution of TF‐encoding genes from cluster G within these deciles was plotted. Two‐sided two‐sample Kolmogorov–Smirnov test was used to assess statistically significant bias in the distribution of genes from cluster G when compared to all other TF‐encoding genes. *P < 0.05. (H) All TF‐encoding genes were grouped into deciles based on increasing liver‐specific expression levels (i.e., expression in mouse liver compared to average expression in other organs). Then, the distribution of TF‐encoding genes from cluster G within these deciles was plotted. Statistical analysis was performed as in panel H.IPrincipal component analysis (PCA) of TF gene expression in mouse (*n* = 39) and human (*n* = 126) primary cell‐types (see Materials and Methods). Individual TFs are displayed as dots projected on the first two components and the three main clusters issued from hierarchical clustering are shown and labeled as UBQ (ubiquitous), CTE (cell‐type enriched) and CTS (cell‐type specific; Fig EV2A and B).JThe data from panel I were used to selectively display TF genes from cluster G.

**Figure EV1:**
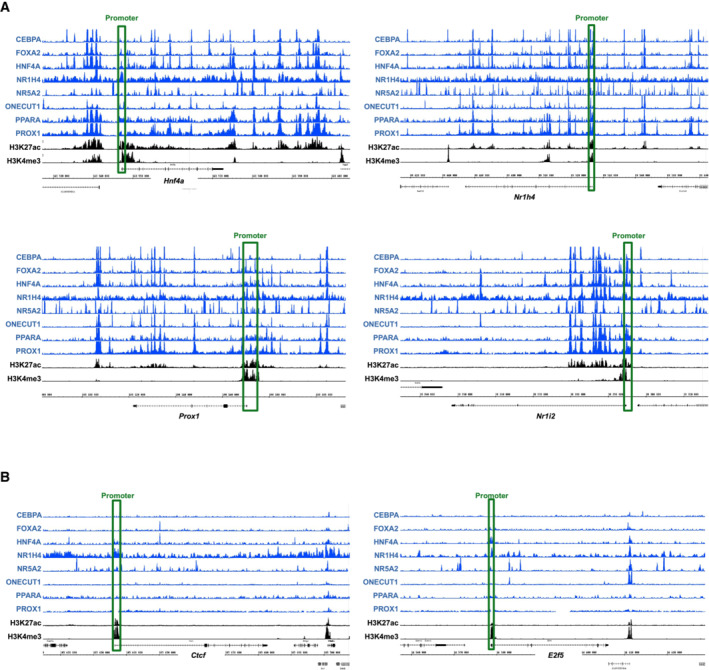
Hep‐ID TF cistromes at example TF‐encoding gene loci A, BThe Integrated Genome Browser (IGB) was used to display the cistromes of the indicated eight Hep‐ID TFs together with levels of H3K4me3 and H3K27ac from mouse liver ChIP‐seq data (Dataset EV2). Example Hep‐ID TF (A) and control TF‐encoding genes (B) are shown. The promoters are highlighted by green boxes. The scales of the individual ChIP‐seq tracks were kept constant for all analyzed genes.

We defined the hepatocyte CoRC as a set of 13 Hep‐ID TFs commonly defined in different studies as interconnected hepatocyte‐specific TFs (Kyrmizi *et al*, 2006; D'Alessio Ana *et al*, 2015; Zhou *et al*, 2017; Dubois *et al*, 2020a; Dataset EV1). Hep‐ID TFs comprise the well‐accepted and thoroughly experimentally verified drivers of hepatocyte identity (Reizel *et al*, 2020; Tachmatzidi *et al*, 2021). As expected from our previous studies (Dubois‐Chevalier *et al*, 2017b, 2020; Dubois *et al*, 2020a), monitoring the mouse liver cistromes of eight Hep‐ID TFs (CEBPA, FOXA2, HNF4A, NR1H4, NR5A2, ONECUT1, PPARA, PROX1; Dataset EV2) pointed to their extensive co‐recruitment at the Hep‐ID TF‐encoding gene promoters when compared to a control set of non‐Hep‐ID TF‐encoding gene promoters (Figs 1A and EV1A and B). This led us to define a strategy called Promoter‐centric TF network analysis (ProTFnet; Fig 1B) with the aim to establish global TF interconnections through promoter binding patterns. Indeed, this approach consists in mining TF cistromes to identify TF binding to all TF‐encoding gene promoters. Here, hepatic TF cistromes (*n* = 49; including those of Hep‐ID TFs and extending to transcriptional cofactors; Dubois‐Chevalier *et al*, 2017b; Dubois *et al*, 2020a) were used to monitor the binding to all TF‐encoding gene promoters active in the mouse liver (*n* = 925 transcriptionally active promoters, i.e., TF‐encoding genes with detectable expression levels; see Materials and Methods for details). These promoters were grouped together based on their TF‐binding pattern similarity, that is, cistromic‐based classification, using a self‐organizing map (SOM; Appendix Fig S1A–D; Dubois‐Chevalier *et al*, 2017a). Hierarchical clustering was next performed and identified seven main clusters of TF‐encoding gene promoters (Fig 1C and Appendix Fig S2A). We annotated these clusters A–G based on progressive TF co‐recruitment pattern complexity (Fig 1C and D, Appendix Figs S2B and C, and S3A–G). Promoters from cluster G were also overall the most strongly active as revealed by chromatin accessibility (DHS), hepatic histone acetylation (H3K27ac) or mRNA expression levels of associated TF‐encoding genes (Fig 1E and Appendix Fig S4A–C). In line, cluster G was enriched for Hep‐ID TF‐encoding genes (odds ratio = 5.2, *P* = 0.006). Moreover, Hep‐ID TF binding was also most pronounced at promoters from cluster G, both when considered individually (Appendix Figs S2D and E, and S3G) or collectively, i.e., Hep‐ID TF co‐recruitment (Fig 1F). Therefore, cluster G is the prominent subset of promoters capturing hepatic CoRC auto−/cross‐binding. Despite being enriched, TF‐encoding genes captured by cluster G were not limited to those displaying the strongest and most liver‐specific expression (Fig 1G and H).

**Figure EV2:**
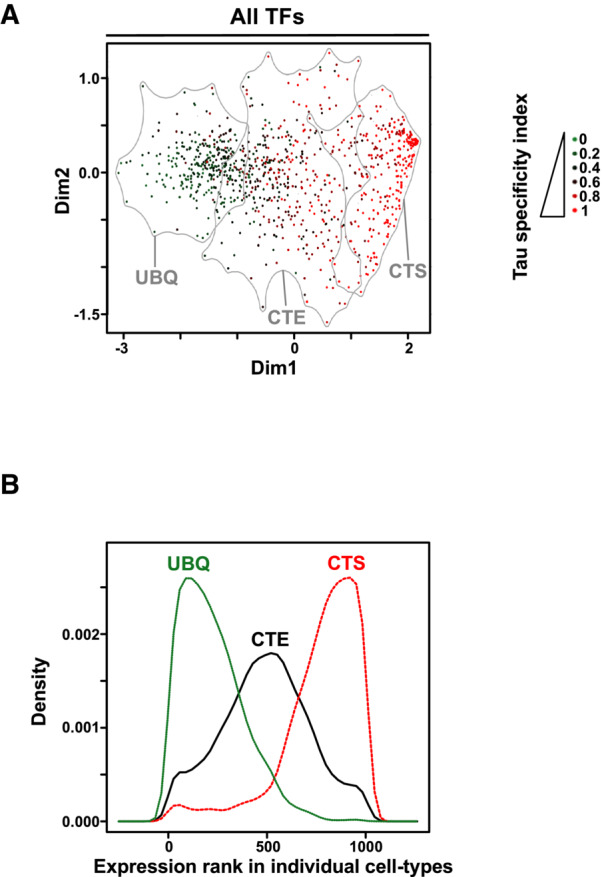
Characterization of CTS, CTE, and UBQ TF genes Data were displayed as in Fig 1J to show the Tau index of tissue‐specific expression for individual TF genes within the CTS (cell‐type specific), CTE (cell‐type enriched), and UBQ (ubiquitous) clusters.Density plot showing the distribution of the expression rank of CTS, CTE, and UBQ TF‐encoding genes in primary mouse cell‐types (*n* = 39). All TF genes were ranked from high to low expression (i.e., from 0 to 1,009 in each cell‐type) and the distribution of TFs from the CTS, CTE and UBQ groups are shown. As expected, CTS TFs display low ranks in a very limited subsets of cell‐types while having high ranks in most cell‐types, which is the opposite from the pattern obtained for UBQ TFs.

To further characterize the TF‐encoding gene heterogeneity in cluster G, we performed a meta‐analysis of transcriptomic data from primary human and mouse cells (*n* = 126 and 39 different cell‐types including hepatocytes, respectively) to define three groups of TFs displaying cell‐type specific (CTS), cell‐type enriched (CTE) or ubiquitous (UBQ) expression patterns (Figs 1I and EV2A and B; see Materials and Methods). This further highlighted that, besides Hep‐ID TFs and an additional set of cell‐specific TFs with well characterized hepatic functions (*Nr1i3*/*Car*, *Rorc*/*Rorg*, *Hnf1a* and *Gata4*; Tachmatzidi *et al*, 2021), the majority of TFs from cluster G belonged to CTE or UBQ TFs (Fig 1J and Dataset EV1).

Altogether, these analyses indicated that extensive co‐recruitment of Hep‐ID TFs is not limited to their own highly hepatocyte‐specific genes within the CoRC but extends to an additional set of TFs.

### Hep‐ID^CONNECT^ TFs are expressed under the control of and collaborate with Hep‐ID TFs in the regulation of hepatocyte identity effector genes

**Figure 2:**
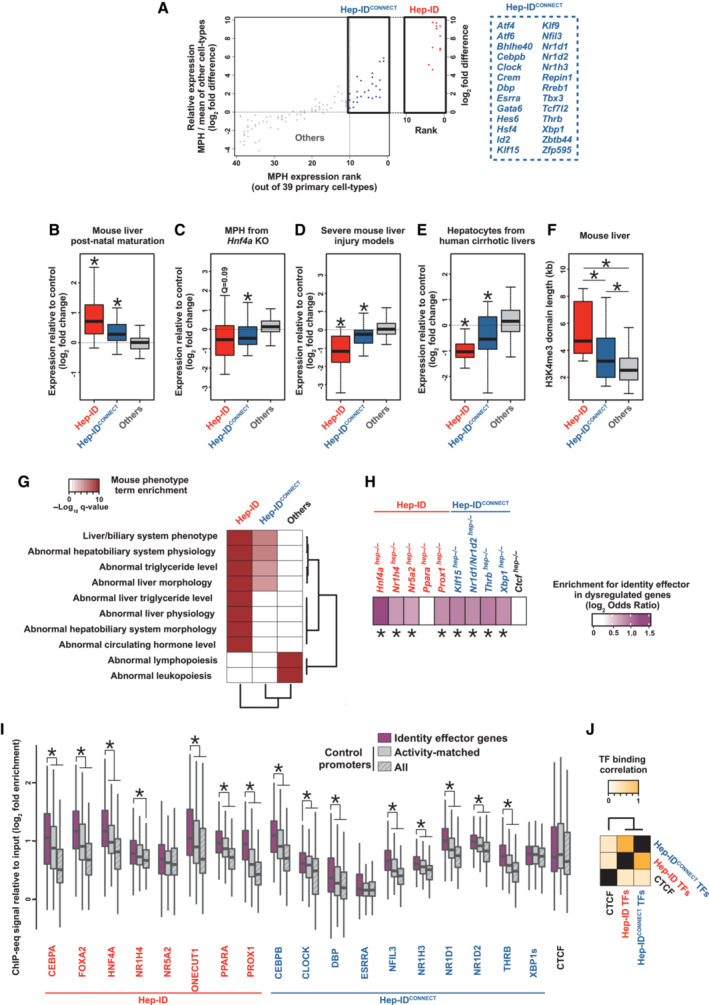
Identification and characterization of Hep‐ID^CONNECT^ TFs AThe CTE/UBQ TFs from cluster G (Fig 1K) were plotted based on their expression in mouse primary hepatocytes (MPH) when compared to other primary mouse cell‐types (*n* = 38). For each individual TF, cells were first ranked according to decreasing gene expression and the rank of MPH was plotted on the *x* axis (i.e., rank 1 indicates highest expression is in MPH). Second, expression in MPH was divided by the average expression in all other primary cells and plotted on the *y* axis as log_2_ fold difference. Hep‐ID^CONNECT^ TF‐encoding genes were defined as those preferentially expressed in MPH (rank ≤ 10 and FC > 0). For comparison, Hep‐ID TF genes were plotted in an additional box on the right of the one highlighting Hep‐ID^CONNECT^ TFs.B–EExpression of Hep‐ID (*n* = 13 genes), Hep‐ID^CONNECT^ (*n* = 26 genes) and remaining TF‐encoding genes from cluster G (Others; *n* = 82 genes) was monitored in indicated transcriptomic data (Dataset EV2). Box plots show log_2_ fold changes between adult *versus* newborn mouse livers (B), MPH of *Hnf4a* ^*hep−/−*^ (*Hnf4a* KO) *versus* wild‐type mice (C), a meta‐analysis of severe mouse liver injuries *versus* control livers (D; see Materials and Methods and Appendix Fig S6) and microdissected hepatocytes from alcohol‐related human liver cirrhosis (alcoholic steatohepatitis) *versus* control livers (E). Box plots are composed of a box from the 25^th^ to the 75^th^ percentile with the median as a line. Whiskers extent to the most extreme data point which is no more than 1.5 times the interquartile range from the box. Statistical significance was assessed using one‐sided Wilcoxon rank sum test with Benjamini–Hochberg correction for multiple testing to determine if the mean log_2_ FC was statistically lower (B, D, E) or higher (C) than 0. *q < 0.05.FDistribution of H3K4me3 domain length at the TSS of Hep‐ID (*n* = 13 genes), Hep‐ID^CONNECT^ (*n* = 26 genes) and remaining TF‐encoding genes from cluster G (Others; *n* = 82 genes) as defined through broad peak calling on mouse liver H3K4me3 ChIP‐seq data. Box plots are composed of a box from the 25^th^ to the 75^th^ percentile with the median as a line. Whiskers extent to the most extreme data point which is no more than 1.5 times the interquartile range from the box. Statistical difference between groups was defined using Kruskal–Wallis with Wilcoxon pairwise comparison tests followed by Benjamini–Hochberg correction for multiple testing correction. *q < 0.05.GMouse phenotypes associated with Hep‐ID, Hep‐ID^CONNECT^ and remaining TF‐encoding genes from cluster G (Others) were defined using ToppCluster. Dendrograms of hierarchical clustering are shown. ToppCluster uses hypergeometric tests and Bonferroni correction.HEnrichment for identity effector genes among the top 1,000 transcriptionally dysregulated genes in the MPH/livers of indicated genetically deficient mice. Log_2_ odds ratios were computed to compare the proportion of dysregulated versus non‐dysregulated genes within identity effector genes or control non‐TF‐encoding genes. Then a two‐sided Fisher exact test was performed to assess if the proportion of dysregulated genes was significantly different within the identity and control gene groups with Benjamini–Hochberg correction. *q < 0.05.IBinding of indicated Hep‐ID and Hep‐ID^CONNECT^ TFs to the promoter of identity effector genes and a control group of non‐TF‐encoding genes of similar size (*n* = 424) was monitored using mouse liver ChIP‐seq data. A control group of non‐TF‐encoding genes (*n* = 424) matched for their promoter mouse liver activity was also used (Appendix Fig S9A and B). The control groups used were selected for providing data representative of those obtained with 1,000 reiterations of this analysis (see Materials and Methods; Appendix Fig S9A and B). The distribution of ChIP‐seq signals is shown using box plots composed of a box from the 25^th^ to the 75^th^ percentile with the median as a line. Whiskers extent to the most extreme data point which is no more than 1.5 times the interquartile range from the box. Pairwise one‐sided Wilcoxon Rank Sum Tests with Benjamini–Hochberg correction was used to define whether the binding at identity effector genes *versus* control genes was significantly greater for each analyzed TF recruitment. *q < 0.05.JCorrelation between Hep‐ID TFs, Hep‐ID^CONNECT^ TFs and CTCF recruitment, as judged through the mining of mouse liver ChIP‐seq data, to identity effector genes. The dendrogram is issued from hierarchical clustering analysis. Source data are available online for this figure.

**Figure EV3:**
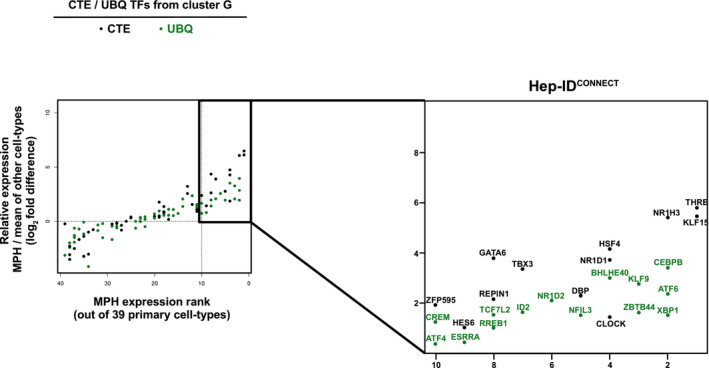
CTE and UBQ TF genes with privileged expression in MPH The right shows a zoomed view of CTE (black) and UBQ (green) TFs from cluster G comprised within the framed area from Fig 2A (shown again on the left).

In order to further characterize the CTE/UBQ TFs from cluster G, we refined our analysis of their individual expression patterns by defining how their levels of expression in mouse primary hepatocytes (MPH) compare to those in other primary cells (*n* = 38 different cell‐types). Interestingly, we found that a subset of the CTE/UBQ TFs from cluster G, hereafter called Hep‐ID^CONNECT^ TFs, was nevertheless characterized by privileged hepatocyte expression (i.e., MPH ranked among the top 10 expressing cells and expression in MPH above average expression in other cells; Figs 2A and EV3). TF‐encoding genes whose ranking was beyond this conservative cut‐off can be retrieved from Table EV1. Reminiscent of Hep‐ID TF‐encoding genes, expression of Hep‐ID^CONNECT^ TFs was increased in the final hepatocyte differentiation stage occurring during liver postnatal maturation (Fig 2B) and mining single‐nuclei RNA‐seq data from adult mouse and human livers confirmed privileged expression in hepatocytes (Appendix Fig S5A and B). These observations suggested that Hep‐ID^CONNECT^ TFs might be directly dependent upon Hep‐ID TFs for their enhanced hepatocyte expression. In line, expression of Hep‐ID^CONNECT^ TFs was decreased in transcriptomic data obtained from MPH of adult mice with hepatocyte‐specific deletion of the Hep‐ID TF *Hnf4a* (Fig 2C). We and others have established that compromised hepatocyte identity due to decreased Hep‐ID TF gene expression is commonly found in severe liver injuries in both mouse models and humans (Argemi *et al*, 2019; Hyun *et al*, 2020; Dubois *et al*, 2020a; Bou Saleh *et al*, 2021; Loft *et al*, 2021; Berasain *et al*, 2022; Gunewardena *et al*, 2022). Therefore, we further mined Hep‐ID^CONNECT^ TF expression in a meta‐analysis of transcriptomic data obtained from mouse liver injury models triggering hepatocyte dedifferentiation (Appendix Fig S6). Interestingly, we found that Hep‐ID^CONNECT^ TFs showed reduced expression levels in the livers of these mouse models (Fig 2D). Similar observations were made using transcriptomic data obtained from microdissected hepatocytes from alcohol‐related human liver cirrhosis (Bou Saleh *et al*, 2021; Fig 2E). Overall, observed changes in expression of Hep‐ID^CONNECT^ TFs were reminiscent of those of Hep‐ID TFs, albeit often with a lower amplitude, and different from those of other TFs from cluster G. A similar observation was made when interrogating the breadth of H3K4me3 labelling at Hep‐ID^CONNECT^ TF gene promoters, a feature positively linked to the role of TFs in the control of cell identity (Pekowska *et al*, 2010; Benayoun *et al*, 2014; Chen *et al*, 2015). Indeed, the breadth of H3K4me3 labeling at the promoters of Hep‐ID^CONNECT^ TF genes was also comprised in between that observed for the Hep‐ID TF and other TF‐encoding genes (Fig 2F). Moreover, H3K4me3 labelling at Hep‐ID^CONNECT^ TF genes displayed intermediate tissue‐specificity when compared to Hep‐ID TF genes, on the one hand, and remaining TF‐encoding genes from cluster G, on the other hand (Appendix Fig S7). Altogether, these data point to the potential importance of Hep‐ID^CONNECT^ TFs in fully differentiated hepatocytes.

Indeed, many Hep‐ID^CONNECT^ TFs have individually, that is, without an identified unifying rationale, been ascribed, at least to some extent, a role in the control of hepatic metabolic activities (Appendix Fig S8). In line, mining genes associated with phenotypes from the Mammalian Phenotype Ontology resource (Smith *et al*, 2005; Chen *et al*, 2007) revealed that, albeit less significant when compared to Hep‐ID TFs, Hep‐ID^CONNECT^ TF‐encoding genes are linked to abnormal liver phenotypes and metabolic functions (Fig 2G). Mining transcriptomic data obtained from the livers of mice with hepatocyte‐specific deletion of individual Hep‐ID^CONNECT^ TF‐encoding genes (hep^−/−^ mice) indicated that this is functionally underlain by a preferential dysregulation of downstream hepatocyte identity effector genes, that is, non‐TF genes with liver‐specific broad H3K4me3 domains defined in Dubois *et al* (2020a) (Fig 2H). While such a bias was also observed in transcriptomic data obtained from the livers of mice deficient for individual Hep‐ID TF genes, deletion of the general chromatin organizer *Ctcf* did not give rise to preferential dysregulation of hepatocyte identity effector genes (Fig 2H). In line, similar to Hep‐ID TFs and different from CTCF, binding of most Hep‐ID^CONNECT^ TFs was stronger at hepatocyte identity effector gene promoters (Fig 2I). We found that preferential binding remained observable when using a more stringent control, that is, promoters with comparable accessibility and activity (activity‐matched control promoters; see Materials and Methods and Appendix Fig S9A and B for further details). This ruled out that chromatin accessibility alone explains preferential binding to Hep‐ID TF gene promoters (Fig 2I). Finally, binding of Hep‐ID and Hep‐ID^CONNECT^ TFs correlated at the promoter of these genes pointing to combinatorial transcriptional regulation (Fig 2J).

Altogether, these data identified that Hep‐ID TFs of the hepatic CoRC target Hep‐ID^CONNECT^ TF genes, and that these two sets of TFs further collaborate to control identity effector genes.

### Hep‐ID^CONNECT^ TFs are reciprocally targeting Hep‐ID TF‐encoding genes to finely tune their expression in homeostatic conditions

**Figure 3:**
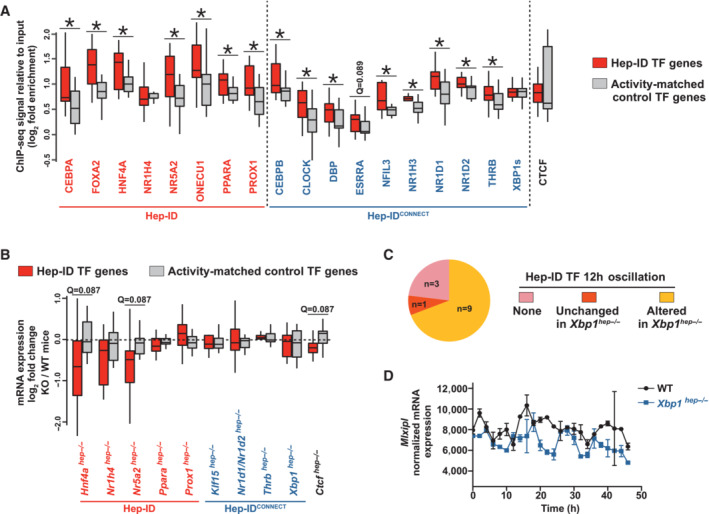
Hep‐ID^CONNECT^ TF binding to and regulation of Hep‐ID TF‐encoding genes in basal conditions Binding of indicated Hep‐ID and Hep‐ID^CONNECT^ TFs to the promoter of Hep‐ID TF genes and a control group of non‐Hep‐ID TF‐encoding genes matched for their promoter activity (Appendix Fig S10A) of similar size (*n* = 13) was monitored using mouse liver ChIP‐seq data. The control group used was selected for providing data representative of those obtained with 1,000 reiterations of this analysis (see Materials and Methods; Appendix Fig S10A). The distribution of ChIP‐seq signals is shown using box plots composed of a box from the 25^th^ to the 75^th^ percentile with the median as a line. Whiskers extent to the most extreme data point which is no more than 1.5 times the interquartile range from the box. One‐sided Wilcoxon rank sum tests with Benjamini–Hochberg correction was used to define whether the binding on Hep‐ID TF gene promoters was greater than on control genes for each individual TF ChIP‐seq dataset. *q < 0.05.Transcriptional modulation of Hep‐ID TFs and a control group of non‐Hep‐ID TF‐encoding genes matched for their promoter activity (Appendix Fig S10A) of similar size (*n* = 13) in mouse liver/MPH of mice deleted for the indicated Hep‐ID or Hep‐ID^CONNECT^ TF genes. The control group used was selected for providing data representative of those obtained with 1,000 reiterations of this analysis (see Materials and Methods; Appendix Fig S10A). The distribution of log_2_ fold changes is shown using box plots composed of a box from the 25^th^ to the 75^th^ percentile with the median as a line. Whiskers extent to the most extreme data point which is no more than 1.5 times the interquartile range from the box. One‐sided Wilcoxon rank sum tests with Benjamini–Hochberg correction was used to define whether log_2_ fold changes for Hep‐ID TF genes were lower than those of the control genes for each individual transcriptomic dataset. *q < 0.05.12 h gene expression oscillation analyses in the mouse liver performed by Meng *et al* (2020) from WT and *XBP1* ^*hep−/−*^ animals were used to identify XBP1‐dependent oscillating expression patterns for Hep‐ID TF genes (Table EV2).Average gene expression levels of *Mlxipl* in the livers of WT and *XBP1* ^*hep−/−*^ mice across circadian time (*n* = 2 mice per group). Error bars show standard deviations. Source data are available online for this figure.

We hypothesized that the intimate connection between Hep‐ID and Hep‐ID^CONNECT^ TFs identified so far could further extend to reciprocal transcriptional regulation where Hep‐ID^CONNECT^ TFs would bind and control expression of Hep‐ID TF‐encoding genes. Mining Hep‐ID^CONNECT^ TFs binding in mouse liver indicated greater recruitment to Hep‐ID TF gene promoters when compared to control activity‐matched TF‐encoding gene promoters, reminiscent of data obtained for Hep‐ID TFs (Fig 3A and Appendix Fig S10A–C). However, this only translated into moderate transcriptional changes in expression of Hep‐ID TF‐encoding genes. Indeed, only the deletion of a subset of the Hep‐ID TF‐encoding genes, and not that of Hep‐ID^CONNECT^ TF genes, individually triggers global down‐regulation of the Hep‐ID TF gene expression (Fig 3B). This most probably relates to the intrinsic role of identity TF networks, which are built‐up to allow robustness of individual cell‐type transcriptional programs (Kyrmizi *et al*, 2006; Almeida *et al*, 2021).

Several Hep‐ID^CONNECT^ TFs are central to establishment of rhythmic hepatocyte gene expression (Appendix Fig S11) including both circadian (BHLHE40, CLOCK, DBP, NFIL3, NR1D1/2; Mukherji *et al*, 2019) and ultradian (XBP1; Meng *et al*, 2020; Pan *et al*, 2020) rhythms. Hence, rather than being instrumental for regulating steady‐state expression levels of Hep‐ID TF genes, we envisioned that Hep‐ID^CONNECT^ TFs might be involved in proper rhythmic expression of Hep‐ID TF‐encoding genes. In line, mining data from Meng *et al* (2020) revealed that a majority of Hep‐ID TF genes (9 out of 13), including for example *Mlxipl*, were characterized by XBP‐1‐dependent ultradian expression patterns (Fig 3C and D, and Table EV2).

### Activation of Hep‐ID^CONNECT^ TFs resets CoRC TF expression and hepatocyte identity in dedifferentiated hepatocytes

**Figure 4:**
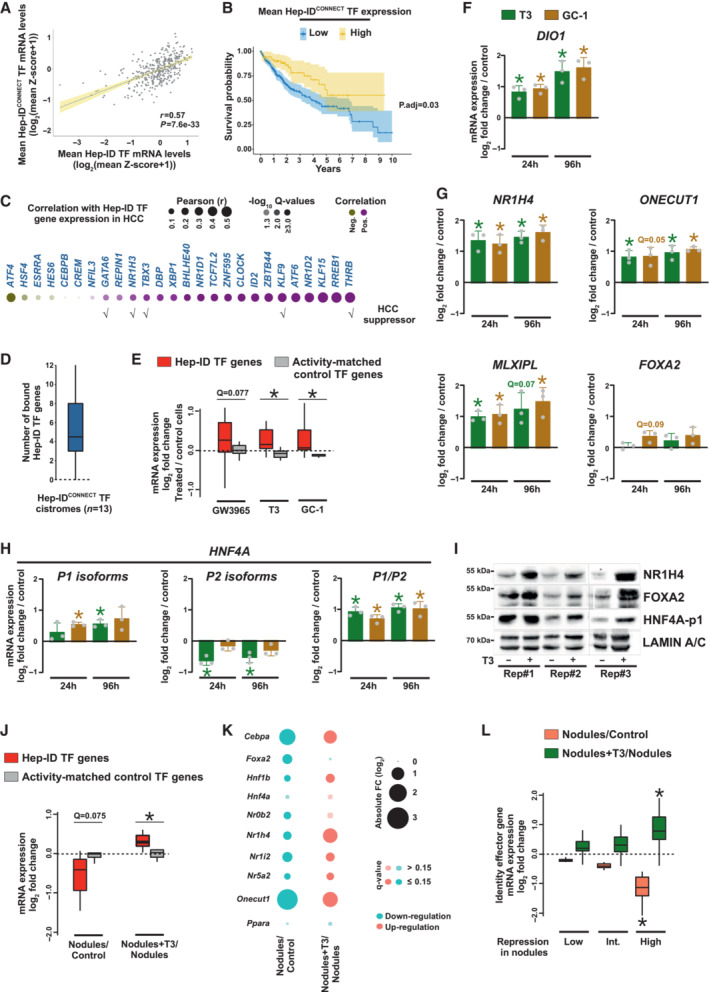
Resetting Hep‐ID TF gene expression through Hep‐ID^CONNECT^ TF activation in hepatocellular carcinoma AComparison of the average expression of Hep‐ID^CONNECT^ and Hep‐ID TF genes in HCC (*n* = 367). The Spearman correlation coefficient *r* and associated *P*‐value is indicated.BOverall survival of patients with HCC expressing low (*n* = 289) or high (*n* = 73) levels of the Hep‐ID^CONNECT^ TF‐encoding genes. Differential overall survival analysis was assessed by Kaplan–Meier (KM) log rank adjusted for 100 permutations (Cheng *et al*, 2022).CAnalyses similar to that described in panel A using individual Hep‐ID^CONNECT^ TF genes. Hep‐ID^CONNECT^ TFs which have been ascribed with HCC suppressive functions in the literature are indicated at the bottom.DHep‐ID^CONNECT^ TF cistromes in HepG2 cells (*n* = 13 independent cistromes) were mined for binding to Hep‐ID TF‐encoding genes. Peaks localized ± 10 kilobases from transcriptional start sites were considered in these analyses.ETranscriptional modulation of Hep‐ID TFs (*n* = 13 genes) and a control group of non‐Hep‐ID TF‐encoding genes matched for their promoter activity (Appendix Fig S10A) of similar size (*n* = 13 genes) in Huh7 or HepG2 cells treated with GW3965, T3 or GC‐1, respectively. The control group used was selected for providing data representative of those obtained with 1,000 reiterations of this analysis (see Materials and Methods; Appendix Fig S10A). The distribution of log_2_ fold changes is shown using box plots. Kruskal–Wallis with two‐sided Wilcoxon pairwise comparison tests followed by Benjamini–Hochberg correction was used to define whether transcriptional regulation of Hep‐ID TF genes was different from that of the control genes for each individual transcriptomic dataset. *q < 0.05.F–HmRNA expression of the indicated genes was monitored using RT‐qPCR in HepG2 cells treated with T3 or GC‐1 for 24 h or 96 h. Bar graphs show mean ± SD (*n* = 3 biological replicates) of log_2_ fold changes in treated *versus* untreated HepG2 cells. For *Hnf4a*, the log_2_ fold change in the ratio of P1 over P2 promoter‐derived isoforms is also shown. Gray dots show the results obtained from the three independent biological replicates (each performed in technical triplicates). One‐sample *t*‐test with Benjamini–Hochberg correction for multiple testing was used to determine if the mean log2 FC was statistically different from 0. *q < 0.05.IWestern blots assays performed using antibodies against the indicated proteins on extracts from HepG2 cells treated or not with T3 for 24 h. Rep#1–3 indicates the three independent biological replicates analyzed.JModulation of Hep‐ID TF gene expression in precancerous nodules compared to control rat livers and in liver nodules of rats treated with T3 compared to nodules of non‐treated rats. A control group of non‐Hep‐ID TF‐encoding genes matched for their promoter activity (Appendix Fig S10A) of similar size (*n* = 13) is also shown. The control group used was selected for providing data representative of those obtained with 1,000 reiterations of this analysis (see Materials and Methods; Appendix Fig S10A). Box plots show the log_2_ fold changes. Two‐sided Wilcoxon rank sum tests with Benjamini–Hochberg correction was used to define whether transcriptional regulation of Hep‐ID TF genes was different from that of the control genes for each individual transcriptomic dataset. *q < 0.05.KDot plots showing the transcriptional regulation of individual Hep‐ID TF gene expression in precancerous nodules compared to control rat livers (left; Nodules/Control) and in liver nodules of rats treated with T3 compared to nodules of non‐treated rats (right; Nodules + T3/Nodules). No data were recovered for *Onecut2*, *Prox1* and *Mlxipl*.LIdentity effector genes significantly downregulated in Nodules/Control (q < 0.05) were split in three groups according to their log_2_ fold changes (i.e., low, intermediate, and high repression; pink boxes) and then monitored for induction in Nodules + T3/Nodules (green boxes). Statistical differences between the High repression and the other groups regarding the Nodules/Control comparison, on the one hand, or the Nodules + T3/Nodules comparison, on the other hand, were defined using Kruskal–Wallis with two‐sided Wilcoxon pairwise comparison tests followed by Benjamini–Hochberg correction for multiple testing correction. *q < 0.05. Data information: Box plots in panels D, E, and J are composed of a box from the 25^th^ to the 75^th^ percentile with the median as a line. Whiskers extent to the most extreme data point which is no more than 1.5 times the interquartile range from the box. Source data are available online for this figure.

**Figure EV4:**
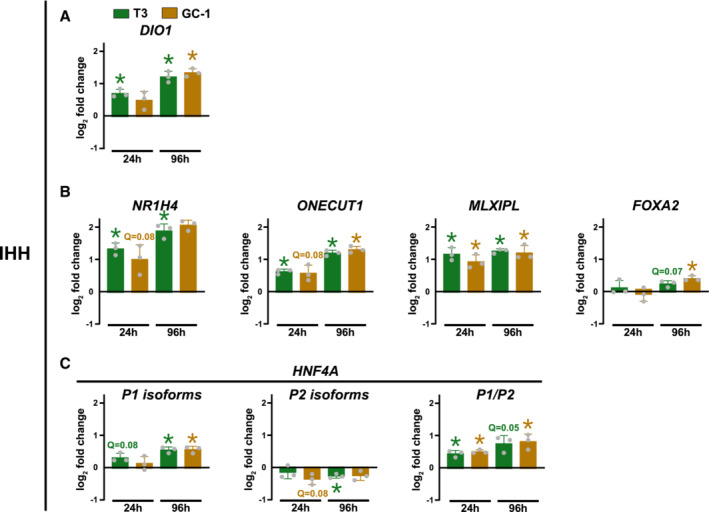
T3‐mediated regulation of Hep‐ID TF gene expression in human IHH cells A–CmRNA expression of the indicated genes was monitored using RT‐qPCR in IHH cells treated with T3 or GC‐1 for 24 or 96 h. Bar graphs show mean ± SD (*n* = 3 biological replicates) of log_2_ fold changes in treated *versus* untreated HepG2 cells. For *Hnf4a*, the log_2_ fold change in the ratio of P1 over P2 promoter‐derived isoforms is also shown. Gray dots show the results obtained from the three independent biological replicates. One‐sample *t*‐test with Benjamini–Hochberg correction for multiple testing was used to determine if the mean log_2_ FC was statistically different from 0. *q < 0.05.

Recent studies have found that TF activities may only be fully revealed in non‐basal conditions and that TFs may be important to (re‐)establish rather than to maintain cell‐specific transcriptomes (Hunter *et al*, 2020; Lo *et al*, 2022). Therefore, we envisioned that a role for Hep‐ID^CONNECT^ TFs in controlling the Hep‐ID TF network might be revealed in a biological context where hepatocyte identity is challenged such as cancer. Mining transcriptomic data of human hepatocarcinoma (HCC) from the cancer genome atlas (TCGA; *n* = 367) indicated a positive correlation between expression of Hep‐ID^CONNECT^ and Hep‐ID TF‐encoding genes (Fig 4A). Moreover, similar to Hep‐ID TFs, high expression of Hep‐ID^CONNECT^ TF genes was linked to better overall survival (Fig 4B and Appendix Fig S12A) and to a trend towards lower cancer grades (Appendix Fig S12B). Expression of many individual Hep‐ID^CONNECT^ TFs was positively correlated with that of Hep‐ID TF genes (Fig 4C) and binding of Hep‐ID^CONNECT^ TFs to Hep‐ID TF genes was also observed in hepatic cancer cells (Fig 4D). Moreover, overexpression or activation of several Hep‐ID^CONNECT^ TFs, GATA‐binding protein 6 (GATA6), krüppel‐like factor 9 (KLF9), T‐box transcription factor 3 (TBX3) and the nuclear receptors liver X receptor alpha (NR1H3 also known as LXR alpha) and thyroid hormone receptor beta (THRB), has been shown to suppress hepatocarcinogenesis (Fig 4C; Sun *et al*, 2014; Tan *et al*, 2019; Kowalik *et al*, 2020; Lin *et al*, 2020; Liang *et al*, 2021). Survival analyses indicated better overall survival of patients with HCC expressing high levels of *KLF9* and *THRB* (Appendix Fig S12C). Based on all this information, we hypothesized that the HCC suppressive effects of Hep‐ID^CONNECT^ TFs may be linked to a resetting of Hep‐ID TF expression. Therefore, we leveraged transcriptomic analyses performed on HCC cell‐lines treated with ligands activating NR1H3 (Huh7 cells treated with GW3965; Vazquez Salgado *et al*, 2022) or THRB (HepG2 cells treated with 3,3′,5‐triiodo‐L‐thyronine (T3) or GC‐1; Yuan *et al*, 2012). In line with our hypothesis, we found that treatment with GW3965, T3, or GC‐1 induced a global increase in Hep‐ID TF gene expression in HCC cell‐lines (Fig 4E). This was linked to the ability of these ligands to interfere with HCC cell‐line growth *in vitro* (Appendix Fig S12D). Induction of Hep‐ID TF gene expression by T3 and GC‐1 in HepG2 cells was independently reproduced as judged using RT‐qPCR analyses, induction of *DIO1* being used as a positive control in these assays (Fig 4F and G). In addition, THRB activation also increased the ratio of HNF4A adult promoter P1‐ to embryonic promoter P2‐derived isoforms (Fig 4H), a phenomenon known to promote hepatocyte differentiation and function (Dubois *et al*, 2020b). Western blot assays confirmed T3‐induced changes in Hep‐ID TF levels (Fig 4I). Similar data were obtained using a non‐cancerous human immortalized hepatic cell‐line (IHH) used as another model of partially dedifferentiated hepatocytes (Schippers *et al*, 1997; Fig EV4A–C). Moreover, interrogating the transcriptomic data obtained in a rat model of hepatocarcinogenesis (Kowalik *et al*, 2020) revealed that T3 was able to counteract the cancer‐driven loss of expression of Hep‐ID TFs (Fig 4J). Indeed, T3 treatment led to robust transcriptional induction of Hep‐ID TF‐encoding genes whose expression was most strongly down‐regulated in the cancerous nodules (Fig 4K). This was in contrast with T3 not triggering a global rise in Hep‐ID TF expression in healthy mice (Appendix Fig S13; Singh *et al*, 2018) further substantiating a role for THRB in controlling Hep‐ID TF genes only in context of dedifferentiated hepatocytes. T3‐mediated re‐expression of Hep‐ID TFs further translated into re‐expression of the most strongly downregulated identity effector genes consistent with concomitant re‐establishment of a transcriptional program closer to that of differentiated hepatocytes (Fig 4L).

Recent studies have revealed a role for T3 in promoting acquisition of a hepatocyte‐like phenotype from pluripotent stem cells (Ma *et al*, 2022), defining that T3‐mediated effects towards promoting a mature hepatocyte cell‐state are not limited to cancer cells. We could not however observe any protective role for T3 treatment with regards to the loss of Hep‐ID TF gene expression spontaneously occurring when primary mouse hepatocytes (MPH) are cultured *in vitro* (Ploton *et al*, 2018; Seirup *et al*, 2022; Appendix Fig S14). This is most probably linked to the immediate and simultaneous drastic changes occurring in MPH, including at the chromatin structure level, which characterize this dedifferentiation model and make it inherently difficult to intervene with (Seirup *et al*, 2022). Indeed, previous reports indicated that MPH dedifferentiation could only be partially inhibited when using combined inhibition of multiple dedifferentiating signaling pathways (Sun *et al*, 2019; Xiang *et al*, 2019) or through forced expression of supra‐physiologic amounts of the Hep‐ID TF HNF4A (Nishikawa *et al*, 2015; Tafaleng *et al*, 2021; Yang *et al*, 2021). Another complementary explanation may come from the rapid decrease in *Thrb* expression which accompanies MPH dedifferentiation (Appendix Fig S14). Therefore, we next aimed to evaluate if T3 could modulate hepatocyte dedifferentiation in a non‐cancerous setting *in vivo*.

**Figure EV5:**
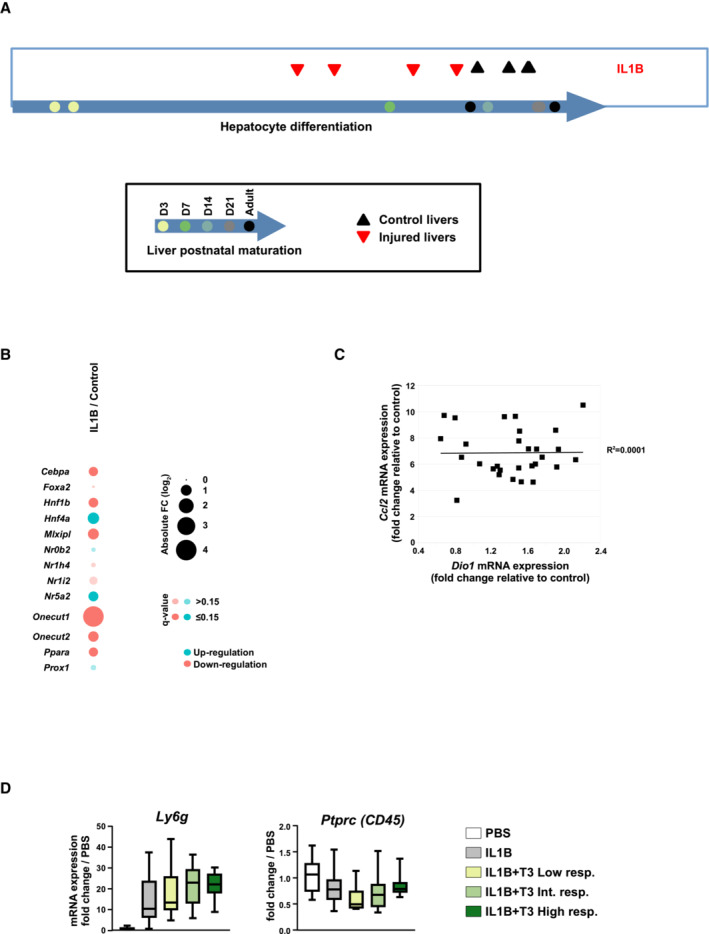
Acute IL1B challenge triggers partial hepatic loss of identity Analysis similar to that shown in Appendix Fig S6 showing that IL1B treatment induced a hepatic transcriptomic profile leaning towards that of not fully mature hepatocytes pointing to partial dedifferentiation.Dot plots showing the transcriptional regulation of individual Hep‐ID TF gene expression in livers of IL1B‐challenged mice compared to non‐treated animals issued from transcriptomic analyses.Correlation between *Dio1* and *Ccl2* mRNA expression levels assessed using RT‐qPCR and livers of all mice treated with IL1B + T3 from Fig 5. Gene expression are log_2_ FC relative to control (PBS injected) mice. Linear regression and coefficient of determination (*r* ^2^) are shown.Gene expression levels of *Ly6g* (neutrophil marker) and *Ptprc* (also known as *CD45*; broad immune cell marker) were analyzed as described for Fig 5B–D. Box plots are composed of a box from the 25^th^ to the 75^th^ percentile with the median as a line (*n* = 13 mice for the PBS group, 17 for the IL1B group and 10 for the other groups). Whiskers extent to the most extreme data point which is no more than 1.5 times the interquartile range from the box.

**Figure 5:**
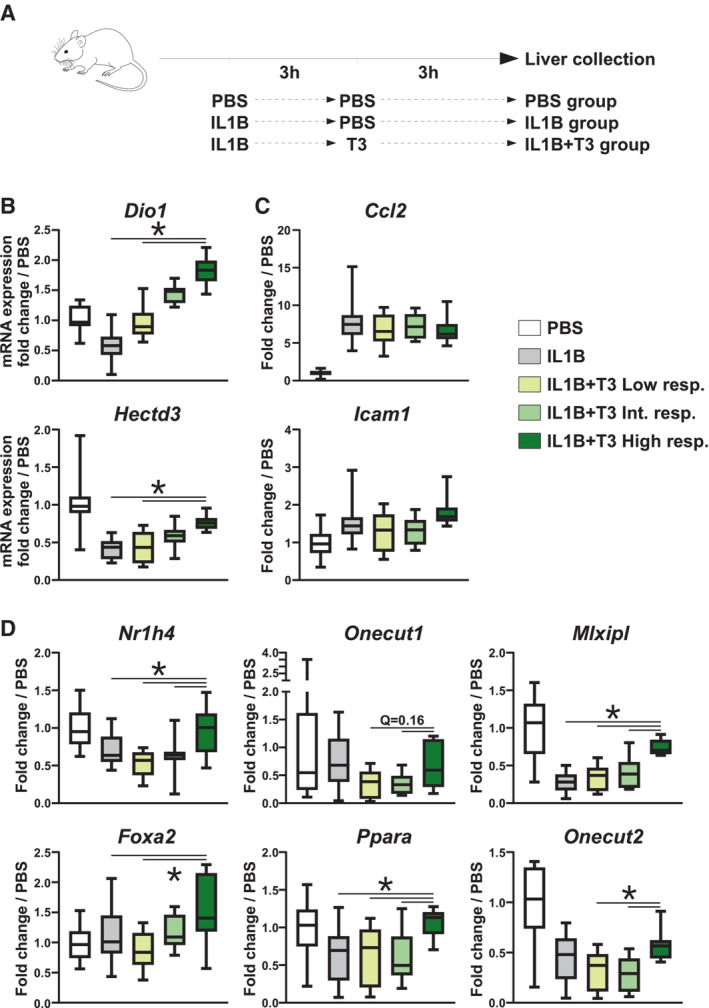
Resetting Hep‐ID TF gene expression through THRB activation in inflammation‐induced hepatocyte dedifferentiation AExperimental protocol for acute inflammation‐induced loss of hepatocyte identity *in vivo*. Mice were injected with IL1B (IL1B; *n* = 17), IL1B followed by T3 (IL1B + T3; *n* = 30) or a control group (PBS; *n* = 14). All livers were collected 6 h after the initial injection.B–DmRNA expression of the indicated genes was monitored in mouse livers using RT‐qPCR. Mice treated with IL1B + T3 were subdivided into tertiles based on the mean expression of the *Dio1* and *Hectd3* genes and defined as low, intermediate or high T3 responsiveness groups (Low resp., Int. resp. and High resp., respectively). Fold change relative to the mean of the control group is shown using box plots composed of a box from the 25^th^ to the 75^th^ percentile with the median as a line (*n* = 13 mice for the PBS group, 17 for the IL1B group and 10 for the other groups). Whiskers extend to the maximum and minimum values. Statistical differences between the High resp. and the other IL1B‐treated groups were defined using Kruskal–Wallis with two‐sided Wilcoxon pairwise comparison tests followed by Benjamini–Hochberg correction for multiple testing correction. *q < 0.05. Source data are available online for this figure.

As stated earlier, we and others have reported that hepatocyte partial loss of identity is a hallmark of diseased livers [recently reviewed in (Berasain *et al*, 2022)]. Indeed, inflammatory cytokines such as tumor necrosis factor or IL1B are well‐established common drivers of hepatocyte dedifferentiation and dysfunction (Del Campo *et al*, 2018; Hyun *et al*, 2020). In this context, we set‐up an experimental model where mice were injected with interleukin 1 beta (IL1B) for 3 h before livers were collected. IL1B treatment indeed triggered loss of hepatocyte identity including decreased expression of several Hep‐ID TF genes as assessed through transcriptomic analyses (Fig EV5A and B). We next determined whether T3 treatment promoted the recovery of Hep‐ID TF gene expression in this model, that is, mice were first injected with IL1B and 3 h latter with or without T3 for an additional 3 h (Fig 5A). This acute setting allowed us to investigate the effect of T3 independent of its previously described pro‐regenerative activities through modulation of hepatocyte proliferation (Tang *et al*, 2022). Inbred laboratory mice raised in standardized conditions nevertheless keep displaying high phenotypic trait variability (Gartner, 1990; Tuttle *et al*, 2018) and are prone to polyphenism, that is, several discrete phenotypes on the same genetic background (Dalgaard *et al*, 2016; Yang *et al*, 2022). Here, mice were subdivided into three groups (low, intermediate and high responsiveness groups) based on their response to T3 as judged using stimulation of the *Dio1* and *Hectd3* gene expression, which are previously identified THRB hepatic target genes (Fig 5B; Paquette *et al*, 2011). Importantly, these differences were due to variability in the T3 response only, since IL1B‐mediated induction of pro‐inflammatory genes was similar between the three groups and no negative correlation between T3 and IL1B responses was detected (Figs 5C and EV5C). We also ruled out any major difference in immune cell infiltration as a potential confounder in these analyses (Fig EV5D). Interestingly, the IL1B + T3 high responsiveness group showed expression recovery of Hep‐ID TF genes whose levels were higher in this group when compared to those in the IL1B only and/or IL1B + T3 low responsiveness groups (Fig 5D).

Altogether, these data have identified the Hep‐ID^CONNECT^ TF THRB as an actionable target to reset expression of hepatic identity TF genes in partially dedifferentiated hepatocytes.

## Discussion

Our promoter‐centric and multi‐omics approach called ProTFnet allowed us to unveil the complexity of the TF network controlling hepatocyte identity and functions. Using this approach, we identified that Hep‐ID TFs of the CoRC are tightly connected with an additional layer of TFs through reciprocal co‐recruitment to their promoters. The CoRC, in addition to being instrumental for cell identity establishment and maintenance, comprises TFs which are also key for controlling and adapting mature cell functions to homeostatic requirements. In this context, the tight connection between Hep‐ID^CONNECT^ TFs and the CoRC provides an additional layer of regulation to finely tune Hep‐ID TF expression. For instance, Hep‐ID^CONNECT^ TFs comprise regulators of rhythmic gene expression including XBP1. Other Hep‐ID^CONNECT^ TFs, TBX3 and TCF7L2, have been identified as regulators of zonated hepatocyte transcriptional programs (preprint: González‐Blas *et al*, 2022) pointing to a role of Hep‐ID^CONNECT^ TFs in specifying proper hepatic gene expression in both space and time. Importantly, rhythmic and zonated hepatic gene expression are crucial to maintain appropriate liver metabolic and non‐metabolic activities (Mukherji *et al*, 2019; Meng *et al*, 2020; Pan *et al*, 2020; Paris & Henderson, 2022).

**Figure 6:**
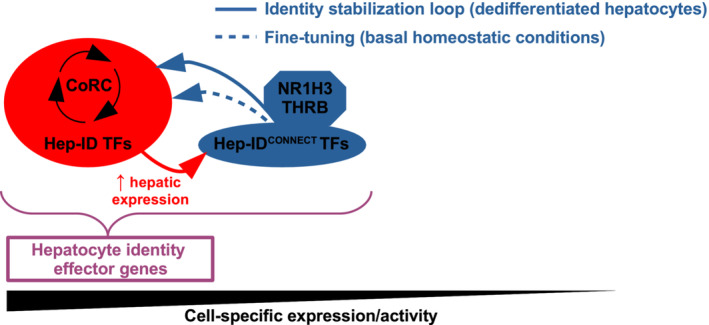
Proposed model for the control of hepatocyte identity by an extended transcription factor network Schematic summarizing the main findings of our study pointing to an extended hepatic TF identity network which includes THRB. See Discussion for greater details.

While not being required to sustain Hep‐ID TF gene expression and cell identity in basal condition, our data obtained with NR1H3 and THRB in dedifferentiated hepatocytes revealed that Hep‐ID^CONNECT^ TF activation can be leveraged to reset the CoRC and hepatocyte identity. These findings are consistent with other recent observations where TF binding to cell‐specific target genes translates into a more critical role in (re)establishment and dynamic regulation rather than maintenance of steady‐state gene expression levels (Lo *et al*, 2022). Our study is also consistent with recent insights into the understanding of cell fate choice, which have suggested a more distributed form of control relying on combinatorial activities of dozens of TFs rather than an organization strictly dominated by a few master TFs (Chubb *et al*, 2021; Mittnenzweig *et al*, 2021). In this context, we propose that Hep‐ID^CONNECT^ TFs are involved in identity stabilization loops, whose functional importance with regards to cell identity is revealed in pathophysiological situations of dedifferentiation (Fig 6). Context‐dependent activities are an intrinsic property of TFs and precisely defining how Hep‐ID^CONNECT^ TFs sense the cellular state to adapt their functions will require additional studies.

Loss of hepatocyte identity is a main feature of tumorigenesis, but is now ascribed a broader pathophysiological relevance including loss of activity of severely injured livers (Berasain *et al*, 2022). In both situations, resetting hepatocyte identity through re‐expression of CoRC TFs is considered of potential therapeutic interest (Chao *et al*, 2020; Berasain *et al*, 2022). By identifying the Hep‐ID^CONNECT^ TF‐controlled identity stabilization loops, our study has identified new avenues to achieve this goal. In particular, our data with agonists of the nuclear receptors NR1H3 and THRB are particularly relevant as several of those are being considered for treatment of liver diseases (Russo‐Savage & Schulman, 2021; Hatziagelaki *et al*, 2022). With regard to HCC, our data provide explanation for the reported beneficial effects exerted by NR1H3 and THRB. Many Hep‐ID^CONNECT^ TFs, including NR1H3 and THRB, do not display typically used features to define hepatocyte identity TFs such as hepatocyte‐specific expression and association with super‐enhancers (Hnisz *et al*, 2013; Dubois *et al*, 2020a; Table EV3). Hence, our study has allowed to uncover an unforeseen role for an extended transcriptional regulatory network beyond the CoRC in the control of hepatocyte identity (Fig 6).

## Materials and Methods

### Data retrieval

Public functional genomics data used in this study were downloaded from the Gene Expression Omnibus (GEO; Barrett *et al*, 2013), ArrayExpress (Sarkans *et al*, 2021), ENCODE (Yue *et al* 2014), the UCSC Genome Browser (Raney *et al* 2011), FANTOM5 (Lizio *et al*, 2015), or from BioGPS (Wu *et al* 2009) and are listed in Dataset EV2.

Hep‐ID TFs were defined as hepatocyte‐identity TFs retrieved in at least two out of three independent studies (D'Alessio Ana *et al*, 2015; Zhou *et al*, 2017; Dubois *et al*, 2020a). The top 20 TFs were used for (D'Alessio Ana *et al*, 2015).

Mouse liver transcriptionally active promoters, that is, giving rise to detectable gene transcription, were defined by Wang and colleagues based on Precision nuclear Run‐On Sequencing (PRO‐Seq) data (Wang *et al*, 2018). HepG2 active promoters were defined as the transcriptional start sites (TSSs) from Gencode v32 (Frankish *et al*, 2018) with H3K27ac ChIP‐seq signal (ENCODE, Dataset EV2) with at least twofold enrichment over control within a 1 kilobase (± 500 bp of TSS) window. Only the most active TSS per gene, that is, highest H3K27ac ChIP‐seq signal, were kept.

The IDR thresholded peaks issued from ChIP‐seq analyses of flag‐tagged Hep‐ID^CONNECT^ TFs (*n* = 14) in HepG2 cells were obtained from ENCODE (Dataset EV2).

Tau indexes of tissue‐specific expression were retrieved from (Kryuchkova‐Mostacci & Robinson‐Rechavi, 2017).

### Self‐organizing maps (SOM) analyses

Active promoters in the mouse liver were defined as *cis*‐regulatory modules, that is, genomic regions bound by at least two different transcriptional regulators (Appendix Fig S1A and B) as in (Dubois‐Chevalier *et al*, 2017a), which overlap active TSSs identified as PRO‐seq data summits in (Wang *et al*, 2018). Non‐unique associations between TSSs and genes in the PRO‐seq data were discarded. Only transcription factor (TF) encoding genes, retrieved using a manually curated gene list originally obtained from the AnimalTFDB2.0 database (Zhang *et al*, 2015), were considered. Transcriptional regulator co‐recruitment analyses made use of mouse liver ChIP‐seq data for 49 factors (Dataset EV2), which were uniformly processed including quality control checks as described in Dubois‐Chevalier *et al* (2017a). All these data were obtained using the liver of untreated adult mice. In line with the vast majority of the liver chromatin stemming from hepatocytes (which are the prominent cell‐type in the liver and are, moreover, mostly polyploid cells – up to 85% in mice with mainly tetraploid hepatocytes; Duncan *et al*, 2010), hepatic TF cistromes can be faithfully inferred from mouse liver ChIP‐seq data (Kyrmizi *et al*, 2006; Schmidt *et al*, 2010; Dubois‐Chevalier *et al*, 2017a; Sommars *et al*, 2019; Dubois *et al*, 2020a).

The SOM were generated using the R package kohonen2 (Wehrens & Buydens, 2007) as described in Dubois‐Chevalier *et al* (2017a). Cells containing promoters with similar transcriptional regulator‐binding patterns (Appendix Fig S1C and D) were further grouped into clusters (denoted A–G) based on hierarchical clustering performed using the hclust function of the R package Stats (R Core Team, 2015). We used the Ward agglomeration method and the best representative transcriptional regulator combination (prototype) for each individual cell. The number of clusters was chosen according to homogeneity analyses (http://lastresortsoftware.blogspot.fr/2010/08/homogeneity‐analysis‐of‐hierarchical.html; Bedward *et al*, 1992) and biological significance. A planar projection of the toroidal map was used for data visualization.

### Analyses of transcriptional regulator co‐recruitment patterns in SOM‐derived clusters

Transcriptional regulator co‐occurrence in clusters A–G was used to calculate Tanimoto distance matrices, which were used to draw heatmaps and perform hierarchical clustering or perform multi‐dimensional scaling (MDS) analyses using R (R Core Team, 2015) as described in Dubois‐Chevalier *et al* (2017a). To analyze the combinations of transcriptional regulators bound at the different promoters of a given cluster, a “frequent itemsets” search (combinations of 2–49 regulators) was performed using the arules R package (Hahsler *et al*, 2005). Only itemsets occurring in at least 50% of promoters of a given cluster were considered and defined as the core co‐recruitment nodes. Finally, the percentage of occurrence of each transcriptional regulator in these nodes was retrieved.

### Transcriptomic data analyses

#### Processing of raw data and differential gene expression analyses

Raw transcriptomic data from Affymetrix microarrays were normalized on a local instance of Galaxy (Afgan *et al*, 2018) using the GIANT APTtool (Affymetrix Power Tools; www.thermofisher.com/fr/fr/home/life‐science/microarray‐analysis/microarray‐analysis‐partners‐programs/affymetrix‐developers‐network/affymetrix‐power‐tools.html; with options: gc correction when available, scale intensity and rma at probeset level) from the GIANT toolbox (Vandel *et al*, 2020). Normalized expressions were averaged per Gene Symbol (NetAffx Annotation Release 36, July 2016). Raw transcriptomic data from Agilent microarrays were processed with the limma R package (Ritchie *et al*, 2015) used to normalize the data through the “backgroundCorrect” function (parameters used were method: “normexp” and normexp.method: “rma”) and to filter out low expressed probes. Normalized data from Illumina Bead Chips were retrieved from the Gene Expression Omnibus database and annotated using GPL6101_Illumina_RatRef‐12_V1_0_R1_11222119. Orthologous mouse genes were retrieved using Ensembl annotations (release 105; Howe *et al*, 2021). Differential expression analyses were performed with GIANT using the limma tool (FDR cutoff set at 0.15).

RNA‐seq data were analyzed using the Galaxy web platform (Afgan *et al*, 2018). Mapping of reads on mm10 was performed with HISAT2 (version 2.21; options: default; Kim *et al*, 2019). Mapped reads mapping to exons were subsequently retrieved and merged by gene_id with Htseq‐count (version 0.9.1; Anders *et al*, 2015) using the mm10 annotation of Ensembl (release 102; Yates *et al*, 2020; with the following options, mode: union, minimum alignment quality: 10, “do not count non uniquely or ambiguously mapped reads,” stranded: no). Normalization and differential analyses were performed with EdgeR (version 3.36.0; Robinson *et al*, 2010; Liu *et al*, 2015; options: lowly expressed genes filtered out, cutoff: < 1 CPM in *n* samples—with *n* corresponding to number of biological replicates in one condition, FDR < 0.15, normalization method: TMM, Robust settings: True). Normalized gene expressions were obtained by averaging data per Gene Symbol using the Ensembl annotation (release 105; Howe *et al*, 2021). Dot plots displaying dysregulation in transcriptomic data were performed with the ggplot2 R package (Wickham, 2016).

#### Analysis of mouse liver TF‐encoding gene expression

Normalized read counts in gene bodies obtained using PRO‐Seq (Wang *et al*, 2018) were used to define TF gene expression in the mouse liver. Gene expression liver specificity was computed using data from the BioGPS Mouse MOE430 Gene Atlas (Wu *et al*, 2016) by dividing the normalized expression in the liver by the average normalized expression in other tissues (Dataset EV2). In both instances, TF‐encoding genes were finally separated into deciles of increasing mouse liver (specific) expression.

#### Mining of CAGE‐seq data to define CTS (cell‐type specific), CTE (cell‐type enriched), and UBQ (ubiquitous) TFs

CAGE‐seq data from FANTOM5 (Lizio *et al*, 2015) were used to define CTS, CTE and UBQ TF‐encoding genes based on their patterns of expression in primary human and mouse cells. Human and mouse orthologous genes were defined using the Ensembl annotation (version 105; Howe *et al*, 2021). Only TF‐encoding genes retrieved in both the human and mouse CAGE‐seq data (*n* = 1,009) were considered. Their expression levels in mouse and human cells were stacked in a unique matrix centered and scaled, which was used to perform a Multiple Factor Analysis (MFA) using the MFA function of the R package FactoMineR (Lê *et al*, 2008) considering expression in mouse and human cells as two distinct groups of variables and TFs as individuals (parameters were set as follows, type: c(‘s’, ‘s’), ncp: 5). The two first components of this MFA accounted for approximately 84% of the variability of the two combined datasets. A hierarchical clustering was performed on these five first components using the HCPC function of the FactoMineR package (parameters were set as follows, nbclust: 3, metric:“euclidean”, method: “ward”). This identified three clusters of TFs characterized by an increasing number of cell‐types displaying high expression from CTS to UBQ TFs (Fig EV2A and B).

#### Mining of transcriptomic data to monitor hepatic dedifferentiation

To monitor hepatocyte dedifferentiation in mouse liver injury models, all liver injury transcriptomic data were batch corrected against the liver development transcriptomic data used as a reference. This was performed with the ComBat function of the R package SVA (parameters were set as follows, mean.only: T, par.prior: T, control samples for the batch correction were the adult livers for the differentiation study and the livers from wild‐type or untreated mice for the injury studies; Leek *et al*, 2022). Then a principal component analysis (PCA) was computed on the liver differentiation study using the R package FactoMineR (scale.unit set to F; Lê *et al*, 2008). Since the first component, representing 64% of the dataset variability, allowed to separate the different stages of liver differentiation, it was used to project the liver injury studies. Fold changes between injured and control livers were computed on the batch corrected data and the median log_2_ fold change for individual genes across the different studies was recovered and plotted.

#### Mining transcriptional changes in identity genes versus control gene sets

Transcriptional modulations of identity effector and TF‐encoding genes were compared to those in control genes matched for mouse liver promoter activity. A hierarchical clustering was performed based on promoter DHS‐seq and H3K27ac ChIP‐seq signals together with mouse liver gene expression levels considering all active non‐TF genes (Appendix Fig S9A) or only TF‐encoding genes (Appendix Fig S10A). Individual signals were scaled and clustering was performed using log_2_‐transformed data. This allowed to define four clusters of promoters with different activity levels among both non‐TF and TF‐encoding genes. Groups of control genes were defined by randomly selecting an equivalent number of non‐identity genes matching the distribution of identity effector or TF genes in these clusters. This was performed 1,000 times (without replacement) to compare the identity genes to the individual control gene sets (Appendix Figs S9A and S10A). Results of Wilcoxon rank sum tests were recorded and the mode of the *P*‐value distribution was used to select a representative control gene set among the 1,000 subsamples. Selected representative control groups were in main figures.

#### Gene expression correlation in human HCC and overall survival analyses

The web portal cSurvival v1.0.1 was used (https://tau.cmmt.ubc.ca/cSurvival/; Cheng *et al*, 2022) to retrieved the spearman correlation between the average normalized mRNA expression of Hep‐ID^CONNECT^ TF and Hep‐ID TF genes taken as gene sets in “Liver Hepatocellular Carcinoma (LIHC from TCGA)” (*n* = 367 curated samples). Complementary analyses were similarly performed using individual Hep‐ID^CONNECT^ TF‐encoding genes. Overall survival data were retrieved in parallel from cSurvival, which uses automated stratification between low and high expressing groups from an optimal cut‐off defined from the minimum *P*‐value (Cheng *et al*, 2022). To mine associations of low versus high expressing HCC with additional clinical parameters, patients ID were retrieved from the cSurvival analyses and used in the cBioPortal (https://www.cbioportal.org/; Gao *et al*, 2013).

#### Mining of single‐nuclei RNA‐seq data

Gene expression counts obtained for healthy adult mouse and human livers were downloaded from https://www.livercellatlas.org/ (Guilliams *et al*, 2022). Single‐nuclei RNA‐seq data were selected by keeping only data labeled as “nucSeq” for their sample type. Counts were normalized using SCTransform from the Seurat v4.0.3 package (Hao *et al*, 2021). Cell types comprising less than 50 cells were removed. Data were finally loaded into ISCEBERG v1.0.0 (preprint: Guille *et al*, 2022) to generate violin plots of Hep‐ID and Hep‐ID^CONNECT^ TF‐encoding gene average expression.

### DHS‐seq and histone ChIP‐seq data analyses

Uniform reprocessing of the data including peak calling and genome‐wide average signal has been described in Dubois‐Chevalier *et al* (2017a). ChIP‐seq signals were also alternatively used as enrichments over input, which were obtained as follows. First, input datasets from several mouse liver ChIP‐seq studies (Dataset EV2) were merged into a “meta‐input” file after removal of duplicated reads and false‐positives regions identified by ENCODE (blacklisted regions v1; Amemiya *et al*, 2019) or defined as repeatedly enriched in inputs and IgG ChIP‐seq in our previous study (Dubois‐Chevalier *et al*, 2017a). Then, the Bam file for each TF ChIP‐seq dataset was used to run MACS2 callpeak (−g = 1.89e9, −q = 0.05, −‐keep‐dup = all, −‐scale‐to = small, B) using the “meta‐input” Bam file as control. Finally, the two BedGraph files issued from MACS2 callpeak were used in MACS2 bdgcmp (m = logFE) to obtain the genome‐wide log_2_ fold enrichment track files over input for each TF (MACS2 version 2.2.7.1; Zhang *et al*, 2008). DHS‐seq or ChIP‐seq signal at a given promoter was defined as the maximum signal within TF binding sites (i.e., *cis*‐regulatory modules as defined hereabove) encompassing this promoter retrieved using the extract bed function of bwtool version 1.0 (Pohl & Beato, 2014). Comparison of individual TF binding to identity and control gene promoters involved reiterative comparisons with independent matched control groups as described hereabove for transcriptomics analyses. Combinatorial binding of Hep‐ID TFs, Hep‐ID^CONNECT^ and CTCF on effector gene promoters was compared using the distance correlation provided by the dcor function of the Rfast package in R (Papadakis *et al*, 2022).

Visualization of ChIP‐seq data at selected genes was performed using the Integrated Genome Browser (IGB 9.1.4; Freese *et al*, 2016).

H3K4me3 ENCODE ChIP‐seq data from mouse tissues (Shen *et al*, 2012) were used to call H3K4me3‐enriched regions using the broad peak calling option of MACS2 as described in Chen *et al* (2015). Genes were assigned to H3K4me3‐enriched regions using the closestBed function (with parameters −t all, −k 1, −d, −mdb) of the BEDTools version 2.3.0 (Quinlan & Hall, 2010), that is, active gene TSS from the mouse liver PRO‐Seq data (Wang *et al*, 2018) were assigned H3K4me3‐enriched regions when distance was 0. Calling of super‐enhancers (SE) and target gene assignation, essentially performed as in (Loven *et al*, 2013; Whyte *et al*, 2013), was previously described (Dubois‐Chevalier *et al*, 2020).

### Mouse phenotype ontology and literature mining

Enrichments within the mouse phenotype ontology or biological pathway data were defined using ToppCluster (Chen *et al*, 2007; Kaimal *et al*, 2010). Default parameters were used and only terms linked to > 5 TF‐encoding genes were considered.

References to Hep‐ID^CONNECT^ TFs in the scientific literature related to liver/hepatocyte metabolic functions were retrieved using the easyPubMed package (https://www.data‐pulse.com/dev_site/easypubmed/) in R. articles with co‐occurrence of a given Hep‐ID^CONNECT^ TF gene name and (“hepatocyte” or “liver”) and “metabolism” in their title or abstract were considered. The number of retrieved manuscripts for each Hep‐ID^CONNECT^ TF was visualized as a heatmap prepared using the heatmap.2 function of the R package gplots.

### Cell culture and treatments

The human cell line HepG2 (ATCC, HB‐8065) was cultured in minimum essential medium (MEM; Gibco, 11095080) supplemented with 10% fetal bovine serum (FBS; Dutscher, 500105H1), MEM non‐essential amino acids (Gibco, 11140035), 1 mM sodium pyruvate (Gibco, 11360070) and 100 U/ml penicillin–streptomycin (Gibco, 15140). Immortalized human hepatocytes (IHH; Schippers *et al*, 1997) were cultured in William's E medium (Gibco, 22551022) supplemented with 10% FBS (Dutscher, SV30160.03), 20 mU/ml bovin insulin (Sigma, I5500), 50 nM dexamethasone (Sigma, D1756) and 100 U/ml penicillin–streptomycin (Gibco, 15140). MPH were prepared from 8 weeks old male C57BL/6J mice (Charles River) and grown as described in (Dubois *et al*, 2020a). 10 nM T3 (T6397, Sigma‐Aldrich) was added to the media used for MPH isolation and *in vitro* cell culture. Cell culture was performed in a humidified atmosphere of 5% CO_2_ in a 37°C incubator. Cell‐lines were routinely monitored for mycoplasma contamination. For cell‐line treatments, cells were grown in media containing 10% dextran‐coated charcoal stripped serum for 48 h and then exposed to 10 nM T3 (T6397, Sigma‐Aldrich), 10 nM GC‐1 (SML1900, Sigma‐Aldrich) or 1 μM GW3965 (405911‐17‐3, Tocris). Vehicle (0.01% DMSO) was used as control for GC‐1 and GW3965 treatments. For serum stripping, activated charcoal solution (C9157, Sigma‐Aldrich) was washed three times and prepared in ultra‐pure water at final concentration 5%. Dextran T70 (2.5 g; 31390, Sigma‐Aldrich) was added to the pellet and charcoal‐coated dextran was mixed with 500 ml of serum and incubated overnight at 4°C. After centrifugation (20 min, 4,000 *g*, room temperature), the serum was filtered using a 0.2‐μm filter and heat inactivated at 56°C for 45 min. In experiments evaluating the effects of drugs on cell growth, cells were trypsinized, resuspended in culture media, stained with trypan blue (P08‐34100, PAN‐Biotech) and counted using a TC20 Automated Cell Counter (BIO‐RAD).

### Mouse experiments

Young adult male C57BL/6J wild‐type (WT) mice (7–11 weeks old) were purchased from Charles River and housed in standard cages in a temperature‐controlled room (22–24°C) with a 12‐h dark–light cycle. They had *ad libitum* access to tap water and standard chow and were allowed to acclimate for 2 weeks prior to initiation of the experimental protocol. Acute liver inflammation was induced by intraperitoneal injection of recombinant mouse IL1B using 0.5 μg/ mouse (575102, BioLegend) or vehicle (PBS) for 3 h followed by T3 treatment 0.2 mpk (T6397, Sigma‐Aldrich). Livers were collected 3 h later. Mice with hepatocyte‐specific deletion of *Thrb* have been described in (Billon *et al*, 2014).

All animal studies were performed in compliance with EU specifications regarding the use of laboratory animals and have been approved by the Nord‐Pas de Calais Ethical Committee (APAFIS#30322‐202102221656794 v4).

### RNA expression analyses

Tissues were homogenized using Minilys and 1.4 mm ceramic beads (Bertin Technologies). Total RNA was extracted from cell lines and tissues using the Nucleospin® RNA kit (Macherey‐Nagel) according to the manufacturer's protocol.

RNA was reverse‐transcribed using the High‐Capacity cDNA Reverse Transcription Kit (Applied Biosystem). Quantitative PCR (qPCR) was performed on a Fast Applied (Applied Biosystem, Life Technologies, Cergy Pontoise, France) using the Takyon kit (Agilent Technologies). The specificity of the amplification was checked by recording the dissociation curves, and the efficiency was verified to be above 95% for each primer pair. mRNA levels were normalized to the expression of housekeeping genes and the fold induction was calculated using the cycle threshold (ΔΔCT) method. The sequences of primers used are listed in Table EV4.

For transcriptomic analyses, RNA integrity and quantity were evaluated using the Agilent 2100 Bioanalyser (Agilent Technologies). RNA was then processed for transcriptomic analysis using Affymetrix GeneChip arrays (MoGene 2.0) or high‐throughput sequencing (RNA‐seq) as previously described (Bobowski‐Gerard *et al*, 2022).

### Protein extraction

Proteins from the chromatin fraction were prepared as in (Dubois *et al*, 2020a). Cells were scraped in ice‐cold Phosphate‐Buffered Saline (PBS), pelleted by centrifugation at 400 *g* for 5 min, lysed in Buffer A (50 mM HEPES pH 7.5, 10 mM KCl, 1.5 mM MgCl_2_, 340 mM sucrose, 10% glycerol, 1 mM DTT, and protease inhibitor cocktail from Roche) and incubated for 10 min at 4°C. Samples were centrifuged at 1,300 *g* for 5 min at 4°C and supernatants were discarded. Nuclear pellets were washed with Buffer A and subsequently lysed in solution B (3 mM EDTA, 0.2 mM EGTA, 1 mM DTT, and protease inhibitor cocktail). After incubation for 30 min at 4°C, samples were centrifuged at 1,700 *g* for 5 min at 4°C and supernatants were discarded. Chromatin pellets were washed with solution B, resuspended in Buffer C (50 mM Tris–HCl pH 8.0, 1 mM MgCl2, and 83 U/μl benzonase) and incubated for 20 min at 4°C. Laemmli buffer 6× was added before loading for Western immunoblotting.

### Western blot assays

Protein extraction and western blotting were performed as described in Dubois *et al* (2020a). Protein concentrations were determined using the PierceTM BCA protein assay kit (Thermo scientific). Then, 20 μg of proteins were separated by 10% SDS–PAGE and immunodetected by Western immunoblotting using the primary antibodies listed in the Table EV5. Primary antibodies were detected using HRP‐conjugated secondary antibodies (Sigma‐Aldrich). Images were acquired using the iBright™ CL1500 Imaging System (Thermo Fisher Scientific).

### Statistical analyses

Statistical analyses were performed using the Prism software (GraphPad, San Diego, CA) and R (R Core Team, 2015). The specific tests and corrections for multiple testing which were used as well as the number of mice or independent biological replicates are indicated in the figure legends. Two‐sided tests were used unless specified in the figure legends. All bar graphs show means ± SD (standard deviations). Box plots are composed of a box from the 25^th^ to the 75^th^ percentile with the median as a line. Unless specified in the figure legend, whiskers extent to the most extreme data point which is no more than 1.5 times the interquartile range from the box.

## Author contributions

**Julie Dubois‐Chevalier:** Conceptualization; data curation; formal analysis; validation; investigation; visualization; writing – review and editing. **Céline Gheeraert:** Conceptualization; data curation; formal analysis; validation; investigation; visualization; writing – review and editing. **Alexandre Berthier:** Investigation. **Clémence Boulet:** Investigation. **Vanessa Dubois:** Investigation. **Loïc Guille:** Investigation. **Marie Fourcot:** Investigation. **Guillemette Marot:** Investigation. **Karine Gauthier:** Conceptualization; resources. **Laurent Dubuquoy:** Conceptualization; resources. **Bart Staels:** Conceptualization; funding acquisition; project administration. **Philippe Lefebvre:** Conceptualization; funding acquisition; project administration. **Jérôme Eeckhoute:** Conceptualization; data curation; formal analysis; funding acquisition; visualization; writing – original draft; project administration.

## Disclosure and competing interests statement

The authors declare that they have no conflict of interest.

## Supporting information



Appendix

Expanded View Figures PDF

Table EV1

Table EV2

Table EV3

Table EV4

Table EV5

Dataset EV1

Dataset EV2

PDF+

Source Data for Figure 2

Source Data for Figure 3

Source Data for Figure 4

Source Data for Figure 5

## Data Availability

Transcriptomics data generated in this study have been deposited into the Gene Expression Omnibus (GEO) database and are available under accession numbers GSE216278 (http://www.ncbi.nlm.nih.gov/geo/query/acc.cgi?acc=GSE216278) and GSE218724 (http://www.ncbi.nlm.nih.gov/geo/query/acc.cgi?acc=GSE218724).
