# Supplementary material for: An extended transcription factor regulatory network controls hepatocyte identity
Source: EMBO Rep. 2023 Jul 10;24(9):e57020. doi: 10.15252/embr.202357020 (PMC10481658; doi:10.15252/embr.202357020)
Supplement: Supplementary file 1 — Appendix [file EMBR-24-e57020-s011.pdf]

Appendix table of contents

Appendix Figure S1. Characteristics of the mouse liver TF-encoding gene promoters and of the SOM issued from their analysis (p2-3).

Appendix Figure S2. Characterization of the main clusters of TF-encoding gene promoters (p3-4).

Appendix Figure S3. Transcriptional regulator co-recruitment at promoters from clusters A-G (p4-13).

Appendix Figure S4. Additional characterization of the main clusters of TF-encoding gene promoters (p14-15).

Appendix Figure S5. Average expression of Hep-ID and Hep-ID^{CONNECT} TF encoding genes in individual mouse and human liver cell types (p16-17).

Appendix Figure S6. Loss of hepatocyte molecular identity in mouse models of severe liver injury (p18-19).

Appendix Figure S7. Length of H3K4me3 domains at the promoter of TF-encoding genes in various mouse tissues/organs (p20-21).

Appendix Figure S8. Association between Hep-ID^{CONNECT} TFs and liver/hepatocyte metabolism in the scientific literature (p22-23).

Appendix Figure S9. Strategy used to compare TF binding to the promoters of identity effector genes and activity-matched control non-TF gene promoters (p24-25).

Appendix Figure S10. Strategy used to compare TF binding to or transcriptional regulation of Hep-ID TFs and activity-matched control TF genes (p26-27).

Appendix Figure S11. Biological pathways linked to Hep-ID, Hep-ID^{CONNECT} and remaining TF-encoding genes from cluster G (Others) (p28-29).

Appendix Figure S12. Clinical features associated with HCC displaying low or high expression levels of Hep-ID^{CONNECT} TF genes (p30-31).

Appendix Figure S13. T3-mediated transcriptional regulation of Hep-ID TF genes in livers of healthy mice (p32-33).

Appendix Figure S14. T3-mediated transcriptional regulation of Hep-ID TF genes in MPH (p34-35).

Fig.S1

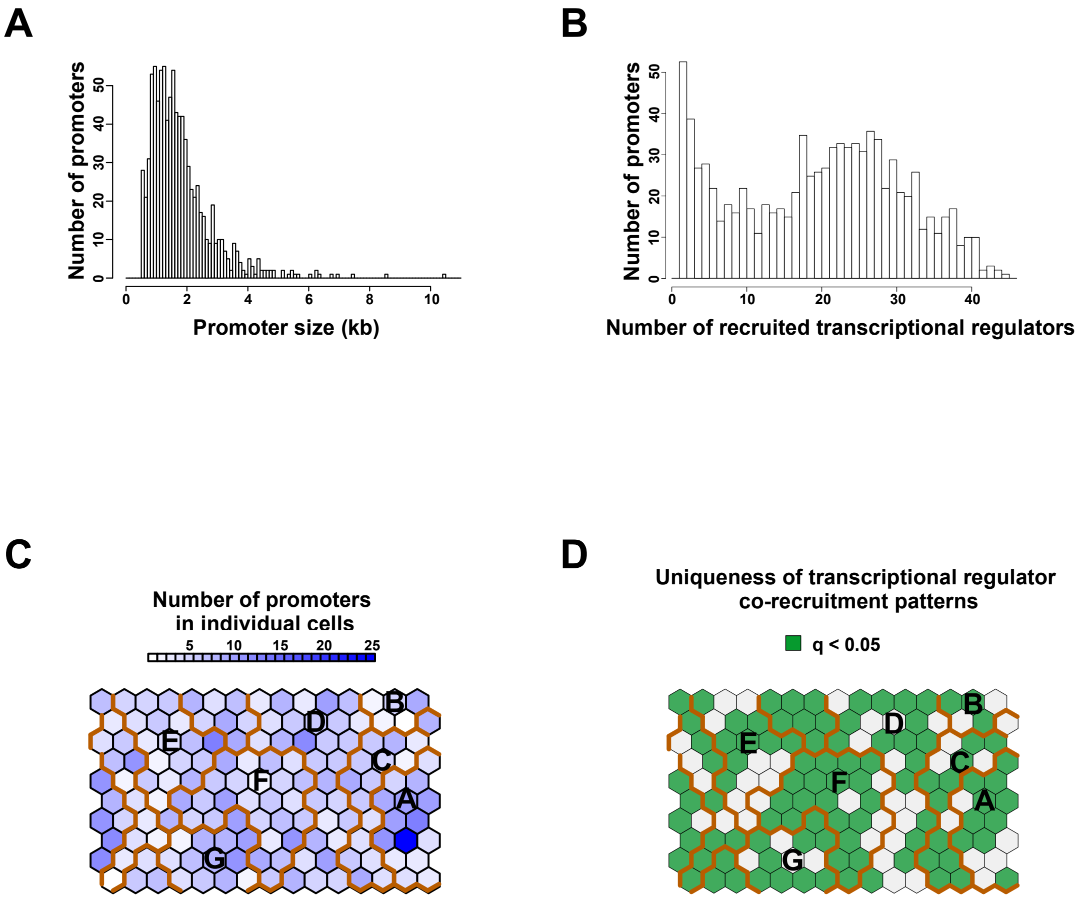
A
Number of promoters
0
10
20
30
40
50
0
2
4
6
8
10
Promoter size (kb)
B
Number of promoters
0
10
20
30
40
50
0
10
20
30
40
Number of recruited transcriptional regulators
C
Number of promoters
in individual cells
5
10
15
20
25
A
B
C
D
E
F
G
D
Uniqueness of transcriptional regulator
co-recruitment patterns
q < 0.05
A
B
C
D
E
F
G

Appendix Figure S1. Characteristics of the mouse liver TF-encoding gene promoters and of the SOM issued from their analysis.

A) Distribution of the promoters used in the SOM analysis as a function of their size. **B)** Distribution of the promoters used in the SOM analysis as a function of the number of co-bound transcriptional regulators. **C)** The SOM was used to indicate the number of independent promoters comprised within each individual cell. **D)** Cells in green are those with a unique transcriptional regulator co-recruitment pattern. This was defined as cells for which the representative transcriptional regulator co-recruitment pattern was statistically different from that of all other cells ($q<0.05$).

Fig.S2

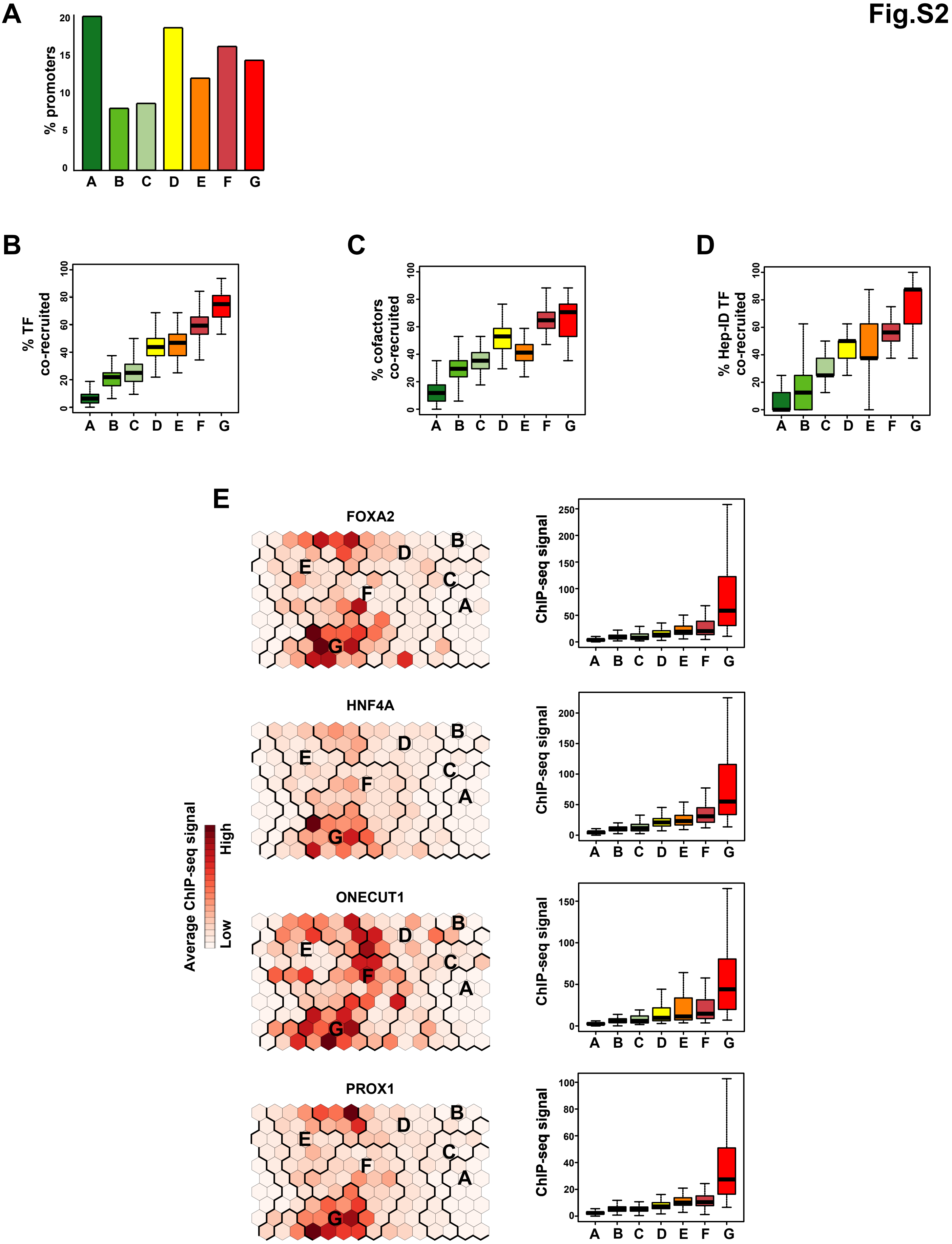
A
% promoters
B
% TF co-recruited
C
% cofactors co-recruited
D
% Hep-ID TF co-recruited
E
FOXA2
HNF4A
ONECUT1
PROX1
Average ChIP-seq signal
High
Low
ChIP-seq signal
A B C D E F G

Appendix Figure S2. Characterization of the main clusters of TF-encoding gene promoters.

A) Percentage of TF-encoding gene promoters analyzed retrieved in each one of clusters A-G. **B-D)** The percentage of analyzed TFs (B), cofactors (C) or Hep-ID TFs (D) co-recruited to individual promoters was monitored and box plots were used to display the data obtained for promoters belonging to clusters A-G. **E)** The map issued from Fig.1 was used to show the average ChIP-seq signal from mouse livers for the Hep-ID TFs FOXA2, HNF1A, HNF4A and ONECUT1 at promoters contained in individual cells. Bold black lines indicate the borders of clusters A-G. The data are also displayed as bar graphs on the right.

Fig.S3

A

Cluster A

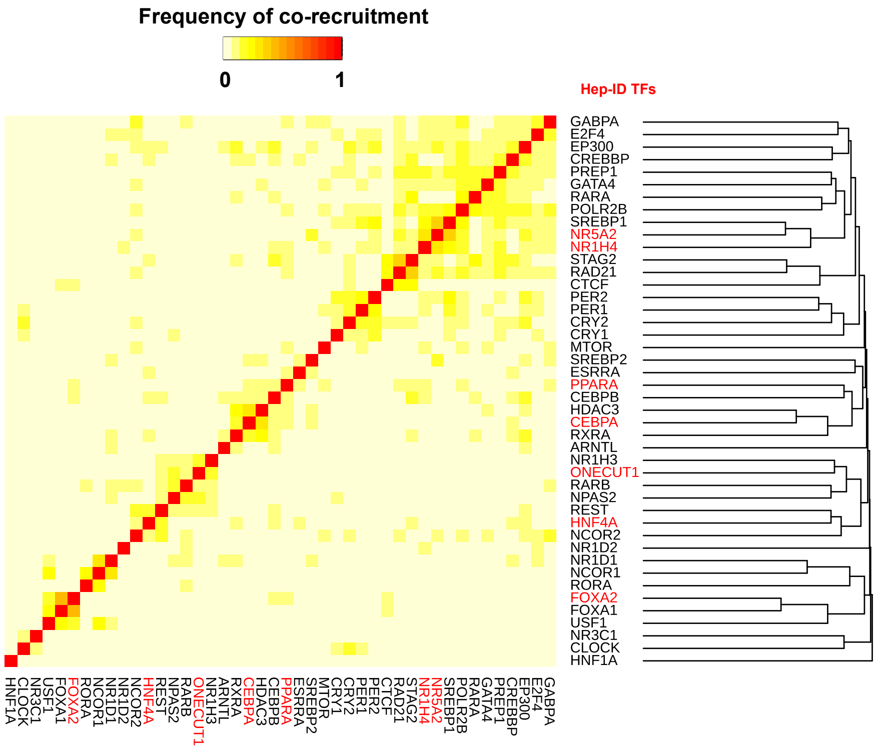

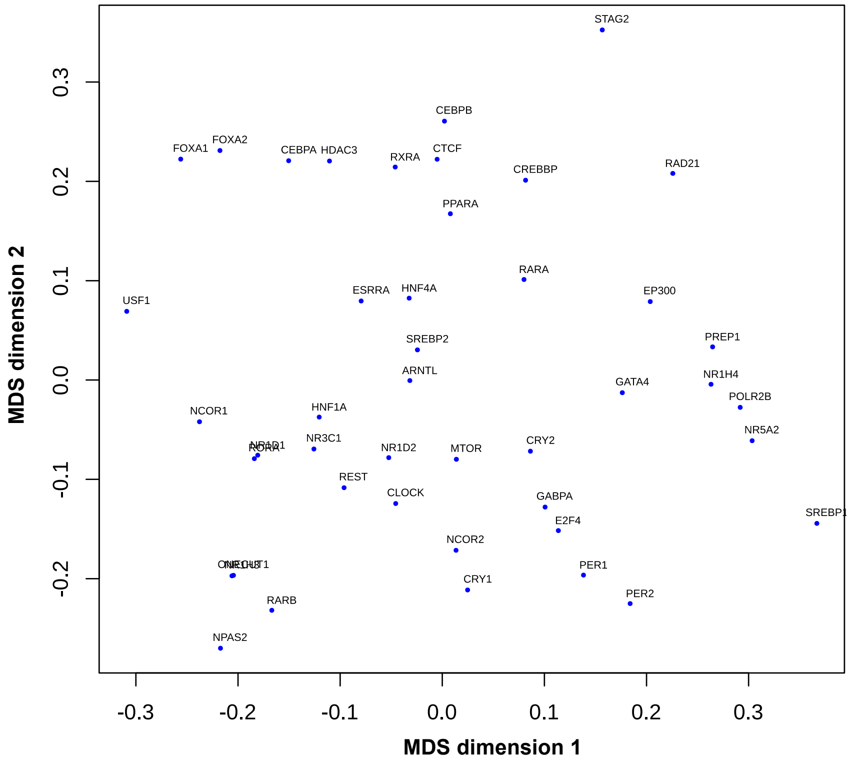

Fig.S3

B

Cluster B

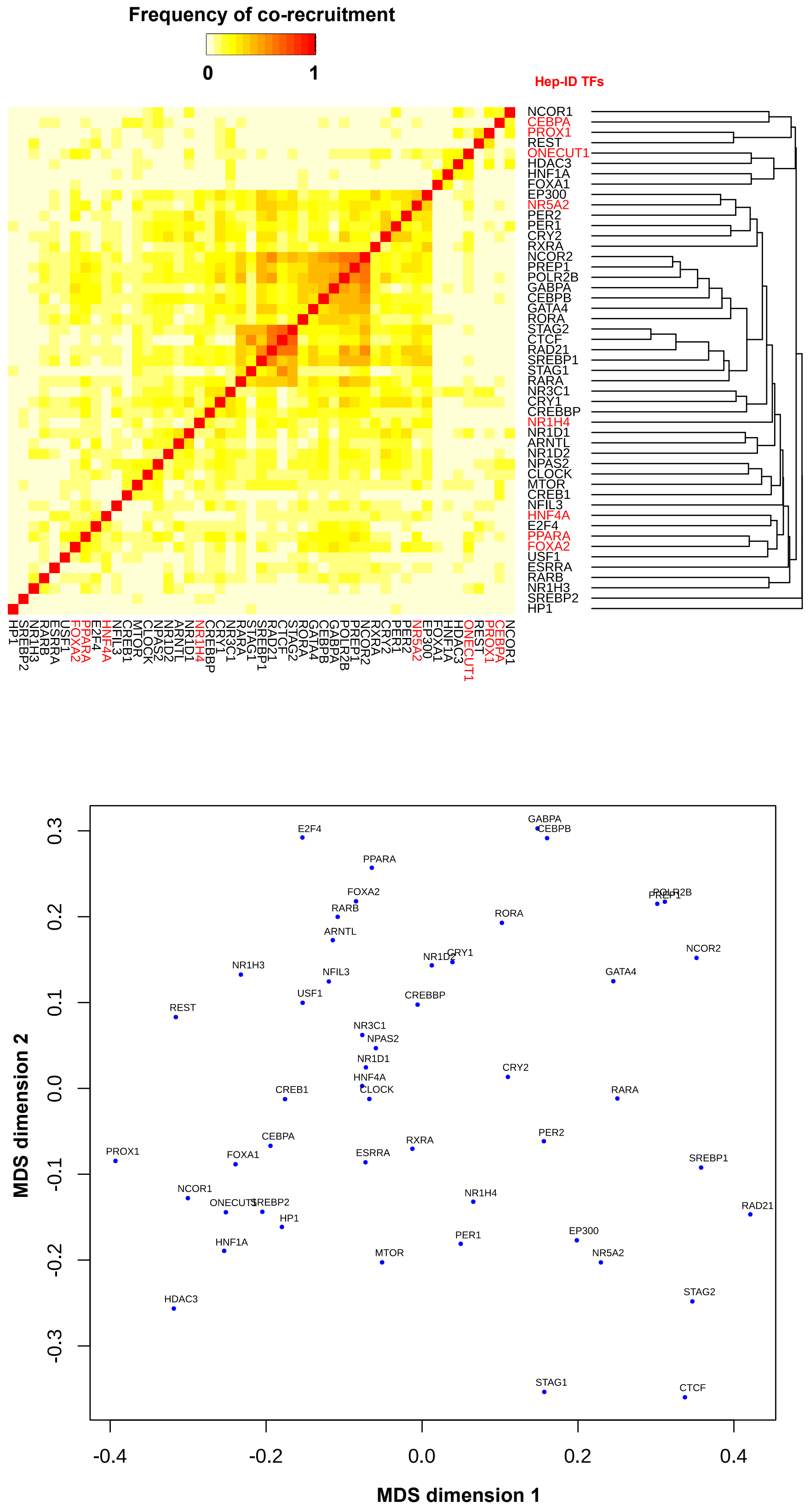

Fig.S3

C

Cluster C

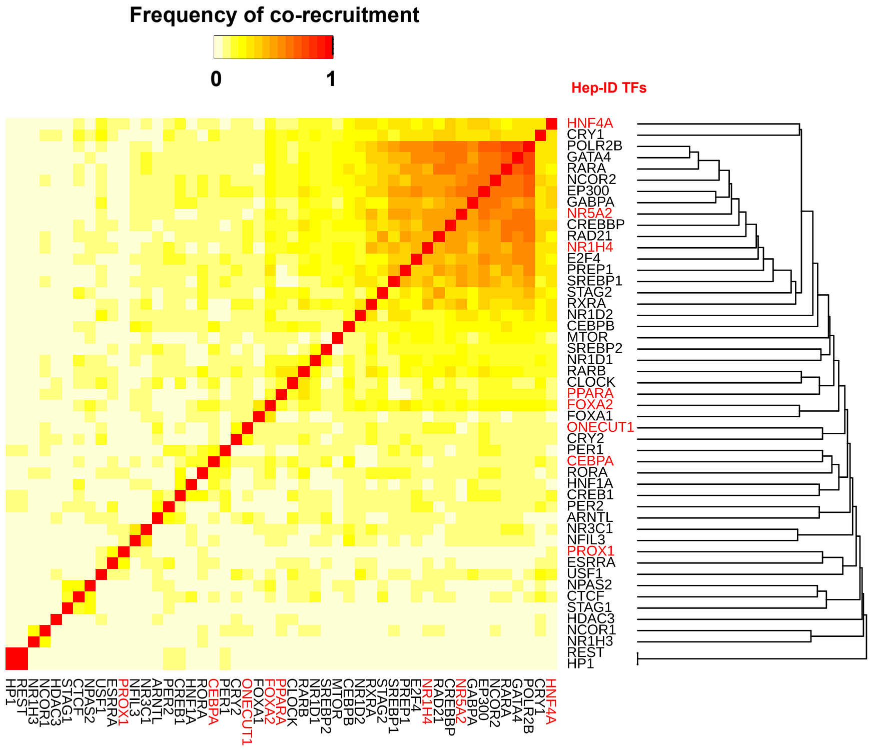

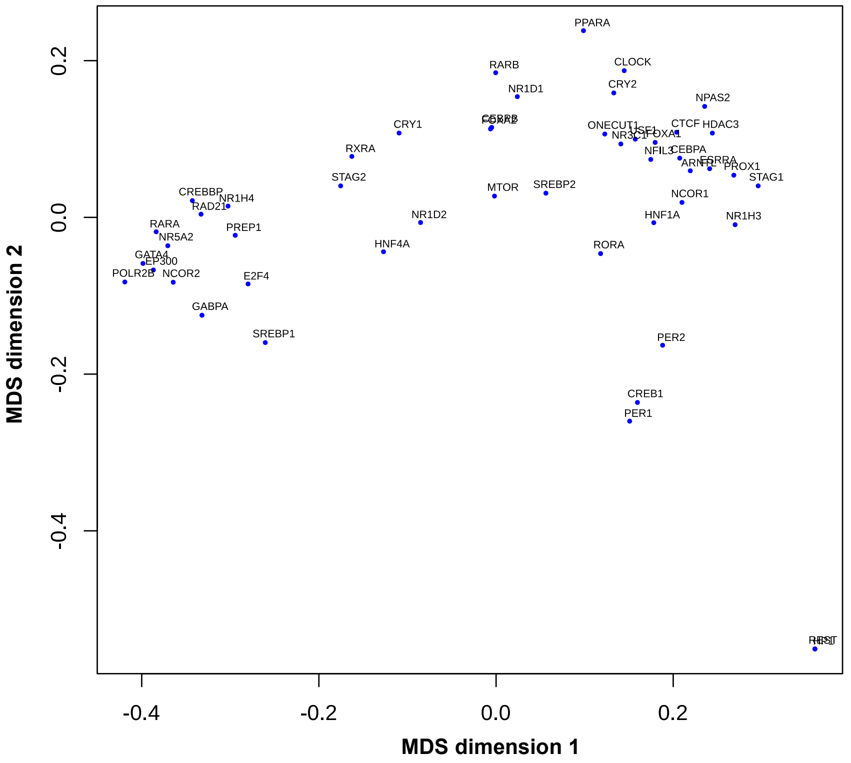

Fig.S3

D

Cluster D

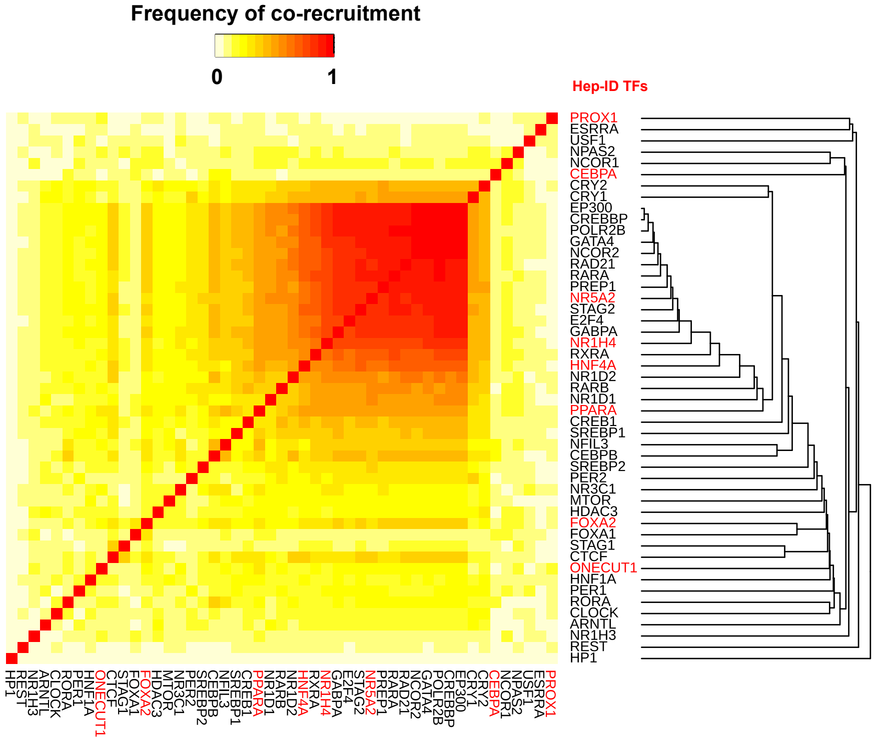

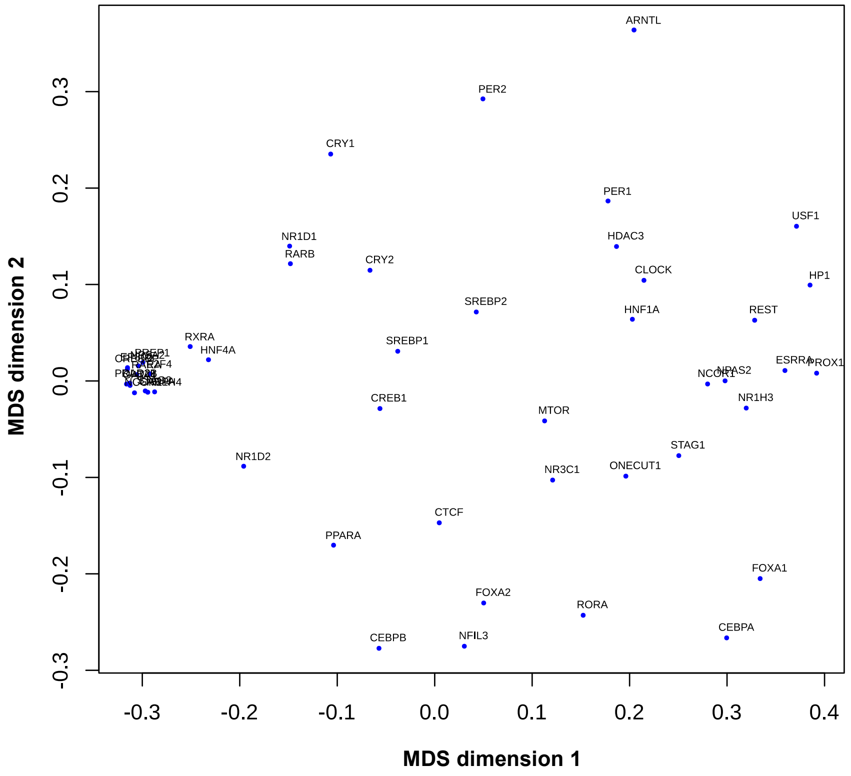

Fig.S3

E

Cluster E

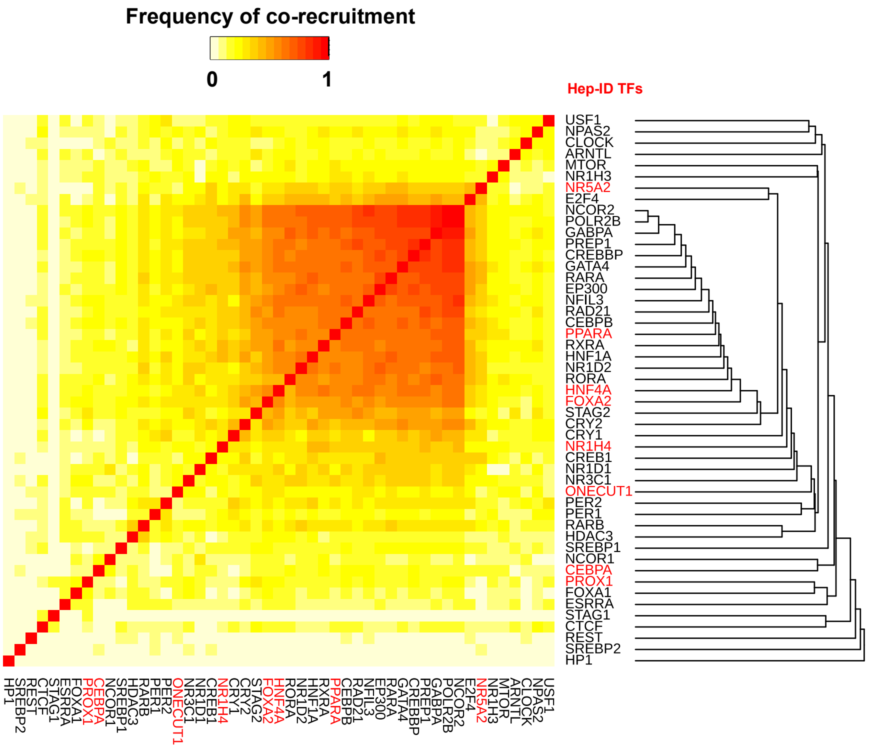

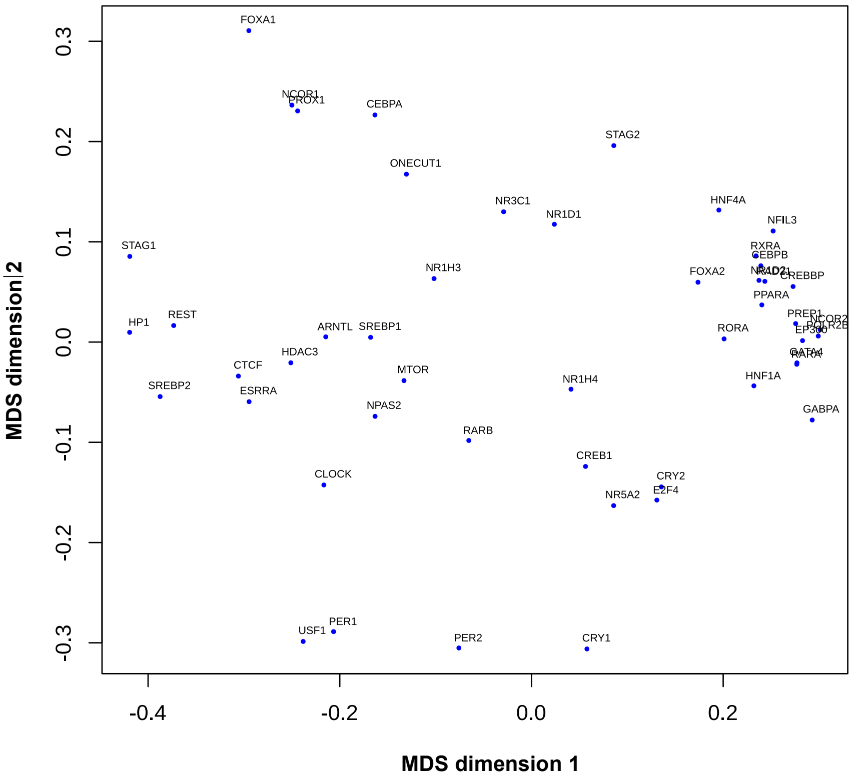

F

Cluster F

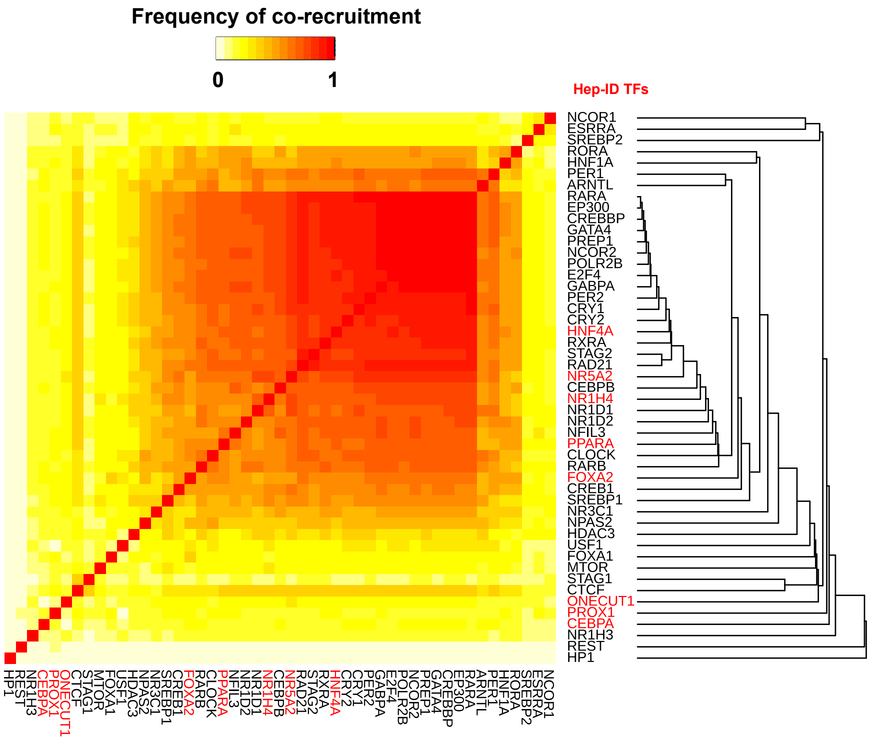
Frequency of co-recruitment
0
1
Hep-ID TFs
NCOR1
ESRRA
SREBP2
RORA
HNF1A
PER1
ARNTL
RARA
EP300
CREBBP
GATA4
PREP1
NCOR2
POLR2B
E2F4
GABPA
PER2
CRY1
CRY2
HNF4A
RXRA
STAG2
RAD21
NR5A2
CEBPB
NR1H4
NR1D1
NR1D2
NFIL3
PPARA
CLOCK
RARB
FOXA2
CREB1
SREBP1
NR3C1
NPAS2
HDAC3
USF1
FOXA1
MTOR
STAG1
CTCF
ONECUT1
PROX1
CEBPA
NR1H3
REST
HP1

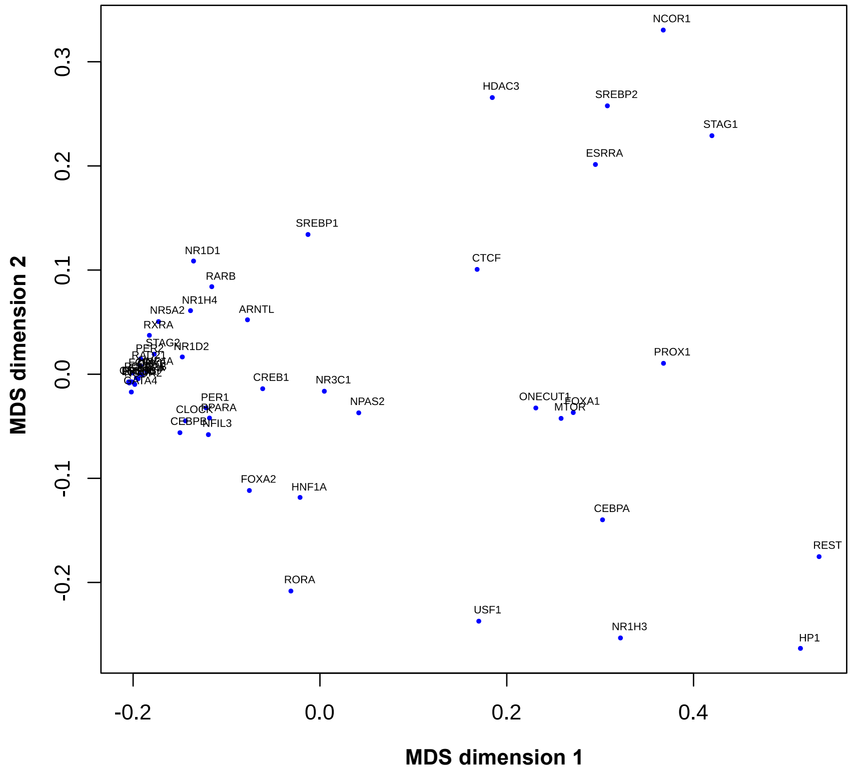
MDS dimension 2
MDS dimension 1
0.3
0.2
0.1
0.0
-0.1
-0.2
0.4
NCOR1
HDAC3
SREBP2
STAG1
ESRRA
SREBP1
NR1D1
CTCF
RARB
NR1H4
ARNTL
NR5A2
RXRA
NR1D2
PROX1
CREB1
NR3C1
PER1
PPARA
NPAS2
ONECUT1
FOXA1
MTOR
NFIL3
FOXA2
HNF1A
CEBPA
REST
RORA
USF1
NR1H3
HP1

Fig.S3

G

Cluster G

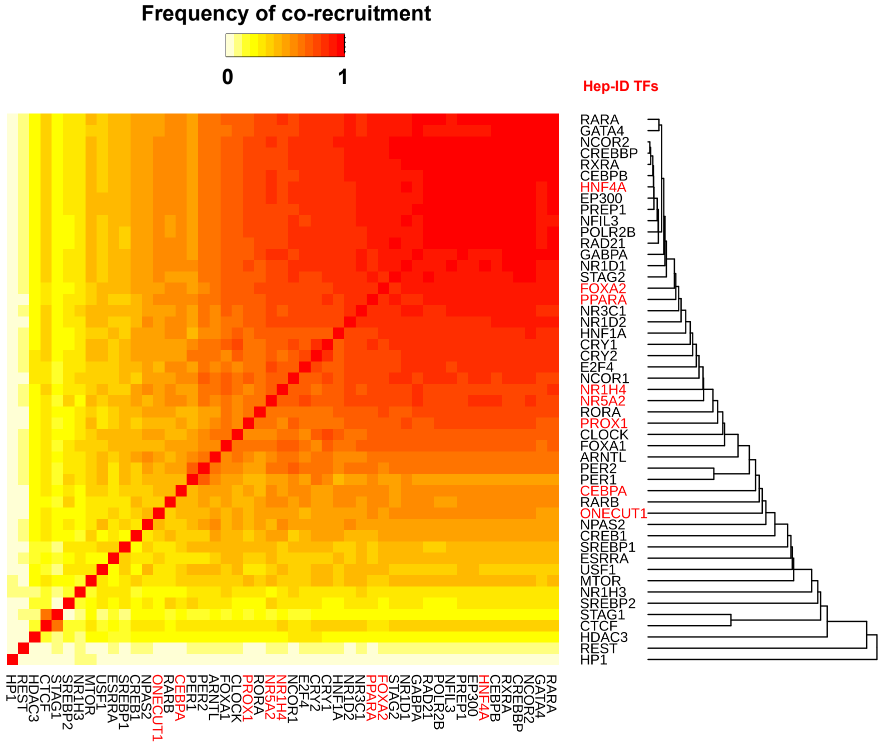

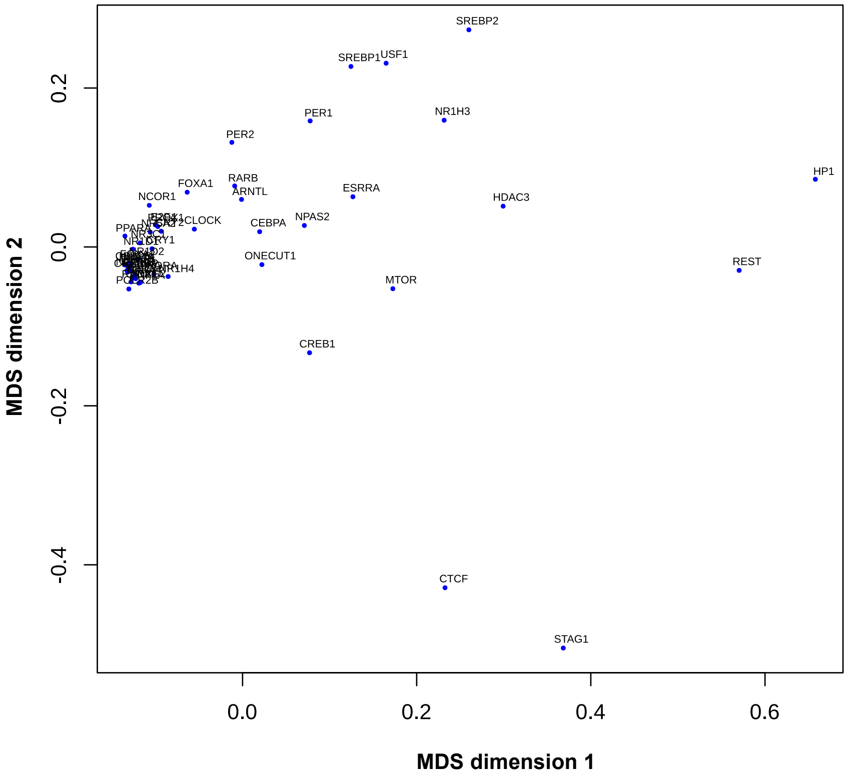

Appendix Figure S3. Transcriptional regulator co-recruitment at promoters from clusters A-G.

A-G) For each cluster of promoters, a heatmap showing transcriptional regulator co-recruitment defined using a Tanimoto index is shown (top). Transcriptional regulators were organized based on hierarchical clustering. In addition, these Tanimoto indexes were used for multidimensional scaling (MDS; see Materials and Methods) to further highlight transcriptional regulator co-recruitment patterns. Proximity in the 2D space used to display the MDS data point to co-recruitment in the set of analyzed promoters. A few TFs never bound to promoters from cluster A are omitted in panel A.

Fig.S4

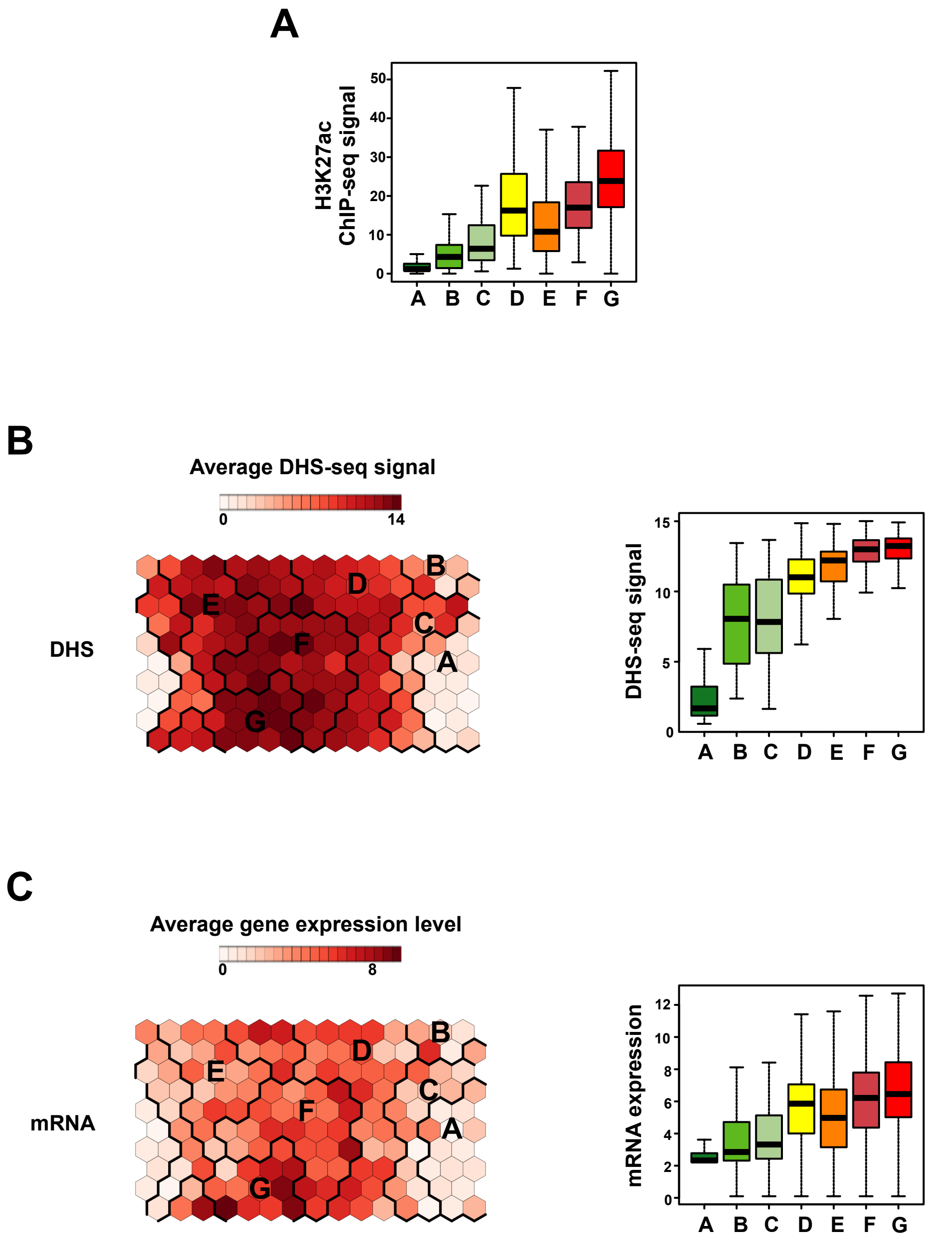
A
H3K27ac
ChIP-seq signal
0
10
20
30
40
50
A B C D E F G
B
Average DHS-seq signal
0
14
DHS
B
D
E
C
F
A
G
DHS-seq signal
0
5
10
15
A B C D E F G
C
Average gene expression level
0
8
mRNA
B
D
E
C
F
A
G
mRNA expression
0
2
4
6
8
10
12
A B C D E F G

Appendix Figure S4. Additional characterization of the main clusters of TF-encoding gene promoters.

A) Average ChIP-seq signal for mouse liver H3K27ac at promoters contained in clusters A-G.

B-C) The map issued from Fig.1 was used to show the average DHS-seq at promoters (B) and mRNA expression levels of the linked genes (C) from mouse livers in individual cells. Bold black lines indicate the borders of clusters A-G. The data are also displayed as bar graphs on the right.

Fig.S5

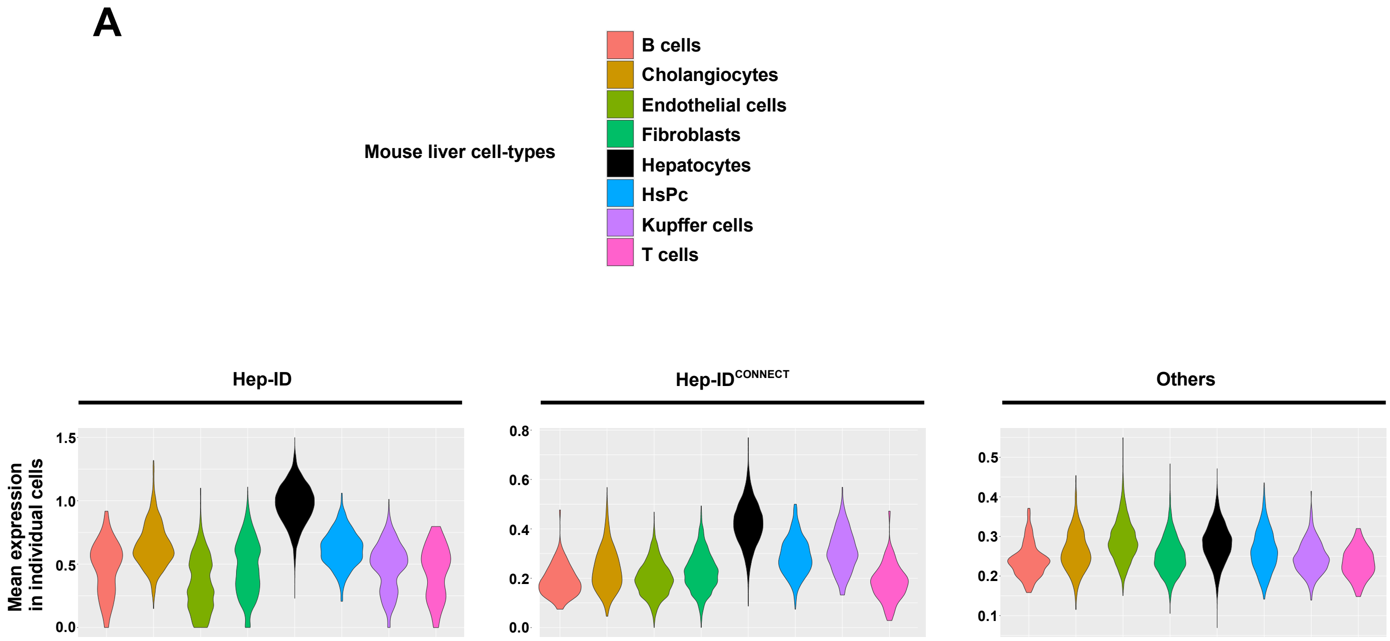
A
Mouse liver cell-types
B cells
Cholangiocytes
Endothelial cells
Fibroblasts
Hepatocytes
HsPc
Kupffer cells
T cells
Hep-ID
Hep-ID CONNECT
Others
Mean expression in individual cells
1.5
1.0
0.5
0.0
0.8
0.6
0.4
0.2
0.0
0.5
0.4
0.3
0.2
0.1

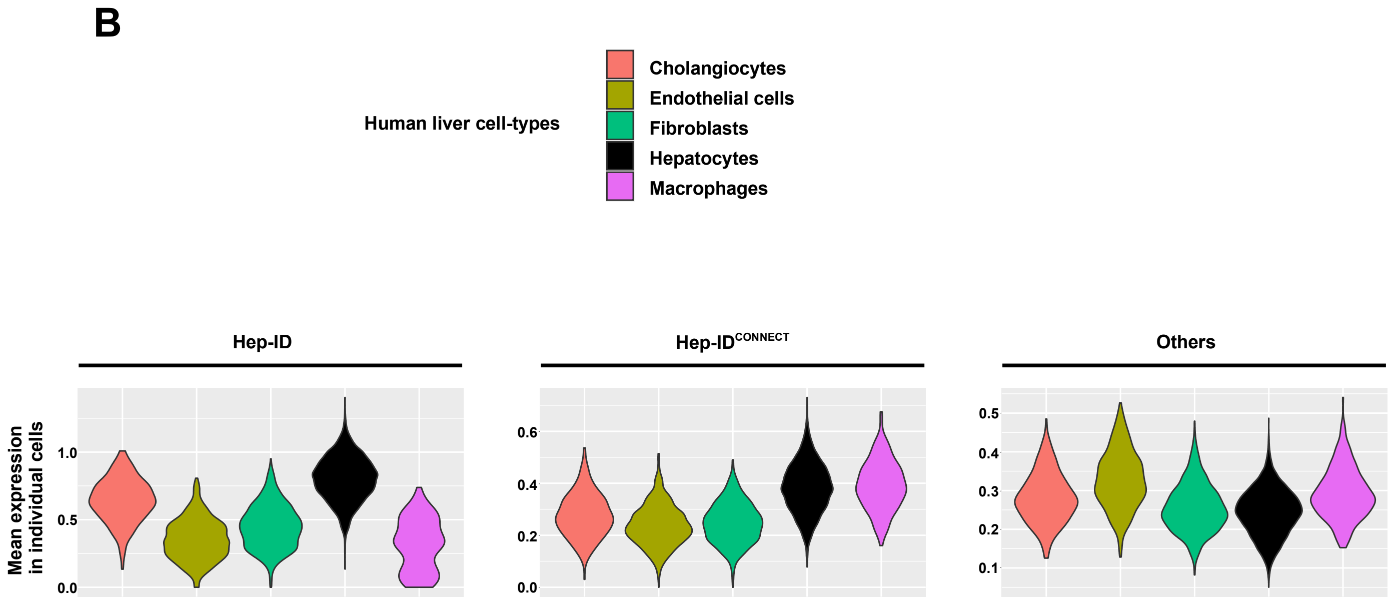
B
Human liver cell-types
Cholangiocytes
Endothelial cells
Fibroblasts
Hepatocytes
Macrophages
Hep-ID
Hep-ID CONNECT
Others
Mean expression in individual cells
1.0
0.5
0.0
0.6
0.4
0.2
0.0
0.5
0.4
0.3
0.2
0.1

Appendix Figure S5. Average expression of Hep-ID and Hep-ID^{CONNECT} TF encoding genes in individual mouse and human liver cell types.

Average expression of Hep-ID, Hep-ID^{CONNECT} and remaining TF-encoding genes from cluster G (Others) was monitored in single-nuclei RNA-seq data obtained from healthy adult mouse (**A**) or human (**B**) livers (Guilliams *et al*, 2022) and plotted as violin plots.

Fig.S6

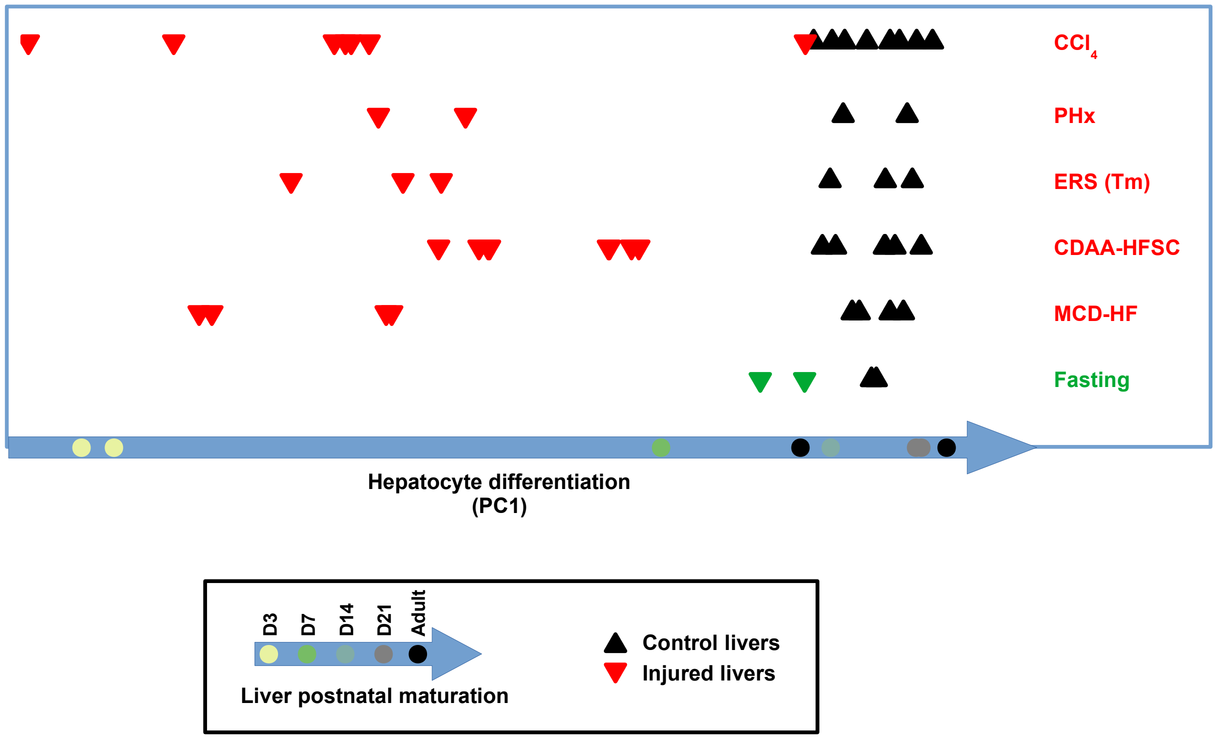
CCl4
PHx
ERS (Tm)
CDAA-HFSC
MCD-HF
Fasting
Hepatocyte differentiation
(PC1)
D3
D7
D14
D21
Adult
Liver postnatal maturation
Control livers
Injured livers

Appendix Figure S6. Loss of hepatocyte molecular identity in mouse models of severe liver injury.

Comparison of the transcriptome of the indicated liver injury models (Table EV2) with that of the developing mouse liver performed as described in our previous study [26] and in the Materials and Methods section. PC1 is the first principal component which represents 63.55% of the variability within the mouse liver differentiation study. PC1 was used to project the liver injury studies (transcriptomic data from individual mice are shown as triangles). Transcriptomic changes induced by fasting were used as a control. CCl_4, carbon tetrachloride hepatotoxicity - model of drug-induced chronic liver injury; PHx, partial liver hepatectomy - model of liver resection which is a frequent clinical practice to remove liver tumors; ERS (Tm), endoplasmic reticulum stress induced by tunicamycin injection - model of drug-induced acute liver injury. CDAA-HFSC, Choline-Deficient L-amino-acid-defined diet with high fat, sucrose and cholesterol diet - model of NASH/fibrosis; MCD-HF, methionine–choline-deficient diet with high fat - model of NASH/fibrosis.

Fig.S7

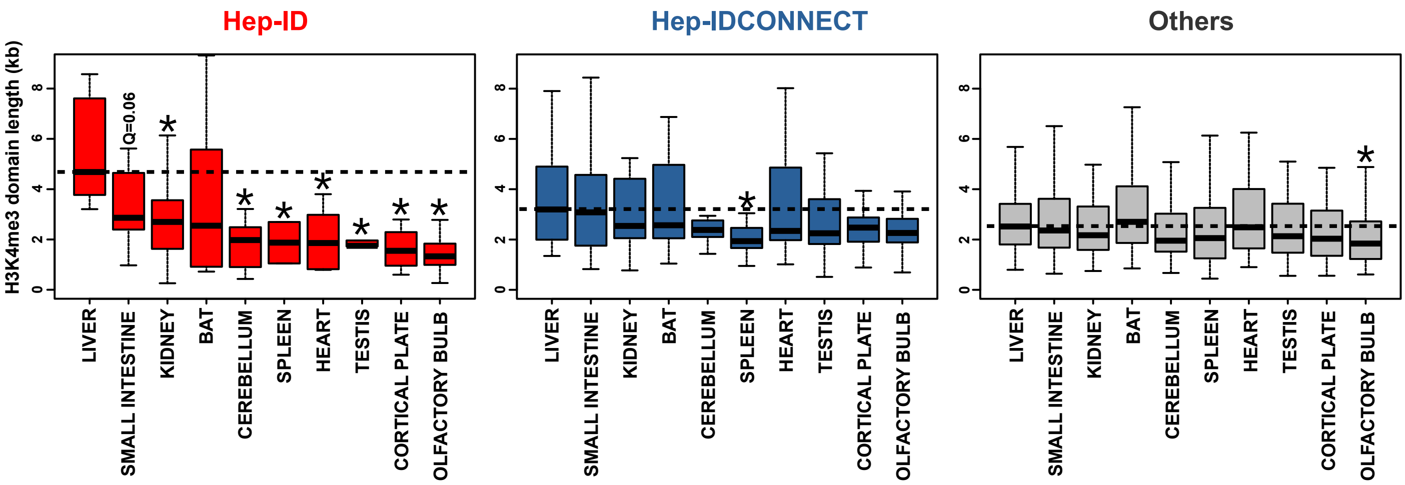
Hep-ID
Hep-IDCONNECT
Others
H3K4me3 domain length (kb)
Q=0.06
LIVER
SMALL INTESTINE
KIDNEY
BAT
CEREBELLUM
SPLEEN
HEART
TESTIS
CORTICAL PLATE
OLFACTORY BULB

Appendix Figure S7. Length of H3K4me3 domains at the promoter of TF-encoding genes in various mouse tissues/organs.

Uniformly processed H3K4me3 ChIP-seq data from various mouse tissues/organs were used to monitor the length of the H3K4me3 domains overlapping the TSS of Hep-ID, Hep-ID^{CONNECT} and remaining TF-encoding genes from cluster G (Others). Dotted lines show the median length in the liver. Statistical difference between liver and other organs was defined using Kruskal-Wallis with Wilcoxon pairwise comparison tests followed by Benjamini-Hochberg correction for multiple testing correction. *q<0.05.

Fig.S8

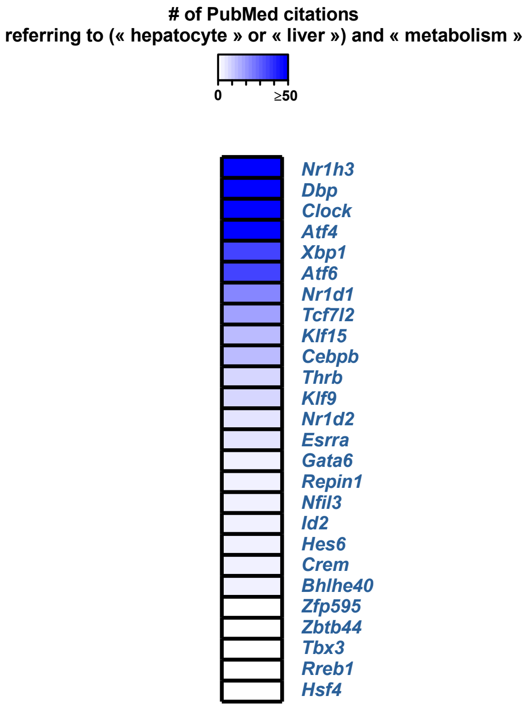

# of PubMed citations
referring to (« hepatocyte » or « liver ») and « metabolism »
0
≥50
Nr1h3
Dbp
Clock
Atf4
Xbp1
Atf6
Nr1d1
Tcf7l2
Klf15
Cebpb
Thrb
Klf9
Nr1d2
Esrra
Gata6
Repin1
Nfil3
Id2
Hes6
Crem
Bhlhe40
Zfp595
Zbtb44
Tbx3
Rreb1
Hsf4

Appendix Figure S8. Association between Hep-ID^{CONNECT} TFs and liver/hepatocyte metabolism in the scientific literature.

The heatmap displays the numbers of PubMed-referenced articles referring to individual Hep-ID^{CONNECT} TFs and ("liver" or "hepatocyte") and "metabolism".

Fig.S9

A

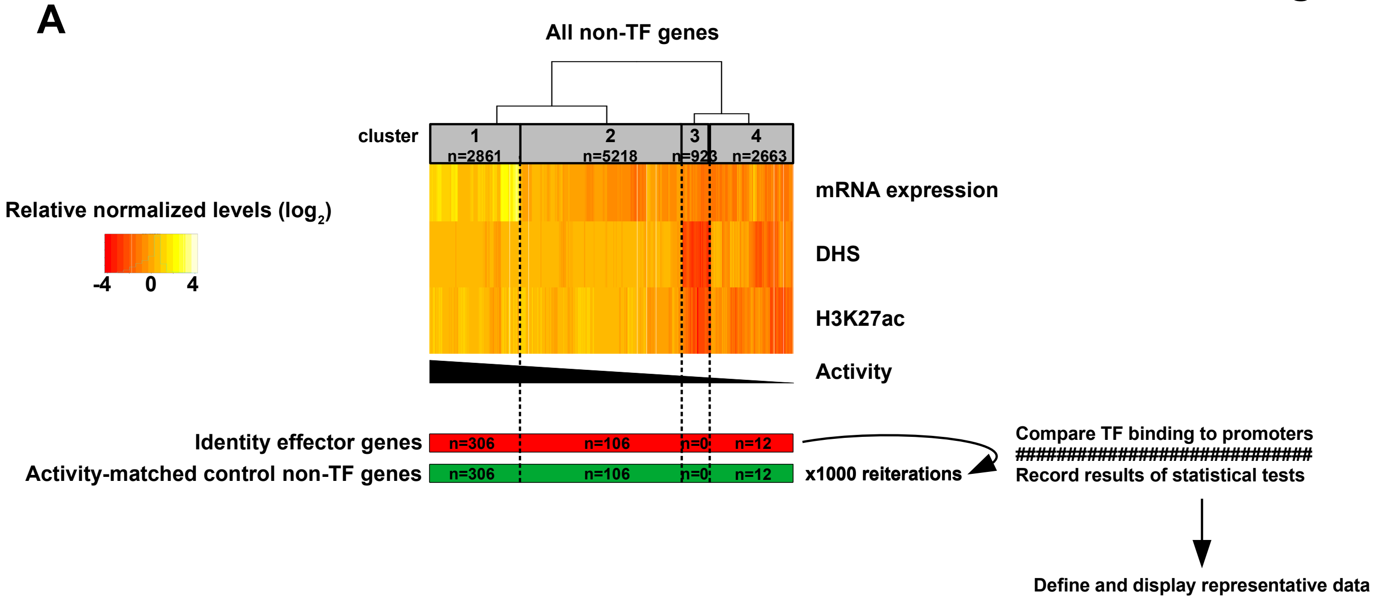

All non-TF genes
cluster
1 n=2861
2 n=5218
3 n=923
4 n=2663
Relative normalized levels (log2)
-4 0 4
mRNA expression
DHS
H3K27ac
Activity
Identity effector genes
n=306 n=106 n=0 n=12
Activity-matched control non-TF genes
n=306 n=106 n=0 n=12
x1000 reiterations
Compare TF binding to promoters
############################
Record results of statistical tests
Define and display representative data

B

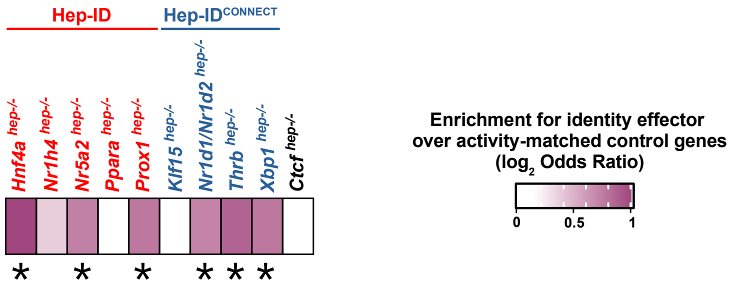

Hep-ID
Hep-ID CONNECT
Hnf4a hep-/-
Nr1h4 hep-/-
Nr5a2 hep-/-
Ppara hep-/-
Prox1 hep-/-
Klf15 hep-/-
Nr1d1/Nr1d2 hep-/-
Thrb hep-/-
Xbp1 hep-/-
Ctcf hep-/-
* * * * * *
Enrichment for identity effector
over activity-matched control genes
(log2 Odds Ratio)
0 0.5 1

Appendix Figure S9. Strategy used to compare TF binding to the promoters of identity effector genes and activity-matched control non-TF gene promoters.

A) Non-TF-encoding gene promoters were clustered based on their activity in the mouse liver defined through mRNA expression levels of associated genes and DHS-seq and H3K27ac ChIP-seq signals. The hierarchical clustering tree is shown on top of the heatmap together with identification of 4 main clusters. The activity-matched control set was obtained by randomly picking within each of the 4 clusters a number of genes equivalent to that of identity effector genes. TF binding to the promoters of identity effector and activity-matched control genes was defined by mining ChIP-seq signal intensity and statistical difference was tested using Wilcoxon Rank Sum Tests. This analysis was reiterated 1000 times and the mode of the p-value distribution was used to select a representative control gene set used in Fig.2I. **B)** Analyses similar to those in Fig.2H but using as control an equivalent number of activity-matched non-TF-encoding genes, which gives data representative of those obtained using 1000 reiterations of the analysis as described hereabove.

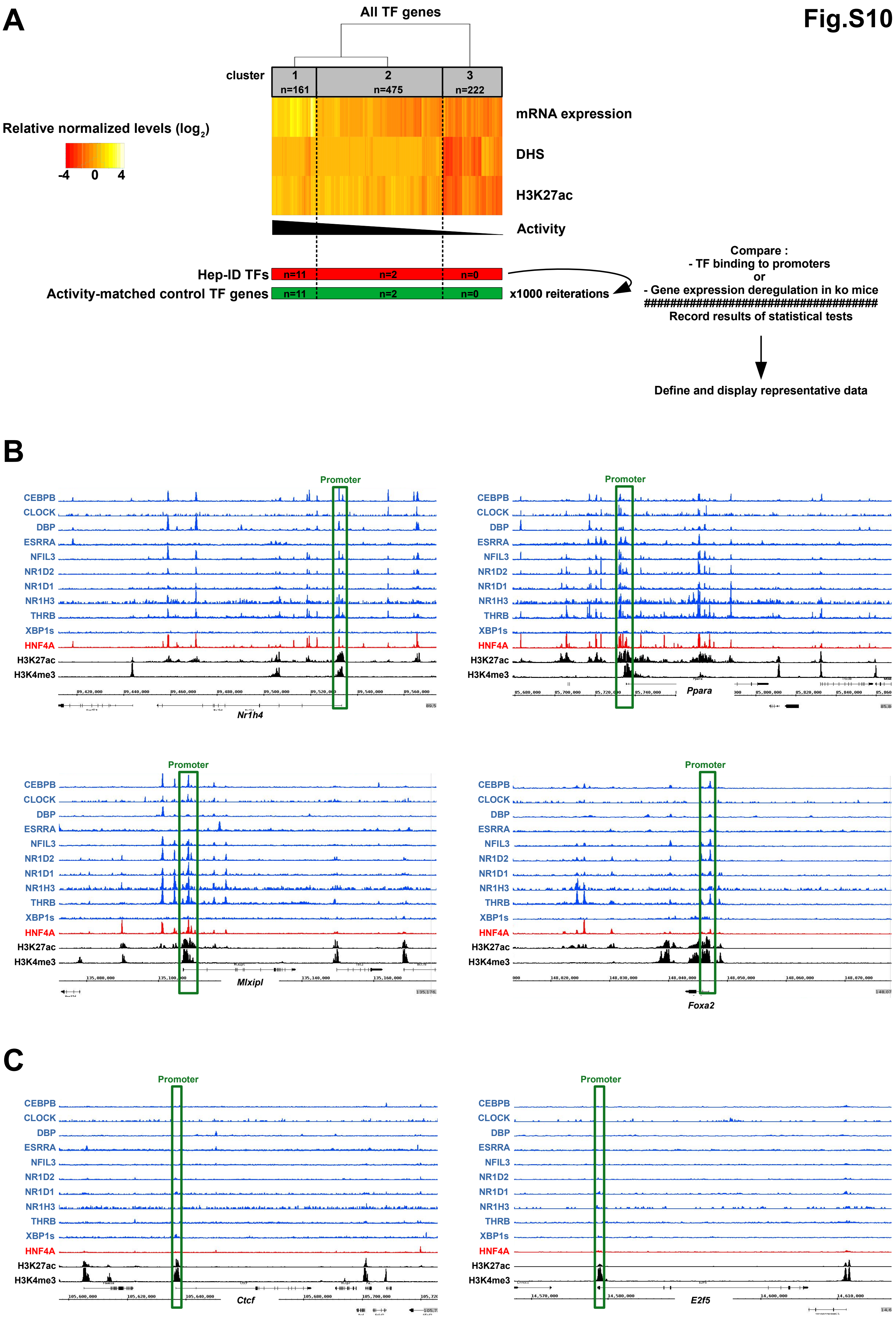
Fig.S10
A
All TF genes
cluster
1
n=161
2
n=475
3
n=222
Relative normalized levels (log2)
-4
0
4
mRNA expression
DHS
H3K27ac
Activity
Hep-ID TFs
n=11
n=2
n=0
Activity-matched control TF genes
n=11
n=2
n=0
x1000 reiterations
Compare :
- TF binding to promoters
or
- Gene expression deregulation in ko mice
######################################
Record results of statistical tests
Define and display representative data
B
Promoter
CEBPB
CLOCK
DBP
ESRRA
NFIL3
NR1D2
NR1D1
NR1H3
THRB
XBP1s
HNF4A
H3K27ac
H3K4me3
Nr1h4
Ppara
Mlxipl
Foxa2
C
Ctcf
E2f5

Appendix Figure S10. Strategy used to compare TF binding to or transcriptional regulation of Hep-ID TFs and activity-matched control TF genes.

A) TF-encoding gene promoters were clustered based on their activity in the mouse liver defined through mRNA expression levels of associated genes and DHS-seq and H3K27ac ChIP-seq signals. The hierarchical clustering tree is shown on top of the heatmap together with identification of 3 main clusters. Hep-ID and non-Hep-ID (Other) TF gene promoters among the most active ones (cluster 1) were split and compared as promoters matched for high activity in subsequent analyses. **B-C)** The Integrated Genome Browser (IGB) was used to display the cistromes of the indicated Hep-ID^{CONNECT} TFs (blue) and the Hep-ID TF HNF4A (red) together with levels of H3K4me3 and H3K27ac (black) from mouse liver ChIP-seq data (Table EV2). Example Hep-ID TF (**B**) and control TF-encoding genes (**C**) are shown. The promoters are highlighted by green boxes. The scales of the individual ChIP-seq tracks were kept constant for all analyzed genes.

Fig.S11

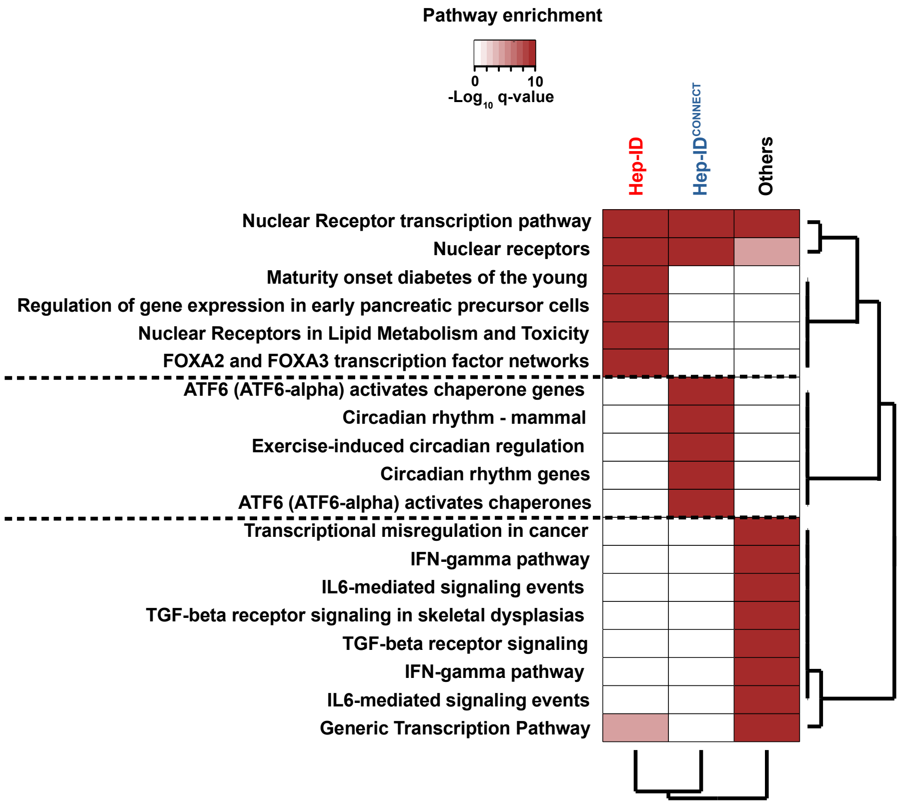

Pathway enrichment
0
10
-Log10 q-value
Hep-ID
Hep-ID CONNECT
Others
Nuclear Receptor transcription pathway
Nuclear receptors
Maturity onset diabetes of the young
Regulation of gene expression in early pancreatic precursor cells
Nuclear Receptors in Lipid Metabolism and Toxicity
FOXA2 and FOXA3 transcription factor networks
ATF6 (ATF6-alpha) activates chaperone genes
Circadian rhythm - mammal
Exercise-induced circadian regulation
Circadian rhythm genes
ATF6 (ATF6-alpha) activates chaperones
Transcriptional misregulation in cancer
IFN-gamma pathway
IL6-mediated signaling events
TGF-beta receptor signaling in skeletal dysplasias
TGF-beta receptor signaling
IFN-gamma pathway
IL6-mediated signaling events
Generic Transcription Pathway

Appendix Figure S11. Biological pathways linked to Hep-ID, Hep-ID^{CONNECT} and remaining TF-encoding genes from cluster G (Others).

Biological pathways associated with Hep-ID, Hep-ID^{CONNECT} and remaining TF-encoding genes from cluster G (Others) were defined using ToppCluster. When several pathways had an identical name, only the one with the greatest q-value was used. Dendrograms of hierarchical clustering are shown. ToppCluster uses hypergeometric tests and Bonferroni correction.

Fig.S12

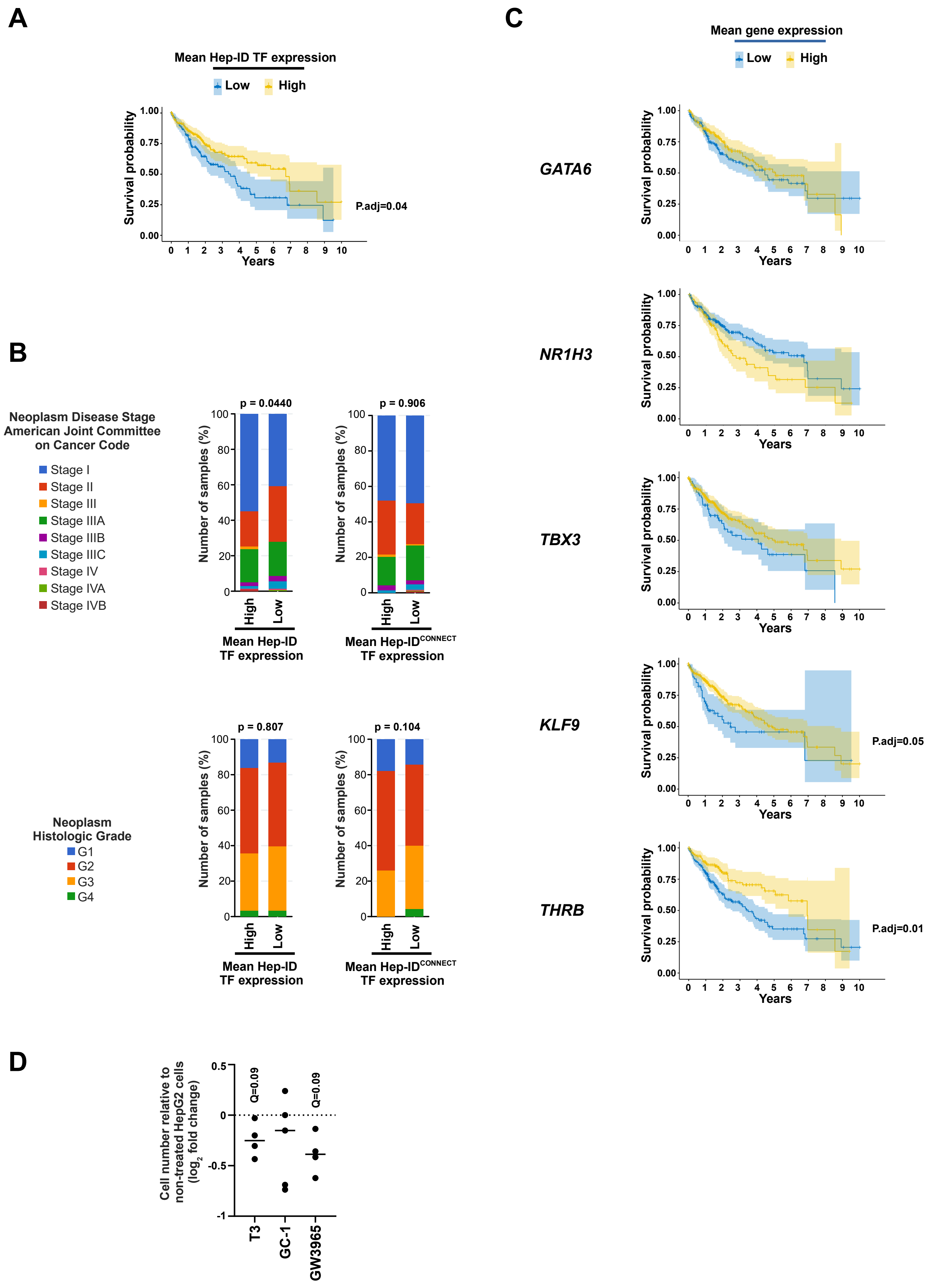
A
Mean Hep-ID TF expression
Low
High
Survival probability
1.00
0.75
0.50
0.25
0.00
Years
P.adj=0.04
B
Neoplasm Disease Stage American Joint Committee on Cancer Code
Stage I
Stage II
Stage III
Stage IIIA
Stage IIIB
Stage IIIC
Stage IV
Stage IVA
Stage IVB
p = 0.0440
p = 0.906
Number of samples (%)
High
Low
Mean Hep-ID TF expression
Mean Hep-ID CONNECT TF expression
Neoplasm Histologic Grade
G1
G2
G3
G4
p = 0.807
p = 0.104
C
Mean gene expression
Low
High
GATA6
NR1H3
TBX3
KLF9
P.adj=0.05
THRB
P.adj=0.01
D
Cell number relative to non-treated HepG2 cells (log2 fold change)
0.5
0
-0.5
-1
Q=0.09
Q=0.09
T3
GC-1
GW3965

Appendix Figure S12. Clinical features associated with HCC displaying low or high expression levels of Hep-ID^{CONNECT} TF genes.

A) Overall survival of patients with HCC expressing low or high levels of the Hep-ID TF encoding genes. Differential overall survival analysis was assessed by Kaplan-Meier (KM) log rank adjusted for 100 permutations (Cheng *et al*, 2022). **B)** Distribution of the stages or grades of HCC with low or high average expression levels of Hep-ID or Hep-ID^{CONNECT} TF genes. Chi-squared tests were used to assess statistical significance. **C)** Similar analyses to that described in panel A performed using individual Hep-ID^{CONNECT} TF encoding genes. Overall, results were less significant here indicating that mining Hep-ID and Hep-ID^{CONNECT} TF genes as gene sets provided robustness to this type of analysis. Nevertheless, high THRB expression was the most significantly associated with greater 10-year overall survival. Note that lack of similar findings for NR1H3 may stem from the role exerted by NR1H3 not only in tumor cells but also in cells of the tumor microenvironment such as macrophages (Zhou *et al*. 2022). **D)** HepG2 cells were treated with T3, GC-1 or GW3965 for 7 days. Viable cell number was determined (based on 3 technical replicates) and expressed relative to that obtained for control conditions (untreated or exposed to DMSO). Data are shown as scatter plot with dots displaying the results obtained from independent biological replicates and the horizontal lines showing the median. One-sample t-test with Benjamini-Hochberg correction for multiple testing was used to determine if the mean log2 FC was statistically different from 0.

Fig.S13

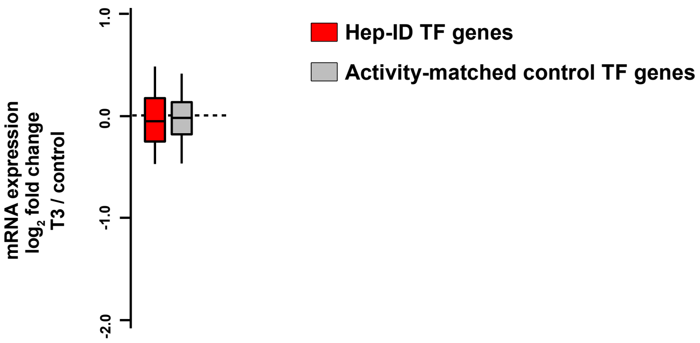
mRNA expression
log2 fold change
T3 / control
1.0
0.0
-1.0
-2.0
Hep-ID TF genes
Activity-matched control TF genes

Appendix Figure S13. T3-mediated transcriptional regulation of Hep-ID TF genes in livers of healthy mice.

Modulation of Hep-ID TF gene expression in the mouse liver upon T3 injection for 3 days. Analyses were performed as in Fig.4J.

Fig.S14

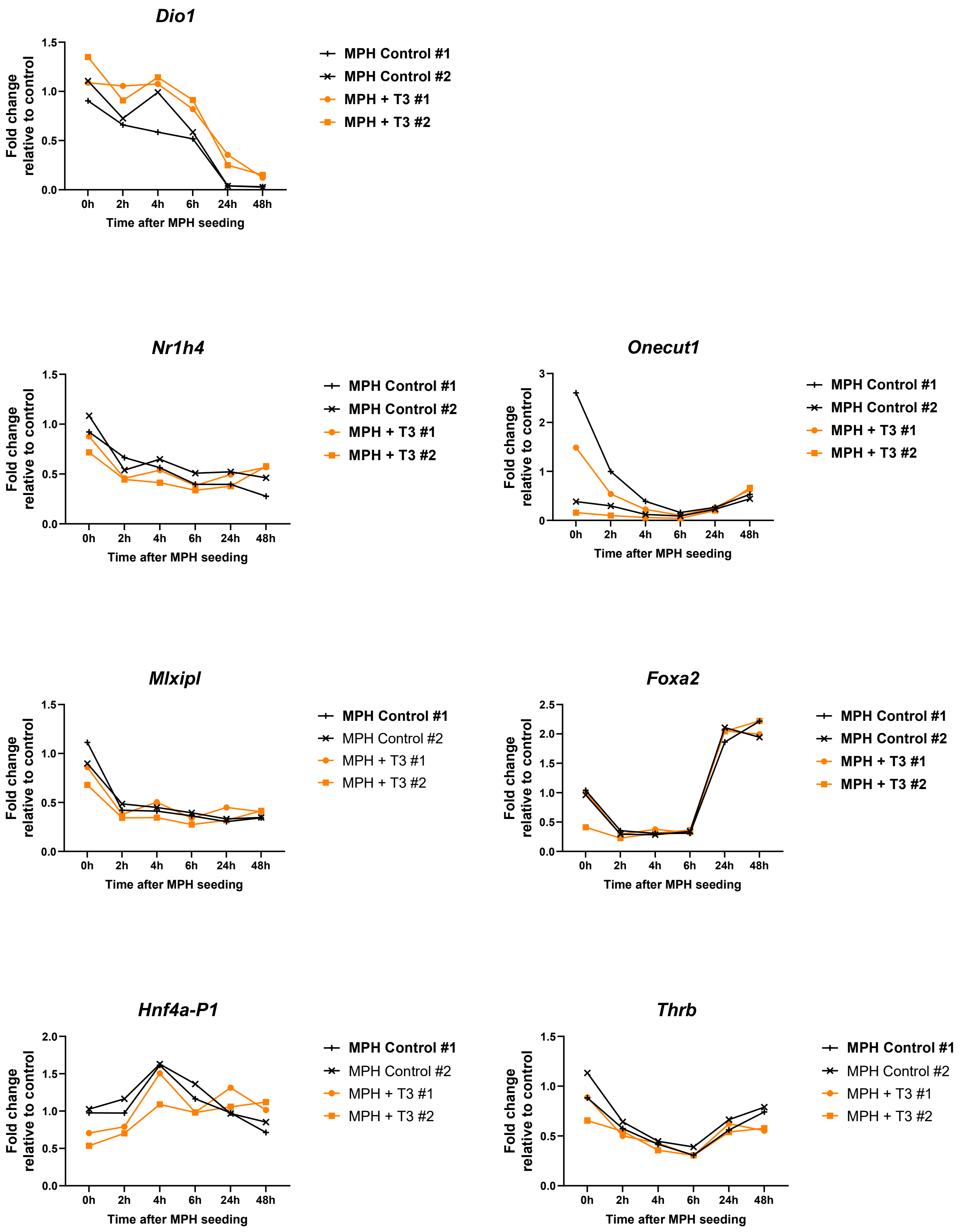
Dio1
Fold change relative to control
1.5
1.0
0.5
0.0
0h 2h 4h 6h 24h 48h
Time after MPH seeding
MPH Control #1
MPH Control #2
MPH + T3 #1
MPH + T3 #2
Nr1h4
Fold change relative to control
1.5
1.0
0.5
0.0
0h 2h 4h 6h 24h 48h
Time after MPH seeding
MPH Control #1
MPH Control #2
MPH + T3 #1
MPH + T3 #2
Onecut1
Fold change relative to control
3
2
1
0
0h 2h 4h 6h 24h 48h
Time after MPH seeding
MPH Control #1
MPH Control #2
MPH + T3 #1
MPH + T3 #2
Mlxipl
Fold change relative to control
1.5
1.0
0.5
0.0
0h 2h 4h 6h 24h 48h
Time after MPH seeding
MPH Control #1
MPH Control #2
MPH + T3 #1
MPH + T3 #2
Foxa2
Fold change relative to control
2.5
2.0
1.5
1.0
0.5
0.0
0h 2h 4h 6h 24h 48h
Time after MPH seeding
MPH Control #1
MPH Control #2
MPH + T3 #1
MPH + T3 #2
Hnf4a-P1
Fold change relative to control
2.0
1.5
1.0
0.5
0.0
0h 2h 4h 6h 24h 48h
Time after MPH seeding
MPH Control #1
MPH Control #2
MPH + T3 #1
MPH + T3 #2
Thrb
Fold change relative to control
1.5
1.0
0.5
0.0
0h 2h 4h 6h 24h 48h
Time after MPH seeding
MPH Control #1
MPH Control #2
MPH + T3 #1
MPH + T3 #2

Appendix Figure S14. T3-mediated transcriptional regulation of Hep-ID TF genes in MPH.

MPH were isolated in the presence or not of T3 (n=2 independent MPH preparation per condition), seeded in cell culture plates and harvested at the indicated times. mRNA expression of the indicated genes was monitored using RT-qPCR. The graphs show how gene expression evolved with time in the different MPH preparations (average expression in non-treated MPH at 0h was set to 1).
