# Supplementary material for: An extended transcription factor regulatory network controls hepatocyte identity
Source: EMBO Rep. 2023 Jul 10;24(9):e57020. doi: 10.15252/embr.202357020 (PMC10481658; doi:10.15252/embr.202357020)
Supplement: Supplementary file 2 — Expanded View Figures PDF [file EMBR-24-e57020-s003.pdf]

Expanded View Figures

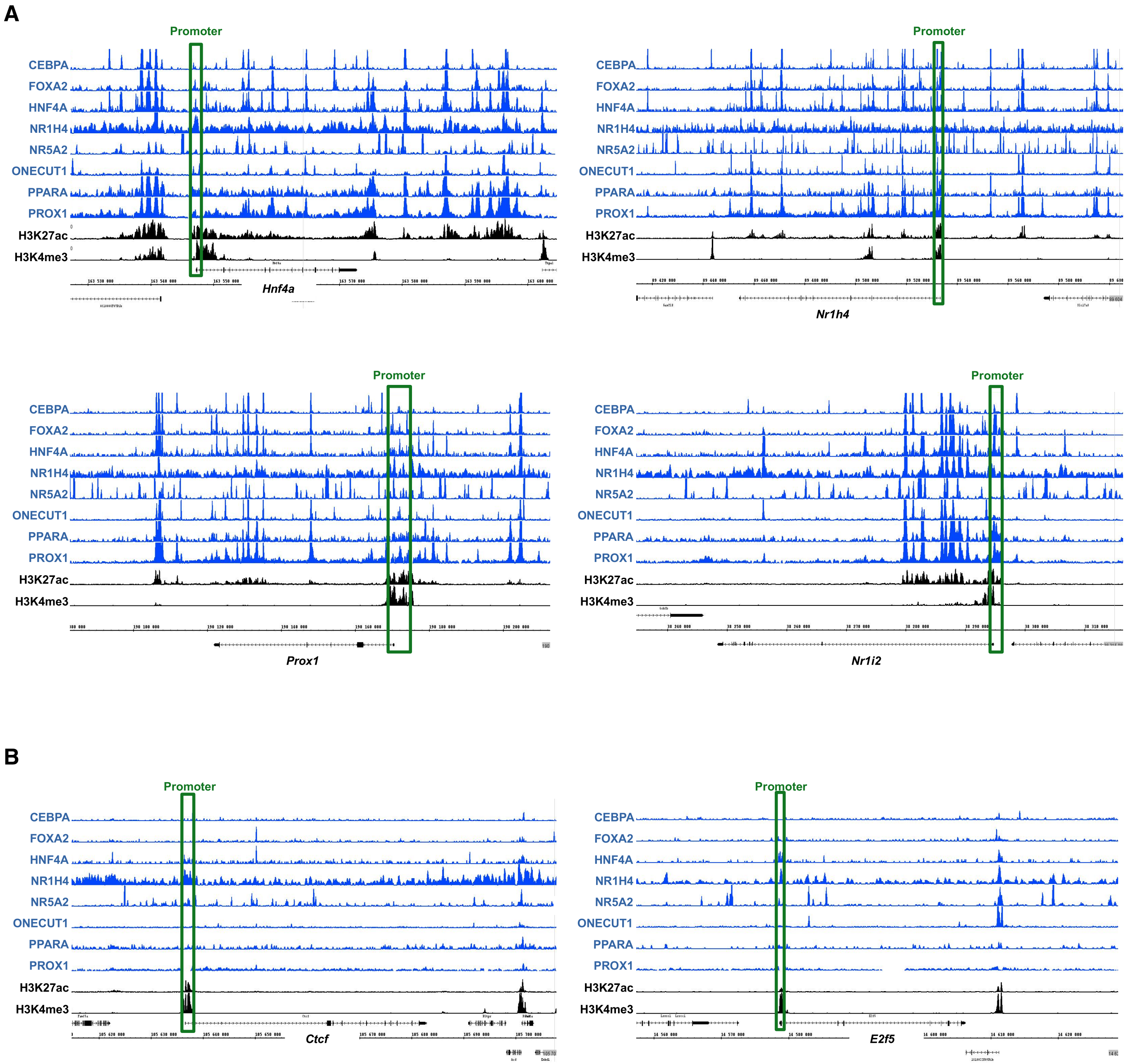

Figure EV1. Hep-ID TF cistromes at example TF-encoding gene loci.

A, B The Integrated Genome Browser (IGB) was used to display the cistromes of the indicated eight Hep-ID TFs together with levels of H3K4me3 and H3K27ac from mouse liver ChIP-seq data (Dataset EV2). Example Hep-ID TF (A) and control TF-encoding genes (B) are shown. The promoters are highlighted by green boxes. The scales of the individual ChIP-seq tracks were kept constant for all analyzed genes.

© 2023 The Authors

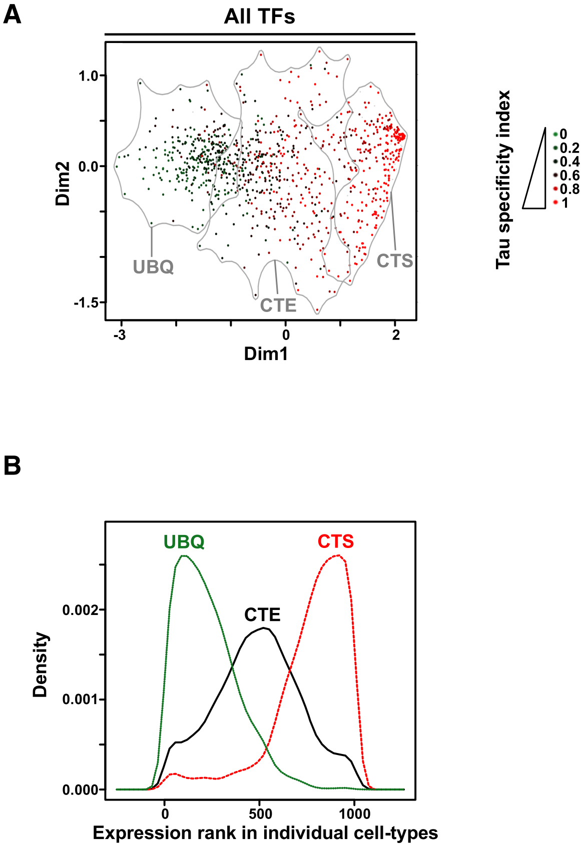

Figure EV2. Characterization of CTS, CTE, and UBQ TF genes.

A Data were displayed as in Fig 1J to show the Tau index of tissue-specific expression for individual TF genes within the CTS (cell-type specific), CTE (cell-type enriched), and UBQ (ubiquitous) clusters.

B Density plot showing the distribution of the expression rank of CTS, CTE, and UBQ TF-encoding genes in primary mouse cell-types (n = 39). All TF genes were ranked from high to low expression (i.e., from 0 to 1,009 in each cell-type) and the distribution of TFs from the CTS, CTE and UBQ groups are shown. As expected, CTS TFs display low ranks in a very limited subsets of cell-types while having high ranks in most cell-types, which is the opposite from the pattern obtained for UBQ TFs.

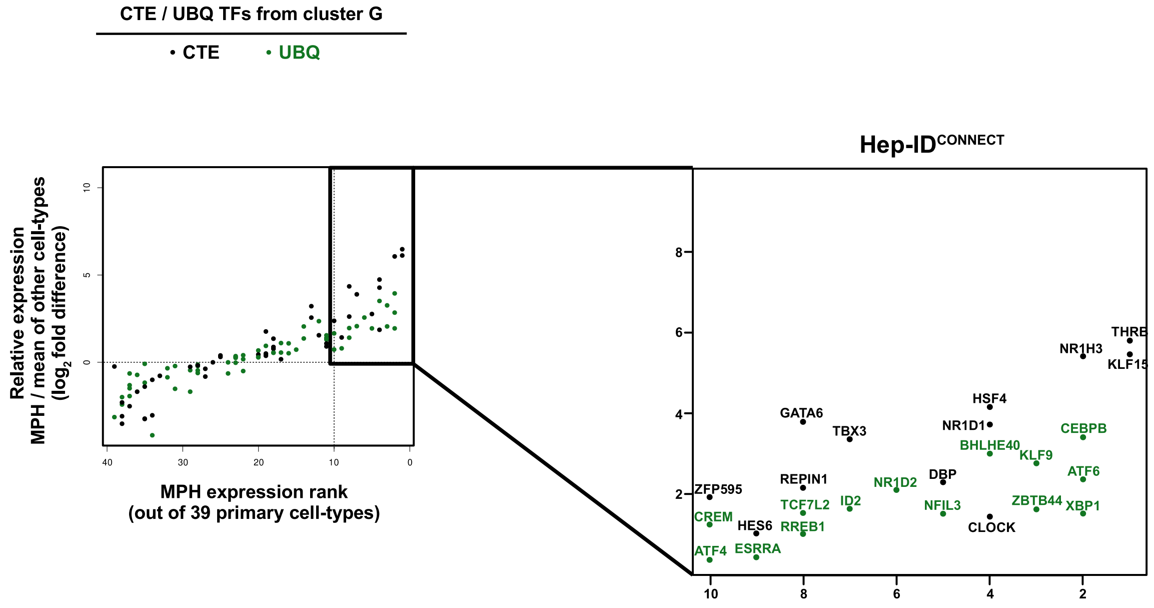

Figure EV3. CTE and UBQ TF genes with privileged expression in MPH.

The right shows a zoomed view of CTE (black) and UBQ (green) TFs from cluster G comprised within the framed area from Fig 2A (shown again on the left).

© 2023 The Authors

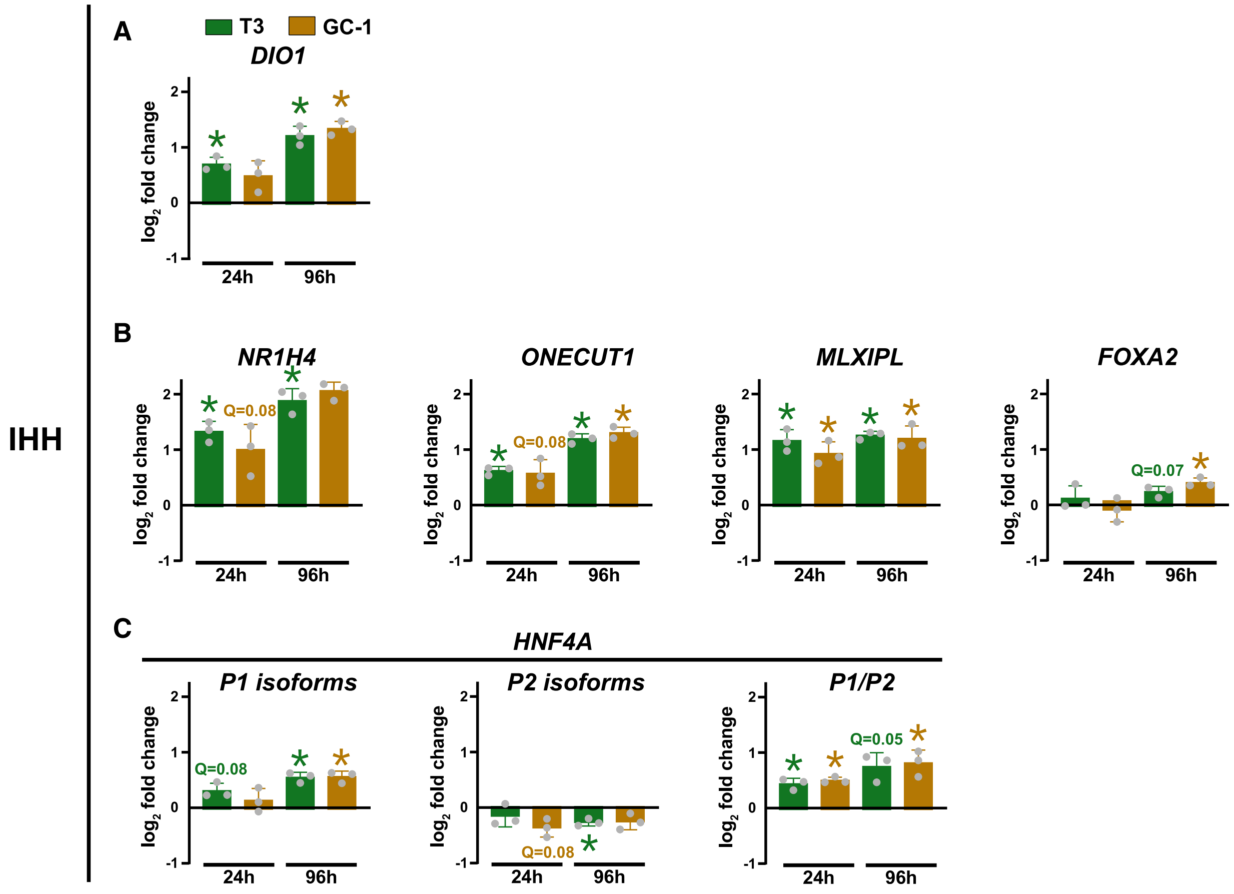

Figure EV4. T3-mediated regulation of Hep-ID TF gene expression in human IHH cells.

A–C mRNA expression of the indicated genes was monitored using RT-qPCR in IHH cells treated with T3 or GC-1 for 24 or 96 h. Bar graphs show mean ± SD ($n = 3$ biological replicates) of $\log_2$ fold changes in treated *versus* untreated HepG2 cells. For *Hnf4a*, the $\log_2$ fold change in the ratio of P1 over P2 promoter-derived isoforms is also shown. Gray dots show the results obtained from the three independent biological replicates. One-sample *t*-test with Benjamini–Hochberg correction for multiple testing was used to determine if the mean $\log_2$ FC was statistically different from 0. *$q < 0.05$.

Figure EV5. Acute IL1B challenge triggers partial hepatic loss of identity.

- A Analysis similar to that shown in Appendix Fig S6 showing that IL1B treatment induced a hepatic transcriptomic profile leaning towards that of not fully mature hepatocytes pointing to partial dedifferentiation.
- B Dot plots showing the transcriptional regulation of individual Hep-ID TF gene expression in livers of IL1B-challenged mice compared to non-treated animals issued from transcriptomic analyses.
- C Correlation between *Dio1* and *Ccl2* mRNA expression levels assessed using RT-qPCR and livers of all mice treated with IL1B + T3 from Fig 5. Gene expression are $\log_2$ FC relative to control (PBS injected) mice. Linear regression and coefficient of determination (r^2) are shown.
- D Gene expression levels of *Ly6g* (neutrophil marker) and *Ptprc* (also known as *CD45*; broad immune cell marker) were analyzed as described for Fig 5B–D. Box plots are composed of a box from the 25th to the 75th percentile with the median as a line ($n = 13$ mice for the PBS group, 17 for the IL1B group and 10 for the other groups). Whiskers extent to the most extreme data point which is no more than 1.5 times the interquartile range from the box.

© 2023 The Authors

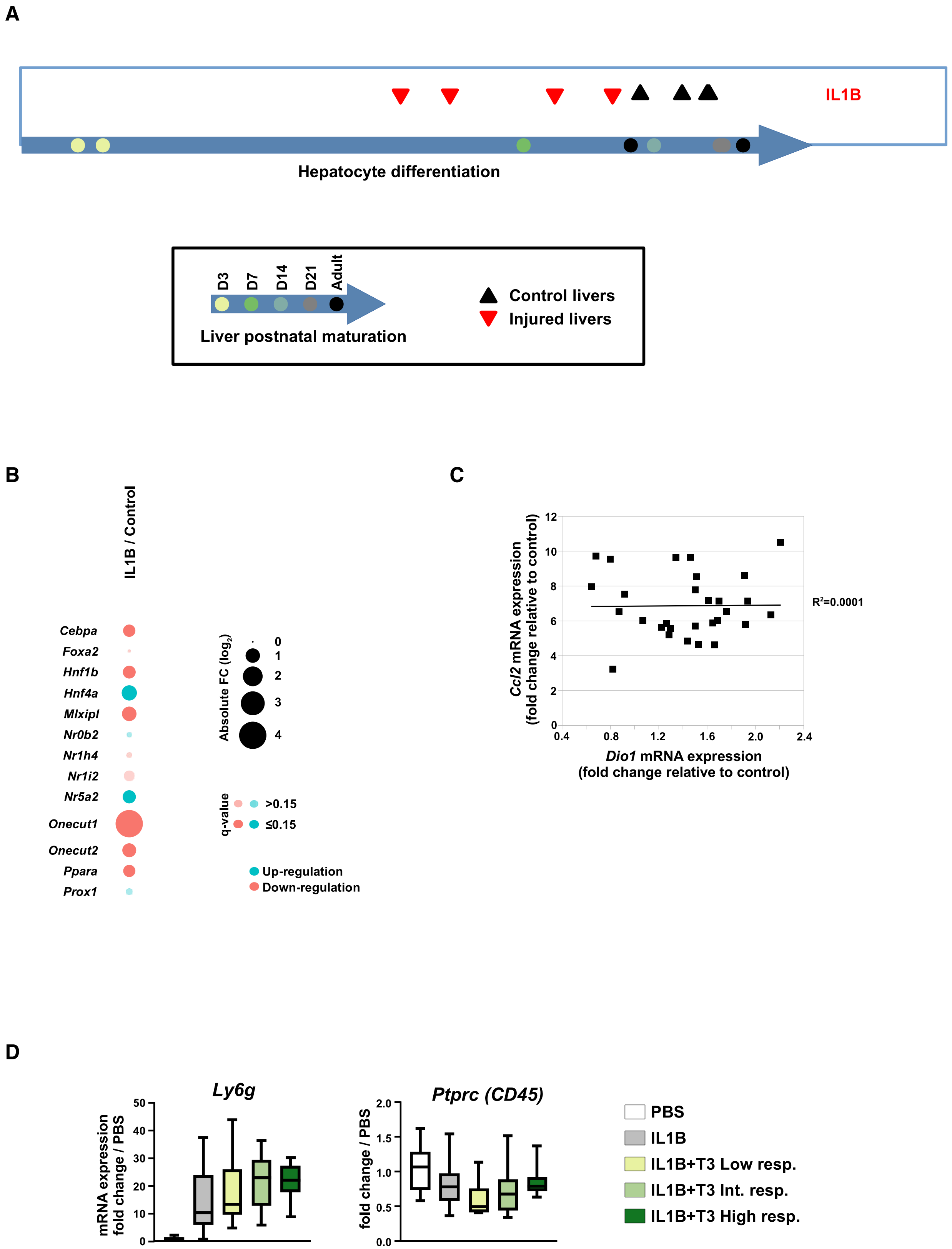

Figure EV5.

 © 2023 The Authors
