# Supplementary material for: An extended transcription factor regulatory network controls hepatocyte identity
Source: EMBO Rep. 2023 Jul 10;24(9):e57020. doi: 10.15252/embr.202357020 (PMC10481658; doi:10.15252/embr.202357020)
Supplement: Supplementary file 10 — PDF+ [file EMBR-24-e57020-s001.pdf]

Article

SOURCE DATA | TRANSPARENT PROCESS | OPEN ACCESS

EMBO reports

# An extended transcription factor regulatory network controls hepatocyte identity

Julie Dubois-Chevalier[1], Céline Gheeraert[1], Alexandre Berthier[1], Clémence Boulet[1], Vanessa Dubois[1,2], Loïc Guille[1], Marie Fourcot[3], Guillemette Marot[4], Karine Gauthier[5], Laurent Dubuquoy[6], Bart Staels[1], Philippe Lefebvre[1,†] & Jérôme Eeckhoute[1,*,†]

## Abstract

**Cell identity is specified by a core transcriptional regulatory circuitry (CoRC), typically limited to a small set of interconnected cell-specific transcription factors (TFs). By mining global hepatic TF regulons, we reveal a more complex organization of the transcriptional regulatory network controlling hepatocyte identity. We show that tight functional interconnections controlling hepatocyte identity extend to non-cell-specific TFs beyond the CoRC, which we call hepatocyte identity (Hep-ID)$^{CONNECT}$ TFs. Besides controlling identity effector genes, Hep-ID$^{CONNECT}$ TFs also engage in reciprocal transcriptional regulation with TFs of the CoRC. In homeostatic basal conditions, this translates into Hep-ID$^{CONNECT}$ TFs being involved in fine tuning CoRC TF expression including their rhythmic expression patterns. Moreover, a role for Hep-ID$^{CONNECT}$ TFs in the control of hepatocyte identity is revealed in dedifferentiated hepatocytes where Hep-ID$^{CONNECT}$ TFs are able to reset CoRC TF expression. This is observed upon activation of NR1H3 or THRB in hepatocarcinoma or in hepatocytes subjected to inflammation-induced loss of identity. Our study establishes that hepatocyte identity is controlled by an extended array of TFs beyond the CoRC.**

**Keywords** cell identity; core regulatory network; hepatocyte dedifferentiation; liver disease; transcription factors
**Subject Categories** Transcription & Genomics; Molecular Biology of Disease
**DOI** 10.15252/embr.202357020 | Received 16 February 2023 | Revised 16 June 2023 | Accepted 21 June 2023 | Published online 10 July 2023
**EMBO Reports (2023) 24: e57020**

## Introduction

Multicellular organisms are built upon collaborative activities of phenotypically and functionally distinct cell-types. Individual cell identities and functions are acquired thanks to the activity of cell-specific transcription factors (TFs). Indeed, cell-specific TFs directly control the expression of non-TF genes exerting activities/functions characterizing individual cell-types, alternatively known as identity effector genes (Arendt *et al*, 2016; Almeida *et al*, 2021). A limited set of identity TFs engaging into auto- and cross-regulatory loops typically defines the core transcriptional regulatory circuitry (CoRC; Arendt *et al*, 2016; Almeida *et al*, 2021). CoRCs are considered cornerstones for establishment and maintenance of cell identities (Arendt *et al*, 2016; Almeida *et al*, 2021). Indeed, CoRCs allows to self-sustain high expression of identity TFs and their target effector genes in addition to being involved in the continuous modulation of the hepatic transcriptome in response to environmental stimuli (Boyer *et al*, 2005; Arendt *et al*, 2016; Wilkinson *et al*, 2017; Almeida *et al*, 2021).

In line, hepatocyte identity is typically defined as relying on a handful of interconnected hepatocyte-specific CoRC TFs (hereafter called Hep-ID TFs) including HNF4A, FOXA or NR1H4 (FXR; Tachmatzidi *et al*, 2021). However, many additional TFs beyond these hepatic identity CoRC TFs have been ascribed with roles in the control of hepatocyte activities (Bideyan *et al*, 2021). However, whether and how these additional TFs exert their functions in concert with the hepatic CoRC is not fully understood. Moreover, considering that sustained expression of Hep-ID TFs has a positive impact on hepatic pathological conditions (Berasain *et al*, 2022), better defining how CoRC TFs are functionally linked to additional TFs is of pathophysiological interest. Indeed, breakdown in liver functions in advanced stages of liver injuries and in cancer results from hepatocyte loss of identity stemming from compromised expression of the hepatic CoRC TFs (Dubois *et al*, 2020a; Berasain *et al*, 2022).

As outlined hereabove, apprehending the transcriptional regulation of cell identity through the lens of CoRCs, by definition, only leads to consider the role of a very limited subset of TFs. In this context, the real architecture and outline of the hepatocyte identity TF network has not been clearly established beyond Hep-ID TFs. We postulated that focusing on TF interconnections at the genomic-

1 Univ. Lille, Inserm, CHU Lille, Institut Pasteur de Lille, U1011-EGID, Lille, France
2 Basic and Translational Endocrinology (BaTE), Department of Basic and Applied Medical Sciences, Ghent University, Ghent, Belgium
3 Univ. Lille, CNRS, Inserm, CHU Lille, Institut Pasteur de Lille, US 41 – UAR 2014 – PLBS, Lille, France
4 Univ. Lille, Inria, CHU Lille, ULR 2694 – METRICS: Évaluation des technologies de santé et des pratiques médicales, Lille, France
5 Institut de Génomique Fonctionnelle de Lyon (IGFL), CNRS UMR 5242, INRAE USC 1370, École Normale Supérieure de Lyon, Lyon, France
6 Univ. Lille, Inserm, CHU Lille, U1286 – INFINITE – Institute for Translational Research in Inflammation, Lille, France
*Corresponding author. Tel: 33(0)3 20 97 42 20; Fax: 33(0)3 20 97 42 01; E-mail: jerome.eeckhoute@inserm.fr
†These authors contributed equally to this work

© 2023 The Authors. Published under the terms of the CC BY 4.0 license.

scale would allow to define how the Hep-ID TF network spreads beyond the CoRC and would refine our understanding of hepatocyte identity transcriptional control.

## Results

### Hepatocyte identity TFs extensively co-bind TF-encoding gene promoters beyond the CoRC

We defined the hepatocyte CoRC as a set of 13 Hep-ID TFs commonly defined in different studies as interconnected hepatocyte-specific TFs (Kyrmizi *et al*, 2006; D'Alessio Ana *et al*, 2015; Zhou *et al*, 2017; Dubois *et al*, 2020a; Dataset EV1). Hep-ID TFs comprise the well-accepted and thoroughly experimentally verified drivers of hepatocyte identity (Reizel *et al*, 2020; Tachmatzidi *et al*, 2021). As expected from our previous studies (Dubois-Chevalier *et al*, 2017b, 2020; Dubois *et al*, 2020a), monitoring the mouse liver cistromes of eight Hep-ID TFs (CEBPA, FOXA2, HNF4A, NR1H4, NR5A2, ONECUT1, PPARA, PROX1; Dataset EV2) pointed to their extensive co-recruitment at the Hep-ID TF-encoding gene promoters when compared to a control set of non-Hep-ID TF-encoding gene promoters (Figs 1A and EV1A and B). This led us to define a strategy called Promoter-centric TF network analysis (ProTFnet; Fig 1B) with the aim to establish global TF interconnections through promoter binding patterns. Indeed, this approach consists in mining TF cistromes to identify TF binding to all TF-encoding gene promoters. Here, hepatic TF cistromes ($n$ = 49; including those of Hep-ID TFs and extending to transcriptional cofactors; Dubois-Chevalier *et al*, 2017b; Dubois *et al*, 2020a) were used to monitor the binding to all TF-encoding gene promoters active in the mouse liver ($n$ = 925 transcriptionally active promoters, i.e., TF-encoding genes with detectable expression levels; see Materials and Methods for details). These promoters were grouped together based on their TF-binding pattern similarity, that is, cistromic-based classification, using a self-organizing map (SOM; Appendix Fig S1A–D; Dubois-Chevalier *et al*, 2017a). Hierarchical clustering was next performed and identified seven main clusters of TF-encoding gene promoters (Fig 1C and Appendix Fig S2A). We annotated these clusters A–G based on progressive TF co-recruitment pattern complexity (Fig 1C and D, Appendix Figs S2B and C, and S3A–G). Promoters from cluster G were also overall the most strongly active as revealed by chromatin accessibility (DHS), hepatic histone acetylation (H3K27ac) or mRNA expression levels of associated TF-encoding genes (Fig 1E and Appendix Fig S4A–C). In line, cluster G was enriched for Hep-ID TF-encoding genes (odds ratio = 5.2, $P$ = 0.006). Moreover, Hep-ID TF binding was also most pronounced at promoters from cluster G, both when considered individually (Appendix Figs S2D and E, and S3G) or collectively, i.e., Hep-ID TF co-recruitment (Fig 1F). Therefore, cluster G is the prominent subset of promoters capturing hepatic CoRC auto−/cross-binding. Despite being enriched, TF-encoding genes captured by cluster G were not limited to those displaying the strongest and most liver-specific expression (Fig 1G and H).

To further characterize the TF-encoding gene heterogeneity in cluster G, we performed a meta-analysis of transcriptomic data from primary human and mouse cells ($n$ = 126 and 39 different cell-types including hepatocytes, respectively) to define three groups of TFs displaying cell-type specific (CTS), cell-type enriched (CTE) or ubiquitous (UBQ) expression patterns (Figs 1I and EV2A and B; see Materials and Methods). This further highlighted that, besides Hep-ID TFs and an additional set of cell-specific TFs with well characterized hepatic functions (*Nr1i3/Car*, *Rorc/Rorg*, *Hnf1a* and *Gata4*; Tachmatzidi *et al*, 2021), the majority of TFs from cluster G belonged to CTE or UBQ TFs (Fig 1J and Dataset EV1).

Altogether, these analyses indicated that extensive co-recruitment of Hep-ID TFs is not limited to their own highly hepatocyte-specific genes within the CoRC but extends to an additional set of TFs.

### Hep-ID$^{CONNECT}$ TFs are expressed under the control of and collaborate with Hep-ID TFs in the regulation of hepatocyte identity effector genes

In order to further characterize the CTE/UBQ TFs from cluster G, we refined our analysis of their individual expression patterns by defining how their levels of expression in mouse primary hepatocytes (MPH) compare to those in other primary cells ($n$ = 38 different cell-types). Interestingly, we found that a subset of the CTE/UBQ TFs from cluster G, hereafter called Hep-ID$^{CONNECT}$ TFs, was nevertheless characterized by privileged hepatocyte expression (i.e., MPH ranked among the top 10 expressing cells and expression in MPH above average expression in other cells; Figs 2A and EV3). TF-encoding genes whose ranking was beyond this conservative cut-off can be retrieved from Table EV1. Reminiscent of Hep-ID TF-encoding genes, expression of Hep-ID$^{CONNECT}$ TFs was increased in the final hepatocyte differentiation stage occurring during liver post-natal maturation (Fig 2B) and mining single-nuclei RNA-seq data from adult mouse and human livers confirmed privileged expression in hepatocytes (Appendix Fig S5A and B). These observations suggested that Hep-ID$^{CONNECT}$ TFs might be directly dependent upon Hep-ID TFs for their enhanced hepatocyte expression. In line, expression of Hep-ID$^{CONNECT}$ TFs was decreased in transcriptomic data obtained from MPH of adult mice with hepatocyte-specific deletion of the Hep-ID TF *Hnf4a* (Fig 2C). We and others have established that compromised hepatocyte identity due to decreased Hep-ID TF gene expression is commonly found in severe liver injuries in both mouse models and humans (Argemi *et al*, 2019; Hyun *et al*, 2020; Dubois *et al*, 2020a; Bou Saleh *et al*, 2021; Loft *et al*, 2021; Berasain *et al*, 2022; Gunewardena *et al*, 2022). Therefore, we further mined Hep-ID$^{CONNECT}$ TF expression in a meta-analysis of transcriptomic data obtained from mouse liver injury models triggering hepatocyte dedifferentiation (Appendix Fig S6). Interestingly, we found that Hep-ID$^{CONNECT}$ TFs showed reduced expression levels in the livers of these mouse models (Fig 2D). Similar observations were made using transcriptomic data obtained from microdissected hepatocytes from alcohol-related human liver cirrhosis (Bou Saleh *et al*, 2021; Fig 2E). Overall, observed changes in expression of Hep-ID$^{CONNECT}$ TFs were reminiscent of those of Hep-ID TFs, albeit often with a lower amplitude, and different from those of other TFs from cluster G. A similar observation was made when interrogating the breadth of H3K4me3 labelling at Hep-ID$^{CONNECT}$ TF gene promoters, a feature positively linked to the role of TFs in the control of cell identity (Pekowska *et al*, 2010; Benayoun *et al*, 2014; Chen *et al*, 2015). Indeed, the breadth of H3K4me3 labeling at the promoters of Hep-ID$^{CONNECT}$ TF genes was also comprised in between that observed for the Hep-ID TF and other TF-encoding

 © 2023 The Authors

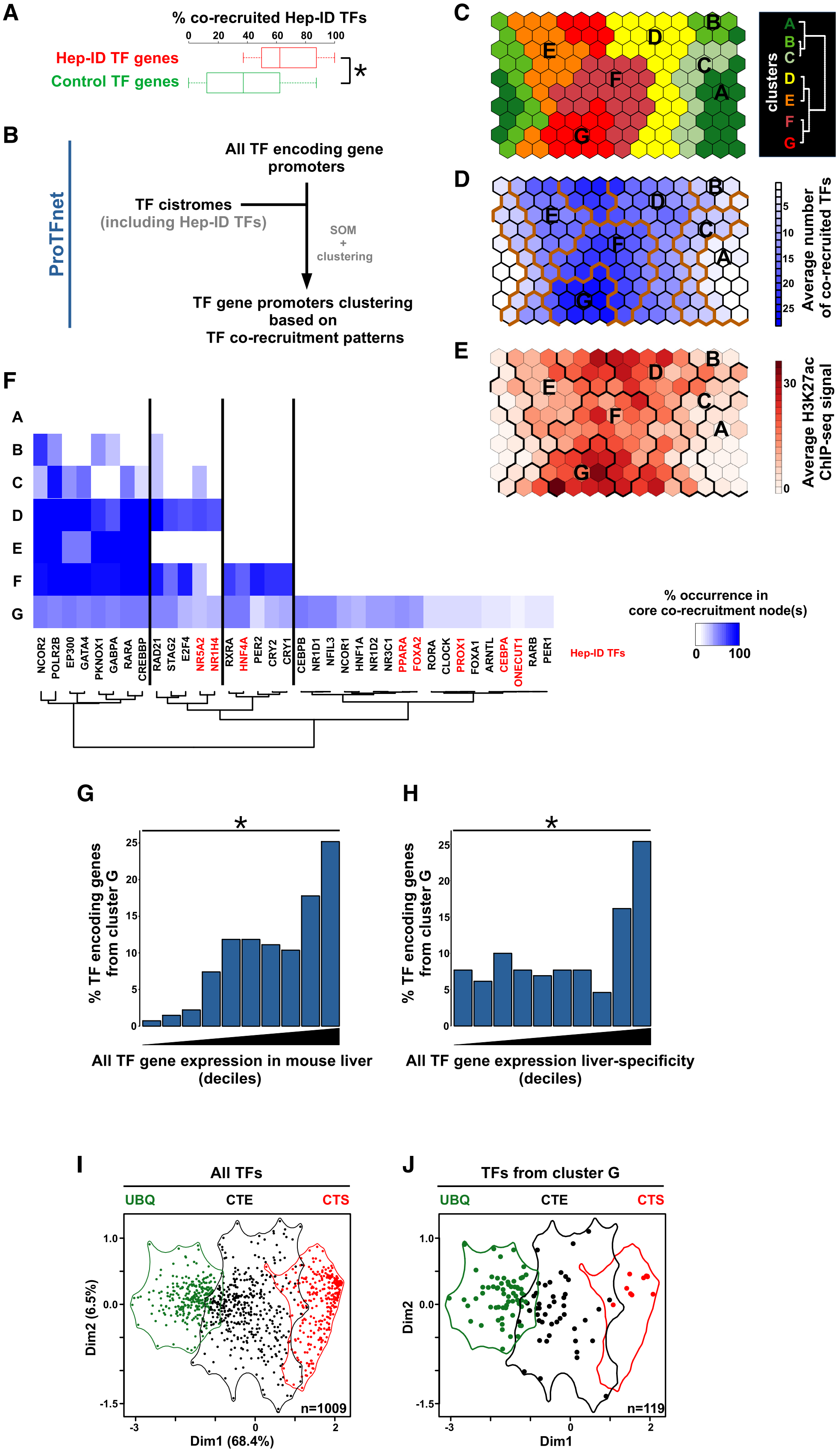
A
% co-recruited Hep-ID TFs
Hep-ID TF genes
Control TF genes
B
ProTFnet
All TF encoding gene promoters
TF cistromes (including Hep-ID TFs)
SOM + clustering
TF gene promoters clustering based on TF co-recruitment patterns
C
clusters
D
Average number of co-recruited TFs
E
Average H3K27ac ChIP-seq signal
F
% occurrence in core co-recruitment node(s)
Hep-ID TFs
NCOR2 POLR2B EP300 GATA4 PKNOX1 GABPA RARA CREBBP RAD21 STAG2 E2F4 NR5A2 NR1H4 RXRA HNF4A PER2 CRY2 CRY1 CEBPB NR1D1 NFIL3 NCOR1 HNF1A NR1D2 NR3C1 PPARA FOXA2 RORA CLOCK PROX1 FOXA1 ARNTL CEBPA ONECUT1 RARB PER1
G
% TF encoding genes from cluster G
All TF gene expression in mouse liver (deciles)
H
All TF gene expression liver-specificity (deciles)
I
All TFs
UBQ
CTE
CTS
Dim2 (6.5%)
Dim1 (68.4%)
n=1009
J
TFs from cluster G
Dim2
Dim1
n=119

**Figure 1.**

◀ **Figure 1. Promoter-centric mining of the hepatic TF network using ProTFnet.**

A The cistromes of 8 Hep-ID TFs (CEBPA, FOXA2, HNF4A, NR1H4, NR5A2, ONECUT1, PPARA, PROX1; Dataset EV2) were used to define the percentage of those TFs binding to Hep-ID TF ($n$ = 13 genes) or non-Hep-ID TF (control TF genes; $n$ = 13) encoding gene promoters. The control group used was selected for providing data representative of those obtained with 1,000 reiterations of this analysis. Box plots are composed of a box from the $25^{th}$ to the $75^{th}$ percentile with the median as a line. Whiskers extent to the most extreme data point which is no more than 1.5 times the interquartile range from the box. Two-sided Wilcoxon rank sum test with continuity correction was used to assess statistical significance. *$P < 0.05$.

B Overview of the ProTFnet strategy implemented in this study where (identity) TF binding to TF-encoding gene promoters is monitored and subsequently used to define distinct clusters of promoters through SOM and hierarchical clustering. Clusters are subsequently characterized using multi-omics (cistromic, epigenomic and transcriptomic) data mining to explore the complexity of the identity TF network.

C Planar view of the toroidal map issued from the SOM analysis was used here to display clusters A–G and hereafter to visualize different features of these clusters (panels D, E and Appendix Fig S2E). The dendrogram issued from the hierarchical clustering analysis is shown on the right.

D The map issued from the SOM analyses was used to show the average number of co-recruited TFs at gene promoters contained in individual cells. Bold orange lines indicate the borders of clusters A–G.

E The map issued from the SOM analyses was used to show the average ChIP-seq signal for mouse liver H3K27ac at gene promoters contained in individual cells. Bold black lines indicate the borders of clusters A–G.

F Heatmap showing the occurrence (percentage) of individual transcriptional regulators in the core co-recruitment nodes of clusters A–G, that is, binding combinations found in at least 50% of the promoters of a given cluster.

G, H (G) All TF-encoding genes were grouped into deciles based on increasing expression levels in the mouse liver. Then, the distribution of TF-encoding genes from cluster G within these deciles was plotted. Two-sided two-sample Kolmogorov–Smirnov test was used to assess statistically significant bias in the distribution of genes from cluster G when compared to all other TF-encoding genes. *$P < 0.05$. (H) All TF-encoding genes were grouped into deciles based on increasing liver-specific expression levels (i.e., expression in mouse liver compared to average expression in other organs). Then, the distribution of TF-encoding genes from cluster G within these deciles was plotted. Statistical analysis was performed as in panel H.

I Principal component analysis (PCA) of TF gene expression in mouse ($n$ = 39) and human ($n$ = 126) primary cell-types (see Materials and Methods). Individual TFs are displayed as dots projected on the first two components and the three main clusters issued from hierarchical clustering are shown and labeled as UBQ (ubiquitous), CTE (cell-type enriched) and CTS (cell-type specific; Fig EV2A and B).

J The data from panel I were used to selectively display TF genes from cluster G.

genes (Fig 2F). Moreover, H3K4me3 labelling at Hep-ID$^{CONNECT}$ TF genes displayed intermediate tissue-specificity when compared to Hep-ID TF genes, on the one hand, and remaining TF-encoding genes from cluster G, on the other hand (Appendix Fig S7). Altogether, these data point to the potential importance of Hep-ID$^{CONNECT}$ TFs in fully differentiated hepatocytes.

Indeed, many Hep-ID$^{CONNECT}$ TFs have individually, that is, without an identified unifying rationale, been ascribed, at least to some extent, a role in the control of hepatic metabolic activities (Appendix Fig S8). In line, mining genes associated with phenotypes from the Mammalian Phenotype Ontology resource (Smith *et al*, 2005; Chen *et al*, 2007) revealed that, albeit less significant when compared to Hep-ID TFs, Hep-ID$^{CONNECT}$ TF-encoding genes are linked to abnormal liver phenotypes and metabolic functions (Fig 2G). Mining transcriptomic data obtained from the livers of mice with hepatocyte-specific deletion of individual Hep-ID$^{CONNECT}$ TF-encoding genes (hep$^{-/-}$ mice) indicated that this is functionally underlain by a preferential dysregulation of downstream hepatocyte identity effector genes, that is, non-TF genes with liver-specific broad H3K4me3 domains defined in Dubois *et al* (2020a) (Fig 2H). While such a bias was also observed in transcriptomic data obtained from the livers of mice deficient for individual Hep-ID TF genes, deletion of the general chromatin organizer *Ctcf* did not give rise to preferential dysregulation of hepatocyte identity effector genes (Fig 2H). In line, similar to Hep-ID TFs and different from CTCF, binding of most Hep-ID$^{CONNECT}$ TFs was stronger at hepatocyte identity effector gene promoters (Fig 2I). We found that preferential binding remained observable when using a more stringent control, that is, promoters with comparable accessibility and activity (activity-matched control promoters; see Materials and Methods and Appendix Fig S9A and B for further details). This ruled out that chromatin accessibility alone explains preferential binding to Hep-ID TF gene promoters (Fig 2I). Finally, binding of Hep-ID and Hep-ID$^{CONNECT}$ TFs correlated at the promoter of these genes pointing to combinatorial transcriptional regulation (Fig 2J).

Altogether, these data identified that Hep-ID TFs of the hepatic CoRC target Hep-ID$^{CONNECT}$ TF genes, and that these two sets of TFs further collaborate to control identity effector genes.

## Hep-ID$^{CONNECT}$ TFs are reciprocally targeting Hep-ID TF-encoding genes to finely tune their expression in homeostatic conditions

We hypothesized that the intimate connection between Hep-ID and Hep-ID$^{CONNECT}$ TFs identified so far could further extend to reciprocal transcriptional regulation where Hep-ID$^{CONNECT}$ TFs would bind and control expression of Hep-ID TF-encoding genes. Mining Hep-ID$^{CONNECT}$ TFs binding in mouse liver indicated greater recruitment to Hep-ID TF gene promoters when compared to control activity-matched TF-encoding gene promoters, reminiscent of data obtained for Hep-ID TFs (Fig 3A and Appendix Fig S10A–C). However, this only translated into moderate transcriptional changes in expression of Hep-ID TF-encoding genes. Indeed, only the deletion of a subset of the Hep-ID TF-encoding genes, and not that of Hep-ID$^{CONNECT}$ TF genes, individually triggers global down-regulation of the Hep-ID TF gene expression (Fig 3B). This most probably relates to the intrinsic role of identity TF networks, which are built-up to allow robustness of individual cell-type transcriptional programs (Kyrmizi *et al*, 2006; Almeida *et al*, 2021).

Several Hep-ID$^{CONNECT}$ TFs are central to establishment of rhythmic hepatocyte gene expression (Appendix Fig S11) including both circadian (BHLHE40, CLOCK, DBP, NFIL3, NR1D1/2; Mukherji *et al*, 2019) and ultradian (XBP1; Meng *et al*, 2020; Pan *et al*, 2020) rhythms. Hence, rather than being instrumental for regulating steady-state expression levels of Hep-ID TF genes, we envisioned that Hep-ID$^{CONNECT}$ TFs might be involved in proper rhythmic expression of Hep-ID TF-encoding genes. In line, mining data from

 © 2023 The Authors

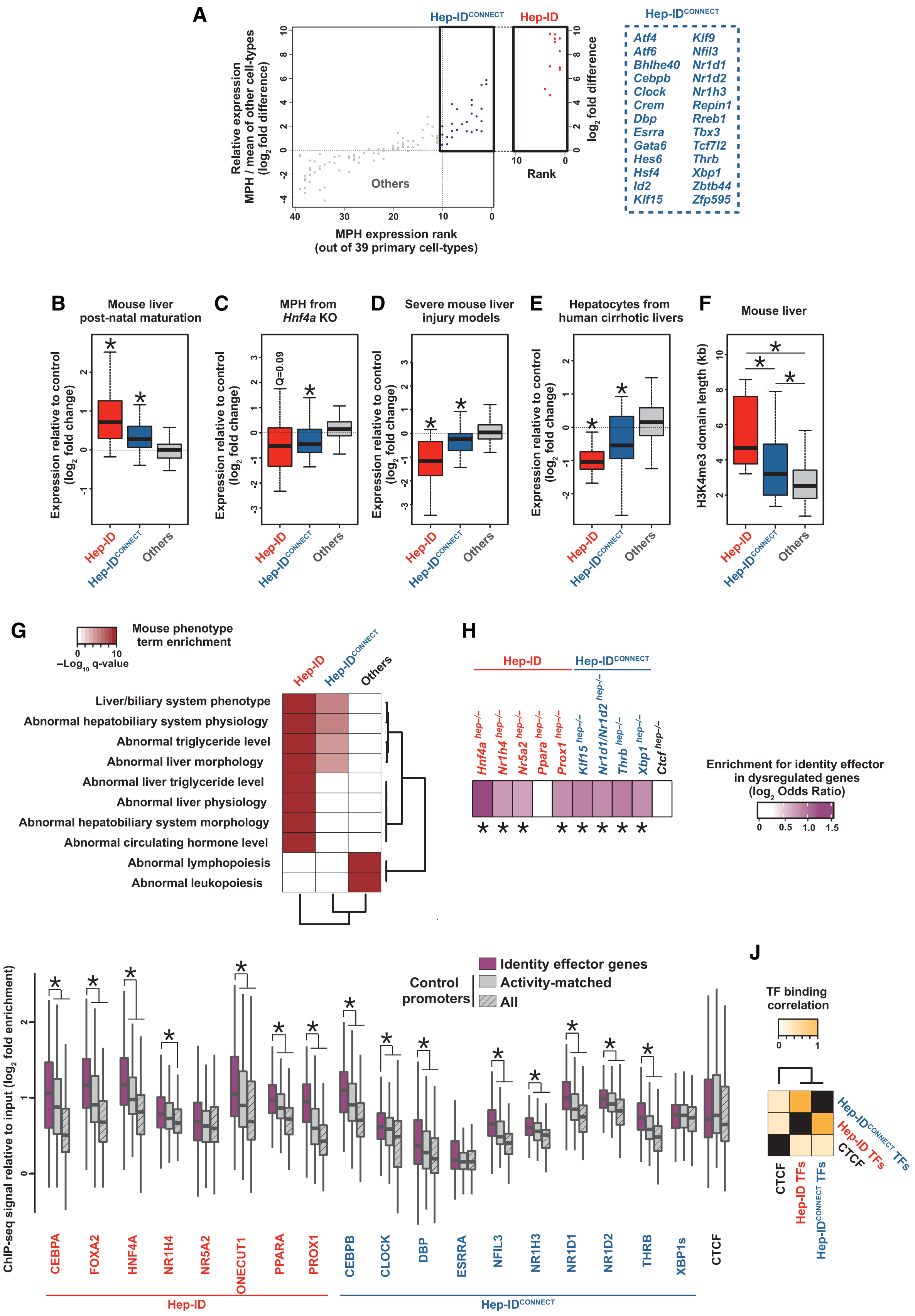
A
Hep-ID CONNECT
Hep-ID
Relative expression MPH / mean of other cell-types (log2 fold difference)
log2 fold difference
Others
Rank
MPH expression rank (out of 39 primary cell-types)
Atf4 Klf9
Atf6 Nfil3
Bhlhe40 Nr1d1
Cebpb Nr1d2
Clock Nr1h3
Crem Repin1
Dbp Rreb1
Esrra Tbx3
Gata6 Tcf7l2
Hes6 Thrb
Hsf4 Xbp1
Id2 Zbtb44
Klf15 Zfp595
B Mouse liver post-natal maturation
C MPH from Hnf4a KO
Q=0.09
D Severe mouse liver injury models
E Hepatocytes from human cirrhotic livers
F Mouse liver
Expression relative to control (log2 fold change)
H3K4me3 domain length (kb)
Hep-ID
Hep-ID CONNECT
Others
G
Mouse phenotype term enrichment
–Log10 q-value
Liver/biliary system phenotype
Abnormal hepatobiliary system physiology
Abnormal triglyceride level
Abnormal liver morphology
Abnormal liver triglyceride level
Abnormal liver physiology
Abnormal hepatobiliary system morphology
Abnormal circulating hormone level
Abnormal lymphopoiesis
Abnormal leukopoiesis
H
Hnf4a hep−/−
Nr1h4 hep−/−
Nr5a2 hep−/−
Ppara hep−/−
Prox1 hep−/−
Klf15 hep−/−
Nr1d1/Nr1d2 hep−/−
Thrb hep−/−
Xbp1 hep−/−
Ctcf hep−/−
Enrichment for identity effector in dysregulated genes (log2 Odds Ratio)
I
ChIP-seq signal relative to input (log2 fold enrichment)
Identity effector genes
Control promoters
Activity-matched
All
CEBPA FOXA2 HNF4A NR1H4 NR5A2 ONECUT1 PPARA PROX1 CEBPB CLOCK DBP ESRRA NFIL3 NR1H3 NR1D1 NR1D2 THRB XBP1s CTCF
J
TF binding correlation
Hep-ID CONNECT TFs
Hep-ID TFs
CTCF

Figure 2.

**Figure 2. Identification and characterization of Hep-ID$^{CONNECT}$ TFs.**

A The CTE/UBQ TFs from cluster G (Fig 1K) were plotted based on their expression in mouse primary hepatocytes (MPH) when compared to other primary mouse cell-types ($n$ = 38). For each individual TF, cells were first ranked according to decreasing gene expression and the rank of MPH was plotted on the $x$ axis (i.e., rank 1 indicates highest expression is in MPH). Second, expression in MPH was divided by the average expression in all other primary cells and plotted on the $y$ axis as $\log_2$ fold difference. Hep-ID$^{CONNECT}$ TF-encoding genes were defined as those preferentially expressed in MPH (rank ≤ 10 and FC > 0). For comparison, Hep-ID TF genes were plotted in an additional box on the right of the one highlighting Hep-ID$^{CONNECT}$ TFs.

B–E Expression of Hep-ID ($n$ = 13 genes), Hep-ID$^{CONNECT}$ ($n$ = 26 genes) and remaining TF-encoding genes from cluster G (Others; $n$ = 82 genes) was monitored in indicated transcriptomic data (Dataset EV2). Box plots show $\log_2$ fold changes between adult *versus* newborn mouse livers (B), MPH of *Hnf4a*$^{hep-/-}$ (*Hnf4a* KO) *versus* wild-type mice (C), a meta-analysis of severe mouse liver injuries *versus* control livers (D; see Materials and Methods and Appendix Fig S6) and microdissected hepatocytes from alcohol-related human liver cirrhosis (alcoholic steatohepatitis) *versus* control livers (E). Box plots are composed of a box from the 25$^{th}$ to the 75$^{th}$ percentile with the median as a line. Whiskers extent to the most extreme data point which is no more than 1.5 times the interquartile range from the box. Statistical significance was assessed using one-sided Wilcoxon rank sum test with Benjamini–Hochberg correction for multiple testing to determine if the mean $\log_2$ FC was statistically lower (B, D, E) or higher (C) than 0. *q < 0.05.

F Distribution of H3K4me3 domain length at the TSS of Hep-ID ($n$ = 13 genes), Hep-ID$^{CONNECT}$ ($n$ = 26 genes) and remaining TF-encoding genes from cluster G (Others; $n$ = 82 genes) as defined through broad peak calling on mouse liver H3K4me3 ChIP-seq data. Box plots are composed of a box from the 25$^{th}$ to the 75$^{th}$ percentile with the median as a line. Whiskers extent to the most extreme data point which is no more than 1.5 times the interquartile range from the box. Statistical difference between groups was defined using Kruskal–Wallis with Wilcoxon pairwise comparison tests followed by Benjamini–Hochberg correction for multiple testing correction. *q < 0.05.

G Mouse phenotypes associated with Hep-ID, Hep-ID$^{CONNECT}$ and remaining TF-encoding genes from cluster G (Others) were defined using ToppCluster. Dendrograms of hierarchical clustering are shown. ToppCluster uses hypergeometric tests and Bonferroni correction.

H Enrichment for identity effector genes among the top 1,000 transcriptionally dysregulated genes in the MPH/livers of indicated genetically deficient mice. $\log_2$ odds ratios were computed to compare the proportion of dysregulated versus non-dysregulated genes within identity effector genes or control non-TF-encoding genes. Then a two-sided Fisher exact test was performed to assess if the proportion of dysregulated genes was significantly different within the identity and control gene groups with Benjamini–Hochberg correction. *q < 0.05.

I Binding of indicated Hep-ID and Hep-ID$^{CONNECT}$ TFs to the promoter of identity effector genes and a control group of non-TF-encoding genes of similar size ($n$ = 424) was monitored using mouse liver ChIP-seq data. A control group of non-TF-encoding genes ($n$ = 424) matched for their promoter mouse liver activity was also used (Appendix Fig S9A and B). The control groups used were selected for providing data representative of those obtained with 1,000 reiterations of this analysis (see Materials and Methods; Appendix Fig S9A and B). The distribution of ChIP-seq signals is shown using box plots composed of a box from the 25$^{th}$ to the 75$^{th}$ percentile with the median as a line. Whiskers extent to the most extreme data point which is no more than 1.5 times the interquartile range from the box. Pairwise one-sided Wilcoxon Rank Sum Tests with Benjamini–Hochberg correction was used to define whether the binding at identity effector genes *versus* control genes was significantly greater for each analyzed TF recruitment. *q < 0.05.

J Correlation between Hep-ID TFs, Hep-ID$^{CONNECT}$ TFs and CTCF recruitment, as judged through the mining of mouse liver ChIP-seq data, to identity effector genes. The dendrogram is issued from hierarchical clustering analysis.

Source data are available online for this figure.

Meng *et al* (2020) revealed that a majority of Hep-ID TF genes (9 out of 13), including for example *Mlxipl*, were characterized by XBP-1-dependent ultradian expression patterns (Fig 3C and D, and Table EV2).

### Activation of Hep-ID$^{CONNECT}$ TFs resets CoRC TF expression and hepatocyte identity in dedifferentiated hepatocytes

Recent studies have found that TF activities may only be fully revealed in non-basal conditions and that TFs may be important to (re-)establish rather than to maintain cell-specific transcriptomes (Hunter *et al*, 2020; Lo *et al*, 2022). Therefore, we envisioned that a role for Hep-ID$^{CONNECT}$ TFs in controlling the Hep-ID TF network might be revealed in a biological context where hepatocyte identity is challenged such as cancer. Mining transcriptomic data of human hepatocarcinoma (HCC) from the cancer genome atlas (TCGA; $n$ = 367) indicated a positive correlation between expression of Hep-ID$^{CONNECT}$ and Hep-ID TF-encoding genes (Fig 4A). Moreover, similar to Hep-ID TFs, high expression of Hep-ID$^{CONNECT}$ TF genes was linked to better overall survival (Fig 4B and Appendix Fig S12A) and to a trend towards lower cancer grades (Appendix Fig S12B). Expression of many individual Hep-ID$^{CONNECT}$ TFs was positively correlated with that of Hep-ID TF genes (Fig 4C) and binding of Hep-ID$^{CONNECT}$ TFs to Hep-ID TF genes was also observed in hepatic cancer cells (Fig 4D). Moreover, overexpression or activation of several Hep-ID$^{CONNECT}$ TFs, GATA-binding protein 6 (GATA6), krüppel-like factor 9 (KLF9), T-box transcription factor 3 (TBX3) and the nuclear receptors liver X receptor alpha (NR1H3 also known as LXR alpha) and thyroid hormone receptor beta (THRB), has been shown to suppress hepatocarcinogenesis (Fig 4C; Sun *et al*, 2014; Tan *et al*, 2019; Kowalik *et al*, 2020; Lin *et al*, 2020; Liang *et al*, 2021). Survival analyses indicated better overall survival of patients with HCC expressing high levels of *KLF9* and *THRB* (Appendix Fig S12C). Based on all this information, we hypothesized that the HCC suppressive effects of Hep-ID$^{CONNECT}$ TFs may be linked to a resetting of Hep-ID TF expression. Therefore, we leveraged transcriptomic analyses performed on HCC cell-lines treated with ligands activating NR1H3 (Huh7 cells treated with GW3965; Vazquez Salgado *et al*, 2022) or THRB (HepG2 cells treated with 3,3′,5-triiodo-L-thyronine (T3) or GC-1; Yuan *et al*, 2012). In line with our hypothesis, we found that treatment with GW3965, T3, or GC-1 induced a global increase in Hep-ID TF gene expression in HCC cell-lines (Fig 4E). This was linked to the ability of these ligands to interfere with HCC cell-line growth *in vitro* (Appendix Fig S12D). Induction of Hep-ID TF gene expression by T3 and GC-1 in HepG2 cells was independently reproduced as judged using RT-qPCR analyses, induction of *DIO1* being used as a positive control in these assays (Fig 4F and G). In addition, THRB activation also increased the ratio of HNF4A adult promoter P1- to embryonic promoter P2-derived isoforms (Fig 4H), a phenomenon known to promote hepatocyte differentiation and function (Dubois *et al*, 2020b). Western blot assays confirmed T3-induced changes in Hep-ID TF levels (Fig 4I). Similar data were obtained using a non-cancerous human immortalized hepatic cell-line (IHH) used as another model


© 2023 The Authors

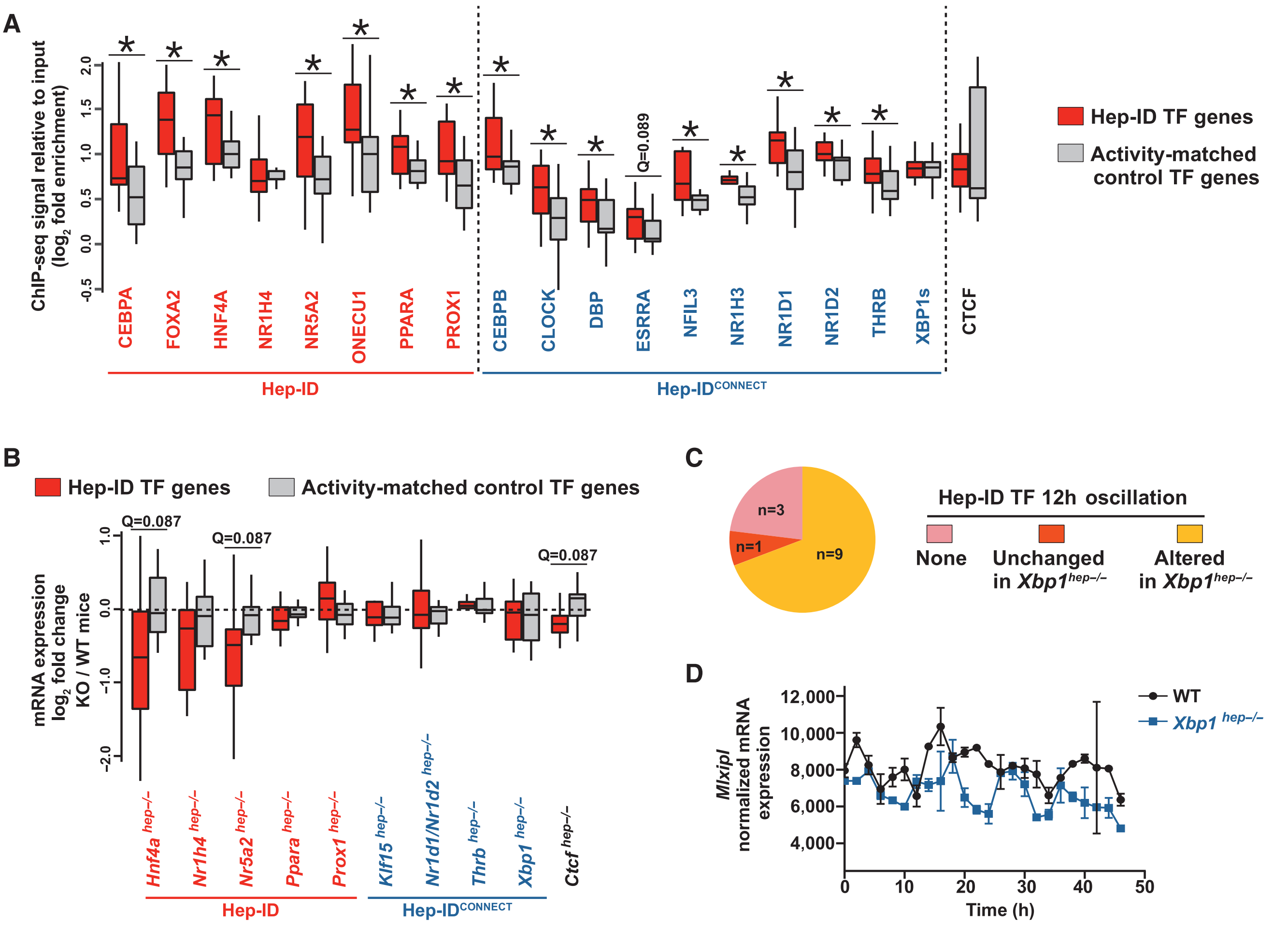


**Figure 3. Hep-ID$^{CONNECT}$ TF binding to and regulation of Hep-ID TF-encoding genes in basal conditions.**

A Binding of indicated Hep-ID and Hep-ID$^{CONNECT}$ TFs to the promoter of Hep-ID TF genes and a control group of non-Hep-ID TF-encoding genes matched for their promoter activity (Appendix Fig S10A) of similar size ($n = 13$) was monitored using mouse liver ChIP-seq data. The control group used was selected for providing data representative of those obtained with 1,000 reiterations of this analysis (see Materials and Methods; Appendix Fig S10A). The distribution of ChIP-seq signals is shown using box plots composed of a box from the 25$^{th}$ to the 75$^{th}$ percentile with the median as a line. Whiskers extent to the most extreme data point which is no more than 1.5 times the interquartile range from the box. One-sided Wilcoxon rank sum tests with Benjamini–Hochberg correction was used to define whether the binding on Hep-ID TF gene promoters was greater than on control genes for each individual TF ChIP-seq dataset. *$q < 0.05$.

B Transcriptional modulation of Hep-ID TFs and a control group of non-Hep-ID TF-encoding genes matched for their promoter activity (Appendix Fig S10A) of similar size ($n = 13$) in mouse liver/MPH of mice deleted for the indicated Hep-ID or Hep-ID$^{CONNECT}$ TF genes. The control group used was selected for providing data representative of those obtained with 1,000 reiterations of this analysis (see Materials and Methods; Appendix Fig S10A). The distribution of $\log_2$ fold changes is shown using box plots composed of a box from the 25$^{th}$ to the 75$^{th}$ percentile with the median as a line. Whiskers extent to the most extreme data point which is no more than 1.5 times the interquartile range from the box. One-sided Wilcoxon rank sum tests with Benjamini–Hochberg correction was used to define whether $\log_2$ fold changes for Hep-ID TF genes were lower than those of the control genes for each individual transcriptomic dataset. *$q < 0.05$.

C 12 h gene expression oscillation analyses in the mouse liver performed by Meng *et al* (2020) from WT and *XBP1*$^{hep-/-}$ animals were used to identify XBP1-dependent oscillating expression patterns for Hep-ID TF genes (Table EV2).

D Average gene expression levels of *Mlxipl* in the livers of WT and *XBP1*$^{hep-/-}$ mice across circadian time ($n = 2$ mice per group). Error bars show standard deviations.

Source data are available online for this figure.

of partially dedifferentiated hepatocytes (Schippers *et al*, 1997; Fig EV4A–C). Moreover, interrogating the transcriptomic data obtained in a rat model of hepatocarcinogenesis (Kowalik *et al*, 2020) revealed that T3 was able to counteract the cancer-driven loss of expression of Hep-ID TFs (Fig 4J). Indeed, T3 treatment led to robust transcriptional induction of Hep-ID TF-encoding genes whose expression was most strongly down-regulated in the cancerous nodules (Fig 4K). This was in contrast with T3 not triggering a global rise in Hep-ID TF expression in healthy mice (Appendix Fig S13; Singh *et al*, 2018) further substantiating a role for THRB in controlling Hep-ID TF genes only in context of dedifferentiated hepatocytes. T3-mediated re-expression of Hep-ID TFs further translated into re-expression of the most strongly down-regulated identity effector genes consistent with concomitant re-establishment of a transcriptional program closer to that of differentiated hepatocytes (Fig 4L).

Recent studies have revealed a role for T3 in promoting acquisition of a hepatocyte-like phenotype from pluripotent stem cells (Ma

© 2023 The Authors 

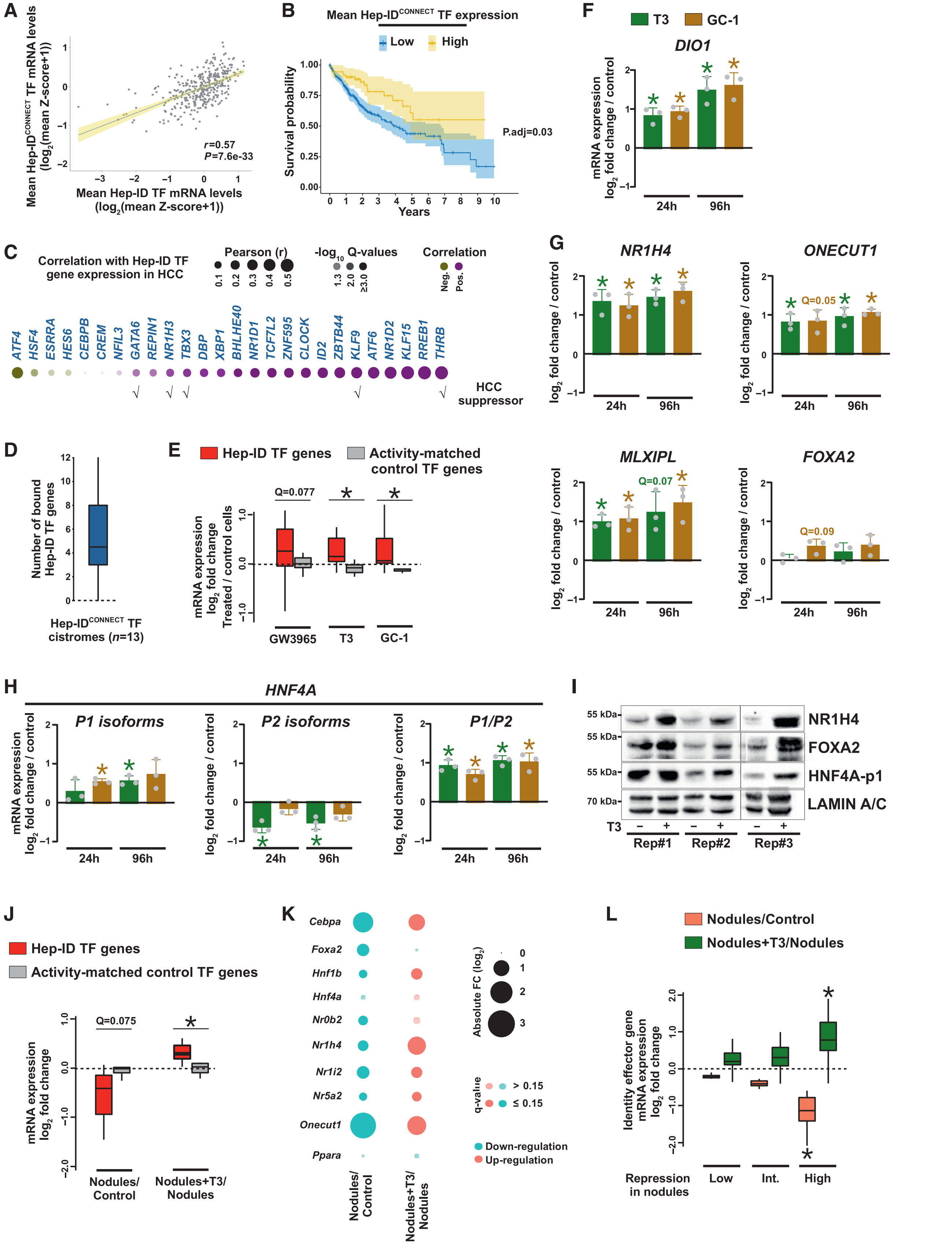


**Figure 4.**


© 2023 The Authors

◀

**Figure 4. Resetting Hep-ID TF gene expression through Hep-ID$^{CONNECT}$ TF activation in hepatocellular carcinoma.**

A Comparison of the average expression of Hep-ID$^{CONNECT}$ and Hep-ID TF genes in HCC ($n = 367$). The Spearman correlation coefficient $r$ and associated $P$-value is indicated.

B Overall survival of patients with HCC expressing low ($n = 289$) or high ($n = 73$) levels of the Hep-ID$^{CONNECT}$ TF-encoding genes. Differential overall survival analysis was assessed by Kaplan–Meier (KM) log rank adjusted for 100 permutations (Cheng *et al*, 2022).

C Analyses similar to that described in panel A using individual Hep-ID$^{CONNECT}$ TF genes. Hep-ID$^{CONNECT}$ TFs which have been ascribed with HCC suppressive functions in the literature are indicated at the bottom.

D Hep-ID$^{CONNECT}$ TF cistromes in HepG2 cells ($n = 13$ independent cistromes) were mined for binding to Hep-ID TF-encoding genes. Peaks localized ± 10 kilobases from transcriptional start sites were considered in these analyses.

E Transcriptional modulation of Hep-ID TFs ($n = 13$ genes) and a control group of non-Hep-ID TF-encoding genes matched for their promoter activity (Appendix Fig S10A) of similar size ($n = 13$ genes) in Huh7 or HepG2 cells treated with GW3965, T3 or GC-1, respectively. The control group used was selected for providing data representative of those obtained with 1,000 reiterations of this analysis (see Materials and Methods; Appendix Fig S10A). The distribution of $\log_2$ fold changes is shown using box plots. Kruskal–Wallis with two-sided Wilcoxon pairwise comparison tests followed by Benjamini–Hochberg correction was used to define whether transcriptional regulation of Hep-ID TF genes was different from that of the control genes for each individual transcriptomic dataset. *$q < 0.05$.

F–H mRNA expression of the indicated genes was monitored using RT-qPCR in HepG2 cells treated with T3 or GC-1 for 24 h or 96 h. Bar graphs show mean ± SD ($n = 3$ biological replicates) of $\log_2$ fold changes in treated *versus* untreated HepG2 cells. For *Hnf4a*, the $\log_2$ fold change in the ratio of P1 over P2 promoter-derived isoforms is also shown. Gray dots show the results obtained from the three independent biological replicates (each performed in technical triplicates). One-sample $t$-test with Benjamini–Hochberg correction for multiple testing was used to determine if the mean log2 FC was statistically different from 0. *$q < 0.05$.

I Western blots assays performed using antibodies against the indicated proteins on extracts from HepG2 cells treated or not with T3 for 24 h. Rep#1–3 indicates the three independent biological replicates analyzed.

J Modulation of Hep-ID TF gene expression in precancerous nodules compared to control rat livers and in liver nodules of rats treated with T3 compared to nodules of non-treated rats. A control group of non-Hep-ID TF-encoding genes matched for their promoter activity (Appendix Fig S10A) of similar size ($n = 13$) is also shown. The control group used was selected for providing data representative of those obtained with 1,000 reiterations of this analysis (see Materials and Methods; Appendix Fig S10A). Box plots show the $\log_2$ fold changes. Two-sided Wilcoxon rank sum tests with Benjamini–Hochberg correction was used to define whether transcriptional regulation of Hep-ID TF genes was different from that of the control genes for each individual transcriptomic dataset. *$q < 0.05$.

K Dot plots showing the transcriptional regulation of individual Hep-ID TF gene expression in precancerous nodules compared to control rat livers (left; Nodules/Control) and in liver nodules of rats treated with T3 compared to nodules of non-treated rats (right; Nodules + T3/Nodules). No data were recovered for *Onecut2*, *Prox1* and *Mlxipl*.

L Identity effector genes significantly downregulated in Nodules/Control ($q < 0.05$) were split in three groups according to their $\log_2$ fold changes (i.e., low, intermediate, and high repression; pink boxes) and then monitored for induction in Nodules + T3/Nodules (green boxes). Statistical differences between the High repression and the other groups regarding the Nodules/Control comparison, on the one hand, or the Nodules + T3/Nodules comparison, on the other hand, were defined using Kruskal–Wallis with two-sided Wilcoxon pairwise comparison tests followed by Benjamini–Hochberg correction for multiple testing correction. *$q < 0.05$.

Data information: Box plots in panels D, E, and J are composed of a box from the 25$^{th}$ to the 75$^{th}$ percentile with the median as a line. Whiskers extent to the most extreme data point which is no more than 1.5 times the interquartile range from the box.
Source data are available online for this figure.

*et al*, 2022), defining that T3-mediated effects towards promoting a mature hepatocyte cell-state are not limited to cancer cells. We could not however observe any protective role for T3 treatment with regards to the loss of Hep-ID TF gene expression spontaneously occurring when primary mouse hepatocytes (MPH) are cultured *in vitro* (Ploton *et al*, 2018; Seirup *et al*, 2022; Appendix Fig S14). This is most probably linked to the immediate and simultaneous drastic changes occurring in MPH, including at the chromatin structure level, which characterize this dedifferentiation model and make it inherently difficult to intervene with (Seirup *et al*, 2022). Indeed, previous reports indicated that MPH dedifferentiation could only be partially inhibited when using combined inhibition of multiple dedifferentiating signaling pathways (Sun *et al*, 2019; Xiang *et al*, 2019) or through forced expression of supra-physiologic amounts of the Hep-ID TF HNF4A (Nishikawa *et al*, 2015; Tafaleng *et al*, 2021; Yang *et al*, 2021). Another complementary explanation may come from the rapid decrease in *Thrb* expression which accompanies MPH dedifferentiation (Appendix Fig S14). Therefore, we next aimed to evaluate if T3 could modulate hepatocyte dedifferentiation in a non-cancerous setting *in vivo*.

As stated earlier, we and others have reported that hepatocyte partial loss of identity is a hallmark of diseased livers [recently reviewed in (Berasain *et al*, 2022)]. Indeed, inflammatory cytokines such as tumor necrosis factor or IL1B are well-established common drivers of hepatocyte dedifferentiation and dysfunction (Del Campo *et al*, 2018; Hyun *et al*, 2020). In this context, we set-up an experimental model where mice were injected with interleukin 1 beta (IL1B) for 3 h before livers were collected. IL1B treatment indeed triggered loss of hepatocyte identity including decreased expression of several Hep-ID TF genes as assessed through transcriptomic analyses (Fig EV5A and B). We next determined whether T3 treatment promoted the recovery of Hep-ID TF gene expression in this model, that is, mice were first injected with IL1B and 3 h later with or without T3 for an additional 3 h (Fig 5A). This acute setting allowed us to investigate the effect of T3 independent of its previously described pro-regenerative activities through modulation of hepatocyte proliferation (Tang *et al*, 2022). Inbred laboratory mice raised in standardized conditions nevertheless keep displaying high phenotypic trait variability (Gartner, 1990; Tuttle *et al*, 2018) and are prone to polyphenism, that is, several discrete phenotypes on the same genetic background (Dalgaard *et al*, 2016; Yang *et al*, 2022). Here, mice were subdivided into three groups (low, intermediate and high responsiveness groups) based on their response to T3 as judged using stimulation of the *Dio1* and *Hectd3* gene expression, which are previously identified THRB hepatic target genes (Fig 5B; Paquette *et al*, 2011). Importantly, these differences were due to variability in the T3 response only, since IL1B-mediated induction of pro-inflammatory genes was similar between the three groups and no negative correlation between T3 and IL1B responses was detected (Figs 5C and EV5C). We also ruled out any major difference in immune cell infiltration as a potential confounder in these analyses (Fig EV5D). Interestingly, the IL1B + T3

© 2023 The Authors 

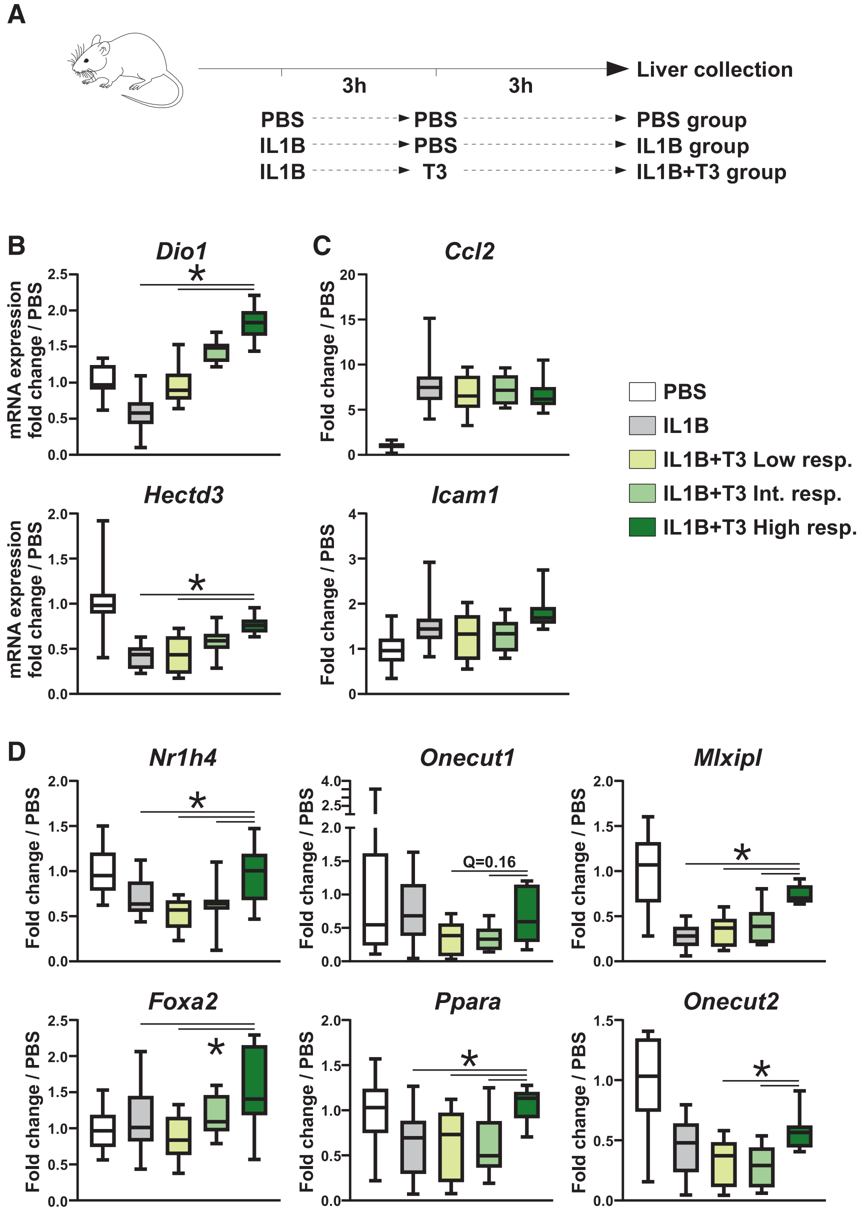


**Figure 5. Resetting Hep-ID TF gene expression through THRB activation in inflammation-induced hepatocyte dedifferentiation.**

A Experimental protocol for acute inflammation-induced loss of hepatocyte identity *in vivo*. Mice were injected with IL1B (IL1B; $n = 17$), IL1B followed by T3 (IL1B + T3; $n = 30$) or a control group (PBS; $n = 14$). All livers were collected 6 h after the initial injection.

B–D mRNA expression of the indicated genes was monitored in mouse livers using RT-qPCR. Mice treated with IL1B + T3 were subdivided into tertiles based on the mean expression of the *Dio1* and *Hectd3* genes and defined as low, intermediate or high T3 responsiveness groups (Low resp., Int. resp. and High resp., respectively). Fold change relative to the mean of the control group is shown using box plots composed of a box from the $25^{th}$ to the $75^{th}$ percentile with the median as a line ($n = 13$ mice for the PBS group, 17 for the IL1B group and 10 for the other groups). Whiskers extend to the maximum and minimum values. Statistical differences between the High resp. and the other IL1B-treated groups were defined using Kruskal–Wallis with two-sided Wilcoxon pairwise comparison tests followed by Benjamini–Hochberg correction for multiple testing correction. *$q < 0.05$.

Source data are available online for this figure.

high responsiveness group showed expression recovery of Hep-ID TF genes whose levels were higher in this group when compared to those in the IL1B only and/or IL1B + T3 low responsiveness groups (Fig 5D).

Altogether, these data have identified the Hep-ID$^{\mathrm{CONNECT}}$ TF THRB as an actionable target to reset expression of hepatic identity TF genes in partially dedifferentiated hepatocytes.

## Discussion

Our promoter-centric and multi-omics approach called ProTFnet allowed us to unveil the complexity of the TF network controlling hepatocyte identity and functions. Using this approach, we identified that Hep-ID TFs of the CoRC are tightly connected with an additional layer of TFs through reciprocal co-recruitment to their

 © 2023 The Authors

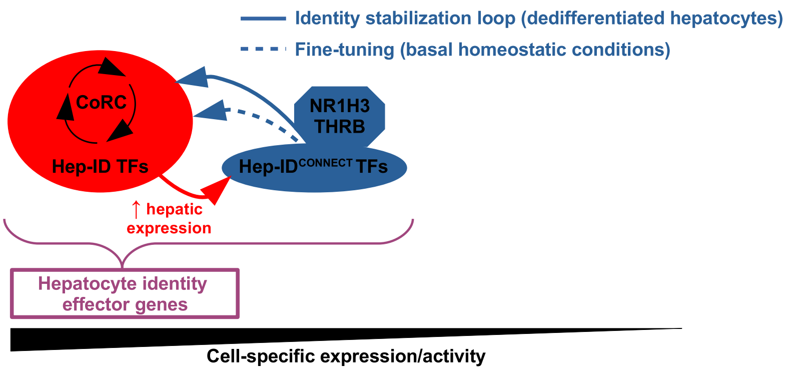


**Figure 6. Proposed model for the control of hepatocyte identity by an extended transcription factor network.**
Schematic summarizing the main findings of our study pointing to an extended hepatic TF identity network which includes THRB. See Discussion for greater details.

promoters. The CoRC, in addition to being instrumental for cell identity establishment and maintenance, comprises TFs which are also key for controlling and adapting mature cell functions to homeostatic requirements. In this context, the tight connection between Hep-ID$^{CONNECT}$ TFs and the CoRC provides an additional layer of regulation to finely tune Hep-ID TF expression. For instance, Hep-ID$^{CONNECT}$ TFs comprise regulators of rhythmic gene expression including XBP1. Other Hep-ID$^{CONNECT}$ TFs, TBX3 and TCF7L2, have been identified as regulators of zonated hepatocyte transcriptional programs (preprint: González-Blas *et al*, 2022) pointing to a role of Hep-ID$^{CONNECT}$ TFs in specifying proper hepatic gene expression in both space and time. Importantly, rhythmic and zonated hepatic gene expression are crucial to maintain appropriate liver metabolic and non-metabolic activities (Mukherji *et al*, 2019; Meng *et al*, 2020; Pan *et al*, 2020; Paris & Henderson, 2022).

While not being required to sustain Hep-ID TF gene expression and cell identity in basal condition, our data obtained with NR1H3 and THRB in dedifferentiated hepatocytes revealed that Hep-ID$^{CONNECT}$ TF activation can be leveraged to reset the CoRC and hepatocyte identity. These findings are consistent with other recent observations where TF binding to cell-specific target genes translates into a more critical role in (re)establishment and dynamic regulation rather than maintenance of steady-state gene expression levels (Lo *et al*, 2022). Our study is also consistent with recent insights into the understanding of cell fate choice, which have suggested a more distributed form of control relying on combinatorial activities of dozens of TFs rather than an organization strictly dominated by a few master TFs (Chubb *et al*, 2021; Mittnenzweig *et al*, 2021). In this context, we propose that Hep-ID$^{CONNECT}$ TFs are involved in identity stabilization loops, whose functional importance with regards to cell identity is revealed in pathophysiological situations of dedifferentiation (Fig 6). Context-dependent activities are an intrinsic property of TFs and precisely defining how Hep-ID$^{CONNECT}$ TFs sense the cellular state to adapt their functions will require additional studies.

Loss of hepatocyte identity is a main feature of tumorigenesis, but is now ascribed a broader pathophysiological relevance including loss of activity of severely injured livers (Berasain *et al*, 2022). In both situations, resetting hepatocyte identity through re-expression of CoRC TFs is considered of potential therapeutic interest (Chao *et al*, 2020; Berasain *et al*, 2022). By identifying the Hep-ID$^{CONNECT}$ TF-controlled identity stabilization loops, our study has identified new avenues to achieve this goal. In particular, our data with agonists of the nuclear receptors NR1H3 and THRB are particularly relevant as several of those are being considered for treatment of liver diseases (Russo-Savage & Schulman, 2021; Hatziagelaki *et al*, 2022). With regard to HCC, our data provide explanation for the reported beneficial effects exerted by NR1H3 and THRB. Many Hep-ID$^{CONNECT}$ TFs, including NR1H3 and THRB, do not display typically used features to define hepatocyte identity TFs such as hepatocyte-specific expression and association with super-enhancers (Hnisz *et al*, 2013; Dubois *et al*, 2020a; Table EV3). Hence, our study has allowed to uncover an unforeseen role for an extended transcriptional regulatory network beyond the CoRC in the control of hepatocyte identity (Fig 6).

## Materials and Methods

### Data retrieval

Public functional genomics data used in this study were downloaded from the Gene Expression Omnibus (GEO; Barrett *et al*, 2013), ArrayExpress (Sarkans *et al*, 2021), ENCODE (Yue *et al* 2014), the UCSC Genome Browser (Raney *et al* 2011), FANTOM5 (Lizio *et al*, 2015), or from BioGPS (Wu *et al* 2009) and are listed in Dataset EV2.

Hep-ID TFs were defined as hepatocyte-identity TFs retrieved in at least two out of three independent studies (D'Alessio Ana *et al*, 2015; Zhou *et al*, 2017; Dubois *et al*, 2020a). The top 20 TFs were used for (D'Alessio Ana *et al*, 2015).

Mouse liver transcriptionally active promoters, that is, giving rise to detectable gene transcription, were defined by Wang and colleagues based on Precision nuclear Run-On Sequencing (PRO-Seq) data (Wang *et al*, 2018). HepG2 active promoters were defined as the transcriptional start sites (TSSs) from Gencode v32 (Frankish *et al*, 2018) with H3K27ac ChIP-seq signal (ENCODE, Dataset EV2) with at least twofold enrichment over control within a 1 kilobase (± 500 bp of TSS) window. Only the most active TSS per gene, that is, highest H3K27ac ChIP-seq signal, were kept.

The IDR thresholded peaks issued from ChIP-seq analyses of flag-tagged Hep-ID$^{CONNECT}$ TFs ($n = 14$) in HepG2 cells were obtained from ENCODE (Dataset EV2).

Tau indexes of tissue-specific expression were retrieved from (Kryuchkova-Mostacci & Robinson-Rechavi, 2017).

### Self-organizing maps (SOM) analyses

Active promoters in the mouse liver were defined as *cis*-regulatory modules, that is, genomic regions bound by at least two different transcriptional regulators (Appendix Fig S1A and B) as in (Dubois-Chevalier *et al*, 2017a), which overlap active TSSs identified as PRO-seq data summits in (Wang *et al*, 2018). Non-unique associations between TSSs and genes in the PRO-seq data were discarded. Only transcription factor (TF) encoding genes, retrieved using a manually curated gene list originally obtained from the AnimalTFDB2.0 database (Zhang *et al*, 2015), were considered. Transcriptional regulator co-recruitment analyses made use of mouse liver ChIP-seq data for 49 factors (Dataset EV2), which were uniformly processed including quality control checks as described in Dubois-Chevalier *et al* (2017a). All these data were obtained using the liver of untreated adult mice. In line with the vast majority of the liver chromatin stemming from hepatocytes (which are the prominent cell-type in the liver and are, moreover, mostly polyploid cells – up to 85% in mice with mainly tetraploid hepatocytes; Duncan *et al*, 2010), hepatic TF cistromes can be faithfully inferred from mouse liver ChIP-seq data (Kyrmizi *et al*, 2006; Schmidt *et al*, 2010; Dubois-Chevalier *et al*, 2017a; Sommars *et al*, 2019; Dubois *et al*, 2020a).

The SOM were generated using the R package kohonen2 (Wehrens & Buydens, 2007) as described in Dubois-Chevalier *et al* (2017a). Cells containing promoters with similar transcriptional regulator-binding patterns (Appendix Fig S1C and D) were further grouped into clusters (denoted A–G) based on hierarchical clustering performed using the hclust function of the R package Stats (R Core Team, 2015). We used the Ward agglomeration method and the best representative transcriptional regulator combination (prototype) for each individual cell. The number of clusters was chosen according to homogeneity analyses (http://lastresortsoftware.blogspot.fr/2010/08/homogeneity-analysis-of-hierarchical.html; Bedward *et al*, 1992) and biological significance. A planar projection of the toroidal map was used for data visualization.

### Analyses of transcriptional regulator co-recruitment patterns in SOM-derived clusters

Transcriptional regulator co-occurrence in clusters A–G was used to calculate Tanimoto distance matrices, which were used to draw heatmaps and perform hierarchical clustering or perform multidimensional scaling (MDS) analyses using R (R Core Team, 2015) as described in Dubois-Chevalier *et al* (2017a). To analyze the combinations of transcriptional regulators bound at the different promoters of a given cluster, a "frequent itemsets" search (combinations of 2–49 regulators) was performed using the arules R package (Hahsler *et al*, 2005). Only itemsets occurring in at least 50% of promoters of a given cluster were considered and defined as the core co-recruitment nodes. Finally, the percentage of occurrence of each transcriptional regulator in these nodes was retrieved.

### Transcriptomic data analyses

#### *Processing of raw data and differential gene expression analyses*

Raw transcriptomic data from Affymetrix microarrays were normalized on a local instance of Galaxy (Afgan *et al*, 2018) using the GIANT APTtool (Affymetrix Power Tools; www.thermofisher.com/fr/fr/home/life-science/microarray-analysis/microarray-analysis-partners-programs/affymetrix-developers-network/affymetrix-power-tools.html; with options: gc correction when available, scale intensity and rma at probeset level) from the GIANT toolbox (Vandel *et al*, 2020). Normalized expressions were averaged per Gene Symbol (NetAffx Annotation Release 36, July 2016). Raw transcriptomic data from Agilent microarrays were processed with the limma R package (Ritchie *et al*, 2015) used to normalize the data through the "backgroundCorrect" function (parameters used were method: "normexp" and normexp.method: "rma") and to filter out low expressed probes. Normalized data from Illumina Bead Chips were retrieved from the Gene Expression Omnibus database and annotated using GPL6101_Illumina_RatRef-12_V1_0_R1_11222119. Orthologous mouse genes were retrieved using Ensembl annotations (release 105; Howe *et al*, 2021). Differential expression analyses were performed with GIANT using the limma tool (FDR cutoff set at 0.15).

RNA-seq data were analyzed using the Galaxy web platform (Afgan *et al*, 2018). Mapping of reads on mm10 was performed with HISAT2 (version 2.21; options: default; Kim *et al*, 2019). Mapped reads mapping to exons were subsequently retrieved and merged by gene_id with Htseq-count (version 0.9.1; Anders *et al*, 2015) using the mm10 annotation of Ensembl (release 102; Yates *et al*, 2020; with the following options, mode: union, minimum alignment quality: 10, "do not count non uniquely or ambiguously mapped reads," stranded: no). Normalization and differential analyses were performed with EdgeR (version 3.36.0; Robinson *et al*, 2010; Liu *et al*, 2015; options: lowly expressed genes filtered out, cutoff: < 1 CPM in $n$ samples—with $n$ corresponding to number of biological replicates in one condition, FDR < 0.15, normalization method: TMM, Robust settings: True). Normalized gene expressions were obtained by averaging data per Gene Symbol using the Ensembl annotation (release 105; Howe *et al*, 2021). Dot plots displaying dysregulation in transcriptomic data were performed with the ggplot2 R package (Wickham, 2016).

#### *Analysis of mouse liver TF-encoding gene expression*

Normalized read counts in gene bodies obtained using PRO-Seq (Wang *et al*, 2018) were used to define TF gene expression in the mouse liver. Gene expression liver specificity was computed using data from the BioGPS Mouse MOE430 Gene Atlas (Wu *et al*, 2016) by dividing the normalized expression in the liver by the average normalized expression in other tissues (Dataset EV2). In both instances, TF-encoding genes were finally separated into deciles of increasing mouse liver (specific) expression.

#### *Mining of CAGE-seq data to define CTS (cell-type specific), CTE (cell-type enriched), and UBQ (ubiquitous) TFs*

CAGE-seq data from FANTOM5 (Lizio *et al*, 2015) were used to define CTS, CTE and UBQ TF-encoding genes based on their patterns of expression in primary human and mouse cells. Human and mouse orthologous genes were defined using the Ensembl annotation (version 105; Howe *et al*, 2021). Only TF-encoding

 © 2023 The Authors

genes retrieved in both the human and mouse CAGE-seq data ($n$ = 1,009) were considered. Their expression levels in mouse and human cells were stacked in a unique matrix centered and scaled, which was used to perform a Multiple Factor Analysis (MFA) using the MFA function of the R package FactoMineR (Lê *et al*, 2008) considering expression in mouse and human cells as two distinct groups of variables and TFs as individuals (parameters were set as follows, type: c('s', 's'), ncp: 5). The two first components of this MFA accounted for approximately 84% of the variability of the two combined datasets. A hierarchical clustering was performed on these five first components using the HCPC function of the FactoMineR package (parameters were set as follows, nbclust: 3, metric:"euclidean", method: "ward"). This identified three clusters of TFs characterized by an increasing number of cell-types displaying high expression from CTS to UBQ TFs (Fig EV2A and B).

### *Mining of transcriptomic data to monitor hepatic dedifferentiation*

To monitor hepatocyte dedifferentiation in mouse liver injury models, all liver injury transcriptomic data were batch corrected against the liver development transcriptomic data used as a reference. This was performed with the ComBat function of the R package SVA (parameters were set as follows, mean.only: T, par.prior: T, control samples for the batch correction were the adult livers for the differentiation study and the livers from wild-type or untreated mice for the injury studies; Leek *et al*, 2022). Then a principal component analysis (PCA) was computed on the liver differentiation study using the R package FactoMineR (scale.unit set to F; Lê *et al*, 2008). Since the first component, representing 64% of the dataset variability, allowed to separate the different stages of liver differentiation, it was used to project the liver injury studies. Fold changes between injured and control livers were computed on the batch corrected data and the median $\log_2$ fold change for individual genes across the different studies was recovered and plotted.

### *Mining transcriptional changes in identity genes versus control gene sets*

Transcriptional modulations of identity effector and TF-encoding genes were compared to those in control genes matched for mouse liver promoter activity. A hierarchical clustering was performed based on promoter DHS-seq and H3K27ac ChIP-seq signals together with mouse liver gene expression levels considering all active non-TF genes (Appendix Fig S9A) or only TF-encoding genes (Appendix Fig S10A). Individual signals were scaled and clustering was performed using $\log_2$-transformed data. This allowed to define four clusters of promoters with different activity levels among both non-TF and TF-encoding genes. Groups of control genes were defined by randomly selecting an equivalent number of non-identity genes matching the distribution of identity effector or TF genes in these clusters. This was performed 1,000 times (without replacement) to compare the identity genes to the individual control gene sets (Appendix Figs S9A and S10A). Results of Wilcoxon rank sum tests were recorded and the mode of the $P$-value distribution was used to select a representative control gene set among the 1,000 subsamples. Selected representative control groups were in main figures.

### *Gene expression correlation in human HCC and overall survival analyses*

The web portal cSurvival v1.0.1 was used (https://tau.cmmt.ubc.ca/cSurvival/; Cheng *et al*, 2022) to retrieved the spearman correlation between the average normalized mRNA expression of Hep-ID$^{CONNECT}$ TF and Hep-ID TF genes taken as gene sets in "Liver Hepatocellular Carcinoma (LIHC from TCGA)" ($n$ = 367 curated samples). Complementary analyses were similarly performed using individual Hep-ID$^{CONNECT}$ TF-encoding genes. Overall survival data were retrieved in parallel from cSurvival, which uses automated stratification between low and high expressing groups from an optimal cut-off defined from the minimum $P$-value (Cheng *et al*, 2022). To mine associations of low versus high expressing HCC with additional clinical parameters, patients ID were retrieved from the cSurvival analyses and used in the cBioPortal (https://www.cbioportal.org/; Gao *et al*, 2013).

### *Mining of single-nuclei RNA-seq data*

Gene expression counts obtained for healthy adult mouse and human livers were downloaded from https://www.livercellatlas.org/ (Guilliams *et al*, 2022). Single-nuclei RNA-seq data were selected by keeping only data labeled as "nucSeq" for their sample type. Counts were normalized using SCTransform from the Seurat v4.0.3 package (Hao *et al*, 2021). Cell types comprising less than 50 cells were removed. Data were finally loaded into ISCEBERG v1.0.0 (preprint: Guille *et al*, 2022) to generate violin plots of Hep-ID and Hep-ID$^{CONNECT}$ TF-encoding gene average expression.

## DHS-seq and histone ChIP-seq data analyses

Uniform reprocessing of the data including peak calling and genome-wide average signal has been described in Dubois-Chevalier *et al* (2017a). ChIP-seq signals were also alternatively used as enrichments over input, which were obtained as follows. First, input datasets from several mouse liver ChIP-seq studies (Dataset EV2) were merged into a "meta-input" file after removal of duplicated reads and false-positives regions identified by ENCODE (blacklisted regions v1; Amemiya *et al*, 2019) or defined as repeatedly enriched in inputs and IgG ChIP-seq in our previous study (Dubois-Chevalier *et al*, 2017a). Then, the Bam file for each TF ChIP-seq dataset was used to run MACS2 callpeak (−g = 1.89e9, −q = 0.05, −-keep-dup = all, −-scale-to = small, B) using the "meta-input" Bam file as control. Finally, the two BedGraph files issued from MACS2 callpeak were used in MACS2 bdgcmp (m = logFE) to obtain the genome-wide $\log_2$ fold enrichment track files over input for each TF (MACS2 version 2.2.7.1; Zhang *et al*, 2008). DHS-seq or ChIP-seq signal at a given promoter was defined as the maximum signal within TF binding sites (i.e., *cis*-regulatory modules as defined hereabove) encompassing this promoter retrieved using the extract bed function of bwtool version 1.0 (Pohl & Beato, 2014). Comparison of individual TF binding to identity and control gene promoters involved reiterative comparisons with independent matched control groups as described hereabove for transcriptomics analyses. Combinatorial binding of Hep-ID TFs, Hep-ID$^{CONNECT}$ and CTCF on effector gene promoters was compared using the distance correlation provided by the dcor function of the Rfast package in R (Papadakis *et al*, 2022).

Visualization of ChIP-seq data at selected genes was performed using the Integrated Genome Browser (IGB 9.1.4; Freese *et al*, 2016).

© 2023 The Authors 

H3K4me3 ENCODE ChIP-seq data from mouse tissues (Shen *et al*, 2012) were used to call H3K4me3-enriched regions using the broad peak calling option of MACS2 as described in Chen *et al* (2015). Genes were assigned to H3K4me3-enriched regions using the closestBed function (with parameters −t all, −k 1, −d, −mdb) of the BEDTools version 2.3.0 (Quinlan & Hall, 2010), that is, active gene TSS from the mouse liver PRO-Seq data (Wang *et al*, 2018) were assigned H3K4me3-enriched regions when distance was 0. Calling of super-enhancers (SE) and target gene assignation, essentially performed as in (Loven *et al*, 2013; Whyte *et al*, 2013), was previously described (Dubois-Chevalier *et al*, 2020).

#### Mouse phenotype ontology and literature mining

Enrichments within the mouse phenotype ontology or biological pathway data were defined using ToppCluster (Chen *et al*, 2007; Kaimal *et al*, 2010). Default parameters were used and only terms linked to > 5 TF-encoding genes were considered.

References to Hep-ID$^{CONNECT}$ TFs in the scientific literature related to liver/hepatocyte metabolic functions were retrieved using the easyPubMed package (https://www.data-pulse.com/dev_site/easypubmed/) in R. articles with co-occurrence of a given Hep-ID$^{CONNECT}$ TF gene name and ("hepatocyte" or "liver") and "metabolism" in their title or abstract were considered. The number of retrieved manuscripts for each Hep-ID$^{CONNECT}$ TF was visualized as a heatmap prepared using the heatmap.2 function of the R package gplots.

#### Cell culture and treatments

The human cell line HepG2 (ATCC, HB-8065) was cultured in minimum essential medium (MEM; Gibco, 11095080) supplemented with 10% fetal bovine serum (FBS; Dutscher, 500105H1), MEM non-essential amino acids (Gibco, 11140035), 1 mM sodium pyruvate (Gibco, 11360070) and 100 U/ml penicillin–streptomycin (Gibco, 15140). Immortalized human hepatocytes (IHH; Schippers *et al*, 1997) were cultured in William's E medium (Gibco, 22551022) supplemented with 10% FBS (Dutscher, SV30160.03), 20 mU/ml bovin insulin (Sigma, I5500), 50 nM dexamethasone (Sigma, D1756) and 100 U/ml penicillin–streptomycin (Gibco, 15140). MPH were prepared from 8 weeks old male C57BL/6J mice (Charles River) and grown as described in (Dubois *et al*, 2020a). 10 nM T3 (T6397, Sigma-Aldrich) was added to the media used for MPH isolation and *in vitro* cell culture. Cell culture was performed in a humidified atmosphere of 5% $CO_2$ in a 37°C incubator. Cell-lines were routinely monitored for mycoplasma contamination. For cell-line treatments, cells were grown in media containing 10% dextran-coated charcoal stripped serum for 48 h and then exposed to 10 nM T3 (T6397, Sigma-Aldrich), 10 nM GC-1 (SML1900, Sigma-Aldrich) or 1 μM GW3965 (405911-17-3, Tocris). Vehicle (0.01% DMSO) was used as control for GC-1 and GW3965 treatments. For serum stripping, activated charcoal solution (C9157, Sigma-Aldrich) was washed three times and prepared in ultra-pure water at final concentration 5%. Dextran T70 (2.5 g; 31390, Sigma-Aldrich) was added to the pellet and charcoal-coated dextran was mixed with 500 ml of serum and incubated overnight at 4°C. After centrifugation (20 min, 4,000 *g*, room temperature), the serum was filtered using a 0.2-μm filter and heat inactivated at 56°C for 45 min. In experiments evaluating the effects of drugs on cell growth, cells were trypsinized, resuspended in culture media, stained with trypan blue (P08-34100, PAN-Biotech) and counted using a TC20 Automated Cell Counter (BIO-RAD).

#### Mouse experiments

Young adult male C57BL/6J wild-type (WT) mice (7–11 weeks old) were purchased from Charles River and housed in standard cages in a temperature-controlled room (22–24°C) with a 12-h dark–light cycle. They had *ad libitum* access to tap water and standard chow and were allowed to acclimate for 2 weeks prior to initiation of the experimental protocol. Acute liver inflammation was induced by intraperitoneal injection of recombinant mouse IL1B using 0.5 μg/mouse (575102, BioLegend) or vehicle (PBS) for 3 h followed by T3 treatment 0.2 mpk (T6397, Sigma-Aldrich). Livers were collected 3 h later. Mice with hepatocyte-specific deletion of *Thrb* have been described in (Billon *et al*, 2014).

All animal studies were performed in compliance with EU specifications regarding the use of laboratory animals and have been approved by the Nord-Pas de Calais Ethical Committee (APAFIS#30322-202102221656794 v4).

#### RNA expression analyses

Tissues were homogenized using Minilys and 1.4 mm ceramic beads (Bertin Technologies). Total RNA was extracted from cell lines and tissues using the Nucleospin® RNA kit (Macherey-Nagel) according to the manufacturer's protocol.

RNA was reverse-transcribed using the High-Capacity cDNA Reverse Transcription Kit (Applied Biosystem). Quantitative PCR (qPCR) was performed on a Fast Applied (Applied Biosystem, Life Technologies, Cergy Pontoise, France) using the Takyon kit (Agilent Technologies). The specificity of the amplification was checked by recording the dissociation curves, and the efficiency was verified to be above 95% for each primer pair. mRNA levels were normalized to the expression of housekeeping genes and the fold induction was calculated using the cycle threshold (ΔΔCT) method. The sequences of primers used are listed in Table EV4.

For transcriptomic analyses, RNA integrity and quantity were evaluated using the Agilent 2100 Bioanalyser (Agilent Technologies). RNA was then processed for transcriptomic analysis using Affymetrix GeneChip arrays (MoGene 2.0) or high-throughput sequencing (RNA-seq) as previously described (Bobowski-Gerard *et al*, 2022).

#### Protein extraction

Proteins from the chromatin fraction were prepared as in (Dubois *et al*, 2020a). Cells were scraped in ice-cold Phosphate-Buffered Saline (PBS), pelleted by centrifugation at 400 *g* for 5 min, lysed in Buffer A (50 mM HEPES pH 7.5, 10 mM KCl, 1.5 mM $MgCl_2$, 340 mM sucrose, 10% glycerol, 1 mM DTT, and protease inhibitor cocktail from Roche) and incubated for 10 min at 4°C. Samples were centrifuged at 1,300 *g* for 5 min at 4°C and supernatants were discarded. Nuclear pellets were washed with Buffer A and subsequently lysed in solution B (3 mM EDTA, 0.2 mM EGTA, 1 mM DTT, and protease inhibitor cocktail). After incubation for 30 min at 4°C,


© 2023 The Authors

samples were centrifuged at 1,700 *g* for 5 min at 4°C and supernatants were discarded. Chromatin pellets were washed with solution B, resuspended in Buffer C (50 mM Tris–HCl pH 8.0, 1 mM MgCl2, and 83 U/μl benzonase) and incubated for 20 min at 4°C. Laemmli buffer 6× was added before loading for Western immunoblotting.

### Western blot assays

Protein extraction and western blotting were performed as described in Dubois *et al* (2020a). Protein concentrations were determined using the PierceTM BCA protein assay kit (Thermo scientific). Then, 20 μg of proteins were separated by 10% SDS–PAGE and immunodetected by Western immunoblotting using the primary antibodies listed in the Table EV5. Primary antibodies were detected using HRP-conjugated secondary antibodies (Sigma-Aldrich). Images were acquired using the iBright™ CL1500 Imaging System (Thermo Fisher Scientific).

### Statistical analyses

Statistical analyses were performed using the Prism software (GraphPad, San Diego, CA) and R (R Core Team, 2015). The specific tests and corrections for multiple testing which were used as well as the number of mice or independent biological replicates are indicated in the figure legends. Two-sided tests were used unless specified in the figure legends. All bar graphs show means ± SD (standard deviations). Box plots are composed of a box from the $25^{th}$ to the $75^{th}$ percentile with the median as a line. Unless specified in the figure legend, whiskers extent to the most extreme data point which is no more than 1.5 times the interquartile range from the box.

## Data availability

Transcriptomics data generated in this study have been deposited into the Gene Expression Omnibus (GEO) database and are available under accession numbers GSE216278 (http://www.ncbi.nlm.nih.gov/geo/query/acc.cgi?acc=GSE216278) and GSE218724 (http://www.ncbi.nlm.nih.gov/geo/query/acc.cgi?acc=GSE218724).

**Expanded View** for this article is available online.

### Acknowledgements

The authors thank Drs Wang and Liu (Vanderbilt University Medical Center, Nashville, TN, USA) for providing the summit coordinates of active mouse liver promoters, Drs Ninon Very N and Marie Bobowski-Gerard (Inserm U1011, Univ. Lille) for technical assistance and Drs David Dombrowicz and Laurent L'homme (Inserm U1011, Univ Lille) for helpful discussions.

This work was supported by the Agence Nationale de la Recherche (ANR) grants "HSCreg" (ANR-21-CE14-0032), "MEdicAL" (ANR-21-CE17-0016) and "European Genomic Institute for Diabetes" E.G.I.D (ANR-10-LABX-0046), a French State fund managed by ANR under the frame program Investissements d'Avenir I-SITE ULNE / ANR-16-IDEX-0004 ULNE as well as by Fondation pour la Recherche Médicale (FRM grants EQU202203014645).

### Author contributions

**Julie Dubois-Chevalier:** Conceptualization; data curation; formal analysis; validation; investigation; visualization; writing – review and editing. **Céline Gheeraert:** Conceptualization; data curation; formal analysis; validation; investigation; visualization; writing – review and editing. **Alexandre Berthier:** Investigation. **Clémence Boulet:** Investigation. **Vanessa Dubois:** Investigation. **Loïc Guille:** Investigation. **Marie Fourcot:** Investigation. **Guillemette Marot:** Investigation. **Karine Gauthier:** Conceptualization; resources. **Laurent Dubuquoy:** Conceptualization; resources. **Bart Staels:** Conceptualization; funding acquisition; project administration. **Philippe Lefebvre:** Conceptualization; funding acquisition; project administration. **Jérôme Eeckhoute:** Conceptualization; data curation; formal analysis; funding acquisition; visualization; writing – original draft; project administration.

### Disclosure and competing interests statement

The authors declare that they have no conflict of interest.

## References

Afgan E, Baker D, Batut B, van den Beek M, Bouvier D, Cech M, Chilton J, Clements D, Coraor N, Gruning BA *et al* (2018) The galaxy platform for accessible, reproducible and collaborative biomedical analyses: 2018 update. *Nucleic Acids Res* 46: W537 – W544

Almeida N, Chung MWH, Drudi EM, Engquist EN, Hamrud E, Isaacson A, Tsang VSK, Watt FM, Spagnoli FM (2021) Employing core regulatory circuits to define cell identity. *EMBO J* 40: e106785

Amemiya HM, Kundaje A, Boyle AP (2019) The ENCODE blacklist: identification of problematic regions of the genome. *Sci Rep* 9: 9354

Anders S, Pyl PT, Huber W (2015) HTSeq–a python framework to work with high-throughput sequencing data. *Bioinformatics* 31: 166 – 169

Arendt D, Musser JM, Baker CVH, Bergman A, Cepko C, Erwin DH, Pavlicev M, Schlosser G, Widder S, Laubichler MD *et al* (2016) The origin and evolution of cell types. *Nat Rev Genet* 17: 744 – 757

Argemi J, Latasa MU, Atkinson SR, Blokhin IO, Massey V, Gue JP, Cabezas J, Lozano JJ, Van Booven D, Bell A *et al* (2019) Defective HNF4alpha-dependent gene expression as a driver of hepatocellular failure in alcoholic hepatitis. *Nat Commun* 10: 3126

Barrett T, Wilhite SE, Ledoux P, Evangelista C, Kim IF, Tomashevsky M, Marshall KA, Phillippy KH, Sherman PM, Holko M *et al* (2013) NCBI GEO: archive for functional genomics data sets–update. *Nucleic Acids Res* 41: D991 – D995

Bedward M, Keith D, Pressey R (1992) Homogeneity analysis: assessing the utility of classifications and maps of natural resources. *Australian Journal of Ecology* 17: 133 – 139

Benayoun BA, Pollina EA, Ucar D, Mahmoudi S, Karra K, Wong ED, Devarajan K, Daugherty AC, Kundaje AB, Mancini E *et al* (2014) H3K4me3 breadth is linked to cell identity and transcriptional consistency. *Cell* 158: 673 – 688

Berasain C, Arechederra M, Argemi J, Fernandez-Barrena MG, Avila MA (2022) Loss of liver function in chronic liver disease: an identity crisis. *J Hepatol* 78: 401 – 414

Bideyan L, Nagari R, Tontonoz P (2021) Hepatic transcriptional responses to fasting and feeding. *Genes Dev* 35: 635 – 657

Billon C, Canaple L, Fleury S, Deloire A, Beylot M, Dombrowicz D, Del Carmine P, Samarut J, Gauthier K (2014) TRalpha protects against atherosclerosis in male mice: identification of a novel anti-inflammatory property for TRalpha in mice. *Endocrinology* 155: 2735 – 2745

Bobowski-Gerard M, Boulet C, Zummo FP, Dubois-Chevalier J, Gheeraert C, Bou Saleh M, Strub JM, Farce A, Ploton M, Guille L *et al* (2022) Functional genomics uncovers the transcription factor BNC2 as required for myofibroblastic activation in fibrosis. *Nat Commun* 13: 5324

© 2023 The Authors 

Bou Saleh M, Louvet A, Ntandja-Wandji LC, Boleslawski E, Gnemmi V, Lassailly G, Truant S, Maggiotto F, Ningarhari M, Artru F *et al* (2021) Loss of hepatocyte identity following aberrant YAP activation: a key mechanism in alcoholic hepatitis. *J Hepatol* 75: 912 – 923

Boyer LA, Lee TI, Cole MF, Johnstone SE, Levine SS, Zucker JP, Guenther MG, Kumar RM, Murray HL, Jenner RG *et al* (2005) Core transcriptional regulatory circuitry in human embryonic stem cells. *Cell* 122: 947 – 956

Chao J, Zhao S, Sun H (2020) Dedifferentiation of hepatocellular carcinoma: molecular mechanisms and therapeutic implications. *Am J Transl Res* 12: 2099 – 2109

Chen J, Xu H, Aronow BJ, Jegga AG (2007) Improved human disease candidate gene prioritization using mouse phenotype. *BMC Bioinformatics* 8: 392

Chen K, Chen Z, Wu D, Zhang L, Lin X, Su J, Rodriguez B, Xi Y, Xia Z, Chen X *et al* (2015) Broad H3K4me3 is associated with increased transcription elongation and enhancer activity at tumor-suppressor genes. *Nat Genet* 47: 1149 – 1157

Cheng X, Liu Y, Wang J, Chen Y, Robertson AG, Zhang X, Jones SJM, Taubert S (2022) cSurvival: a web resource for biomarker interactions in cancer outcomes and in cell lines. *Brief Bioinform* 23: bbac090

Chubb JR, Ford HZ, Antolović V (2021) Distributed and centralized control during differentiation. *Dev Cell* 56: 2142 – 2144

D'Alessio Ana C, Fan Zi P, Wert Katherine J, Baranov P, Cohen Malkiel A, Saini Janmeet S, Cohick E, Charniga C, Dadon D, Hannett Nancy M *et al* (2015) A systematic approach to identify candidate transcription factors that control cell identity. *Stem Cell Reports* 5: 763 – 775

Dalgaard K, Landgraf K, Heyne S, Lempradl A, Longinotto J, Gossens K, Ruf M, Orthofer M, Strogantsev R, Selvaraj M *et al* (2016) Trim28 Haploinsufficiency triggers Bi-stable epigenetic obesity. *Cell* 164: 353 – 364

Del Campo JA, Gallego P, Grande L (2018) Role of inflammatory response in liver diseases: therapeutic strategies. *World J Hepatol* 10: 1 – 7

Dubois V, Gheeraert C, Vankrunkelsven W, Dubois-Chevalier J, Dehondt H, Bobowski-Gerard M, Vinod M, Zummo FP, Güiza F, Ploton M *et al* (2020a) Endoplasmic reticulum stress actively suppresses hepatic molecular identity in damaged liver. *Mol Syst Biol* 16: e9156

Dubois V, Staels B, Lefebvre P, Verzi MP, Eeckhoute J (2020b) Control of cell identity by the nuclear receptor HNF4 in organ pathophysiology. *Cell* 9: 2185

Dubois-Chevalier J, Dubois V, Dehondt H, Mazrooei P, Mazuy C, Sérandour AA, Gheeraert C, Guillaume P, Baugé E, Derudas B *et al* (2017a) The logic of transcriptional regulator recruitment architecture at cis-regulatory modules controlling liver functions. *Genome Res* 27: 985 – 996

Dubois-Chevalier J, Mazrooei P, Lupien M, Staels B, Lefebvre P, Eeckhoute J (2017b) Organizing combinatorial transcription factor recruitment at cis-regulatory modules. *Transcription* 9: 1 – 7

Dubois-Chevalier J, Dubois V, Staels B, Lefebvre P, Eeckhoute J (2020) Perspectives on the use of super-enhancers as a defining feature of cell/tissue-identity genes. *Epigenomics* 12: 715 – 723

Duncan AW, Taylor MH, Hickey RD, Newell AEH, Lenzi ML, Olson SB, Finegold MJ, Grompe M (2010) The ploidy conveyor of mature hepatocytes as a source of genetic variation. *Nature* 467: 707 – 710

Frankish A, Diekhans M, Ferreira AM, Johnson R, Jungreis I, Loveland J, Mudge JM, Sisu C, Wright J, Armstrong J *et al* (2018) GENCODE reference annotation for the human and mouse genomes. *Nucleic Acids Res* 47: D766 – D773

Freese NH, Norris DC, Loraine AE (2016) Integrated genome browser: visual analytics platform for genomics. *Bioinformatics* 32: 2089 – 2095

Gao J, Aksoy BA, Dogrusoz U, Dresdner G, Gross B, Sumer SO, Sun Y, Jacobsen A, Sinha R, Larsson E *et al* (2013) Integrative analysis of complex cancer genomics and clinical profiles using the cBioPortal. *Sci Signal* 6: pl1

Gartner K (1990) A third component causing random variability beside environment and genotype. A reason for the limited success of a 30 year long effort to standardize laboratory animals? *Lab Anim* 24: 71 – 77

González-Blas CB, Matetovici I, Hillen H, Taskiran II, Vandepoel R, Christiaens V, Sansores-García L, Verboven E, Hulselmans G, Poovathingal S *et al* (2022) Enhancer grammar of liver cell types and hepatocyte zonation states. *bioRxiv* https://doi.org/10.1101/2022.12.08.519575 [PREPRINT]

Guille L, Johanns M, Zummo FP, Staels B, Lefebvre P, Eeckhoute J, Dubois-Chevalier J (2022) ISCEBERG: interactive single cell expression browser for exploration of RNAseq data using graphics (v1.0.1). *Zenodo* https://doi.org/10.5281/zenodo.6563734 [PREPRINT]

Guilliams M, Bonnardel J, Haest B, Vanderborght B, Wagner C, Remmerie A, Bujko A, Martens L, Thone T, Browaeys R *et al* (2022) Spatial proteogenomics reveals distinct and evolutionarily conserved hepatic macrophage niches. *Cell* 185: 379 – 396

Gunewardena S, Huck I, Walesky C, Robarts D, Weinman S, Apte U (2022) Progressive loss of HNF4alpha activity in chronic liver diseases in humans. *Hepatology* 76: 372 – 386

Hahsler M, Gruen B, Hornik K (2005) Arules – a computational environment for mining association rules and frequent item sets. *J Stat Softw* 14: 1 – 25

Hao Y, Hao S, Andersen-Nissen E, Mauck WM 3rd, Zheng S, Butler A, Lee MJ, Wilk AJ, Darby C, Zager M *et al* (2021) Integrated analysis of multimodal single-cell data. *Cell* 184: 3573 – 3587

Hatziagelaki E, Paschou SA, Schon M, Psaltopoulou T, Roden M (2022) NAFLD and thyroid function: pathophysiological and therapeutic considerations. *Trends Endocrinol Metab* 33: 755 – 768

Hnisz D, Abraham BJ, Lee TI, Lau A, Saint-Andre V, Sigova AA, Hoke HA, Young RA (2013) Super-enhancers in the control of cell identity and disease. *Cell* 155: 934 – 947

Howe KL, Achuthan P, Allen J, Allen J, Alvarez-Jarreta J, Amode MR, Armean IM, Azov AG, Bennett R, Bhai J *et al* (2021) Ensembl 2021. *Nucleic Acids Res* 49: D884 – D891

Hunter AL, Pelekanou CE, Adamson A, Downton P, Barron NJ, Cornfield T, Poolman TM, Humphreys N, Cunningham PS, Hodson L *et al* (2020) Nuclear receptor REVERBalpha is a state-dependent regulator of liver energy metabolism. *Proc Natl Acad Sci U S A* 117: 25869 – 25879

Hyun J, Sun Z, Ahmadi AR, Bangru S, Chembazhi UV, Du K, Chen T, Tsukamoto H, Rusyn I, Kalsotra A *et al* (2020) Epithelial splicing regulatory protein 2-mediated alternative splicing reprograms hepatocytes in severe alcoholic hepatitis. *J Clin Invest* 130: 2129 – 2145

Kaimal V, Bardes EE, Tabar SC, Jegga AG, Aronow BJ (2010) ToppCluster: a multiple gene list feature analyzer for comparative enrichment clustering and network-based dissection of biological systems. *Nucleic Acids Res* 38: W96 – W102

Kim D, Paggi JM, Park C, Bennett C, Salzberg SL (2019) Graph-based genome alignment and genotyping with HISAT2 and HISAT-genotype. *Nat Biotechnol* 37: 907 – 915

Kowalik MA, Puliga E, Cabras L, Sulas P, Petrelli A, Perra A, Ledda-Columbano GM, Morandi A, Merlin S, Orru C *et al* (2020) Thyroid hormone inhibits hepatocellular carcinoma progression via induction of differentiation and metabolic reprogramming. *J Hepatol* 72: 1159 – 1169

Kryuchkova-Mostacci N, Robinson-Rechavi M (2017) A benchmark of gene expression tissue-specificity metrics. *Brief Bioinform* 18: 205 – 214

Kyrmizi I, Hatzis P, Katrakili N, Tronche F, Gonzalez FJ, Talianidis I (2006) Plasticity and expanding complexity of the hepatic transcription factor network during liver development. *Genes Dev* 20: 2293 – 2305

Lê S, Josse J, Husson F (2008) FactoMineR: an R package for multivariate analysis. *J Stat Softw* 25: 1 – 18

 © 2023 The Authors

Leek JT, Johnson WE, Parker HS, Fertig EJ, Jaffe AE, Zhang Y, Storey JD, Torres LC (2022) sva: Surrogate Variable Analysis. *R package version 3440*

Liang B, Zhou Y, Qian M, Xu M, Wang J, Zhang Y, Song X, Wang H, Lin S, Ren C *et al* (2021) TBX3 functions as a tumor suppressor downstream of activated CTNNB1 mutants during hepatocarcinogenesis. *J Hepatol* 75: 120 – 131

Lin Z, Xia S, Liang Y, Ji L, Pan Y, Jiang S, Wan Z, Tao L, Chen J, Lin C *et al* (2020) LXR activation potentiates sorafenib sensitivity in HCC by activating microRNA-378a transcription. *Theranostics* 10: 8834 – 8850

Liu R, Holik AZ, Su S, Jansz N, Chen K, Leong HS, Blewitt ME, Asselin-Labat ML, Smyth GK, Ritchie ME (2015) Why weight? Modelling sample and observational level variability improves power in RNA-seq analyses. *Nucleic Acids Res* 43: e97

Lizio M, Harshbarger J, Shimoji H, Severin J, Kasukawa T, Sahin S, Abugessaisa I, Fukuda S, Hori F, Ishikawa-Kato S *et al* (2015) Gateways to the FANTOM5 promoter level mammalian expression atlas. *Genome Biol* 16: 22

Lo JH, Edwards M, Langerman J, Sridharan R, Plath K, Smale ST (2022) Oct4: Sox2 binding is essential for establishing but not maintaining active and silent states of dynamically regulated genes in pluripotent cells. *Genes Dev* 36: 1079 – 1095

Loft A, Alfaro AJ, Schmidt SF, Pedersen FB, Terkelsen MK, Puglia M, Chow KK, Feuchtinger A, Troullinaki M, Maida A *et al* (2021) Liver-fibrosis-activated transcriptional networks govern hepatocyte reprogramming and intra-hepatic communication. *Cell Metab* 33: 1685 – 1700

Loven J, Hoke HA, Lin CY, Lau A, Orlando DA, Vakoc CR, Bradner JE, Lee TI, Young RA (2013) Selective inhibition of tumor oncogenes by disruption of super-enhancers. *Cell* 153: 320 – 334

Ma H, de Zwaan E, Guo YE, Cejas P, Thiru P, van de Bunt M, Jeppesen JF, Syamala S, Dall'Agnese A, Abraham BJ *et al* (2022) The nuclear receptor THRB facilitates differentiation of human PSCs into more mature hepatocytes. *Cell Stem Cell* 29: 1611

Meng H, Gonzales NM, Lonard DM, Putluri N, Zhu B, Dacso CC, York B, O'Malley BW (2020) XBP1 links the 12-hour clock to NAFLD and regulation of membrane fluidity and lipid homeostasis. *Nat Commun* 11: 6215

Mittnenzweig M, Mayshar Y, Cheng S, Ben-Yair R, Hadas R, Rais Y, Chomsky E, Reines N, Uzonyi A, Lumerman L *et al* (2021) A single-embryo, single-cell time-resolved model for mouse gastrulation. *Cell* 184: 2825 – 2842

Mukherji A, Bailey SM, Staels B, Baumert TF (2019) The circadian clock and liver function in health and disease. *J Hepatol* 71: 200 – 211

Nishikawa T, Bell A, Brooks JM, Setoyama K, Melis M, Han B, Fukumitsu K, Handa K, Tian J, Kaestner KH *et al* (2015) Resetting the transcription factor network reverses terminal chronic hepatic failure. *J Clin Invest* 125: 1533 – 1544

Pan Y, Ballance H, Meng H, Gonzalez N, Kim SM, Abdurehman L, York B, Chen X, Schnytzer Y, Levy O *et al* (2020) 12-h clock regulation of genetic information flow by XBP1s. *PLoS Biol* 18: e3000580

Papadakis M, Tsagris M, Dimitriadis M, Fafalios S, Tsamardinos I, Fasiolo M, Borboudakis G, Burkardt J, Zou C, Lakiotaki K *et al* (2022) Rfast: A Collection of Efficient and Extremely Fast R Functions. R package version 2.0.6. https://CRAN.R-project.org/package=Rfast

Paquette MA, Dong H, Gagne R, Williams A, Malowany M, Wade MG, Yauk CL (2011) Thyroid hormone-regulated gene expression in juvenile mouse liver: identification of thyroid response elements using microarray profiling and in silico analyses. *BMC Genomics* 12: 634

Paris J, Henderson NC (2022) Liver zonation, revisited. *Hepatology* 76: 1219 – 1230

Pekowska A, Benoukraf T, Ferrier P, Spicuglia S (2010) A unique H3K4me2 profile marks tissue-specific gene regulation. *Genome Res* 20: 1493 – 1502

Ploton M, Mazuy C, Gheeraert C, Dubois V, Berthier A, Dubois-Chevalier J, Marechal X, Bantubungi K, Diemer H, Cianferani S *et al* (2018) The nuclear bile acid receptor FXR is a PKA- and FOXA2-sensitive activator of fasting hepatic gluconeogenesis. *J Hepatol* 69: 1099 – 1109

Pohl A, Beato M (2014) Bwtool: a tool for bigWig files. *Bioinformatics* 30: 1618 – 1619

Quinlan AR, Hall IM (2010) BEDTools: a flexible suite of utilities for comparing genomic features. *Bioinformatics* 26: 841 – 842

R Core Team (2015) *R: a language and environment for statistical computing*. Vienna: R Foundation for Statistical Computing

Raney BJ, Cline MS, Rosenbloom KR, Dreszer TR, Learned K, Barber GP, Meyer LR, Sloan CA, Malladi VS, Roskin KM *et al* (2011) ENCODE whole-genome data in the UCSC genome browser (2011 update). *Nucleic Acids Res* 39 (Suppl 1): D871 – D875

Reizel Y, Morgan A, Gao L, Lan Y, Manduchi E, Waite EL, Wang AW, Wells A, Kaestner KH (2020) Collapse of the hepatic gene regulatory network in the absence of FoxA factors. *Genes Dev* 34: 1039 – 1050

Ritchie ME, Phipson B, Wu D, Hu Y, Law CW, Shi W, Smyth GK (2015) Limma powers differential expression analyses for RNA-sequencing and microarray studies. *Nucleic Acids Res* 43: e47

Robinson MD, McCarthy DJ, Smyth GK (2010) edgeR: a Bioconductor package for differential expression analysis of digital gene expression data. *Bioinformatics* 26: 139 – 140

Russo-Savage L, Schulman IG (2021) Liver X receptors and liver physiology. *Biochim Biophys Acta Mol Basis Dis* 1867: 166121

Sarkans U, Fullgrabe A, Ali A, Athar A, Behrangi E, Diaz N, Fexova S, George N, Iqbal H, Kurri S *et al* (2021) From ArrayExpress to BioStudies. *Nucleic Acids Res* 49: D1502 – D1506

Schippers IJ, Moshage H, Roelofsen H, Muller M, Heymans HS, Ruiters M, Kuipers F (1997) Immortalized human hepatocytes as a tool for the study of hepatocytic (de-)differentiation. *Cell Biol Toxicol* 13: 375 – 386

Schmidt D, Wilson MD, Ballester B, Schwalie PC, Brown GD, Marshall A, Kutter C, Watt S, Martinez-Jimenez CP, Mackay S *et al* (2010) Five-vertebrate ChIP-seq reveals the evolutionary dynamics of transcription factor binding. *Science* 328: 1036 – 1040

Seirup M, Sengupta S, Swanson S, McIntosh BE, Collins M, Chu LF, Cheng Z, Gorkin DU, Duffin B, Bolin JM *et al* (2022) Rapid changes in chromatin structure during dedifferentiation of primary hepatocytes *in vitro*. *Genomics* 114: 110330

Shen Y, Yue F, McCleary DF, Ye Z, Edsall L, Kuan S, Wagner U, Dixon J, Lee L, Lobanenkov VV *et al* (2012) A map of the cis-regulatory sequences in the mouse genome. *Nature* 488: 116 – 120

Singh BK, Sinha RA, Tripathi M, Mendoza A, Ohba K, Sy JAC, Xie SY, Zhou J, Ho JP, Chang CY *et al* (2018) Thyroid hormone receptor and ERRalpha coordinately regulate mitochondrial fission, mitophagy, biogenesis, and function. *Sci Signal* 11: eaam5855

Smith CL, Goldsmith CA, Eppig JT (2005) The mammalian phenotype ontology as a tool for annotating, analyzing and comparing phenotypic information. *Genome Biol* 6: R7

Sommars MA, Ramachandran K, Senagolage MD, Futtner CR, Germain DM, Allred AL, Omura Y, Bederman IR, Barish GD (2019) Dynamic repression by BCL6 controls the genome-wide liver response to fasting and steatosis. *Elife* 8: e43922

Sun J, Wang B, Liu Y, Zhang L, Ma A, Yang Z, Ji Y, Liu Y (2014) Transcription factor KLF9 suppresses the growth of hepatocellular carcinoma cells in vivo and positively regulates p53 expression. *Cancer Lett* 355: 25 – 33

Sun P, Zhang G, Su X, Jin C, Yu B, Yu X, Lv Z, Ma H, Zhang M, Wei W *et al* (2019) Maintenance of primary hepatocyte functions In vitro by inhibiting mechanical tension-induced YAP activation. *Cell Rep* 29: 3212 – 3222

© 2023 The Authors

Tachmatzidi EC, Galanopoulou O, Talianidis I (2021) Transcription control of liver development. *Cell* 10: 2026

Tafaleng EN, Mukherjee A, Bell A, Morita K, Guzman-Lepe J, Haep N, Florentino RM, Diaz-Aragon R, Frau C, Ostrowska A *et al* (2021) Hepatocyte nuclear factor 4 alpha 2 messenger RNA reprograms liver-enriched transcription factors and functional proteins in end-stage cirrhotic human hepatocytes. *Hepatol Commun* 5: 1911 – 1926

Tan HW, Leung CO, Chan KK, Ho DW, Leung MS, Wong CM, Ng IO, Lo RC (2019) Deregulated GATA6 modulates stem cell-like properties and metabolic phenotype in hepatocellular carcinoma. *Int J Cancer* 145: 1860 – 1873

Tang Q, Zeng M, Chen L, Fu N (2022) Targeting thyroid hormone/thyroid hormone receptor Axis: an attractive therapy strategy in liver diseases. *Front Pharmacol* 13: 871100

Tuttle AH, Philip VM, Chesler EJ, Mogil JS (2018) Comparing phenotypic variation between inbred and outbred mice. *Nat Methods* 15: 994 – 996

Vandel J, Gheeraert C, Staels B, Eeckhoute J, Lefebvre P, Dubois-Chevalier J (2020) GIANT: galaxy-based tool for interactive analysis of transcriptomic data. *Sci Rep* 10: 19835

Vazquez Salgado AM, Preziosi ME, Yin D, Holczbauer A, Zahm AM, Erez N, Kieckhaefer J, Ackerman D, Gade TP, Kaestner KH *et al* (2022) In vivo screen identifies liver X receptor alpha Agonism potentiates Sorafenib killing of hepatocellular carcinoma. *Gastro Hep Adv* 1: 905 – 908

Wang J, Zhao Y, Zhou X, Hiebert SW, Liu Q, Shyr Y (2018) Nascent RNA sequencing analysis provides insights into enhancer-mediated gene regulation. *BMC Genomics* 19: 633

Wehrens R, Buydens L (2007) Self- and super-organizing maps in R: the kohonen package. *J Stat Softw* 21: 1 – 19

Whyte WA, Orlando DA, Hnisz D, Abraham BJ, Lin CY, Kagey MH, Rahl PB, Lee TI, Young RA (2013) Master transcription factors and mediator establish super-enhancers at key cell identity genes. *Cell* 153: 307 – 319

Wickham H (2016) *ggplot2: elegant graphics for data analysis*. New York, NY: Springer-Verlag

Wilkinson AC, Nakauchi H, Gottgens B (2017) Mammalian transcription factor networks: recent advances in interrogating biological complexity. *Cell Syst* 5: 319 – 331

Wu C, Jin X, Tsueng G, Afrasiabi C, Su AI (2016) BioGPS: building your own mash-up of gene annotations and expression profiles. *Nucleic Acids Res* 44: D313 – D316

Wu C, Orozco C, Boyer J, Leglise M, Goodale J, Batalov S, Hodge CL, Haase J, Janes J, Huss 3rd JW, Su AI (2009) BioGPS: an extensible and customizable portal for querying and organizing gene annotation resources. *Genome Biol* 10: R130

Xiang C, Du Y, Meng G, Soon Yi L, Sun S, Song N, Zhang X, Xiao Y, Wang J, Yi Z *et al* (2019) Long-term functional maintenance of primary human hepatocytes *in vitro*. *Science* 364: 399 – 402

Yang T, Poenisch M, Khanal R, Hu Q, Dai Z, Li R, Song G, Yuan Q, Yao Q, Shen X *et al* (2021) Therapeutic HNF4A mRNA attenuates liver fibrosis in a preclinical model. *J Hepatol* 75: 1420 – 1433

Yang CH, Fagnocchi L, Apostle S, Wegert V, Casani-Galdon S, Landgraf K, Panzeri I, Dror E, Heyne S, Worpel T *et al* (2022) Independent phenotypic plasticity axes define distinct obesity sub-types. *Nat Metab* 4: 1150 – 1165

Yates AD, Achuthan P, Akanni W, Allen J, Allen J, Alvarez-Jarreta J, Amode MR, Armean IM, Azov AG, Bennett R *et al* (2020) Ensembl 2020. *Nucleic Acids Res* 48: D682 – D688

Yuan C, Lin JZ, Sieglaff DH, Ayers SD, Denoto-Reynolds F, Baxter JD, Webb P (2012) Identical gene regulation patterns of T3 and selective thyroid hormone receptor modulator GC-1. *Endocrinology* 153: 501 – 511

Zhang Y, Liu T, Meyer CA, Eeckhoute J, Johnson DS, Bernstein BE, Nusbaum C, Myers RM, Brown M, Li W *et al* (2008) Model-based analysis of ChIP-Seq (MACS). *Genome Biol* 9: R137

Yue F, Cheng Y, Breschi A, Vierstra J, Wu W, Ryba T, Sandstrom R, Ma Z, Davis C, Pope BD *et al* (2014) A comparative encyclopedia of DNA elements in the mouse genome. *Nature* 515: 355 – 364

Zhang HM, Liu T, Liu CJ, Song S, Zhang X, Liu W, Jia H, Xue Y, Guo AY (2015) AnimalTFDB 2.0: a resource for expression, prediction and functional study of animal transcription factors. *Nucleic Acids Res* 43: D76 – D81

Zhou Q, Liu M, Xia X, Gong T, Feng J, Liu W, Liu Y, Zhen B, Wang Y, Ding C *et al* (2017) A mouse tissue transcription factor atlas. *Nat Commun* 8: 15089


**License:** This is an open access article under the terms of the Creative Commons Attribution License, which permits use, distribution and reproduction in any medium, provided the original work is properly cited.


© 2023 The Authors

# Expanded View Figures

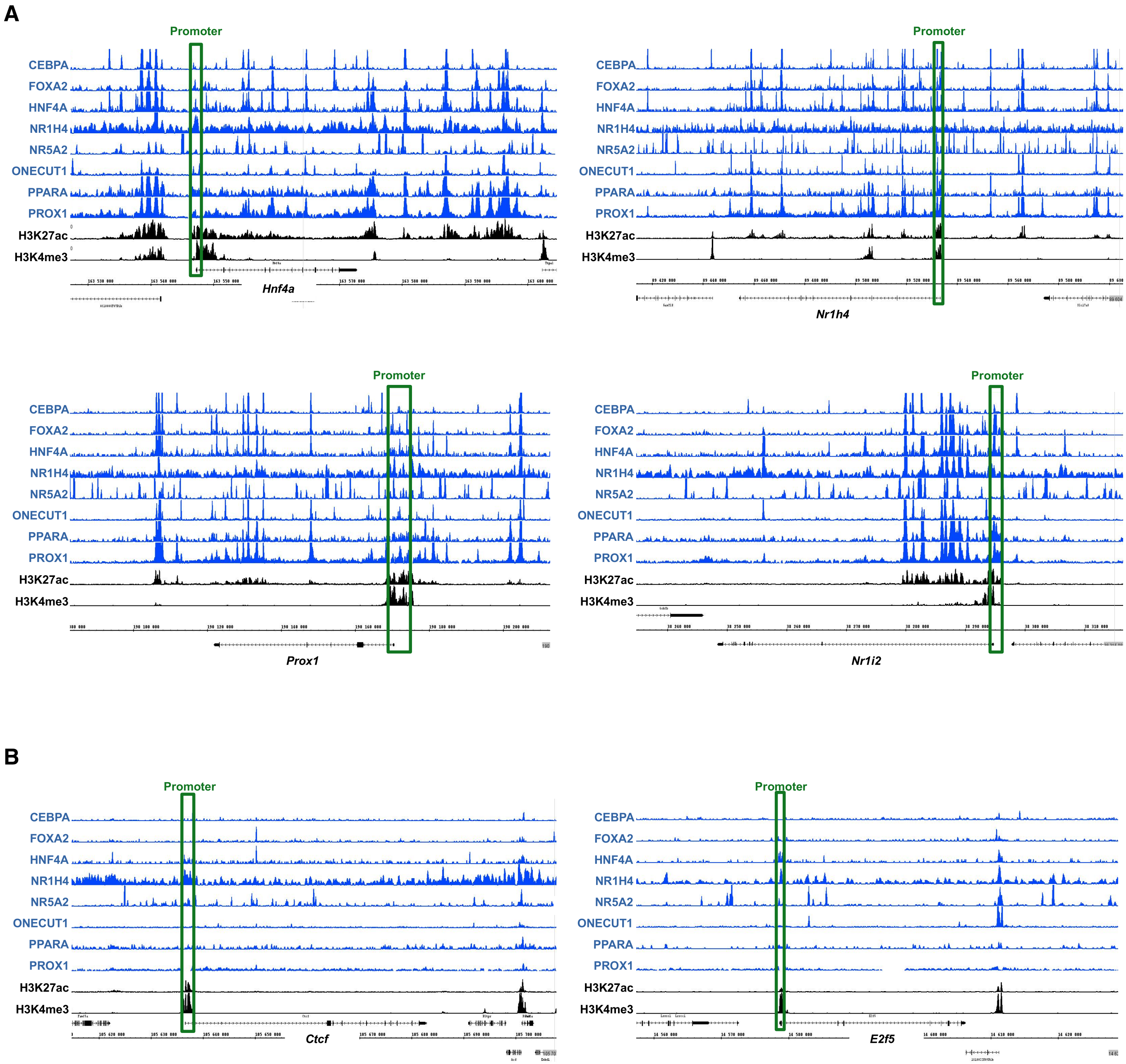


**Figure EV1. Hep-ID TF cistromes at example TF-encoding gene loci.**

A, B The Integrated Genome Browser (IGB) was used to display the cistromes of the indicated eight Hep-ID TFs together with levels of H3K4me3 and H3K27ac from mouse liver ChIP-seq data (Dataset EV2). Example Hep-ID TF (A) and control TF-encoding genes (B) are shown. The promoters are highlighted by green boxes. The scales of the individual ChIP-seq tracks were kept constant for all analyzed genes.

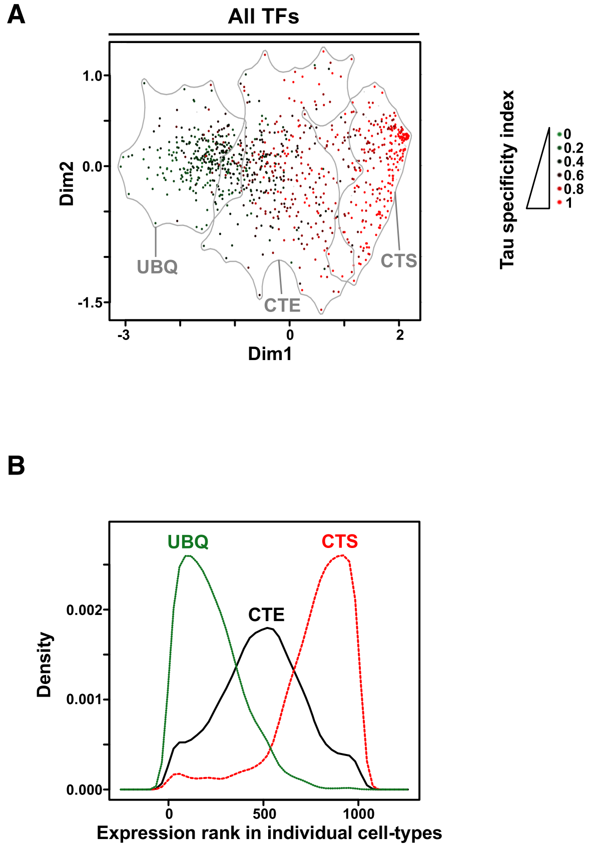


**Figure EV2. Characterization of CTS, CTE, and UBQ TF genes.**

A Data were displayed as in Fig 1J to show the Tau index of tissue-specific expression for individual TF genes within the CTS (cell-type specific), CTE (cell-type enriched), and UBQ (ubiquitous) clusters.

B Density plot showing the distribution of the expression rank of CTS, CTE, and UBQ TF-encoding genes in primary mouse cell-types ($n$ = 39). All TF genes were ranked from high to low expression (i.e., from 0 to 1,009 in each cell-type) and the distribution of TFs from the CTS, CTE and UBQ groups are shown. As expected, CTS TFs display low ranks in a very limited subsets of cell-types while having high ranks in most cell-types, which is the opposite from the pattern obtained for UBQ TFs.

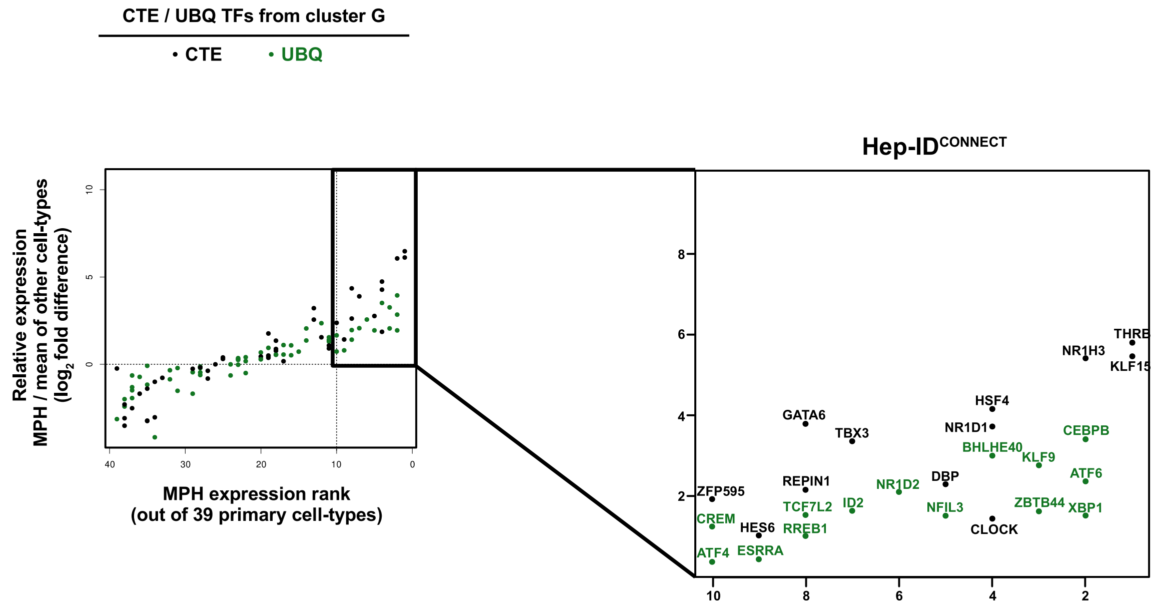


**Figure EV3. CTE and UBQ TF genes with privileged expression in MPH.**

The right shows a zoomed view of CTE (black) and UBQ (green) TFs from cluster G comprised within the framed area from Fig 2A (shown again on the left).

 © 2023 The Authors

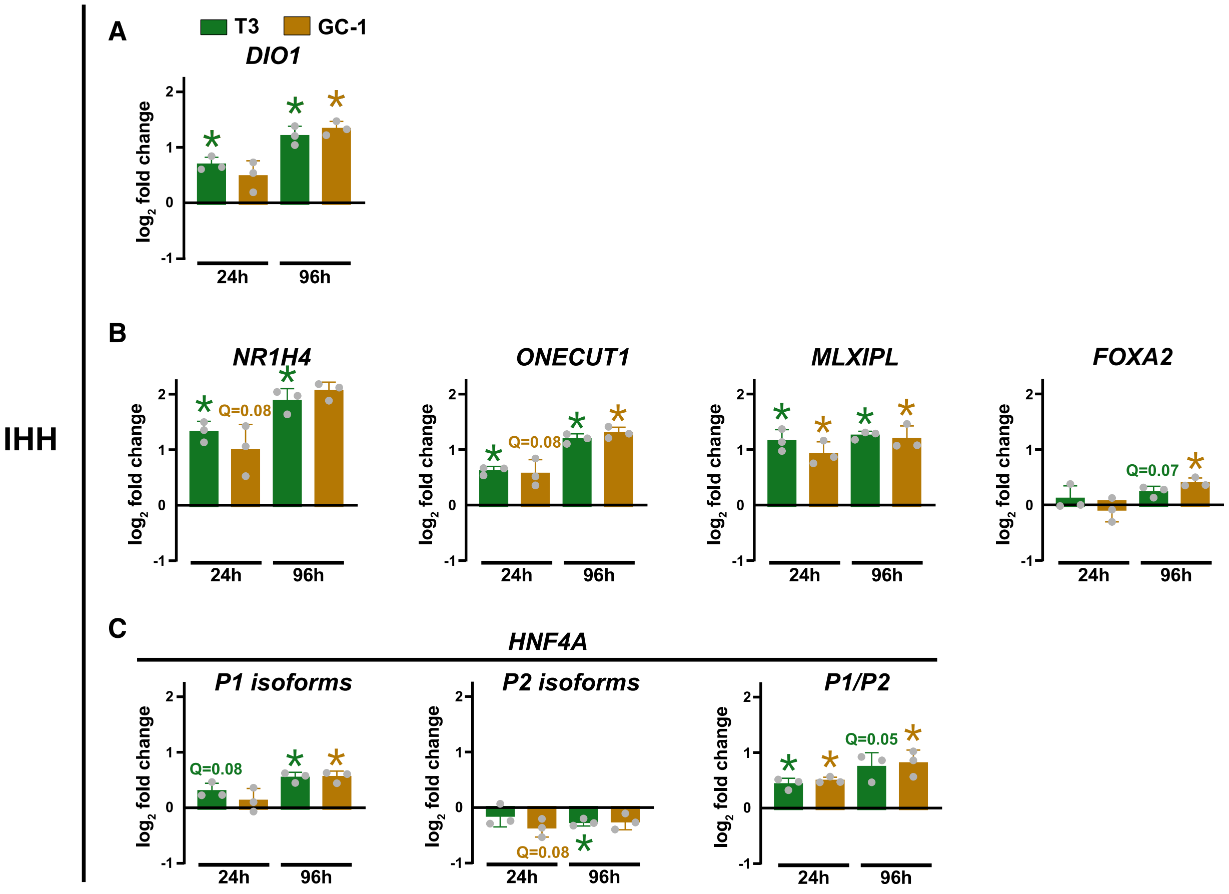


**Figure EV4. T3-mediated regulation of Hep-ID TF gene expression in human IHH cells.**

A–C mRNA expression of the indicated genes was monitored using RT-qPCR in IHH cells treated with T3 or GC-1 for 24 or 96 h. Bar graphs show mean ± SD ($n$ = 3 biological replicates) of $\log_2$ fold changes in treated *versus* untreated HepG2 cells. For *Hnf4a*, the $\log_2$ fold change in the ratio of P1 over P2 promoter-derived isoforms is also shown. Gray dots show the results obtained from the three independent biological replicates. One-sample *t*-test with Benjamini–Hochberg correction for multiple testing was used to determine if the mean $\log_2$ FC was statistically different from 0. *q < 0.05.

**Figure EV5. Acute IL1B challenge triggers partial hepatic loss of identity.**

A Analysis similar to that shown in Appendix Fig S6 showing that IL1B treatment induced a hepatic transcriptomic profile leaning towards that of not fully mature hepatocytes pointing to partial dedifferentiation.

B Dot plots showing the transcriptional regulation of individual Hep-ID TF gene expression in livers of IL1B-challenged mice compared to non-treated animals issued from transcriptomic analyses.

C Correlation between *Dio1* and *Ccl2* mRNA expression levels assessed using RT-qPCR and livers of all mice treated with IL1B + T3 from Fig 5. Gene expression are $\log_2$ FC relative to control (PBS injected) mice. Linear regression and coefficient of determination ($r^2$) are shown.

D Gene expression levels of *Ly6g* (neutrophil marker) and *Ptprc* (also known as *CD45*; broad immune cell marker) were analyzed as described for Fig 5B–D. Box plots are composed of a box from the 25th to the 75th percentile with the median as a line ($n$ = 13 mice for the PBS group, 17 for the IL1B group and 10 for the other groups). Whiskers extent to the most extreme data point which is no more than 1.5 times the interquartile range from the box.

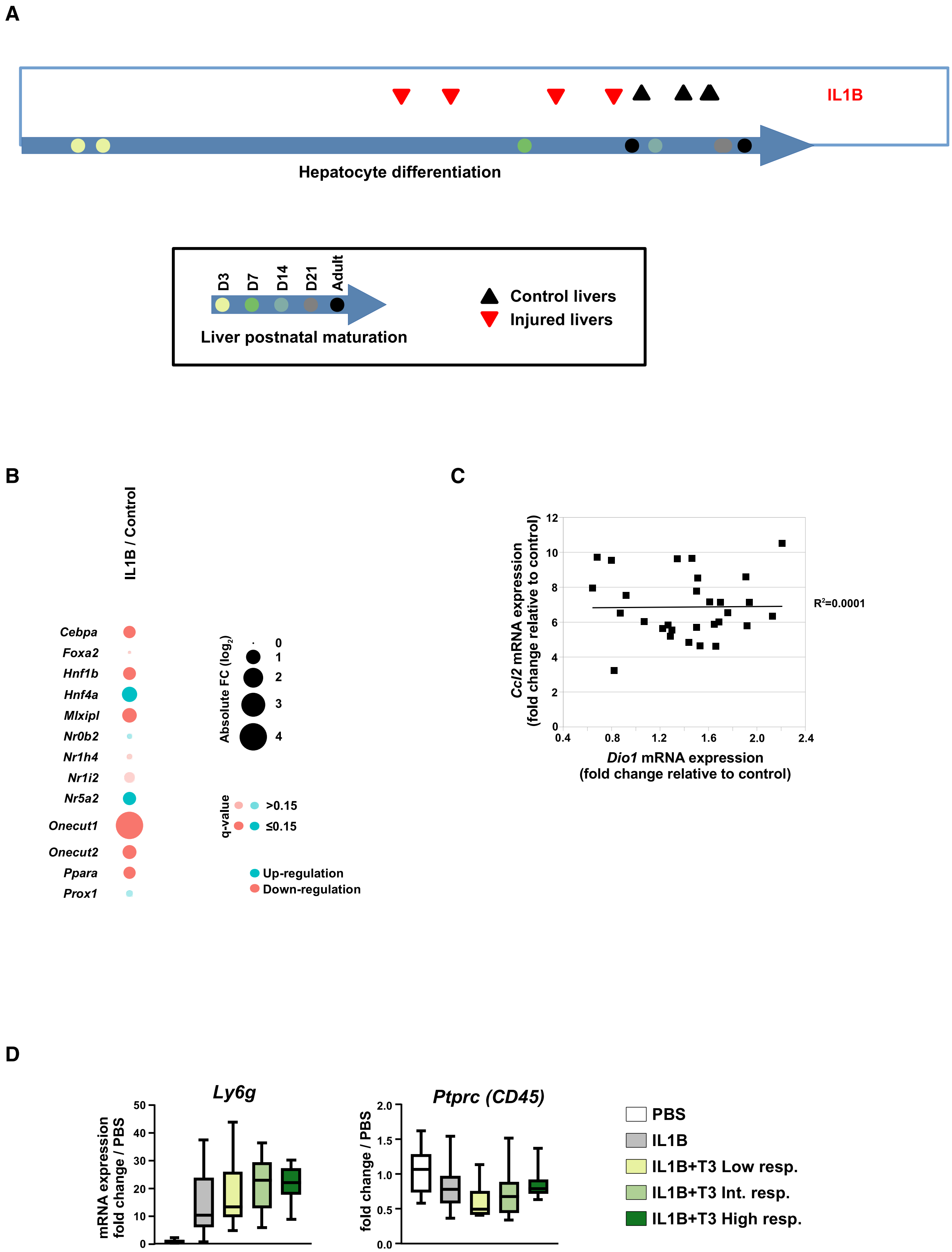


**Figure EV5.**


© 2023 The Authors

**Appendix table of contents**

Appendix Figure S1. Characteristics of the mouse liver TF-encoding gene promoters and of the SOM issued from their analysis (p2-3).

Appendix Figure S2. Characterization of the main clusters of TF-encoding gene promoters (p3-4).

Appendix Figure S3. Transcriptional regulator co-recruitment at promoters from clusters A-G (p4-13).

Appendix Figure S4. Additional characterization of the main clusters of TF-encoding gene promoters (p14-15).

Appendix Figure S5. Average expression of Hep-ID and Hep-ID$^{CONNECT}$ TF encoding genes in individual mouse and human liver cell types (p16-17).

Appendix Figure S6. Loss of hepatocyte molecular identity in mouse models of severe liver injury (p18-19).

Appendix Figure S7. Length of H3K4me3 domains at the promoter of TF-encoding genes in various mouse tissues/organs (p20-21).

Appendix Figure S8. Association between Hep-ID$^{CONNECT}$ TFs and liver/hepatocyte metabolism in the scientific literature (p22-23).

Appendix Figure S9. Strategy used to compare TF binding to the promoters of identity effector genes and activity-matched control non-TF gene promoters (p24-25).

Appendix Figure S10. Strategy used to compare TF binding to or transcriptional regulation of Hep-ID TFs and activity-matched control TF genes (p26-27).

Appendix Figure S11. Biological pathways linked to Hep-ID, Hep-ID$^{CONNECT}$ and remaining TF-encoding genes from cluster G (Others) (p28-29).

Appendix Figure S12. Clinical features associated with HCC displaying low or high expression levels of Hep-ID$^{CONNECT}$ TF genes (p30-31).

Appendix Figure S13. T3-mediated transcriptional regulation of Hep-ID TF genes in livers of healthy mice (p32-33).

Appendix Figure S14. T3-mediated transcriptional regulation of Hep-ID TF genes in MPH (p34-35).

Fig.S1

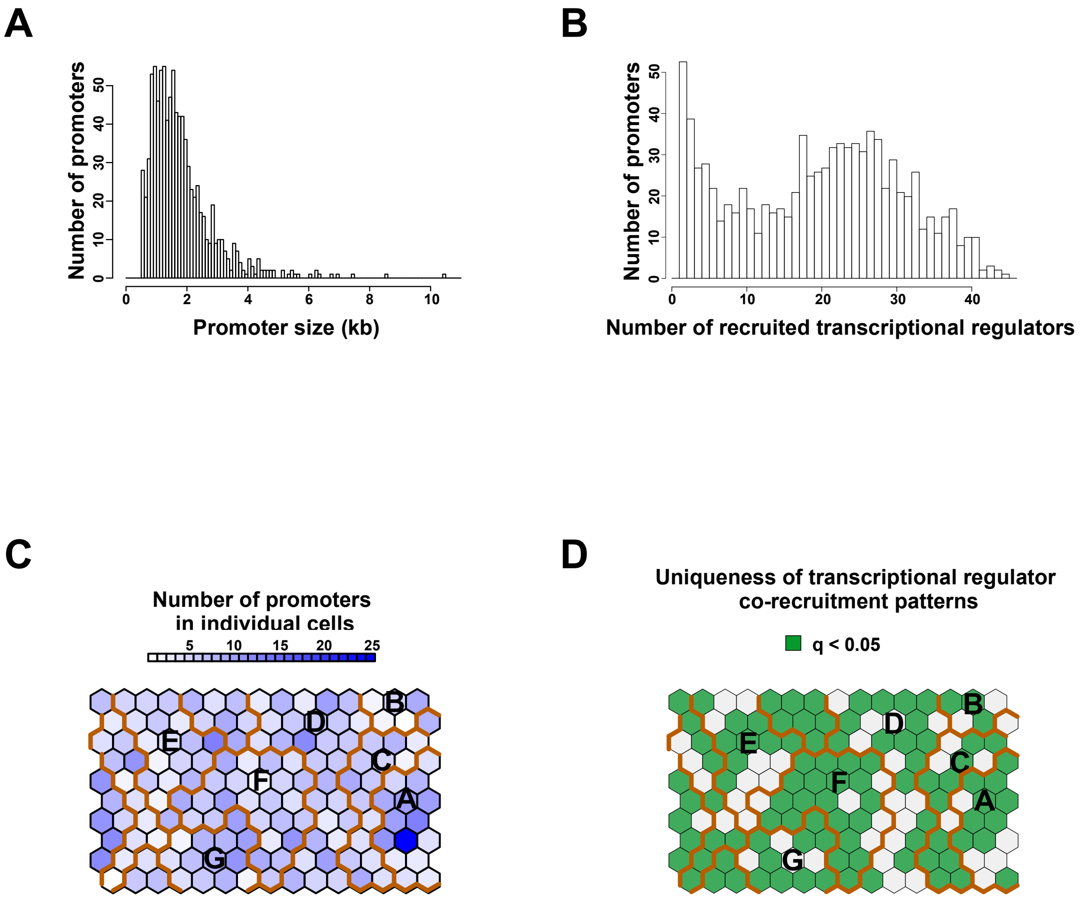

A
Number of promoters
0
10
20
30
40
50
0
2
4
6
8
10
Promoter size (kb)
B
Number of promoters
0
10
20
30
40
50
0
10
20
30
40
Number of recruited transcriptional regulators
C
Number of promoters
in individual cells
5
10
15
20
25
A
B
C
D
E
F
G
D
Uniqueness of transcriptional regulator
co-recruitment patterns
q < 0.05
A
B
C
D
E
F
G

**Appendix Figure S1. Characteristics of the mouse liver TF-encoding gene promoters and of the SOM issued from their analysis.**

**A)** Distribution of the promoters used in the SOM analysis as a function of their size. **B)** Distribution of the promoters used in the SOM analysis as a function of the number of co-bound transcriptional regulators. **C)** The SOM was used to indicate the number of independent promoters comprised within each individual cell. **D)** Cells in green are those with a unique transcriptional regulator co-recruitment pattern. This was defined as cells for which the representative transcriptional regulator co-recruitment pattern was statistically different from that of all other cells ($q<0.05$).

Fig.S2

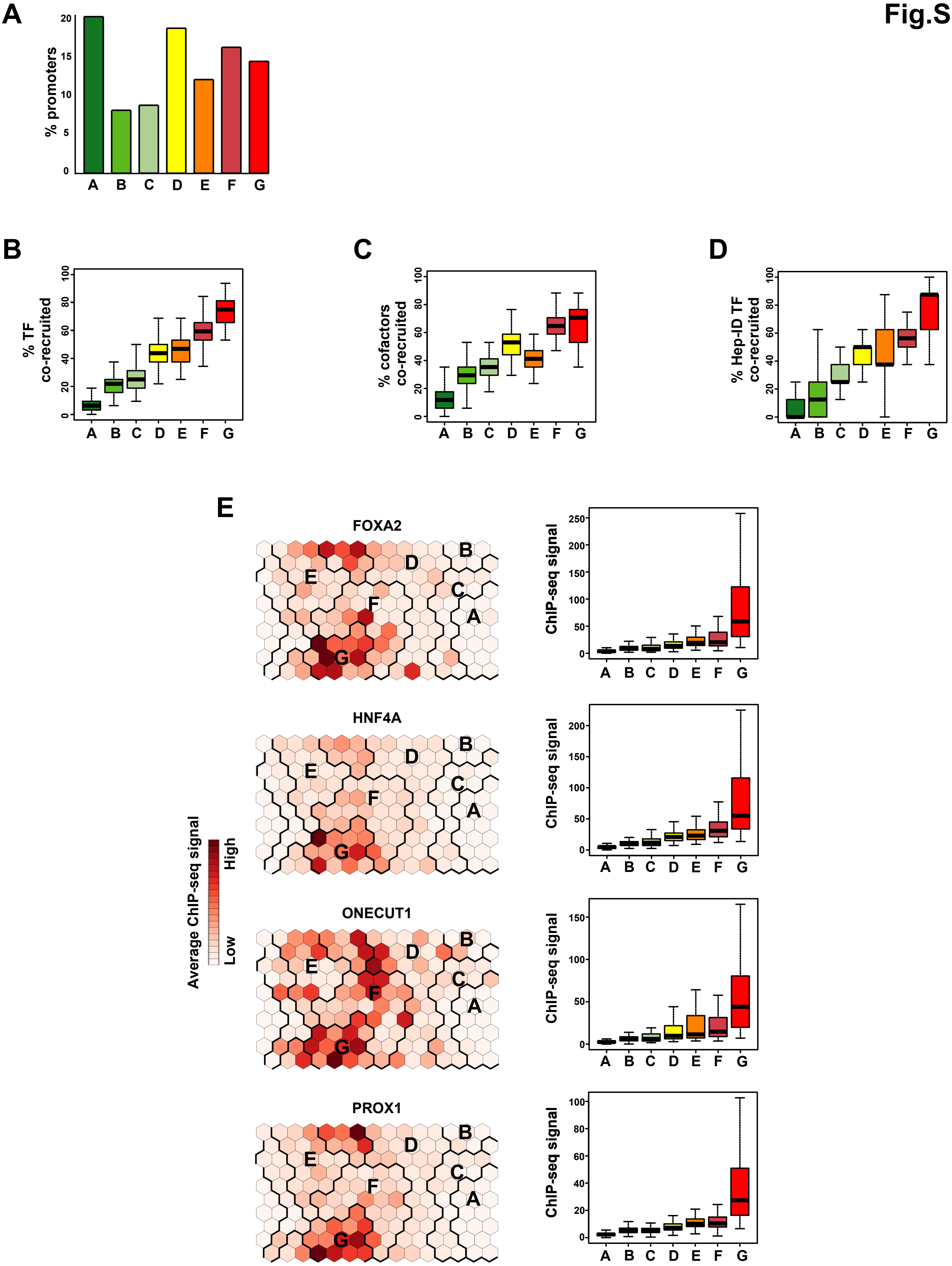
A
% promoters
20
15
10
5
0
A B C D E F G
B
% TF co-recruited
C
% cofactors co-recruited
D
% Hep-ID TF co-recruited
100
80
60
40
20
0
A B C D E F G
E
FOXA2
HNF4A
ONECUT1
PROX1
Average ChIP-seq signal
High
Low
ChIP-seq signal
250
200
150
100
50
0

**Appendix Figure S2. Characterization of the main clusters of TF-encoding gene promoters.**

**A)** Percentage of TF-encoding gene promoters analyzed retrieved in each one of clusters A-G. **B-D)** The percentage of analyzed TFs (B), cofactors (C) or Hep-ID TFs (D) co-recruited to individual promoters was monitored and box plots were used to display the data obtained for promoters belonging to clusters A-G. **E)** The map issued from Fig.1 was used to show the average ChIP-seq signal from mouse livers for the Hep-ID TFs FOXA2, HNF1A, HNF4A and ONECUT1 at promoters contained in individual cells. Bold black lines indicate the borders of clusters A-G. The data are also displayed as bar graphs on the right.

**Fig.S3**

**A**

## Cluster A

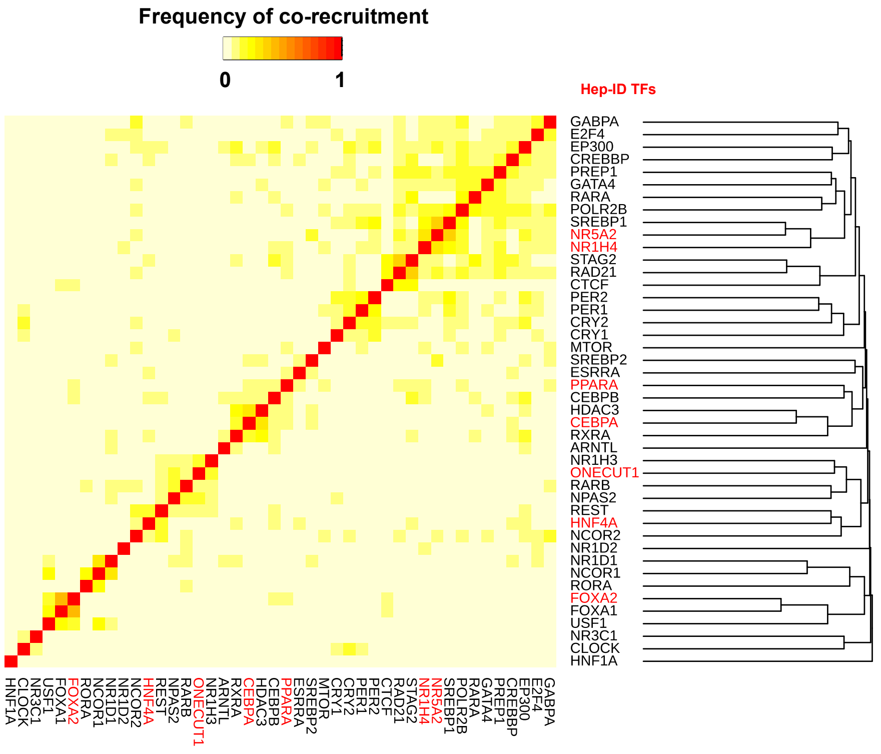


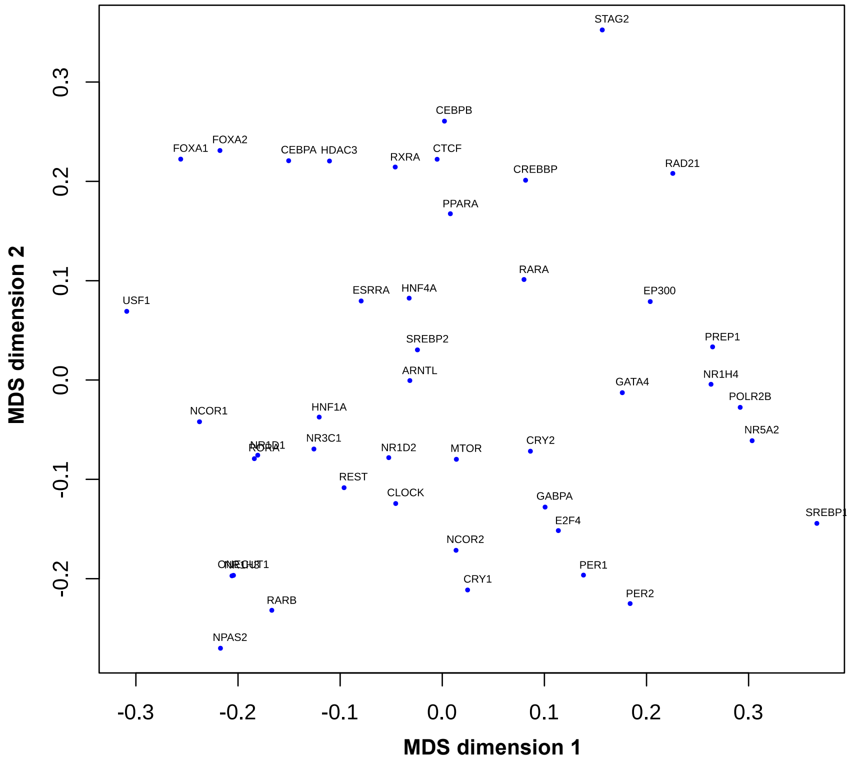

**B**

# Cluster B

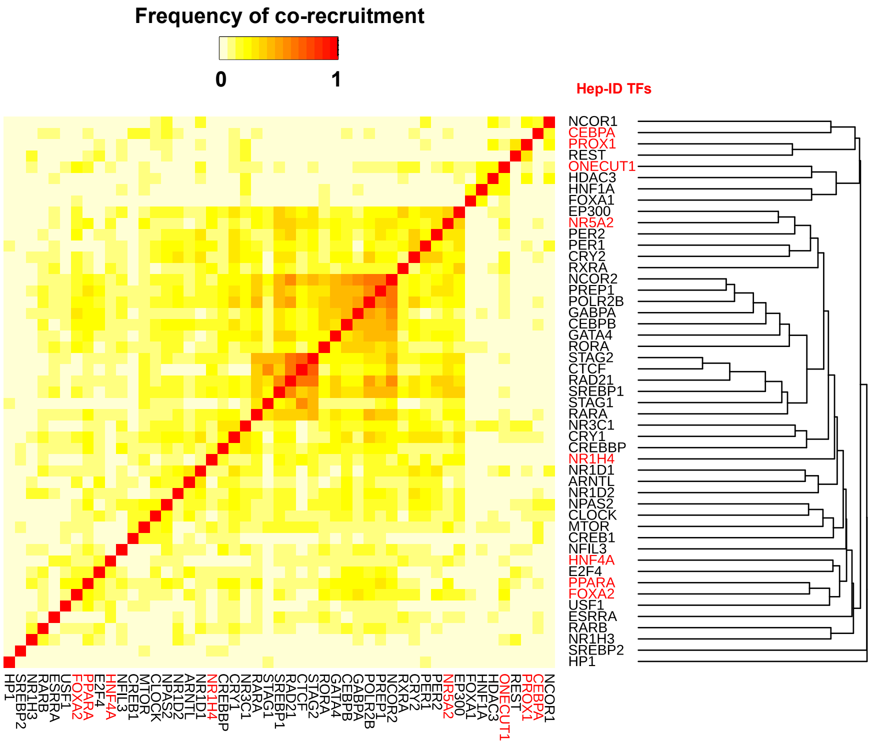
Frequency of co-recruitment
0
1
Hep-ID TFs
NCOR1
CEBPA
PROX1
REST
ONECUT1
HDAC3
HNF1A
FOXA1
EP300
NR5A2
PER2
PER1
CRY2
RXRA
NCOR2
PREP1
POLR2B
GABPA
CEBPB
GATA4
RORA
STAG2
CTCF
RAD21
SREBP1
STAG1
RARA
NR3C1
CRY1
CREBBP
NR1H4
NR1D1
ARNTL
NR1D2
NPAS2
CLOCK
MTOR
CREB1
NFIL3
HNF4A
E2F4
PPARA
FOXA2
USF1
ESRRA
RARB
NR1H3
SREBP2
HP1

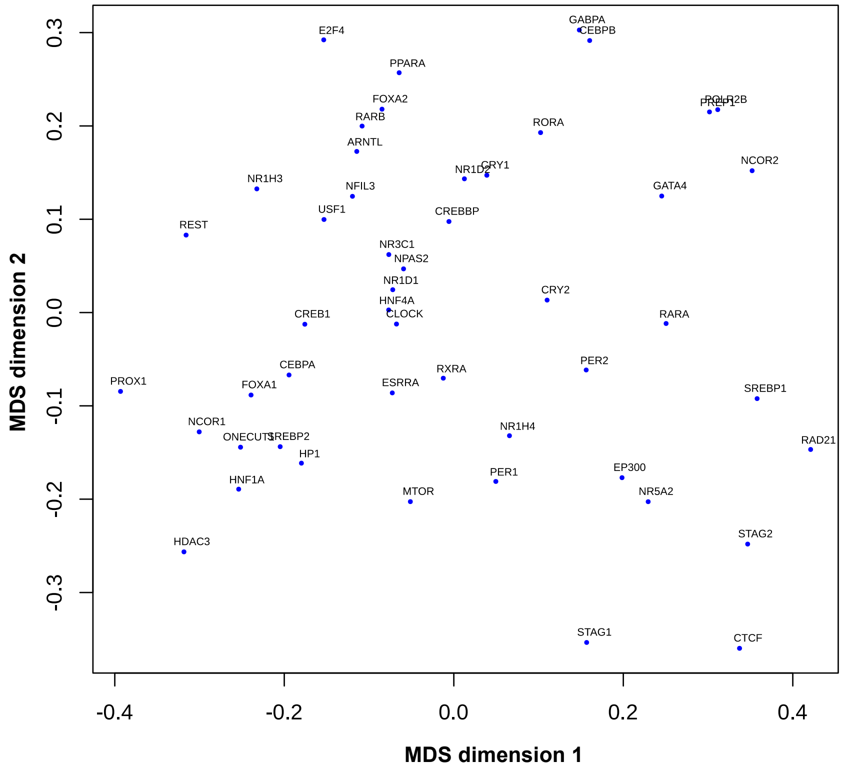
MDS dimension 1
MDS dimension 2
-0.4
-0.2
0.0
0.2
0.4
0.3
0.2
0.1
0.0
-0.1
-0.2
-0.3
GABPA
CEBPB
E2F4
PPARA
FOXA2
RARB
ARNTL
PREP1
POLR2B
RORA
NCOR2
NR1D2
CRY1
NR1H3
NFIL3
GATA4
USF1
CREBBP
REST
NR3C1
NPAS2
NR1D1
HNF4A
CLOCK
CREB1
CRY2
RARA
CEBPA
PER2
RXRA
PROX1
FOXA1
ESRRA
SREBP1
NCOR1
NR1H4
ONECUT1
SREBP2
HP1
RAD21
EP300
PER1
HNF1A
MTOR
NR5A2
HDAC3
STAG2
STAG1
CTCF

# C

## Cluster C

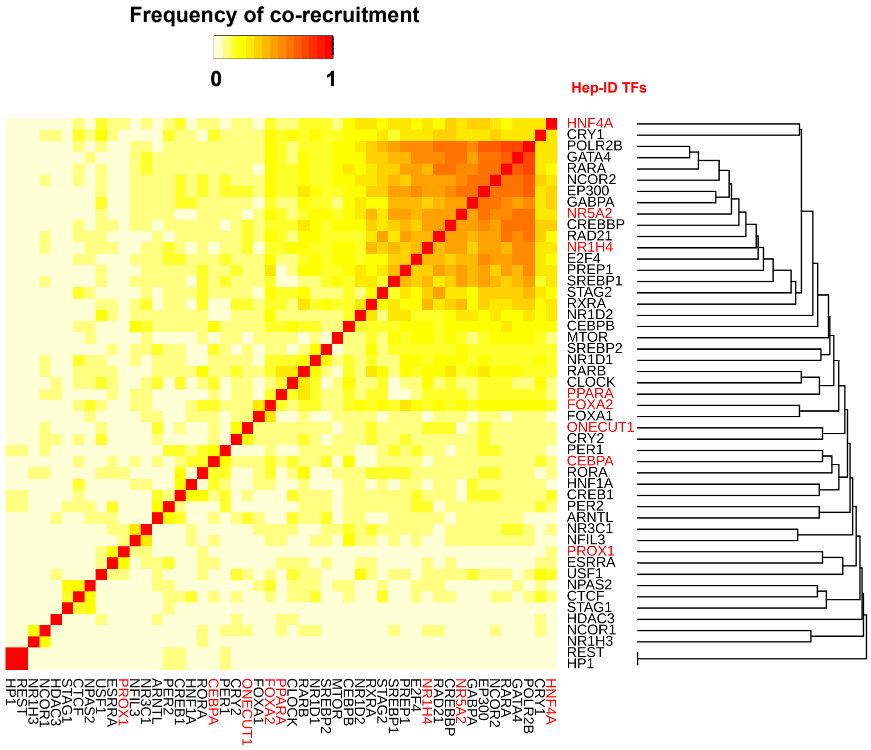
Frequency of co-recruitment
0
1
Hep-ID TFs
HNF4A
CRY1
POLR2B
GATA4
RARA
NCOR2
EP300
GABPA
NR5A2
CREBBP
RAD21
NR1H4
E2F4
PREP1
SREBP1
STAG2
RXRA
NR1D2
CEBPB
MTOR
SREBP2
NR1D1
RARB
CLOCK
PPARA
FOXA2
FOXA1
ONECUT1
CRY2
PER1
CEBPA
RORA
HNF1A
CREB1
PER2
ARNTL
NR3C1
NFIL3
PROX1
ESRRA
USF1
NPAS2
CTCF
STAG1
HDAC3
NCOR1
NR1H3
REST
HP1

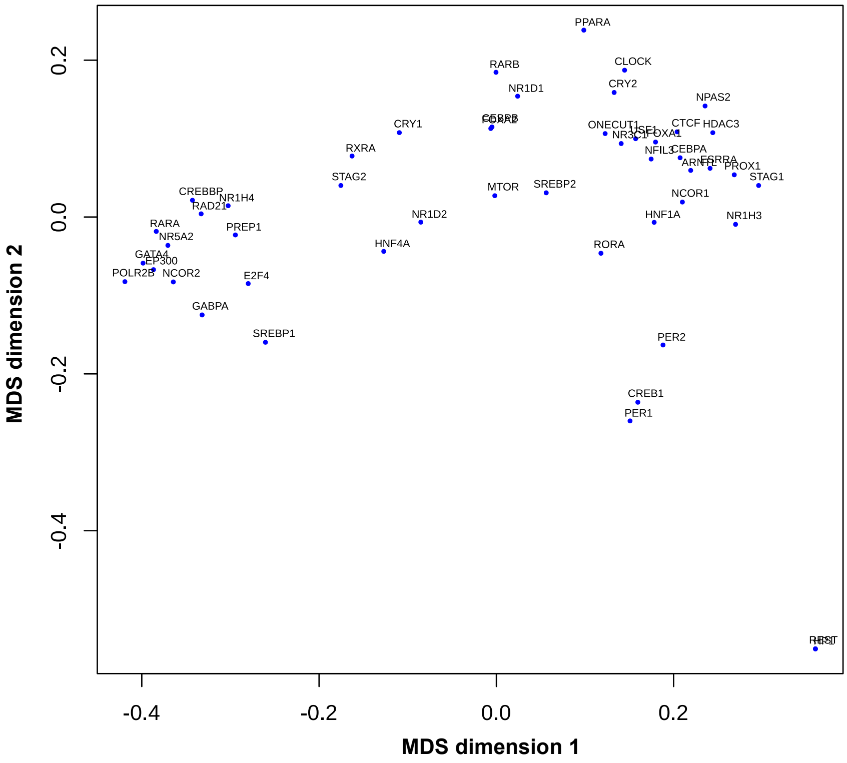
MDS dimension 2
MDS dimension 1
0.2
0.0
-0.2
-0.4
-0.4
-0.2
0.0
0.2
PPARA
RARB
CLOCK
NR1D1
CRY2
NPAS2
CRY1
ONECUT1
CTCF
HDAC3
RXRA
NFIL3
CEBPA
ESRRA
PROX1
STAG2
STAG1
CREBBP
NR1H4
MTOR
SREBP2
NCOR1
RAD21
NR1D2
HNF1A
NR1H3
RARA
PREP1
NR5A2
HNF4A
RORA
GATA4
EP300
POLR2B
NCOR2
E2F4
GABPA
SREBP1
PER2
CREB1
PER1

D

# Cluster D

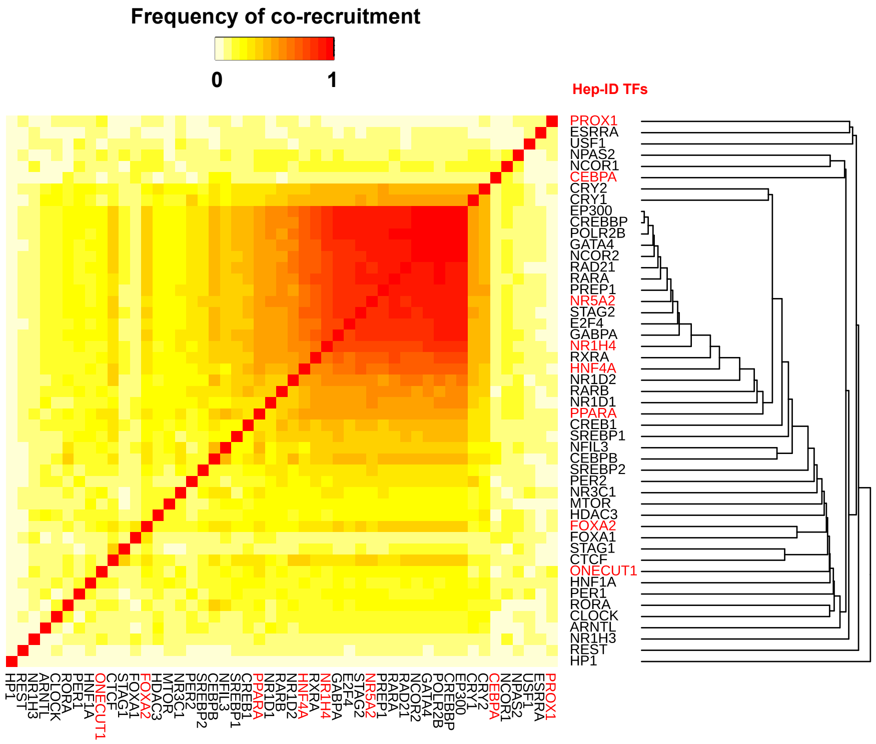
Frequency of co-recruitment
0
1
Hep-ID TFs
PROX1
ESRRA
USF1
NPAS2
NCOR1
CEBPA
CRY2
CRY1
EP300
CREBBP
POLR2B
GATA4
NCOR2
RAD21
RARA
PREP1
NR5A2
STAG2
E2F4
GABPA
NR1H4
RXRA
HNF4A
NR1D2
RARB
NR1D1
PPARA
CREB1
SREBP1
NFIL3
CEBPB
SREBP2
PER2
NR3C1
MTOR
HDAC3
FOXA2
FOXA1
STAG1
CTCF
ONECUT1
HNF1A
PER1
RORA
CLOCK
ARNTL
NR1H3
REST
HP1

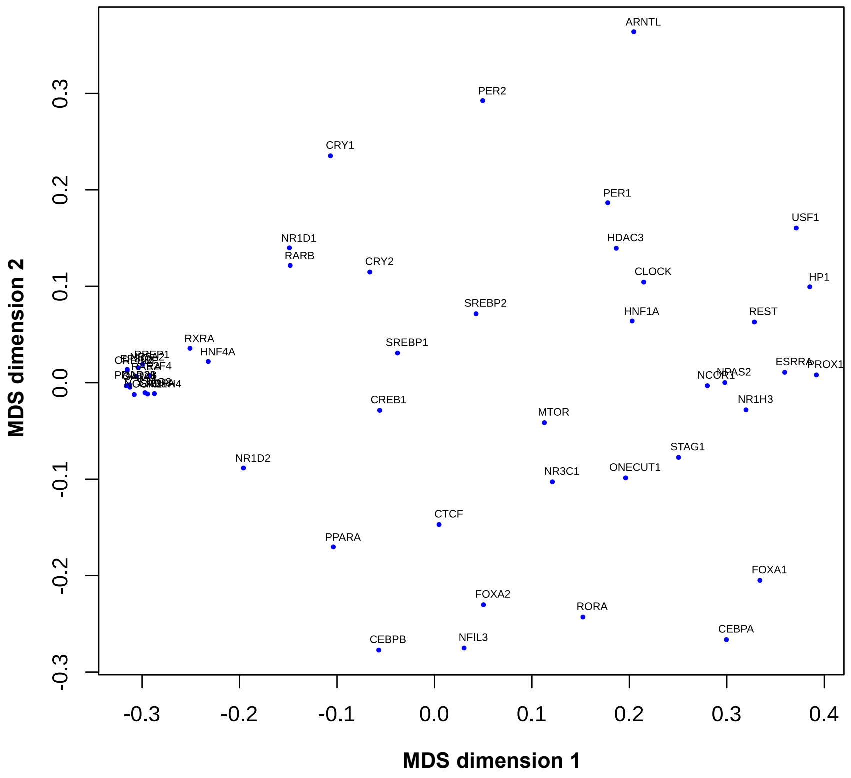
MDS dimension 1
MDS dimension 2
-0.3
-0.2
-0.1
0.0
0.1
0.2
0.3
0.4
ARNTL
PER2
CRY1
PER1
USF1
HDAC3
NR1D1
RARB
CRY2
CLOCK
HP1
SREBP2
HNF1A
REST
RXRA
HNF4A
SREBP1
ESRRA
PROX1
NPAS2
CREB1
NR1H3
MTOR
STAG1
NR1D2
NR3C1
ONECUT1
CTCF
PPARA
FOXA1
FOXA2
RORA
CEBPA
CEBPB
NFIL3

Fig.S3

E

# Cluster E

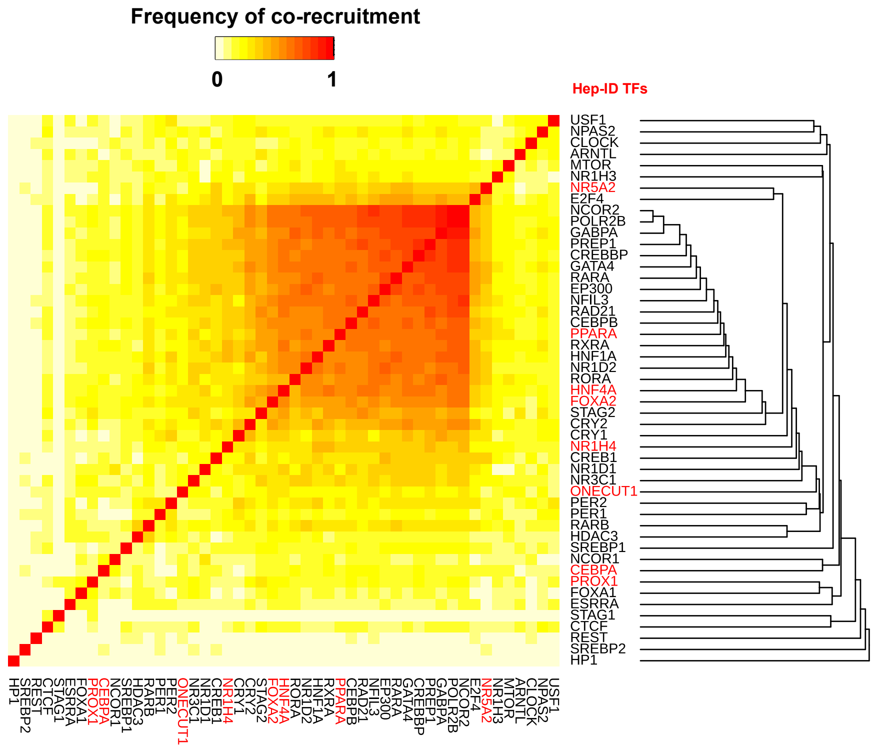


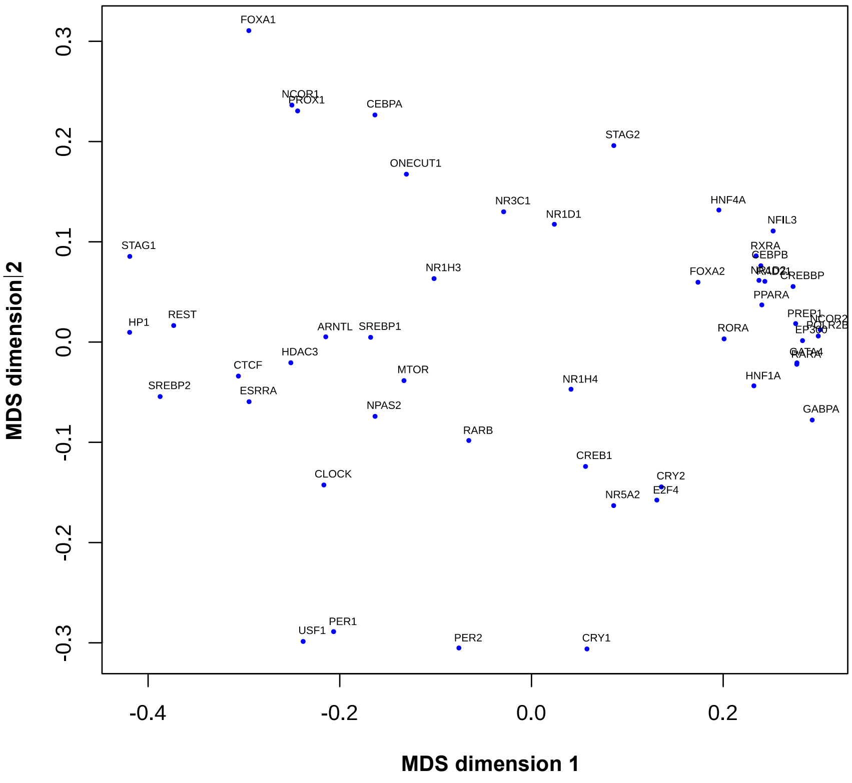

Fig.S3

F

# Cluster F

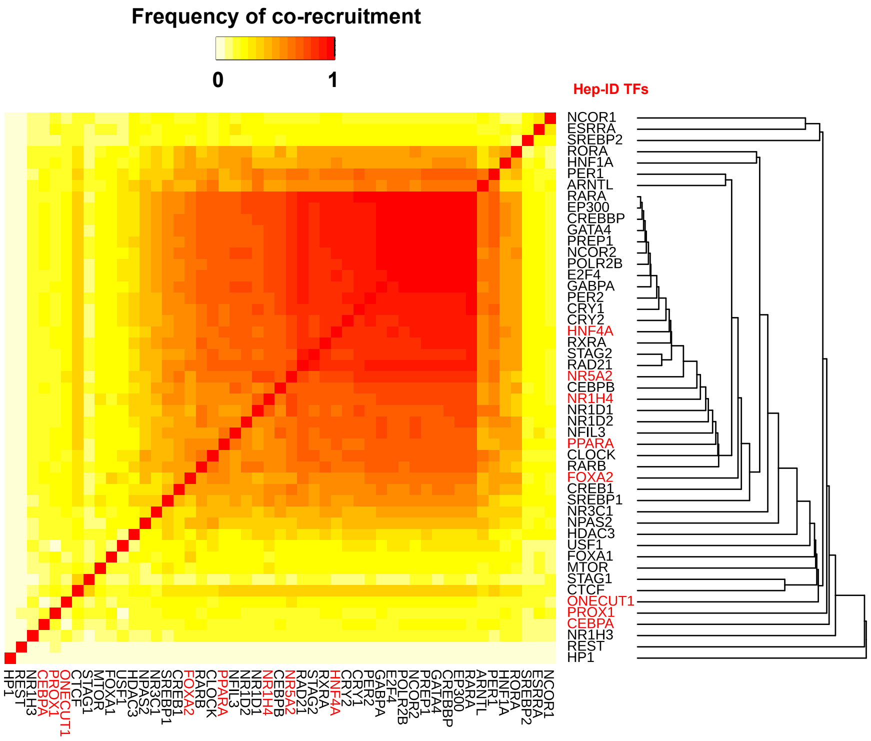


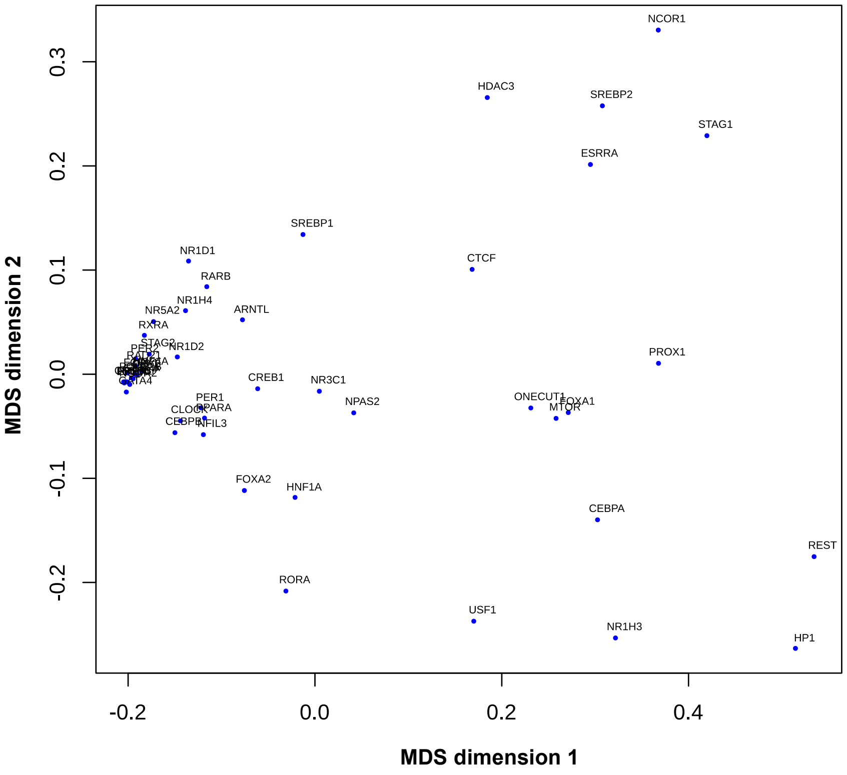

Fig.S3

G

## Cluster G

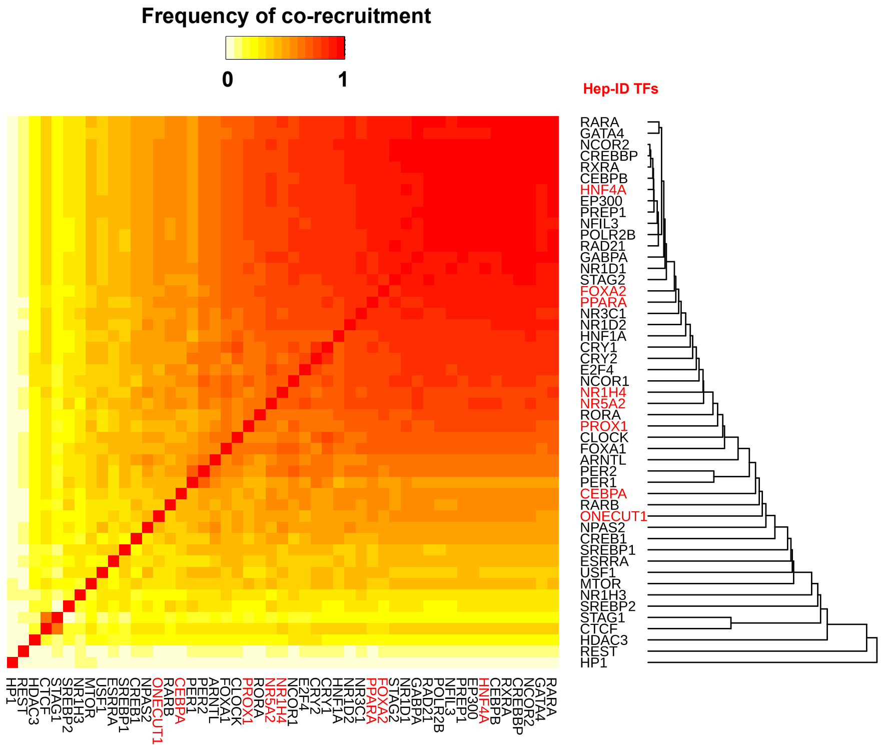


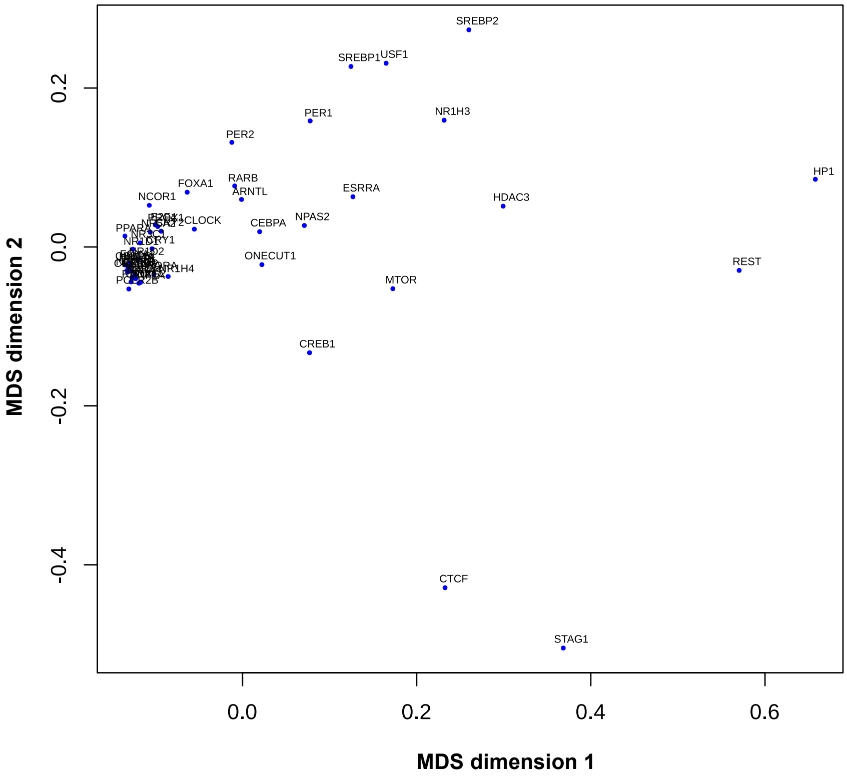

**Appendix Figure S3. Transcriptional regulator co-recruitment at promoters from clusters A-G.**

**A-G)** For each cluster of promoters, a heatmap showing transcriptional regulator co-recruitment defined using a Tanimoto index is shown (top). Transcriptional regulators were organized based on hierarchical clustering. In addition, these Tanimoto indexes were used for multidimensional scaling (MDS; see Materials and Methods) to further highlight transcriptional regulator co-recruitment patterns. Proximity in the 2D space used to display the MDS data point to co-recruitment in the set of analyzed promoters. A few TFs never bound to promoters from cluster A are omitted in panel A.

Fig.S4

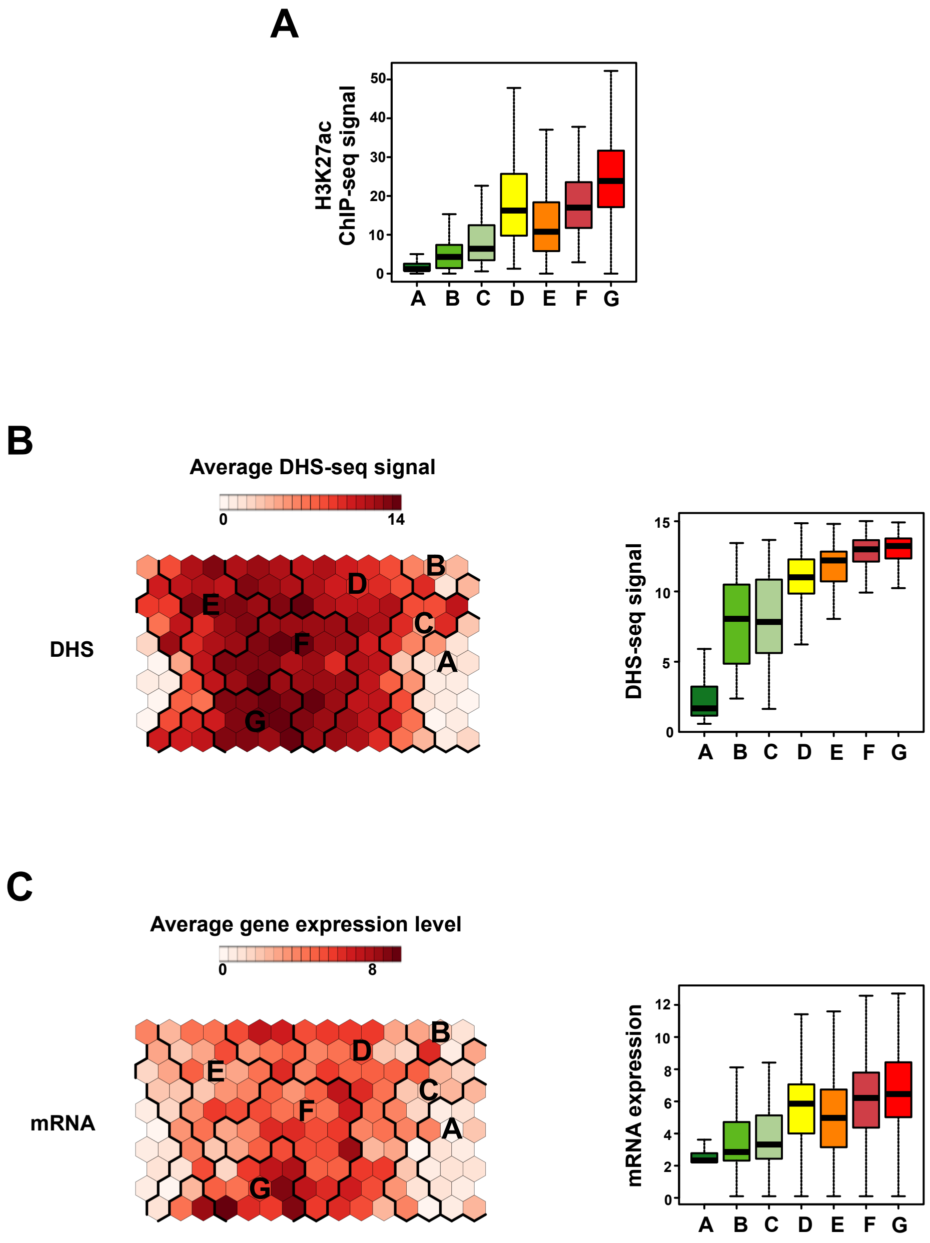

A
H3K27ac
ChIP-seq signal
0
10
20
30
40
50
A B C D E F G
B
Average DHS-seq signal
0
14
DHS
B
D
E
C
F
A
G
DHS-seq signal
0
5
10
15
A B C D E F G
C
Average gene expression level
0
8
mRNA
B
D
E
C
F
A
G
mRNA expression
0
2
4
6
8
10
12
A B C D E F G

**Appendix Figure S4. Additional characterization of the main clusters of TF-encoding gene promoters.**

**A)** Average ChIP-seq signal for mouse liver H3K27ac at promoters contained in clusters A-G.

**B-C)** The map issued from Fig.1 was used to show the average DHS-seq at promoters (B) and mRNA expression levels of the linked genes (C) from mouse livers in individual cells. Bold black lines indicate the borders of clusters A-G. The data are also displayed as bar graphs on the right.

# Fig.S5

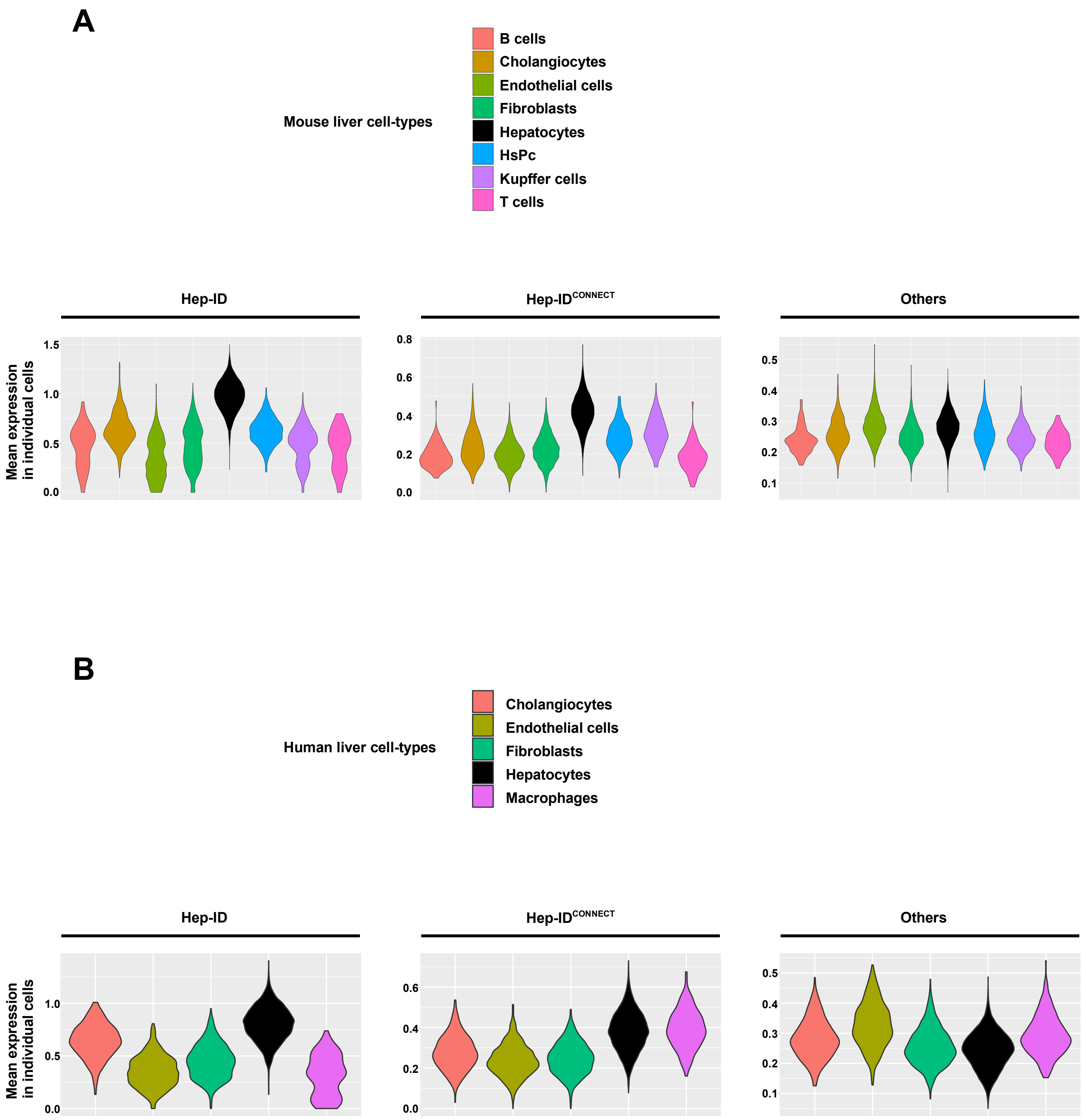
A
Mouse liver cell-types
B cells
Cholangiocytes
Endothelial cells
Fibroblasts
Hepatocytes
HsPc
Kupffer cells
T cells
Hep-ID
Hep-ID^CONNECT
Others
Mean expression in individual cells
0.0
0.5
1.0
1.5
0.0
0.2
0.4
0.6
0.8
0.1
0.2
0.3
0.4
0.5
B
Human liver cell-types
Cholangiocytes
Endothelial cells
Fibroblasts
Hepatocytes
Macrophages
Hep-ID
Hep-ID^CONNECT
Others
Mean expression in individual cells
0.0
0.5
1.0
0.0
0.2
0.4
0.6
0.1
0.2
0.3
0.4
0.5

**Appendix Figure S5. Average expression of Hep-ID and Hep-ID$^{CONNECT}$ TF encoding genes in individual mouse and human liver cell types.**

Average expression of Hep-ID, Hep-ID$^{CONNECT}$ and remaining TF-encoding genes from cluster G (Others) was monitored in single-nuclei RNA-seq data obtained from healthy adult mouse (**A**) or human (**B**) livers (Guilliams *et al*, 2022) and plotted as violin plots.

Fig.S6

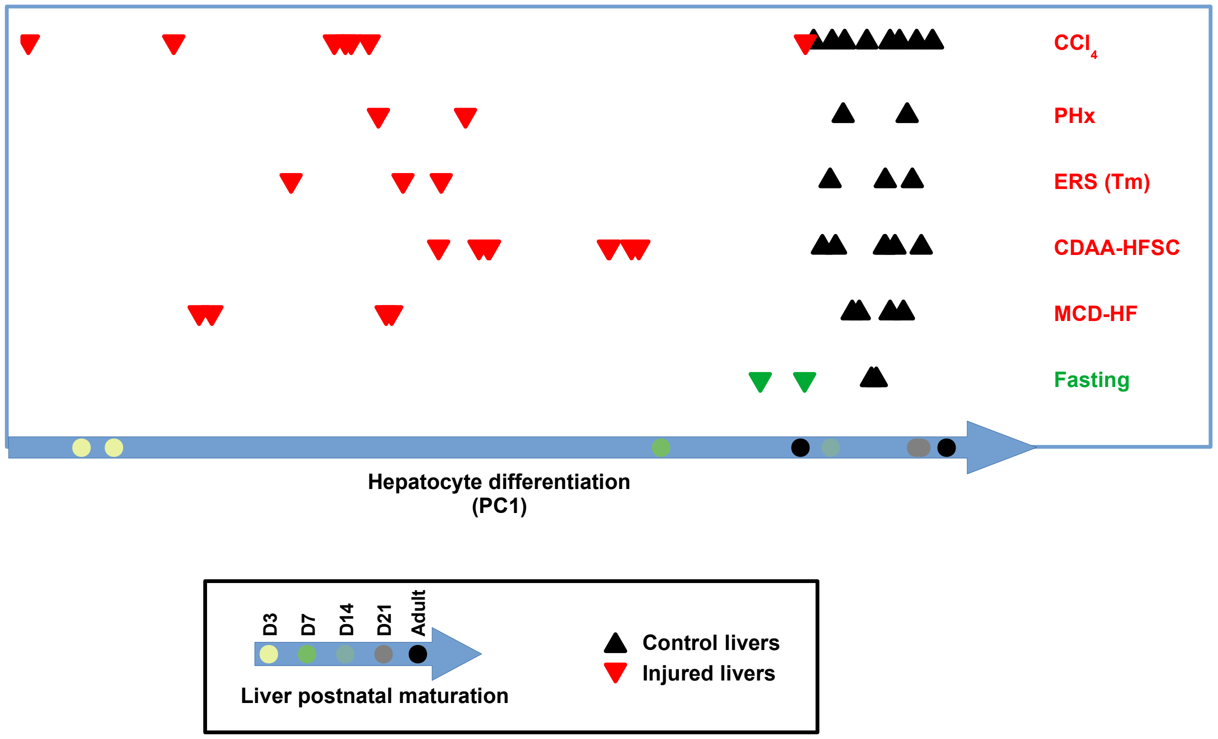

$CCl_4$
PHx
ERS (Tm)
CDAA-HFSC
MCD-HF
Fasting
Hepatocyte differentiation
(PC1)
D3
D7
D14
D21
Adult
Liver postnatal maturation
Control livers
Injured livers

**Appendix Figure S6. Loss of hepatocyte molecular identity in mouse models of severe liver injury.**

Comparison of the transcriptome of the indicated liver injury models (Table EV2) with that of the developing mouse liver performed as described in our previous study [26] and in the Materials and Methods section. PC1 is the first principal component which represents 63.55% of the variability within the mouse liver differentiation study. PC1 was used to project the liver injury studies (transcriptomic data from individual mice are shown as triangles). Transcriptomic changes induced by fasting were used as a control. $CCl_4$, carbon tetrachloride hepatotoxicity - model of drug-induced chronic liver injury; PHx, partial liver hepatectomy - model of liver resection which is a frequent clinical practice to remove liver tumors; ERS (Tm), endoplasmic reticulum stress induced by tunicamycin injection - model of drug-induced acute liver injury. CDAA-HFSC, Choline-Deficient L-amino-acid-defined diet with high fat, sucrose and cholesterol diet - model of NASH/fibrosis; MCD-HF, methionine–choline-deficient diet with high fat - model of NASH/fibrosis.

**Fig.S7**

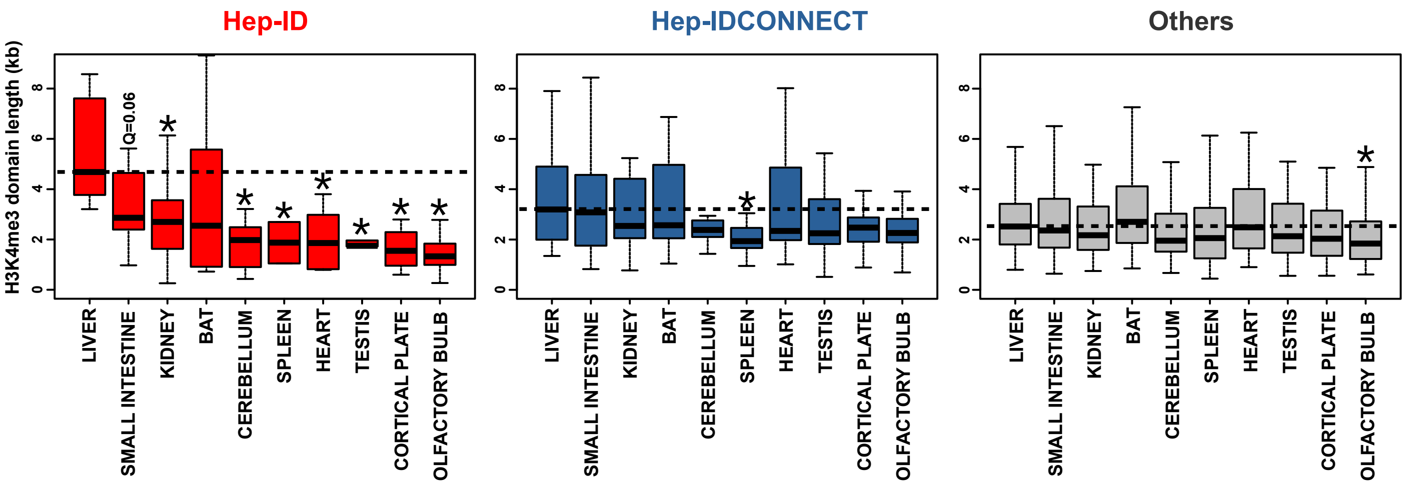
Hep-ID
Hep-IDCONNECT
Others
H3K4me3 domain length (kb)
Q=0.06
LIVER
SMALL INTESTINE
KIDNEY
BAT
CEREBELLUM
SPLEEN
HEART
TESTIS
CORTICAL PLATE
OLFACTORY BULB

**Appendix Figure S7. Length of H3K4me3 domains at the promoter of TF-encoding genes in various mouse tissues/organs.**

Uniformly processed H3K4me3 ChIP-seq data from various mouse tissues/organs were used to monitor the length of the H3K4me3 domains overlapping the TSS of Hep-ID, Hep-ID$^{CONNECT}$ and remaining TF-encoding genes from cluster G (Others). Dotted lines show the median length in the liver. Statistical difference between liver and other organs was defined using Kruskal-Wallis with Wilcoxon pairwise comparison tests followed by Benjamini-Hochberg correction for multiple testing correction. *q<0.05.

Fig.S8

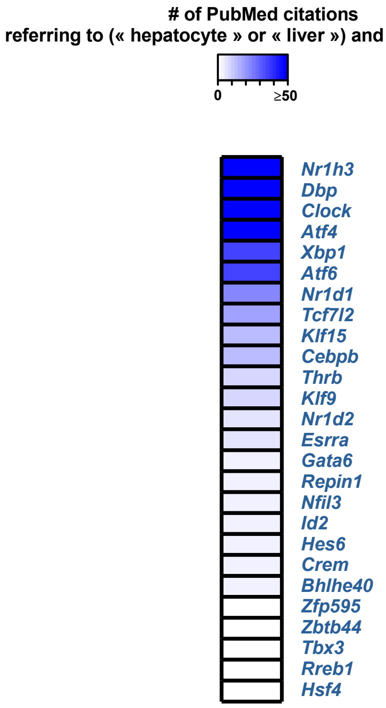
# of PubMed citations
referring to (« hepatocyte » or « liver ») and « metabolism »
0
≥50
Nr1h3
Dbp
Clock
Atf4
Xbp1
Atf6
Nr1d1
Tcf7l2
Klf15
Cebpb
Thrb
Klf9
Nr1d2
Esrra
Gata6
Repin1
Nfil3
Id2
Hes6
Crem
Bhlhe40
Zfp595
Zbtb44
Tbx3
Rreb1
Hsf4

**Appendix Figure S8. Association between Hep-ID$^{CONNECT}$ TFs and liver/hepatocyte metabolism in the scientific literature.**

The heatmap displays the numbers of PubMed-referenced articles referring to individual Hep-ID$^{CONNECT}$ TFs and ("liver" or "hepatocyte") and "metabolism".

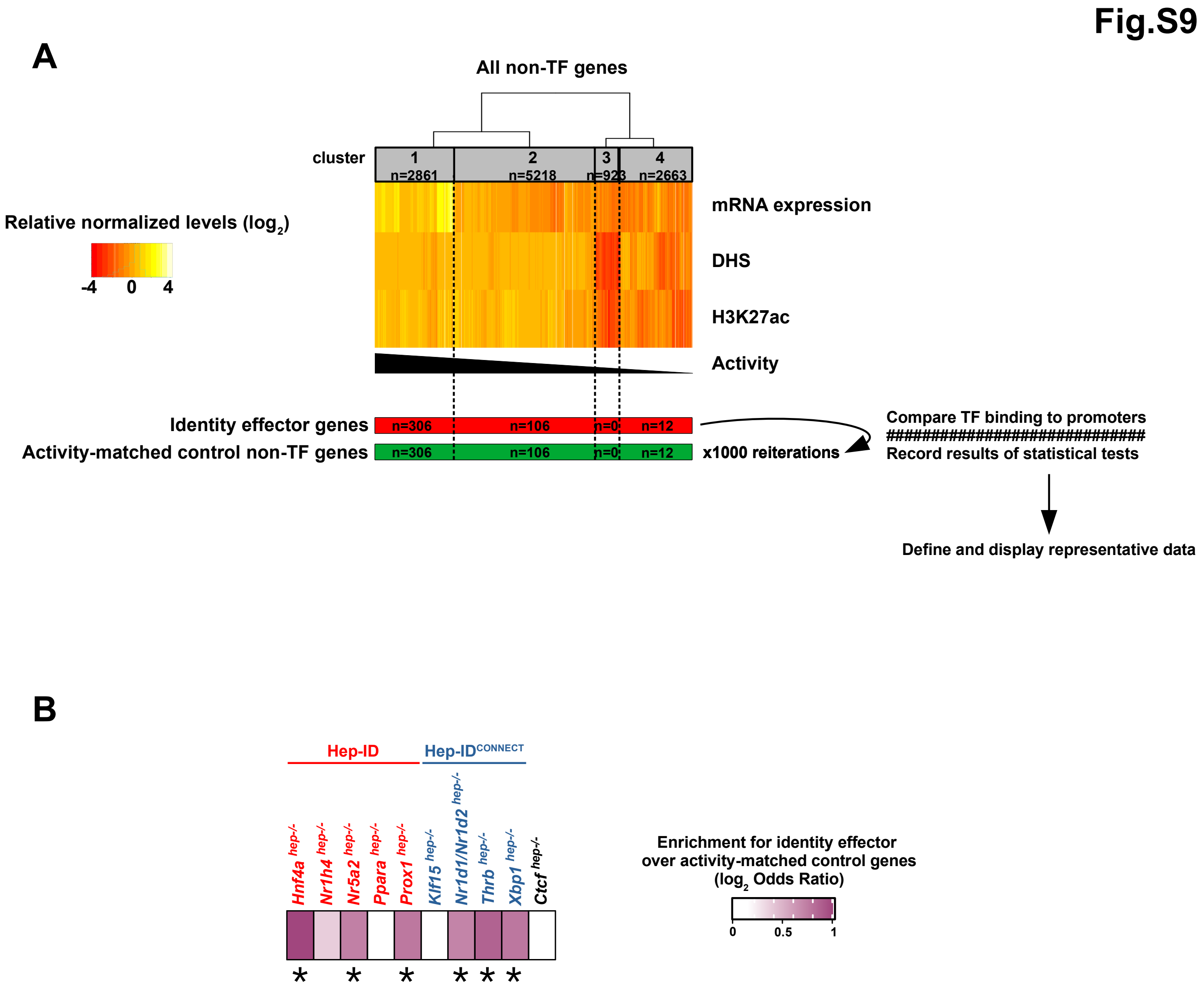
Fig.S9
A
All non-TF genes
cluster
1 n=2861
2 n=5218
3 n=923
4 n=2663
Relative normalized levels (log2)
-4 0 4
mRNA expression
DHS
H3K27ac
Activity
Identity effector genes
Activity-matched control non-TF genes
n=306
n=106
n=0
n=12
x1000 reiterations
Compare TF binding to promoters
Record results of statistical tests
Define and display representative data
B
Hep-ID
Hep-ID CONNECT
Hnf4a hep-/-
Nr1h4 hep-/-
Nr5a2 hep-/-
Ppara hep-/-
Prox1 hep-/-
Klf15 hep-/-
Nr1d1/Nr1d2 hep-/-
Thrb hep-/-
Xbp1 hep-/-
Ctcf hep-/-
Enrichment for identity effector
over activity-matched control genes
(log2 Odds Ratio)
0 0.5 1

**Appendix Figure S9. Strategy used to compare TF binding to the promoters of identity effector genes and activity-matched control non-TF gene promoters.**

**A)** Non-TF-encoding gene promoters were clustered based on their activity in the mouse liver defined through mRNA expression levels of associated genes and DHS-seq and H3K27ac ChIP-seq signals. The hierarchical clustering tree is shown on top of the heatmap together with identification of 4 main clusters. The activity-matched control set was obtained by randomly picking within each of the 4 clusters a number of genes equivalent to that of identity effector genes. TF binding to the promoters of identity effector and activity-matched control genes was defined by mining ChIP-seq signal intensity and statistical difference was tested using Wilcoxon Rank Sum Tests. This analysis was reiterated 1000 times and the mode of the p-value distribution was used to select a representative control gene set used in Fig.2I. **B)** Analyses similar to those in Fig.2H but using as control an equivalent number of activity-matched non-TF-encoding genes, which gives data representative of those obtained using 1000 reiterations of the analysis as described hereabove.

Fig.S10

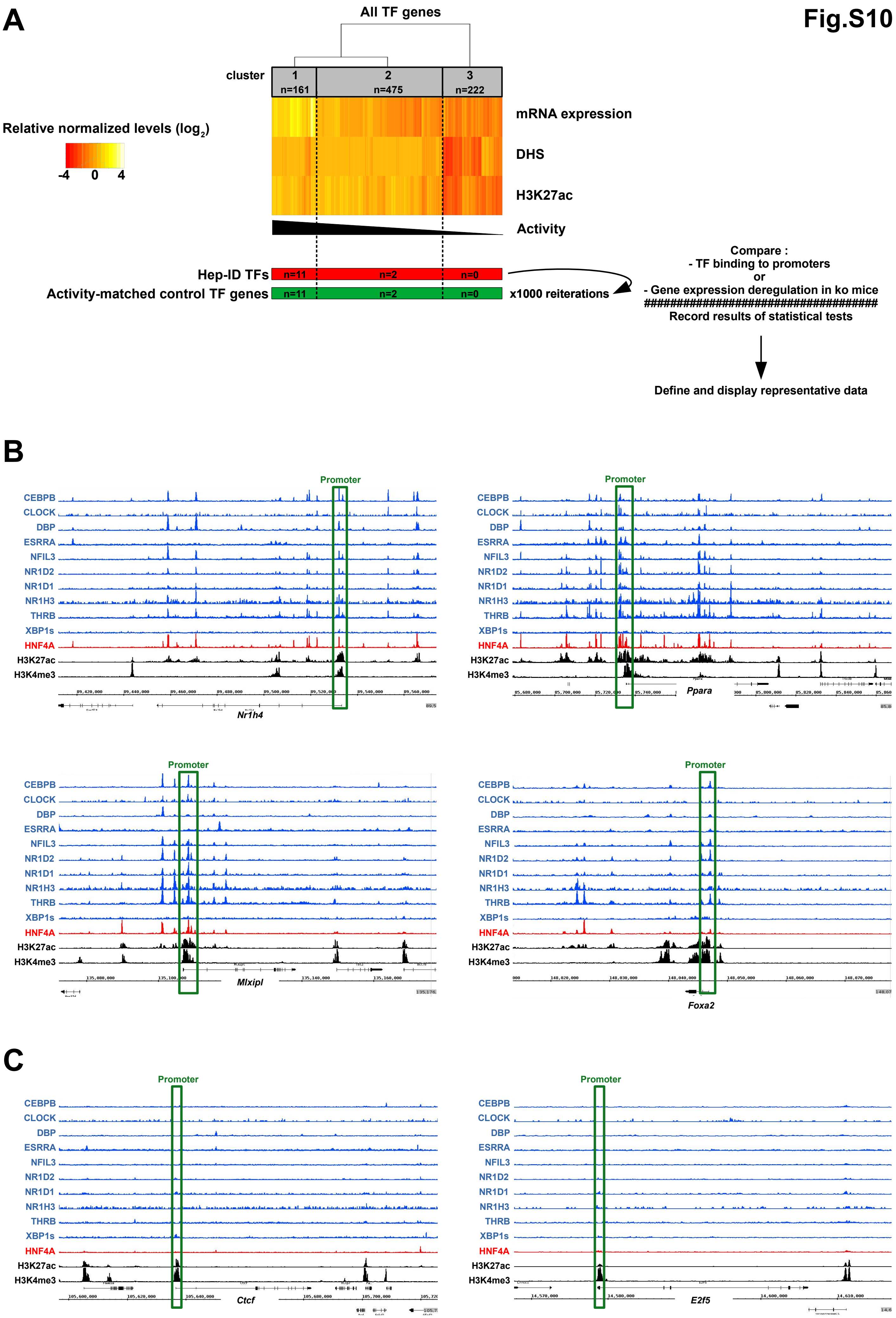
A
All TF genes
cluster
1
n=161
2
n=475
3
n=222
Relative normalized levels (log2)
-4
0
4
mRNA expression
DHS
H3K27ac
Activity
Hep-ID TFs
n=11
n=2
n=0
Activity-matched control TF genes
n=11
n=2
n=0
x1000 reiterations
Compare :
- TF binding to promoters
or
- Gene expression deregulation in ko mice
####################################
Record results of statistical tests
Define and display representative data
B
Promoter
CEBPB
CLOCK
DBP
ESRRA
NFIL3
NR1D2
NR1D1
NR1H3
THRB
XBP1s
HNF4A
H3K27ac
H3K4me3
Nr1h4
Ppara
Mlxipl
Foxa2
C
Ctcf
E2f5

**Appendix Figure S10. Strategy used to compare TF binding to or transcriptional regulation of Hep-ID TFs and activity-matched control TF genes.**

**A)** TF-encoding gene promoters were clustered based on their activity in the mouse liver defined through mRNA expression levels of associated genes and DHS-seq and H3K27ac ChIP-seq signals. The hierarchical clustering tree is shown on top of the heatmap together with identification of 3 main clusters. Hep-ID and non-Hep-ID (Other) TF gene promoters among the most active ones (cluster 1) were split and compared as promoters matched for high activity in subsequent analyses. **B-C)** The Integrated Genome Browser (IGB) was used to display the cistromes of the indicated Hep-ID$^{CONNECT}$ TFs (blue) and the Hep-ID TF HNF4A (red) together with levels of H3K4me3 and H3K27ac (black) from mouse liver ChIP-seq data (Table EV2). Example Hep-ID TF (**B**) and control TF-encoding genes (**C**) are shown. The promoters are highlighted by green boxes. The scales of the individual ChIP-seq tracks were kept constant for all analyzed genes.

**Fig.S11**

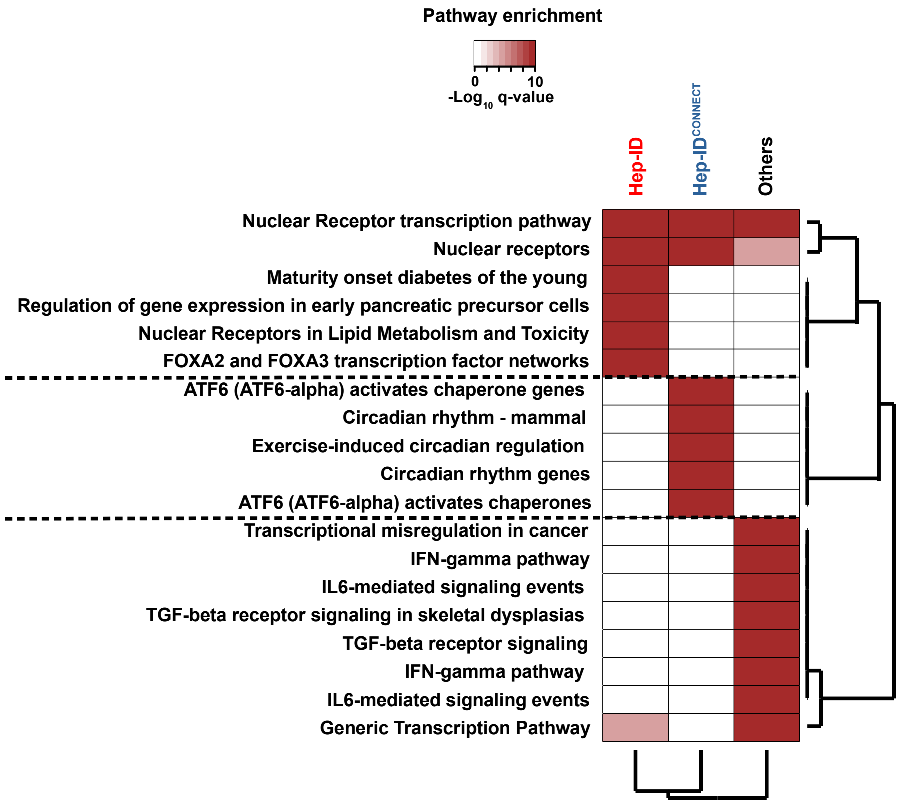
Pathway enrichment
0
10
-Log10 q-value
Hep-ID
Hep-ID CONNECT
Others
Nuclear Receptor transcription pathway
Nuclear receptors
Maturity onset diabetes of the young
Regulation of gene expression in early pancreatic precursor cells
Nuclear Receptors in Lipid Metabolism and Toxicity
FOXA2 and FOXA3 transcription factor networks
ATF6 (ATF6-alpha) activates chaperone genes
Circadian rhythm - mammal
Exercise-induced circadian regulation
Circadian rhythm genes
ATF6 (ATF6-alpha) activates chaperones
Transcriptional misregulation in cancer
IFN-gamma pathway
IL6-mediated signaling events
TGF-beta receptor signaling in skeletal dysplasias
TGF-beta receptor signaling
IFN-gamma pathway
IL6-mediated signaling events
Generic Transcription Pathway

**Appendix Figure S11. Biological pathways linked to Hep-ID, Hep-ID$^{CONNECT}$ and remaining TF-encoding genes from cluster G (Others).**

Biological pathways associated with Hep-ID, Hep-ID$^{CONNECT}$ and remaining TF-encoding genes from cluster G (Others) were defined using ToppCluster. When several pathways had an identical name, only the one with the greatest q-value was used. Dendrograms of hierarchical clustering are shown. ToppCluster uses hypergeometric tests and Bonferroni correction.

Fig.S12

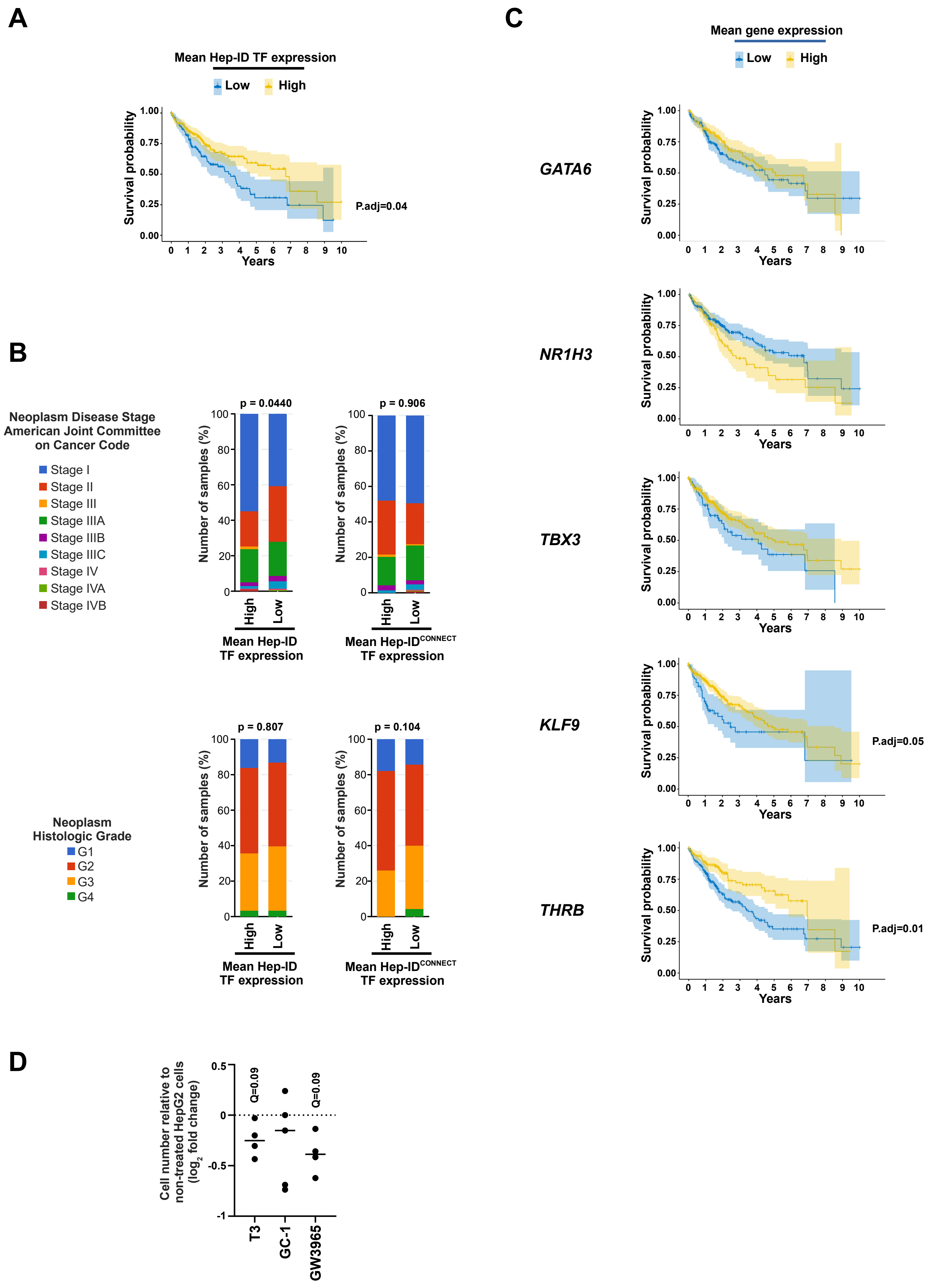
A
Mean Hep-ID TF expression
Low
High
Survival probability
1.00
0.75
0.50
0.25
0.00
0 1 2 3 4 5 6 7 8 9 10
Years
P.adj=0.04
B
Neoplasm Disease Stage American Joint Committee on Cancer Code
Stage I
Stage II
Stage III
Stage IIIA
Stage IIIB
Stage IIIC
Stage IV
Stage IVA
Stage IVB
p = 0.0440
p = 0.906
Number of samples (%)
100
80
60
40
20
0
High
Low
Mean Hep-ID TF expression
Mean Hep-ID^CONNECT TF expression
Neoplasm Histologic Grade
G1
G2
G3
G4
p = 0.807
p = 0.104
C
Mean gene expression
Low
High
GATA6
NR1H3
TBX3
KLF9
P.adj=0.05
THRB
P.adj=0.01
D
Cell number relative to non-treated HepG2 cells (log2 fold change)
0.5
0
-0.5
-1
Q=0.09
Q=0.09
T3
GC-1
GW3965

**Appendix Figure S12. Clinical features associated with HCC displaying low or high expression levels of Hep-ID$^{CONNECT}$ TF genes.**

**A)** Overall survival of patients with HCC expressing low or high levels of the Hep-ID TF encoding genes. Differential overall survival analysis was assessed by Kaplan-Meier (KM) log rank adjusted for 100 permutations (Cheng *et al*, 2022). **B)** Distribution of the stages or grades of HCC with low or high average expression levels of Hep-ID or Hep-ID$^{CONNECT}$ TF genes. Chi-squared tests were used to assess statistical significance. **C)** Similar analyses to that described in panel A performed using individual Hep-ID$^{CONNECT}$ TF encoding genes. Overall, results were less significant here indicating that mining Hep-ID and Hep-ID$^{CONNECT}$ TF genes as gene sets provided robustness to this type of analysis. Nevertheless, high THRB expression was the most significantly associated with greater 10-year overall survival. Note that lack of similar findings for NR1H3 may stem from the role exerted by NR1H3 not only in tumor cells but also in cells of the tumor microenvironment such as macrophages (Zhou *et al*. 2022). **D)** HepG2 cells were treated with T3, GC-1 or GW3965 for 7 days. Viable cell number was determined (based on 3 technical replicates) and expressed relative to that obtained for control conditions (untreated or exposed to DMSO). Data are shown as scatter plot with dots displaying the results obtained from independent biological replicates and the horizontal lines showing the median. One-sample t-test with Benjamini-Hochberg correction for multiple testing was used to determine if the mean log2 FC was statistically different from 0.

Fig.S13

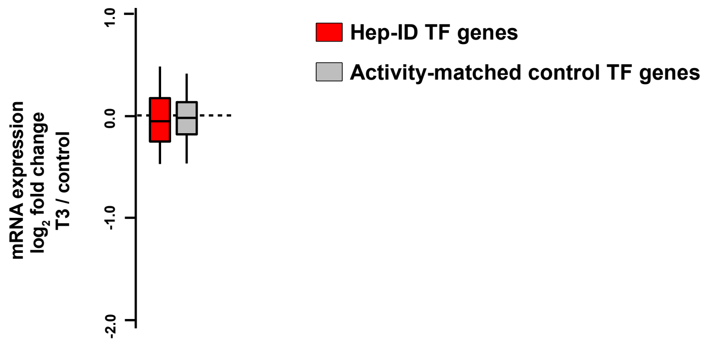

mRNA expression
log2 fold change
T3 / control
1.0
0.0
-1.0
-2.0
Hep-ID TF genes
Activity-matched control TF genes

**Appendix Figure S13. T3-mediated transcriptional regulation of Hep-ID TF genes in livers of healthy mice.**

Modulation of Hep-ID TF gene expression in the mouse liver upon T3 injection for 3 days. Analyses were performed as in Fig.4J.

Fig.S14

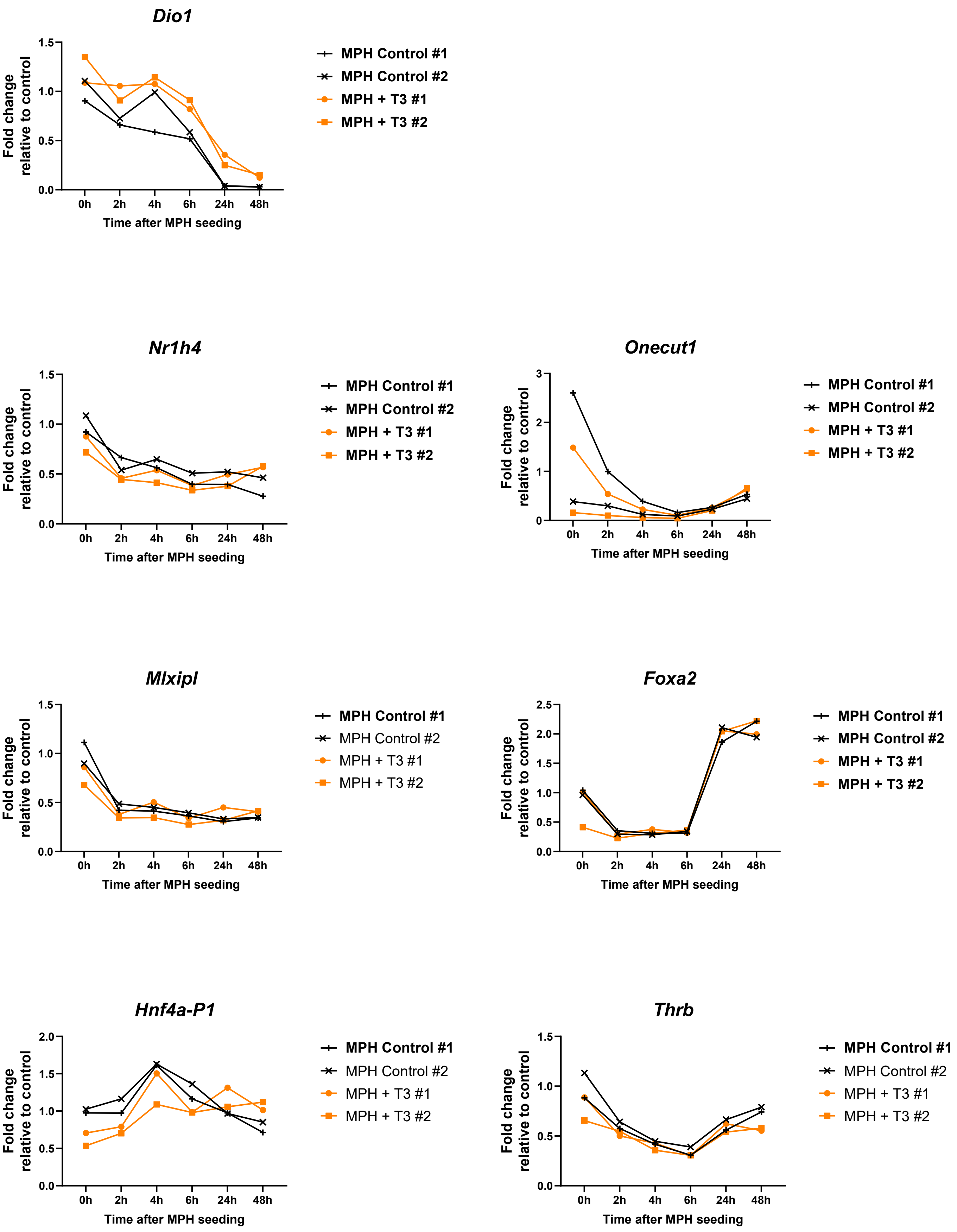

Dio1
Fold change relative to control
Time after MPH seeding
0h
2h
4h
6h
24h
48h
MPH Control #1
MPH Control #2
MPH + T3 #1
MPH + T3 #2
Nr1h4
Onecut1
Mlxipl
Foxa2
Hnf4a-P1
Thrb

**Appendix Figure S14. T3-mediated transcriptional regulation of Hep-ID TF genes in MPH.**

MPH were isolated in the presence or not of T3 (n=2 independent MPH preparation per condition), seeded in cell culture plates and harvested at the indicated times. mRNA expression of the indicated genes was monitored using RT-qPCR. The graphs show how gene expression evolved with time in the different MPH preparations (average expression in non-treated MPH at 0h was set to 1).
