# Supplementary figures and images for: An extended transcription factor regulatory network controls hepatocyte identity
Source: EMBO Rep. 2023 Jul 10;24(9):e57020. doi: 10.15252/embr.202357020 (PMC10481658; doi:10.15252/embr.202357020)

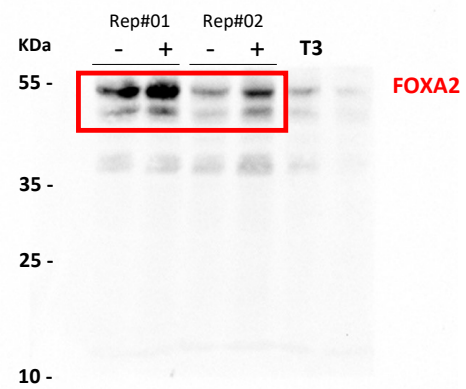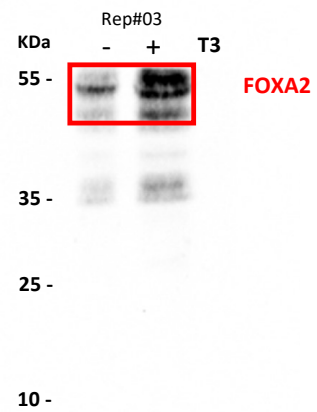

Supplement: Supplementary file 13 — Source Data for Figure 4 [file EMBR-24-e57020-s012.zip › Figure4/4I/western_FOXA2.pdf]

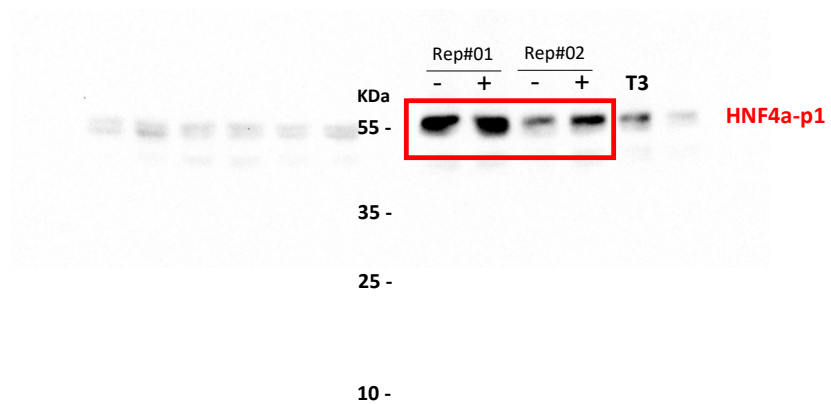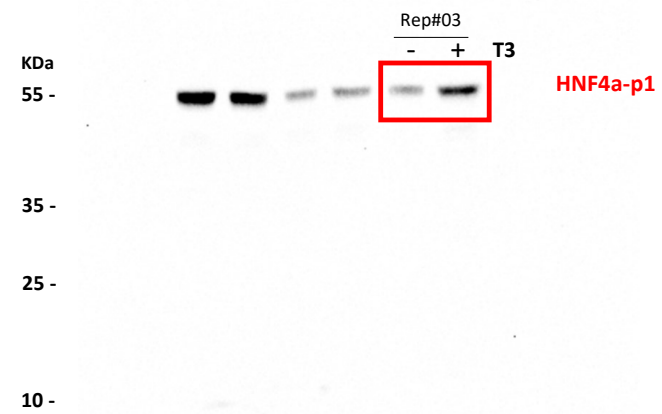

Supplement: Supplementary file 13 — Source Data for Figure 4 [file EMBR-24-e57020-s012.zip › Figure4/4I/western_HNF4A.pdf]

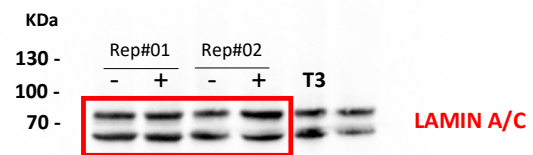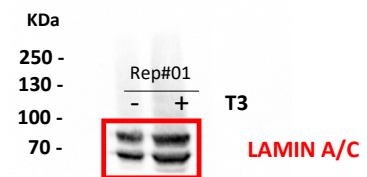

Supplement: Supplementary file 13 — Source Data for Figure 4 [file EMBR-24-e57020-s012.zip › Figure4/4I/western_LaminAC.pdf]

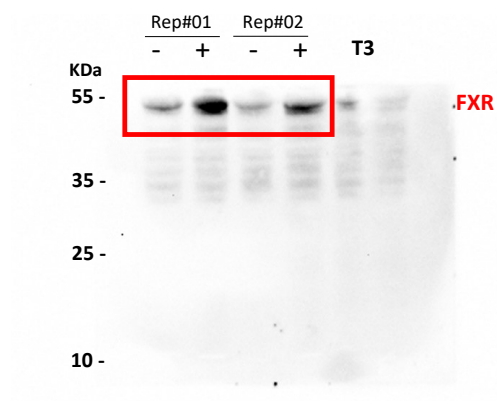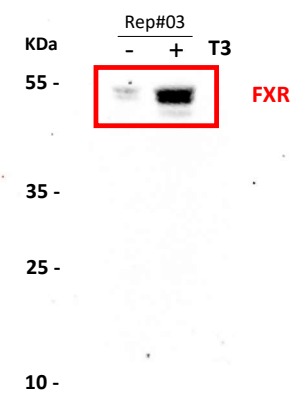

Supplement: Supplementary file 13 — Source Data for Figure 4 [file EMBR-24-e57020-s012.zip › Figure4/4I/western_NR1H4.pdf]
