# Supplementary material for: An extended transcription factor regulatory network controls hepatocyte identity
Source: EMBO Rep. 2023 Jul 10;24(9):e57020. doi: 10.15252/embr.202357020 (PMC10481658; doi:10.15252/embr.202357020)
Supplement: Supplementary file 15 [file EMBR-24-e57020-s006.pdf]

EMBO reports

An extended transcription factor regulatory network controls hepatocyte identity

Julie Dubois-Chevalier, Céline Gheeraert, Alexandre Berthier, Clémence Boulet, Vanessa Dubois, Loïc Guille, Marie Fourcot, Guillemette Marot, Karine Gauthier, Laurent Dubuquoy, Bart Staels, Philippe Lefebvre, and Jerome Eeckhoute
DOI: 10.15252/embr.202357020

Corresponding author(s): Jerome Eeckhoute (jerome.eeckhoute@inserm.fr)

Review Timeline:

Submission Date:	16th Feb 23
Editorial Decision:	3rd Apr 23
Revision Received:	17th May 23
Editorial Decision:	15th Jun 23
Revision Received:	16th Jun 23
Accepted:	21st Jun 23

Editor: Esther Schnapp

Transaction Report:

(Note: With the exception of the correction of typographical or spelling errors that could be a source of ambiguity, letters and reports are not edited. Depending on transfer agreements, referee reports obtained elsewhere may or may not be included in this compilation. Referee reports are anonymous unless the Referee chooses to sign their reports.)

1st Editorial Decision **3rd Apr 2023**

Dear Dr. Eeckhoute,

Thank you for the submission of your manuscript to EMBO reports. We have now received the comments from referees 2 and 3, which are pasted below. Referee 1 has unfortunately not submitted a report, despite several reminders.

As you will see, the referees acknowledge that the findings are potentially interesting. However, they also raise several concerns, especially referee 3, that should be addressed. Please let me know if you have any questions or comments regarding the revisions, and we can discuss this further.

I would thus like to invite you to revise your manuscript with the understanding that the referee concerns must be fully addressed and their suggestions taken on board. Please address all referee concerns in a complete point-by-point response. Acceptance of the manuscript will depend on a positive outcome of a second round of review. It is EMBO reports policy to allow a single round of major revision only and acceptance or rejection of the manuscript will therefore depend on the completeness of your responses included in the next, final version of the manuscript.

We realize that it is difficult to revise to a specific deadline. In the interest of protecting the conceptual advance provided by the work, we recommend a revision within 3 months (4th Jul 2023). Please discuss the revision progress ahead of this time with the editor if you require more time to complete the revisions.

You can either publish the study as a short report or as a full article. For short reports, the revised manuscript should not exceed 27,000 characters (including spaces but excluding materials & methods and references) and 5 main plus 5 expanded view figures. The results and discussion sections must further be combined, which will help to shorten the manuscript text by eliminating some redundancy that is inevitable when discussing the same experiments twice. For a normal article there are no length limitations, but it should have more than 5 main figures and the results and discussion sections must be separate. In both cases, the entire materials and methods must be included in the main manuscript file.

IMPORTANT NOTE: we perform an initial quality control of all revised manuscripts before re-review. Your manuscript will FAIL this control and the handling will be DELAYED if the following APPLIES:
1) A data availability section providing access to data deposited in public databases is missing. If you have not deposited any data, please add a sentence to the data availability section that explains that.
2) Your manuscript contains statistics and error bars based on n=2. Please use scatter blots in these cases. No statistics should be calculated if n=2.

When submitting your revised manuscript, please carefully review the instructions that follow below. Failure to include requested items will delay the evaluation of your revision.

1) a .docx formatted version of the manuscript text (including legends for main figures, EV figures and tables). Please make sure that the changes are highlighted to be clearly visible.

2) individual production quality figure files as .eps, .tif, .jpg (one file per figure). See https://wol-prod-cdn.literatumonline.com/pb-assets/embo-site/EMBOPress_Figure_Guidelines_061115-1561436025777.pdf for more info on how to prepare your figures.

3) We replaced Supplementary Information with Expanded View (EV) Figures and Tables that are collapsible/expandable online. A maximum of 5 EV Figures can be typeset. EV Figures should be cited as 'Figure EV1, Figure EV2" etc... in the text and their respective legends should be included in the main text after the legends of regular figures.

- For the figures that you do NOT wish to display as Expanded View figures, they should be bundled together with their legends in a single PDF file called *Appendix*, which should start with a short Table of Content. Appendix figures should be referred to in the main text as: "Appendix Figure S1, Appendix Figure S2" etc. See detailed instructions regarding expanded view here: <https://www.embopress.org/page/journal/14693178/authorguide#expandedview>

- Additional Tables/Datasets should be labeled and referred to as Table EV1, Dataset EV1, etc. Legends have to be provided in a separate tab in case of .xls files. Alternatively, the legend can be supplied as a separate text file (README) and zipped together with the Table/Dataset file.

4) a .docx formatted letter INCLUDING the reviewers' reports and your detailed point-by-point responses to their comments. As part of the EMBO Press transparent editorial process, the point-by-point response is part of the Review Process File (RPF), which will be published alongside your paper.

5) a complete author checklist, which you can download from our author guidelines <https://www.embopress.org/page/journal/14693178/authorguide>. Please insert information in the checklist that is also reflected in the manuscript. The completed author checklist will also be part of the RPF.

6) Please note that all corresponding authors are required to supply an ORCID ID for their name upon submission of a revised manuscript (<https://orcid.org/>). Please find instructions on how to link your ORCID ID to your account in our manuscript tracking system in our Author guidelines <https://www.embopress.org/page/journal/14693178/authorguide#authorshipguidelines>

7) Before submitting your revision, primary datasets produced in this study need to be deposited in an appropriate public database (see https://www.embopress.org/page/journal/14693178/authorguide#datadeposition). Please remember to provide a reviewer password if the datasets are not yet public. The accession numbers and database should be listed in a formal "Data Availability" section placed after Materials & Method (see also https://www.embopress.org/page/journal/14693178/authorguide#datadeposition). Please note that the Data Availability Section is restricted to new primary data that are part of this study. * Note - All links should resolve to a page where the data can be accessed. *
If your study has not produced novel datasets, please mention this fact in the Data Availability Section.

8) At EMBO Press we ask authors to provide source data for the main manuscript figures. Our source data coordinator will contact you to discuss which figure panels we would need source data for and will also provide you with helpful tips on how to upload and organize the files.

9) Our journal also encourages inclusion of *data citations in the reference list* to directly cite datasets that were re-used and obtained from public databases. Data citations in the article text are distinct from normal bibliographical citations and should directly link to the database records from which the data can be accessed. In the main text, data citations are formatted as follows: "Data ref: Smith et al, 2001" or "Data ref: NCBI Sequence Read Archive PRJNA342805, 2017". In the Reference list, data citations must be labeled with "[DATASET]". A data reference must provide the database name, accession number/identifiers and a resolvable link to the landing page from which the data can be accessed at the end of the reference. Further instructions are available at https://www.embopress.org/page/journal/14693178/authorguide#referencesformat

10) Regarding data quantification (see Figure Legends: https://www.embopress.org/page/journal/14693178/authorguide#figureformat)

The following points must be specified in each figure legend:

- the name of the statistical test used to generate error bars and P values,

- the number (n) of independent experiments (please specify technical or biological replicates) underlying each data point,

- the nature of the bars and error bars (s.d., s.e.m.),

- If the data are obtained from n Program fragment delivered error ``Can't locate object method "less" via package "than" (perhaps you forgot to load "than"?) at //ejpvfs23/sites23b/embor_www/letters/embor_decision_revise_and_review.txt line 56.' 2, use scatter blots showing the individual data points.

Discussion of statistical methodology can be reported in the materials and methods section, but figure legends should contain a basic description of n, P and the test applied.

- Please also include scale bars in all microscopy images.

11) The journal requires a statement specifying whether or not authors have competing interests (defined as all potential or actual interests that could be perceived to influence the presentation or interpretation of an article). In case of competing interests, this must be specified in your disclosure statement. Further information: https://www.embopress.org/competing-interests

We would also welcome the submission of cover suggestions, or motifs to be used by our Graphics Illustrator in designing a cover.

As part of the EMBO publication's Transparent Editorial Process, EMBO reports publishes online a Review Process File (RPF) to accompany accepted manuscripts. This File will be published in conjunction with your paper and will include the referee reports, your point-by-point response and all pertinent correspondence relating to the manuscript.

You are able to opt out of this by letting the editorial office know (emboreports@embo.org). If you do opt out, the Review Process File link will point to the following statement: "No Review Process File is available with this article, as the authors have chosen not to make the review process public in this case."

I look forward to seeing a revised form of your manuscript when it is ready. Please use this link to submit your revision: https://embor.msubmit.net/cgi-bin/main.plex

Yours sincerely,

Esther Schnapp, PhD
Senior Editor
EMBO reports

Referee #2:

In this paper Dubois-Chevalier et al, presents an extensive analyses of transcription factor (TF) regulons aimed at better understanding the regulatory networks that control hepatocyte identity.
The authors followed a strategy which detects TF binding to TF encoding regulatory regions and define clusters of promoters through self-organizing map analysis and hierarchical clustering. These are subsequently characterized using cistrome, epigenome and transcriptome data sets to explore TF networks. They identified a cluster, which captured most TF-encoding genes preferentially targeted by the so-called Hep-ID TFs that have previously been identified as core transcriptional regulators in liver. A subset of TFs of the newly identified cluster, called Hep-ID CONNECT, has been found to represent an important part of the regulatory circuit determining hepatocyte identity.
The new findings provide evidence for a functional interaction of a set of more widely expressed, non-liver specific TFs, Hep-ID CONNECT, in controlling hepatocyte identity. This, unexpected, conclusion is based on a number of convincing data, including reciprocal regulation of the known Hep-ID TFs, co-binding to promoter targets and the pattern and domain length of H3K4Me3 nucleosomes at the near-TSS nucleosomes.
Another interesting finding is the role of Hep-ID CONNECT regulators in re-setting Hep-ID expression in cancer cells following treatment with LXRa ligand (GW3965) and THRb ligand (T3). In this part, an important new finding is that T3 can induce Hep-ID regulators, which are normally downregulated in cancerous cells.
Taken together the above, this paper provides a novel conceptual framework for understanding transcriptional control in cell identity: it proposes a more distributed control mechanism, where operation of combinatorial control of a larger number of regulators is necessary in addition to the function of few master regulators. These additional factors may function as identity stabilization loop that sense cellular states and resetting hepatocyte identity in de-differentiated cells.

Some minor questions, comments:
1) The data with LXR and THRb agonists are very fascinating. Besides transcript and western blot analyses of TFs have the authors observed any effect on growth properties?

2) Analyses of TCGA data in Figure 4A shows box plots for correlation between Hep-ID and Hep-ID CONNECT gene expression. Significance values need to be indicated in each box plot.

3) In Figure 4A, five of the Hep-ID CONNECT genes are indicated as tumor suppressors, which is based on literature. It would be nice if the authors could show for these few genes directly, that clinical features, like overall survival, tumor stage, tumor grade, metastatic features like lymph node invasion are affected in high and low expressing patients in the TCGA database.

Referee #3:

Dubois-Chevalier et al use a bioinformatic approach to identify key transcription factor networks important for hepatocyte cell identity. Their approach builds on the observation that many TFs important for hepatocyte function occupy the promoters of the genes coding for the very same TFs (auto-regulatory loops). They extend the analysis to include additional TFs that are suggested to support these regulatory loops. To test the importance of these TF regulatory networks they analyze RNAseq data from livers with genetic disruption of specific TFs within the network. Moreover, they test the importance using RNAseq analysis from two different liver disease/injury models: human hepatocarcinoma (HCC) and acute liver injury. In the HCC, they support analysis with RNAseq data of hepatic cancer cell lines treated with activators of specific TFs in the network. Treatment leads to increased hepatocyte identity of these cell lines. This collectively suggests that the TF network described is important for the maintenance of hepatic cellular identity under pathophysiological conditions.

The bioinformatic approach they authors use to identify the TF networks is interesting and seems sound. However, the presentation and description of the bioinformatic analysis is convoluted and could be simplified. To test the importance of the model the authors primarily use RNAseq data from liver tissue. Since liver tissue is composed of many different cell types it will be hard to pinpoint observed differential expression to the hepatocytes and verification by single-cell RNAseq analysis is needed.

Specific comments:

The authors present three converging bioinformatic strategies to classify TF promoters in hepatocytes. They use this to identify TF regulatory networks - networks of TFs regulating each other's expression.

Classification 1
First, TF gene promoters active in hepatocytes are identified. This is defined by TF occupancy and PROseq data. This definition is different from promoter classification 2 (see below). Why do the authors have two different promoter activity classifications?

They then measure TF binding to these TF promoters using published cistromes from liver tissue (n=49) and perform a cluster analysis. This identifies TF promoters with different degrees of TF occupancy (clusters A-G). The TF promoters in cluster G show high co-occupancy of TF cistomes. From cluster G, eight HepID TFs are identified. They then move to integrate these TFs into TF promoter classification 2.

Classification 2
They first take all TF gene promoters and score activity based on mRNA level, DHS, H3K27Ac etc. And then perform cluster analysis which leads to the identification of 7 clusters. They compare clusters 1-7 with clusters A-G and identify cluster G-7 to contain TF promoters highly active in hepatocytes. This second promoter activity score seems more robust compared to the one described in classification 1 (above). Why not use this one to score for highly active promoters and then use hepatic TF cistromes to score for TF regulatory networks?

Classification 3
Here they rank the expression level of TFs in primary hepatocytes compared to 38 other cell types. They then compare this with clusters G (classification 1) and clusters 4-7 (classification 2) leading to G4-7 clusters. In the ranking of TFs they set an arbitrary cutoff at 10 and HepID(connect) TFs are identified. Why is the cutoff set at 10 and not 15 or 5?.

Collectively, the integration of classifications 1-3 ends up with two classes of TFs called Hep-ID (8 TFs) and Hep-ID(connect) (26 TFs). Analysis for classifications 1-3 is presented in figure 1 and figures S1-S9. The analysis seems convoluted and, in my opinion, contains too many rounds of iterations, and thus hard to fully understand. A simplification would improve the reading and understanding of this section of the manuscript.

The two classes of TF promoters, Hep-ID and Hep-ID(connect), indicate an important role of these TFs for hepatocyte cell identification. With the numerous liver single-cell RNAseq studies published in recent years, the authors have an opportunity to also analyze if these TF promoters/genes are also specifically active/expressed in hepatocytes based on single-cell RNAseq data of liver tissue and other tissues (human and mouse). This can potentially refine the presented analysis.

The authors argue that the Hep-ID(connect) TFs serves as transcriptional stabilizers to maintain hepatocyte cell identity. They test this by analyzing TF expression in HCC and an acute liver injury mouse model. Both analysis strategies are based on bulk RNAseq data from tissues. However, diseased liver tissue dramatically changes cellular composition which changes the sequenced reads obtained from hepatocytes. For example, the mouse model for loss of hepatocyte cell identity (liver injury) presented in figure 5 is based on bulk liver RNAseq analysis. Treatment of IL1B leads to massive immune cell infiltration to the liver and dramatically shifts hepatocyte to non-hepatocyte cell ratio. When RNAseq is performed this shift sequenced reads towards transcripts from infiltrating immune cells and fewer reads are consequently detected from hepatocyte-specific genes. Thus, the reduced expression of hepatocyte-specific TFs seen in figure 5 may not reflect the change in hepatocyte identity but may just reflect the relative change in cell numbers. To address this the authors must perform single-cell RNAseq analysis or experiments in primary hepatocytes isolated from the injury model.

Another experiment to analyze Hep-ID(connect) TF expression during the loss of cell identity could be done by following the dedifferentiation of primary hepatocytes in vitro. Maintenance of primary hepatocytes in culture leads to the loss of many key hepatocyte functions and thus would be a good cell intrinsic model to test the importance of Hep-ID and Hep-ID(connect) TFs for hepatic cell identity. This model could also be used for treatment with ligands against Hep-ID(connect) TFs and see if this maintains intrinsic hepatocyte identity. For example, treatment with T3 and GW3965.

Minor comments:

Page 10: (Meng et al., 2020) should not be in brackets

Page 11: "has been shown to opposite hepatocarcinogenesis". Opposite should be replaced with a better-fitting word. For example, prevent.

Page 11: "Moreover, binging of Hep-IDCONNECT TFs to Hep-ID TF genes". Binging is not the correct word. Maybe the authors mean grouping or combining?

Page 23: "HepIDCONNECTHep TF binding to Hep-ID TF encoding genes in HepG2 cells". This heading/paragraph seems to lack content.

The authors use several previously published NGS data sets in their bioinformatic analysis. It would be informative with a list of all the GEO accession numbers used for the analysis so one would have the opportunity to reproduce the analysis.

We would like to thank the reviewers for their constructive evaluation of our manuscript. We provide below a point-by-point response to their comments. Changes in the manuscript are highlighted in red (see provided .doc Word file; page and line numbers provided below refer to this Word file).

Referee 2:

In this paper Dubois-Chevalier et al, presents an extensive analyses of transcription factor (TF) regulons aimed at better understanding the regulatory networks that control hepatocyte identity.

The authors followed a strategy which detects TF binding to TF encoding regulatory regions and define clusters of promoters through self-organizing map analysis and hierarchical clustering. These are subsequently characterized using cistrome, epigenome and transcriptome data sets to explore TF networks. They identified a cluster, which captured most TF-encoding genes preferentially targeted by the so-called Hep-ID TFs that have previously been identified as core transcriptional regulators in liver. A subset of TFs of the newly identified cluster, called Hep-ID CONNECT, has been found to represent an important part of the regulatory circuit determining hepatocyte identity.

The new findings provide evidence for a functional interaction of a set of more widely expressed, non-liver specific TFs, Hep-ID CONNECT, in controlling hepatocyte identity. This, unexpected, conclusion is based on a number of convincing data, including reciprocal regulation of the known Hep-ID TFs, co-binding to promoter targets and the pattern and domain length of H3K4Me3 nucleosomes at the near-TSS nucleosomes.

Another interesting finding is the role of Hep-ID CONNECT regulators in re-setting Hep-ID expression in cancer cells following treatment with LXRa ligand (GW3965) and THRb ligand (T3). In this part, an important new finding is that T3 can induce Hep-ID regulators, which are normally downregulated in cancerous cells.

Taken together the above, this paper provides a novel conceptual framework for understanding transcriptional control in cell identity: it proposes a more distributed control mechanism, where

operation of combinatorial control of a larger number of regulators is necessary in addition to the function of few master regulators. These additional factors may function as identity stabilization loop that sense cellular states and resetting hepatocyte identity in de-differentiated cells.

Some minor questions, comments:

1) The data with LXR and THRb agonists are very fascinating. Besides transcript and western blot analyses of TFs have the authors observed any effect on growth properties?

In order to answer this question, we treated HepG2 cells and counted viable cells after 7 days. We observed that the rise in Hep-ID TFs levels triggered by the THRB and LXR agonists (Fig.4) translated into reduced cell number (Appendix Figure S12D, referred to in the revised manuscript page 11 lines 18-19). These data are consistent with previous reports of THRB- and LXR-mediated growth inhibition of HCC cells (PMID 32528965 and PMID 23812424) and are in keeping with the recent study by Kowalik et al (PMID 31954205) which showed that treatment of HCC-bearing rats with T3 strongly reduced the number and burden of HCCs.

2) Analyses of TCGA data in Figure 4A shows box plots for correlation between Hep-ID and Hep-ID CONNECT gene expression. Significance values need to be indicated in each box plot.

We would like to thank the reviewer for this comment, which led us to improve our gene expression correlation analyses. Indeed, we are now showing the correlations between expression of individual Hep-ID^{CONNECT} genes and all Hep-ID TF genes taken as a gene set (i.e. their mean expression was used for correlation). This allowed us to show correlations and significance on the same plot (Fig.4C). This is also justified by the increased robustness observed when involving gene sets in such analyses (PMID: 35368077). In that regard, a novel panel has been added to show that the most significant positive correlation is obtained when comparing the mean expression of all Hep-ID^{CONNECT} with that of all Hep-ID TF encoding genes in HCC (Fig.4A). As requested by the reviewer, significance values are now provided for all correlations analyzed (Fig.4A and C). These data are described from page 10 line 22 to page 11 line 3. The methods section and figure legend have been amended accordingly.

3) In Figure 4A, five of the Hep-ID CONNECT genes are indicated as tumor suppressors, which is based on literature. It would be nice if the authors could show for these few genes directly, that clinical features, like overall survival, tumor stage, tumor grade, metastatic features like lymph node invasion are affected in high and low expressing patients in the TCGA database.

We have extended our analyses and now show that, similarly to Hep-ID TF encoding genes, expression of Hep-ID^{CONNECT} TF encoding genes (taken as a gene set) is linked to significantly increased 10-year overall survival in patients with HCC (Fig.4B and Appendix Figure S12A). High expression of Hep-ID and Hep-ID^{CONNECT} TF encoding genes was also linked to a trend towards early HCC stages (Appendix Figure S12B). Overall, similar analyses performed with individual genes gave less significant results indicating that mining Hep-ID and Hep-ID^{CONNECT} TF genes as gene sets provided robustness to our analyses as already stated. Nevertheless, survival analyses for the five Hep-ID^{CONNECT} TFs highlighted in Fig.4C (formerly Fig.4A) are shown in Appendix Figure S12C as per the reviewer's request. These data show that among those, high THRB expression was the most significantly associated with greater 10-year overall survival. Note that lack of similar findings for NR1H3 may stem from the role exerted by NR1H3 not only in tumor cells but also in cells of the tumor microenvironment such as macrophages (PMID 35359989). These data are described from page 10 lines 25 to page 11 line 2 and page 11 lines 10-11. The methods section and figure legend have been amended accordingly.

Referee 3:

Dubois-Chevalier et al use a bioinformatic approach to identify key transcription factor networks important for hepatocyte cell identity. Their approach builds on the observation that many TFs important for hepatocyte function occupy the promoters of the genes coding for the very same TFs (auto-regulatory loops). They extend the analysis to include additional TFs that are suggested to support these regulatory loops. To test the importance of these TF regulatory networks they analyze RNAseq data from livers with genetic disruption of specific TFs within the network. Moreover, they test the importance using RNAseq analysis from two different liver

disease/injury models: human hepatocarcinoma (HCC) and acute liver injury. In the HCC, they support analysis with RNAseq data of hepatic cancer cell lines treated with activators of specific TFs in the network. Treatment leads to increased hepatocyte identity of these cell lines. This collectively suggests that the TF network described is important for the maintenance of hepatic cellular identity under pathophysiological conditions.

The bioinformatic approach they authors use to identify the TF networks is interesting and seems sound. However, the presentation and description of the bioinformatic analysis is convoluted and could be simplified. To test the importance of the model the authors primarily use RNAseq data from liver tissue. Since liver tissue is composed of many different cell types it will be hard to pinpoint observed differential expression to the hepatocytes and verification by single-cell RNAseq analysis is needed.

Specific comments:

The authors present three converging bioinformatic strategies to classify TF promoters in hepatocytes. They use this to identify TF regulatory networks - networks of TFs regulating each other's expression.

Classification 1

First, TF gene promoters active in hepatocytes are identified. This is defined by TF occupancy and PROseq data. This definition is different from promoter classification 2 (see below). Why do the authors have two different promoter activity classifications?

In order to mine the hepatocyte transcriptional regulatory network, our strategy was to first mine TF binding to TF-encoding genes through Promoter-centric TF network analysis (ProTFnet) (here referred to as classification 1 by the reviewer). In this context, we decided to remove non-expressed genes to avoid non-informative and noisy information, which could compromise our analyses. With this aim, we restricted our analyses to transcriptionally active genes, i.e. TF-encoding genes with detectable expression levels as defined using Pro-seq by Wang et al. (PMID: 30139328). This initial step was therefore a filter to remove inactive genes while subsequent analyses were meant to classify active genes based on their levels of activity.

This clarification has been made page 6 lines 2-3 and in the Material and Methods section page 16 lines 13-14.

They then measure TF binding to these TF promoters using published cistromes from liver tissue (n=49) and perform a cluster analysis. This identifies TF promoters with different degrees of TF occupancy (clusters A-G). The TF promoters in cluster G show high co-occupancy of TF cistomes. From cluster G, eight HepID TFs are identified. They then move to integrate these TFs into TF promoter classification 2.

Classification 2

They first take all TF gene promoters and score activity based on mRNA level, DHS, H3K27Ac etc. And then perform cluster analysis which leads to the identification of 7 clusters. They compare clusters 1-7 with clusters A-G and identify cluster G-7 to contain TF promoters highly active in hepatocytes. This second promoter activity score seems more robust compared to the one described in classification 1 (above). Why not use this one to score for highly active promoters and then use hepatic TF cistromes to score for TF regulatory networks?

As stated hereabove, classification 1 did not mean to organize genes with regards to their activity levels but rather with regards to their TF recruitment patterns since the original aim of the study was to mine the hepatocyte TF network. However, we acknowledge that our subsequent assessment of gene activity levels could be improved and simplified as requested by the reviewer hereafter. In particular, classification 2 was mostly used to show that TF-encoding genes from cluster G were not restricted to the most active and liver-specific genes in order to introduce classification 3. Therefore, we removed classification 2 and now make this point in the revised manuscript by more simply showing the distribution of the liver gene expression levels and specificity (revised Fig.1G and H described page 6 lines 16-18). This simplification allowed us to remove the description of classification 2 in the Results section together with removal of the former Fig.S6 and S7 and simplification of Table EV1. The methods section and figure legend have been amended taking into account the aforementioned amendments.

Classification 3

Here they rank the expression level of TFs in primary hepatocytes compared to 38 other cell types. They then compare this with clusters G (classification 1) and clusters 4-7 (classification 2) leading to G4-7 clusters. In the ranking of TFs they set an arbitrary cutoff at 10 and HepID(connect) TFs are identified. Why is the cutoff set at 10 and not 15 or 5?.

Our aim was to provide a proof-of-concept that TFs from cluster G characterized by privileged hepatocyte expression were involved in the control of hepatocyte identity. We therefore chose to use a conservative cut-off (set at 10) to define Hep-ID^{CONNECT} genes in Fig.2A. Additional TF-encoding genes (for example ranked between 10 and 15) also display biased hepatocyte expression and could bear similar roles as those described for Hep-ID^{CONNECT} TFs. The entire set of data used to plot Fig.2A is now made available as a novel Table EV3 so that any reader interested in mining additional potential TFs beyond the ones comprised in our restricted set of Hep-ID^{CONNECT} TFs can easily do so. This is now stated page 7 lines 14-15.

Collectively, the integration of classifications 1-3 ends up with two classes of TFs called Hep-ID (8 TFs) and Hep-ID(connect) (26 TFs). Analysis for classifications 1-3 is presented in figure 1 and figures S1-S9. The analysis seems convoluted and, in my opinion, contains too many rounds of iterations, and thus hard to fully understand. A simplification would improve the reading and understanding of this section of the manuscript.

As detailed hereabove, this part of the manuscript has been simplified as per the reviewer's request.

The two classes of TF promoters, Hep-ID and Hep-ID(connect), indicate an important role of these TFs for hepatocyte cell identification. With the numerous liver single-cell RNAseq studies published in recent years, the authors have an opportunity to also analyze if these TF promoters/genes are also specifically active/expressed in hepatocytes based on single-cell

RNAseq data of liver tissue and other tissues (human and mouse). This can potentially refine the presented analysis.

In order to answer this point, we have re-analyzed single-nuclei RNA-seq data obtained from healthy mouse and human livers from PMID 35021063. Indeed, single-nuclei RNA-seq allows to have a better representation of the various liver cell-types including hepatocytes when compared to single-cell RNA-seq (PMID 35021063). Overall, the data show that expression of Hep-ID^{CONNECT} genes is the greatest in hepatocytes (except for macrophages in human livers), a pattern similar to that of Hep-ID TF genes but at lower expression ranges (Appendix Figure S5). These data are in line with previous gene expression analyses we had performed using bulk RNA-seq analyses from livers and primary cells and are described page 7 lines 17-19. The methods section now includes a description of single-nuclei RNA-seq data analysis.

The authors argue that the Hep-ID(connect) TFs serves as transcriptional stabilizers to maintain hepatocyte cell identity. They test this by analyzing TF expression in HCC and an acute liver injury mouse model. Both analysis strategies are based on bulk RNAseq data from tissues. However, diseased liver tissue dramatically changes cellular composition which changes the sequenced reads obtained from hepatocytes. For example, the mouse model for loss of hepatocyte cell identity (liver injury) presented in figure 5 is based on bulk liver RNAseq analysis. Treatment of IL1B leads to massive immune cell infiltration to the liver and dramatically shifts hepatocyte to non-hepatocyte cell ratio. When RNAseq is performed this shift sequenced reads towards transcripts from infiltrating immune cells and fewer reads are consequently detected from hepatocyte-specific genes. Thus, the reduced expression of hepatocyte-specific TFs seen in figure 5 may not reflect the change in hepatocyte identity but may just reflect the relative change in cell numbers. To address this the authors must perform single-cell RNAseq analysis or experiments in primary hepatocytes isolated from the injury model.

In Fig.5, we deliberately chose to use an acute liver injury model (consisting in an intraperitoneal injection of IL1B for a short time (6h) before livers were collected) to avoid

adverse effects linked to longer or more chronic inflammation such as massive immune cell infiltration. Indeed, immune cell infiltration had not been detected 6h after induction of acute liver injuries except for initial neutrophil infiltration (PMID: 36569272). Indeed, neutrophils are the largest population of circulating leukocytes and the first responder against invading pathogens or other danger signals (PMID: 33159158 and PMID: 33763069). In line, we found using RT-qPCR from our mouse livers that IL1B treatment led to increased levels of the neutrophil marker *Ly6g*. However, this did not translate into a rise of the broad immune cell marker *Ptprc* (also known as *CD45*). Moreover, expression of *Ly6g* and *CD45* was not different between the IL1B and IL1B+T3 groups (Figure EV5D). This led us to conclude that only a limited and initial infiltration of neutrophils occurs in our experimental model of IL1B-induced acute liver injury, which is inconsistent with a dramatic change in cell populations which would have compromised our gene expression analyses and conclusions regarding the effect of T3 treatment. These novel data are described from page 13 line 25 to page 14 line 1 and shown in Figure EV5D.

Another experiment to analyze Hep-ID(connect) TF expression during the loss of cell identity could be done by following the dedifferentiation of primary hepatocytes in vitro. Maintenance of primary hepatocytes in culture leads to the loss of many key hepatocyte functions and thus would be a good cell intrinsic model to test the importance of Hep-ID and Hep-ID(connect) TFs for hepatic cell identity. This model could also be used for treatment with ligands against Hep-ID(connect) TFs and see if this maintains intrinsic hepatocyte identity. For example, treatment with T3 and GW3965.

Although they maintain some levels of functionalities, the reviewer is right when stating that primary hepatocytes undergo dedifferentiation *in vitro*. We have assessed the ability of T3 to intervene with this spontaneous dedifferentiation and could not detect any protective effect on loss of Hep-ID TF gene expression (Appendix Figure S14). However, interfering with this process has been notoriously very difficult to achieve. For instance, previous reports indicate that dampening this process requires a combined inhibition of multiple dedifferentiating

signaling pathways (PMID: 31801084 and PMID: 31023926). Indeed, MPH dedifferentiation *in vitro* is a complex phenomenon not fully characterized but involving signaling triggered by mechanical tensions, TGFβ and EMT (PMID: 31801084 and PMID: 31023926). Moreover, forced expression of supra-physiologic amounts of the Hep-ID TF HNF4A was only able to partially impede MPH dedifferentiation (PMID: 34453962, PMID: 25774505, PMID: 34558820). Altogether, these studies and our own experience point to rapid and dramatic induction of dedifferentiating signals in MPH, which are extremely difficult to circumvent. In line, Seirup et al. recently defined that drastic and rapid changes in chromatin accessibility occur immediately upon the start of MPH culture (PMID: 29981427). Moreover, *Thrb* expression rapidly decreases in MPH (Appendix Figure S14). Taken together, these events may limit the ability of T3 to trigger re-expression of Hep-ID TF genes. Data obtained the MPH intrinsic dedifferentiation model are discussed in the revised manuscript from page 12 lines 13 to page 13 line 3.

Minor comments:

Page 10: (Meng et al., 2020) should not be in brackets

This has been corrected.

Page 11: "has been shown to opposite hepatocarcinogenesis". Opposite should be replaced with a better-fitting word. For example, prevent.

“Opposite” has been replaced by “suppress” in this sentence.

Page 11: "Moreover, binging of Hep-IDCONNECT TFs to Hep-ID TF genes". Binging is not the correct word. Maybe the authors mean grouping or combining?

This typo has been corrected. “Binding” is now indicated in this sentence.

Page 23: "HepIDCONNECTHep TF binding to Hep-ID TF encoding genes in HepG2 cells". This heading/paragraph seems to lack content.

This sentence has been removed.

The authors use several previously published NGS data sets in their bioinformatic analysis. It would be informative with a list of all the GEO accession numbers used for the analysis so one would have the opportunity to reproduce the analysis.

All publicly available data and their accession numbers are provided in Table EV2.

1st Revision - Editorial Decision **15th Jun 2023**

Dear Dr. Eeckhoute,

Thank you for the submission of your revised manuscript. We have now received the enclosed reports from the referees and I am happy to say that both support its publication now.

There are only a few editorial requests that will need to be addressed before we can proceed with the official acceptance of your manuscript.

- Please correct the conflict of interest subheading to "DISCLOSURE AND COMPETING INTEREST STATEMENT"

- Please remove the author credits from the ms file. We now use CRediT to specify the contributions of each author in the journal submission system. CRediT replaces the author contribution section. Please use the free text box to provide more detailed descriptions, if you wish. See also our guide to authors https://www.embopress.org/page/journal/14693178/authorguide#authorshipguidelines.

- Please add these missing figure callouts: Fig. EV2A-B, EV4A-C, Appendix Figure S3A-F, Appendix Figure S4A-C, Appendix Figure S5A-B, Appendix Figure S9A-B, Appendix Figure S1-A-C

- Tables EV1 and EV2 should be renamed to Dataset EV1-EV2 with the legends uploaded as a separate sheet in each Excel file. Tables EV3-EV7 should be renumbered to EV1-EV5. Please also correct all callouts in the ms text.

- In the Appendix table of content page numbers are missing, please add. The nomenclature should be Appendix Figure S1-S14 with the corresponding callouts in the ms text.

- The Data availability section should be placed before the Acknowledgements section.

- The funding information should be included in the Acknowledgements section.

- I attach to this email a related ms file with comments by our data editors. Please address all comments in the final ms.

EMBO press papers are accompanied online by A) a short (1-2 sentences) summary of the findings and their significance, B) 2-3 bullet points highlighting key results and C) a synopsis image that is exactly 550 pixels wide and 200-600 pixels high (the height is variable). You can either show a model or key data in the synopsis image. Please note that text needs to be readable at the final size. Please send us this information along with the final manuscript.

I would like to suggest some changes to the title and abstract that needs to be written in present tense. Please let me know whether you agree with the following:

An extended transcription factor regulatory network controls hepatocyte identity

Cell identity is specified by a core transcriptional regulatory circuitry (CoRC), typically limited to a small set of interconnected cell-specific transcription factors (TFs). By mining global hepatic TF regulons, we reveal a more complex organization of the transcriptional regulatory network controlling hepatocyte identity. We show that tight functional interconnections controlling hepatocyte identity extend to non-cell-specific TFs beyond the CoRC, which we call hepatocyte identity (Hep-ID)CONNECT TFs. Besides controlling identity effector genes, Hep-IDCONNECT TFs also engage in reciprocal transcriptional regulation with TFs of the CoRC. In homeostatic basal conditions, this translates into Hep-IDCONNECT TFs being involved in fine tuning CoRC TF expression including their rhythmic expression patterns. Also, a role for Hep-IDCONNECT TFs in the control of hepatocyte identity is revealed in dedifferentiated hepatocytes where Hep-IDCONNECT TFs are able to reset CoRC TF expression. This is observed upon activation of NR1H3 or THRB in hepatocarcinoma or in hepatocytes subjected to inflammation-induced loss of identity. Our study establishes that hepatocyte identity is controlled by an extended array of TFs beyond the CoRC.

I removed the "stabilization loops" as it is somewhat unclear what exactly you mean by that.

I look forward to seeing a final version of your manuscript as soon as possible. Please use this link to submit your revision: https://embor.msubmit.net/cgi-bin/main.plex

Best regards,
Esther

Esther Schnapp, PhD
Senior Editor
EMBO reports

Referee #2:

I am satisfied with the answers of the authors and the data addressing my questions. The revised version represents a high quality work, which will be of interest to a wide audience.

Referee #3:

The revised manuscript including additional data analysis has addressed the concerns raised by the initial review. I have no additional comments to the revised manuscript.

The authors have addressed all minor editorial requests.

2nd Revision - Editorial Decision 21st Jun 2023

Jerome Eeckhoute
Université de Lille Nord
INSERM U1011
Faculté de Médecine de Lille - Pôle Recherche Boulevard du Professeur Leclerc, Bâtiment J&K
Lille, France 59045
France

Dear Dr. Eeckhoute,

I am very pleased to accept your manuscript for publication in the next available issue of EMBO reports. Thank you for your contribution to our journal.

At the end of this email I include important information about how to proceed. Please ensure that you take the time to read the information and complete and return the necessary forms to allow us to publish your manuscript as quickly as possible.

As part of the EMBO publication's Transparent Editorial Process, EMBO reports publishes online a Review Process File to accompany accepted manuscripts. As you are aware, this File will be published in conjunction with your paper and will include the referee reports, your point-by-point response and all pertinent correspondence relating to the manuscript.

If you do NOT want this File to be published, please inform the editorial office within 2 days, if you have not done so already, otherwise the File will be published by default [contact: emboreports@embo.org]. If you do opt out, the Review Process File link will point to the following statement: "No Review Process File is available with this article, as the authors have chosen not to make the review process public in this case."

Thank you again for your contribution to EMBO reports and congratulations on a successful publication. Please consider us again in the future for your most exciting work.

Yours sincerely,

Esther Schnapp, PhD
Senior Editor
EMBO reports

****

THINGS TO DO NOW:

Please note that you will be contacted by Wiley Author Services to complete licensing and payment information. The required 'Page Charges Authorization Form' is available here: https://www.embopress.org/pb-assets/embo-site/er_apc.pdf - please download and complete the form and return to embopressproduction@wiley.com

EMBO Press participates in many Publish and Read agreements that allow authors to publish Open Access with reduced/no publication charges. Check your eligibility: https://authorservices.wiley.com/author-resources/Journal-Authors/open-access/affiliation-policies-payments/index.html

You will receive proofs by e-mail approximately 2-3 weeks after all relevant files have been sent to our Production Office; you should return your corrections within 2 days of receiving the proofs.

Please inform us if there is likely to be any difficulty in reaching you at the above address at that time. Failure to meet our deadlines may result in a delay of publication, or publication without your corrections.

All further communications concerning your paper should quote reference number EMBOR-2023-57020V3 and be addressed to emboreports@wiley.com.

Should you be planning a Press Release on your article, please get in contact with emboreports@wiley.com as early as possible, in order to coordinate publication and release dates.

EMBO Press Author Checklist

Corresponding Author Name: EECKHOUTE Jerome	
Journal Submitted to: EMBO reports	
Manuscript Number: EMBOR-2023-57020-T	

USEFUL LINKS FOR COMPLETING THIS FORM
The EMBO Journal - Author Guidelines
EMBO Reports - Author Guidelines
Molecular Systems Biology - Author Guidelines
EMBO Molecular Medicine - Author Guidelines

Reporting Checklist for Life Science Articles (updated January 2022)
This checklist is adapted from Materials Design Analysis Reporting (MDAR) Checklist for Authors. MDAR establishes a minimum set of requirements in transparent reporting in the life sciences (see Statement of Task: 10.31222/osf.io/9sm4x). Please follow the journal's guidelines in preparing your manuscript.
Please note that a copy of this checklist will be published alongside your article.

Abridged guidelines for figures
1. Data
The data shown in figures should satisfy the following conditions:
- the data were obtained and processed according to the field's best practice and are presented to reflect the results of the experiments in an accurate and unbiased manner.
- ideally, figure panels should include only measurements that are directly comparable to each other and obtained with the same assay.
- plots include clearly labeled error bars for independent experiments and sample sizes. Unless justified, error bars should not be shown for technical replicates.
- if n<5, the individual data points from each experiment should be plotted. Any statistical test employed should be justified.
- Source Data should be included to report the data underlying figures according to the guidelines set out in the authorship guidelines on Data Presentation.

2. Captions
Each figure caption should contain the following information, for each panel where they are relevant:
- a specification of the experimental system investigated (eg cell line, species name).
- the assay(s) and method(s) used to carry out the reported observations and measurements.
- an explicit mention of the biological and chemical entity(ies) that are being measured.
- an explicit mention of the biological and chemical entity(ies) that are altered/varied/perturbed in a controlled manner.
- the exact sample size (n) for each experimental group/condition, given as a number, not a range;
- a description of the sample collection allowing the reader to understand whether the samples represent technical or biological replicates (including how many animals, litters, cultures, etc.).
- a statement of how many times the experiment shown was independently replicated in the laboratory.
- definitions of statistical methods and measures:
  - common tests, such as t-test (please specify whether paired vs. unpaired), simple χ2 tests, Wilcoxon and Mann-Whitney tests, can be unambiguously identified by name only, but more complex techniques should be described in the methods section;
  - are tests one-sided or two-sided?
  - are there adjustments for multiple comparisons?
  - exact statistical test results, e.g., P values = x but not P values < x;
  - definition of 'center values' as median or average;
  - definition of error bars as s.d. or s.e.m.

Please complete ALL of the questions below.
Select "Not Applicable" only when the requested information is not relevant for your study.

Materials

Newly Created Materials	Information included in the manuscript?	In which section is the information available? (Reagents and Tools Table, Materials and Methods, Figures, Data Availability Section)
New materials and reagents need to be available; do any restrictions apply?	Not Applicable	

Antibodies	Information included in the manuscript?	In which section is the information available? (Reagents and Tools Table, Materials and Methods, Figures, Data Availability Section)
For **antibodies** provide the following information:<br>- Commercial antibodies: RRID (if possible) or supplier name, catalogue number and or/clone number<br>- Non-commercial: RRID or citation	Yes	Table EV6

DNA and RNA sequences	Information included in the manuscript?	In which section is the information available? (Reagents and Tools Table, Materials and Methods, Figures, Data Availability Section)
**Short novel DNA or RNA including primers, probes**: provide the sequences.	Yes	Table EV5

Cell materials	Information included in the manuscript?	In which section is the information available? (Reagents and Tools Table, Materials and Methods, Figures, Data Availability Section)
**Cell lines:** Provide species information, strain. Provide accession number in repository **OR** supplier name, catalog number, clone number, and/**OR** RRID.	Yes	Table EV6
**Primary cultures:** Provide species, strain, sex of origin, genetic modification status.	Yes	Materials and methods
Report if the cell lines were recently **authenticated** (e.g., by STR profiling) and tested for mycoplasma contamination.	Yes	Materials and methods

Experimental animals	Information included in the manuscript?	In which section is the information available? (Reagents and Tools Table, Materials and Methods, Figures, Data Availability Section)
**Laboratory animals or Model organisms:** Provide species, strain, sex, age, genetic modification status. Provide accession number in repository **OR** supplier name, catalog number, clone number, **OR** RRID.	Yes	Materials and methods
**Animal observed in or captured from the field:** Provide species, sex, and age where possible.	Not Applicable	
Please detail **housing and husbandry conditions**.	Yes	Materials and methods

Plants and microbes	Information included in the manuscript?	In which section is the information available? (Reagents and Tools Table, Materials and Methods, Figures, Data Availability Section)
**Plants:** provide species and strain, ecotype and cultivar where relevant, unique accession number if available, and source (including location for collected wild specimens).	Not Applicable	
**Microbes:** provide species and strain, unique accession number if available, and source.	Not Applicable	

Human research participants	Information included in the manuscript?	In which section is the information available? (Reagents and Tools Table, Materials and Methods, Figures, Data Availability Section)
If collected and within the bounds of privacy constraints report on age, sex and gender or ethnicity for all study participants.	Not Applicable	

Core facilities	Information included in the manuscript?	In which section is the information available? (Reagents and Tools Table, Materials and Methods, Figures, Data Availability Section)
If your work benefited from core facilities, was their service mentioned in the acknowledgments section?	Not Applicable	

Design

Study protocol	Information included in the manuscript?	In which section is the information available? (Reagents and Tools Table, Materials and Methods, Figures, Data Availability Section)

If study protocol has been **pre-registered, provide DOI in the manuscript**. For clinical trials, provide the trial registration number **OR** cite DOI.	Not Applicable	
Report the **clinical trial registration number** (at ClinicalTrials.gov or equivalent), where applicable.	Not Applicable	

Laboratory protocol	Information included in the manuscript?	In which section is the information available? (Reagents and Tools Table, Materials and Methods, Figures, Data Availability Section)
Provide DOI OR other citation details if **external detailed step-by-step protocols** are available.	Not Applicable	

Experimental study design and statistics	Information included in the manuscript?	In which section is the information available? (Reagents and Tools Table, Materials and Methods, Figures, Data Availability Section)
Include a statement about **sample size** estimate even if no statistical methods were used.	Yes	The sample size was chosen as a function of the inherent variability of the different types of experiments/models. In vitro experiments were repeated at least three times (independent biological experiments), based on experience and previous work (e.g. Lefebvre et al, JCI insight 2017, Dubois et al MolSystBio 2020).<br>For in vivo experiments, a preliminary assessment of T3-mediated effects was performed and used to define group sizes of additional experiments.
Were any steps taken to minimize the effects of subjective bias when allocating animals/samples to treatment (e.g. **randomization procedure**)? If yes, have they been described?	Yes	No randomization procedure was implemented in this study.
Include a statement about **blinding** even if no blinding was done.	Yes	Blinding was not relevant since absolute quantitative methods without human subjectivity were used.
Describe **inclusion/exclusion criteria** if samples or animals were excluded from the analysis. Were the criteria pre-established?<br><br>If sample or data points were omitted from analysis, report if this was due to attrition or intentional exclusion and provide justification.	Yes	No samples or animals have been excluded from our analyses. Rare missing data in RT-qPCR analyses of gene expression stem from exclusion based on technical failure.
For every figure, are **statistical tests** justified as appropriate? Do the data meet the assumptions of the tests (e.g., normal distribution)? Describe any methods used to assess it. Is there an estimate of variation within each group of data? Is the variance similar between the groups that are being statistically compared?	Yes	Materials and methods, Figure legends. No assumptions for normal distribution and variance equality are required for non-parametric tests. Visual inspection was used to control for similar distributions. Regarding one-sided Wilcoxon rank sum tests used to determine if a median was statistically significanlty different from 0, we visualy checked that the log2 fold changes of all genes was symetric around 0. Regarding one-sample t-test on RT-qPCR data (n=3), we assumed normal distribution of log2 fold changes.

Sample definition and in-laboratory replication	Information included in the manuscript?	In which section is the information available? (Reagents and Tools Table, Materials and Methods, Figures, Data Availability Section)
In the figure legends: state number of times the experiment was **replicated** in laboratory.	Yes	Figure legends
In the figure legends: define whether data describe **technical or biological replicates**.	Yes	Figure legends

Ethics

Ethics	Information included in the manuscript?	In which section is the information available? (Reagents and Tools Table, Materials and Methods, Figures, Data Availability Section)
Studies involving **human participants**: State details of **authority granting ethics approval** (IRB or equivalent committee(s), provide reference number for approval.	Not Applicable	
Studies involving **human participants**: Include a statement confirming that **informed consent** was obtained from all subjects and that the experiments conformed to the principles set out in the WMA Declaration of Helsinki and the Department of Health and Human Services Belmont Report.	Not Applicable	
Studies involving **human participants:** For publication of **patient photos**, include a statement confirming that consent to publish was obtained.	Not Applicable	
Studies involving experimental **animals**: State details of **authority granting ethics approval** (IRB or equivalent committee(s), provide reference number for approval. Include a statement of compliance with ethical regulations.	Not Applicable	
Studies involving **specimen and field samples:** State if relevant **permits** obtained, provide details of authority approving study; if none were required, explain why.	Not Applicable	

Dual Use Research of Concern (DURC)	Information included in the manuscript?	In which section is the information available? (Reagents and Tools Table, Materials and Methods, Figures, Data Availability Section)
Could your study fall under dual use research restrictions? Please check biosecurity documents and list of **select agents and toxins** (CDC): https://www.selectagents.gov/sat/list.htm	Not Applicable	
If you used a select agent, is the security level of the lab appropriate and reported in the manuscript?	Not Applicable	
If a study is subject to dual use research of concern regulations, is the name of the **authority granting approval and reference number** for the regulatory approval provided in the manuscript?	Not Applicable	

Reporting

The MDAR framework recommends adoption of discipline-specific guidelines, established and endorsed through community initiatives. Journals have their own policy about requiring specific guidelines and recommendations to complement MDAR.

Adherence to community standards	Information included in the manuscript?	In which section is the information available? (Reagents and Tools Table, Materials and Methods, Figures, Data Availability Section)
State if relevant guidelines or checklists (e.g., **ICMJE, MIBBI, ARRIVE, PRISMA**) have been followed or provided.	Not Applicable	
For **tumor marker prognostic studies,** we recommend that you follow the **REMARK** reporting guidelines (see link list at top right). See author guidelines, under 'Reporting Guidelines'. Please confirm you have followed these guidelines.	Not Applicable	
For **phase II and III randomized controlled trials**, please refer to the **CONSORT** flow diagram (see link list at top right) and submit the CONSORT checklist (see link list at top right) with your submission. See author guidelines, under 'Reporting Guidelines'. Please confirm you have submitted this list.	Not Applicable	

Data Availability

Data availability	Information included in the manuscript?	In which section is the information available? (Reagents and Tools Table, Materials and Methods, Figures, Data Availability Section)
Have **primary datasets** been deposited according to the journal's guidelines (see 'Data Deposition' section) and the respective accession numbers provided in the Data Availability Section?	Yes	Data availability section
Were **human clinical and genomic datasets** deposited in a public access-controlled repository in accordance to ethical obligations to the patients and to the applicable consent agreement?	Not Applicable	
Are **computational models** that are central and integral to a study available without restrictions in a machine-readable form? Were the relevant accession numbers or links provided?	Not Applicable	
If publicly available data were reused, provide the respective **data citations in the reference list**.	Yes	Reference list and Table EV2
